# Integrated analysis identifies a palmitoylation-associated prognostic model (ACSM5/SKA3) for lung adenocarcinoma across multiple cohorts

**DOI:** 10.7717/peerj.21160

**Published:** 2026-04-29

**Authors:** Chaopeng Wu, Jinsong Wang, Yihan Liu, Zihan Ni, Yujie Zhan, Wanying Feng, Jia Feng, Min Peng

**Affiliations:** Department of Oncology, Renmin Hospital of Wuhan University, Wuhan, China

**Keywords:** Lung adenocarcinoma, Protein palmitoylation, Prognosis, Tumor microenvironment, Immunotherapy

## Abstract

**Background:**

Lung adenocarcinoma (LUAD) is the most common subtype of non-small cell lung cancer and remains associated with poor prognosis despite therapeutic advances. Protein palmitoylation, particularly reversible S-palmitoylation, modulates oncogenic signaling, membrane localization, and immune checkpoint stability, but the prognostic landscape of palmitoylation-related genes (PRGs) in LUAD and their relationship with the tumor microenvironment are not well defined.

**Methods:**

PRGs were curated from GeneCards and integrated with transcriptomic and clinical data from The Cancer Genome Atlas Lung Adenocarcinoma (TCGA-LUAD) cohort. Differential expression and univariate Cox analyses were performed to identify prognostic PRGs, which were then used for consensus clustering to define molecular subtypes and to construct a LASSO-Cox prognostic model. Associations between the risk score and functional pathways, genomic alterations, tumor mutational burden (TMB), tumor immune dysfunction and exclusion (TIDE) scores, and immune infiltration were evaluated. Three Gene Expression Omnibus (GEO) cohorts were used for external validation. Single-cell RNA sequencing (scRNA-seq) datasets and reverse transcription-quantitative polymerase chain reaction (RT-qPCR) of paired LUAD and adjacent tissues were used to assess cell type–associated expression and validate key genes.

**Results:**

We identified 201 differentially expressed PRGs in LUAD and 15 genes significantly associated with overall survival, including the protective gene ACSM5 and the risk gene SKA3. PRG-based consensus clustering defined two molecular subtypes with distinct prognoses, differential enrichment of metabolism- versus proliferation-related pathways, and divergent immune microenvironment features. A two-gene signature comprising ACSM5 and SKA3 stratified patients into high- and low-risk groups in The Cancer Genome Atlas (TCGA) and three external cohorts, with time-dependent area under the curve (AUCs) of 0.717, 0.733, and 0.697 at 1, 3, and 5 years in TCGA and comparable performance in GEO validation cohorts (5-year AUC up to 0.778), indicating moderate discriminative performance. Low-risk patients generally showed higher stromal and immune scores and increased infiltration of multiple immune cell subsets, along with complex patterns in TMB and TIDE indices. Single-cell analyses indicated predominant expression of ACSM5 in macrophages and of SKA3 in proliferating CD8+ T cells and macrophages, and RT-qPCR confirmed downregulation of ACSM5 and upregulation of SKA3 in LUAD tissues.

**Conclusion:**

This integrated analysis outlines a palmitoylation-associated molecular framework in LUAD and supports an ACSM5/SKA3 two-gene signature as a candidate prognostic tool that stratifies patient outcomes and reflects distinct metabolic, proliferative, and immune microenvironmental states, potentially providing a palmitoylation-associated basis for risk stratification and for the design of future mechanistic and therapeutic studies in LUAD.

## Introduction

Lung cancer remains one of the most serious global public health challenges and the leading cause of cancer-related mortality worldwide (Bade & Dela Cruz, 2020; Bray et al., 2024). In 2022, lung cancer accounted for approximately 2.5 million new cases and 1.8 million deaths, representing about 12% of all newly diagnosed cancers and 19% of all cancer deaths. The Global Burden of Disease study further reported that tracheal, bronchus, and lung cancers together contributed around 46.5 million disability-adjusted life-years in 2021, underscoring their enormous impact on premature mortality and loss of healthy life years (Kuang et al., 2024; Lin et al., 2025). As the most common histological subtype of non-small cell lung cancer, lung adenocarcinoma (LUAD) accounts for a substantial proportion of the incidence and mortality burden of lung cancer. Despite continuous progress in low-dose computed tomography (CT) screening, surgical techniques, molecularly targeted therapies, and immune checkpoint inhibitors, survival outcomes for patients with advanced LUAD remain far from satisfactory (Miller & Hanna, 2021).

Over the past decade, large-scale genomic and transcriptomic studies have greatly deepened our understanding of the molecular heterogeneity of LUAD (Herbst, Morgensztern & Boshoff, 2018; Mamdani et al., 2022). Analyses of cohorts such as The Cancer Genome Atlas (TCGA) have defined transcriptomic subtypes including proximal-inflammatory, proximal-proliferative, and terminal respiratory unit, which reflect distinct oncogenic drivers and tumor microenvironmental characteristics (Cancer Genome Atlas Research Network, 2014). In parallel, the identification of driver gene alterations in EGFR, KRAS, ALK, ROS1, and others has propelled the development of targeted therapies and laid the foundation for precision oncology in LUAD (Mazieres et al., 2019; Tan & Tan, 2022). More recently, novel classification systems based on metabolic and immune features have further revealed the complexity of LUAD biology (Wang et al., 2025a; Yang et al., 2025). However, existing biomarker frameworks still cannot fully explain the marked variability in patient prognosis, accurately predict responses to immunotherapy, or integrate multi-dimensional molecular information. Thus, there remains a pressing clinical need to identify new prognostic biomarkers and to construct risk models that can complement current systems.

Post-translational modifications (PTMs) are crucial regulatory mechanisms that modulate protein stability, subcellular localization, and functional activity in cancer development and progression. Palmitoylation is a widespread lipid modification in which palmitic acid (C16:0) is covalently attached to specific amino acid residues of target proteins. Among the different forms, reversible S-palmitoylation is the most common and is catalyzed by the ZDHHC family of palmitoyl acyltransferases (“writers”) and removed by depalmitoylating enzymes (“erasers”) such as acyl-protein thioesterases 1/2 (APT1/2), *α*/*β*-hydrolase domain-containing proteins (*e.g.*, ABHD17), and palmitoyl-protein thioesterase 1 (PPT1) (Kleuss & Krause, 2003; Linder & Deschenes, 2007; Hernandez, Majmudar & Martin, 2013; Fan et al., 2024). This dynamic regulatory system participates in membrane localization, vesicular trafficking, protein–protein interactions, and protein degradation, thereby finely tuning signaling pathways and immune responses (Salaun, Greaves & Chamberlain, 2010; Mesquita et al., 2024).

Accumulating evidence indicates that dysregulated palmitoylation can drive malignant progression in multiple tumor types by stabilizing oncogenic proteins, sustaining proliferative signals, enhancing invasive and metastatic potential, and promoting immune evasion (Cuiffo & Ren, 2010; Zhang et al., 2021; Jin et al., 2024; Lu et al., 2025; Wang et al., 2025b). In LUAD, palmitoylation of KRAS and EGFR has been shown to enhance their membrane localization and signaling output, thereby promoting tumor cell growth, survival, and treatment resistance (Nussinov et al., 2017; Ali et al., 2018; Kharbanda et al., 2020; Zhou et al., 2025). In addition, palmitoylation of the immune checkpoint molecule PD-L1 can maintain its stability at the cell surface and attenuate antitumor immune responses (Von Knethen & Brüne, 2019). Several members of the ZDHHC family, such as ZDHHC11B and ZDHHC9, have also been implicated in epithelial-mesenchymal transition, tumorigenesis, and remodeling of the tumor immune microenvironment in LUAD (Li et al., 2023; Dai et al., 2023). Together, these findings suggest that palmitoylation-related genes (PRGs) may act as key hubs integrating intrinsic oncogenic signaling with the extrinsic immune milieu (Chen et al., 2025). However, systematic studies that comprehensively evaluate PRGs in LUAD in terms of prognosis, molecular subtypes, and immune characteristics are still lacking.

Based on this rationale, the present study systematically analyzed the expression patterns and prognostic value of PRGs in The Cancer Genome Atlas Lung Adenocarcinoma (TCGA-LUAD) cohort. We used consensus clustering to identify palmitoylation-related molecular subtypes and applied Cox and least absolute shrinkage and selection operator (LASSO) regression to construct a prognostic model, which was externally validated in multiple Gene Expression Omnibus (GEO) cohorts. We further explored the associations between the risk signature and the tumor immune microenvironment, genomic alterations, and potential therapeutic responses. Finally, by integrating bulk transcriptomic data, single-cell RNA sequencing (scRNA-seq) data, and reverse transcription-quantitative polymerase chain reaction (RT-qPCR) analysis of LUAD clinical samples, we characterized the cell type-specific expression and regulatory features of key PRGs, including ACSM5 and SKA3. This study aims to outline a palmitoylation-related molecular framework for LUAD and to propose a prognostic model that may assist risk stratification and individualized therapeutic strategies, particularly in the era of immunotherapy.

## Materials & Methods

### Dataset and preprocessing

To obtain a comprehensive set of palmitoylation-related genes (PRGs), we queried the GeneCards database (https://www.genecards.org/) using the keyword “palmitoylation” and extracted all protein-coding genes annotated as functionally associated with palmitoylation. A total of 2,183 genes were retrieved, of which 2,166 were present in the TCGA-LUAD expression matrix and were therefore included in this study as an annotation-based PRG set.

RNA sequencing data (FPKM, Fragments Per Kilobase Million) and corresponding clinical information for LUAD patients were downloaded from TCGA. FPKM values were transformed to TPM, followed by log2(TPM + 1) conversion and quantile normalization. Samples lacking complete clinical information or with overall survival (OS) <30 days were excluded, leaving 493 tumor and 59 normal lung samples for further analysis.

Three independent GEO cohorts (GSE30219, GSE31210, and GSE50081) were additionally obtained as external validation datasets. Probe IDs were mapped to official gene symbols according to platform annotations, and when multiple probes corresponded to the same gene, their average expression value was used.

### Differential expression and enrichment analysis

Differential expression of PRGs between LUAD and normal lung tissues in the TCGA cohort was analyzed using the “limma” R package (Me et al., 2015). Genes with FDR-adjusted *P*  <  0.05 and |log2 FC| > 1 were considered significantly differentially expressed. Heatmaps and volcano plots were generated to visualize the expression patterns of differentially expressed PRGs. Functional enrichment analyses, including Gene Ontology (GO), Kyoto Encyclopedia of Genes and Genomes (KEGG), and Disease Ontology (DO), were performed using the “clusterProfiler” R package to explore the biological processes and pathways associated with these genes (Yu et al., 2012).

### Univariate Cox regression analysis

Univariate Cox regression was conducted to identify differentially expressed genes significantly associated with OS using the “survival” R package. Genes with *P* <0.001 were selected as candidate prognostic genes.

### Copy number variation analysis

Copy number variation (CNV) of PRGs was assessed using the GISTIC algorithm to identify recurrent amplifications or deletions across the cohort (Mermel et al., 2011). CNV plots were generated to visualize genomic regions with significant gains or losses and their potential relevance to LUAD progression.

### Consensus clustering analysis

Unsupervised consensus clustering analysis was performed based on the expression of the candidate prognostic genes to classify LUAD samples into distinct molecular subtypes. Principal component analysis (PCA) was conducted to assess the separation between the identified subtypes. Consensus clustering was carried out using the “ConsensusClusterPlus” R package with the k-means algorithm, a random seed of 999,999 to ensure reproducibility, and 1,000 resampling iterations to evaluate clustering stability (Wilkerson & Hayes, 2010). The optimal number of clusters was determined to be two (*k* = 2). Kaplan–Meier survival analysis was applied to estimate OS differences between subtypes, and the log-rank test was used to assess statistical significance.

### Construction and validation of PRG-based prognostic signature

To further evaluate the prognostic significance of PRGs in LUAD, we developed a PRG-based risk score system using the TCGA dataset to predict patient prognosis and to explore potential associations with immunotherapeutic response. A prognostic signature was constructed through Cox regression analysis combined with the LASSO penalty method, based on the previously identified candidate genes. The risk score model was defined as: riskScore = Σ (coefi × expi). According to the median risk score, patients were stratified into high-risk and low-risk groups. Subsequently, survival analysis, PCA, and receiver operating characteristic (ROC) curve analyses were performed. Moreover, a nomogram was established to further assess individual prognosis. In addition, the proportional hazards assumption of Cox models was examined using Schoenfeld residuals. Model discrimination was quantified by Harrell’s concordance index (C-index). Model calibration was assessed by comparing predicted *versus* observed 1-, 3-, and 5-year OS probabilities using calibration curves. To validate the robustness of this signature, we applied it to external GEO datasets, which supported its predictive performance.

### Tumor microenvironment and immune characteristics analysis

The tumor microenvironment (TME) is a complex system composed of stromal fibroblasts, infiltrating immune cells, vascular networks, and noncellular components such as the extracellular matrix. To characterize TME features among molecular subtypes, the ESTIMATE algorithm was applied to evaluate non-tumor cell content, stromal and immune scores, and tumor purity (Yoshihara et al., 2013; Xu et al., 2025). Immune differences were further investigated by analyzing multiple categories of immune-related molecules, including chemokines, immunostimulators, immunoinhibitors, MHC molecules, and receptors. Associations between key immune checkpoints and risk scores were assessed using Spearman correlation. The relative abundance of immune cell types in the TME was quantified using single-sample gene set enrichment analysis (ssGSEA) (Hänzelmann, Castelo & Guinney, 2013; Xu et al., 2022). This integrated analysis provided a comprehensive overview of the cellular and molecular composition of the TME across different LUAD subgroups.

### Tumor mutation burden analysis

Somatic mutation data for LUAD patients were processed using the “maftools” R package. Tumor mutation burden (TMB) was calculated as the total number of nonsynonymous mutations per megabase for each sample (Chalmers et al., 2017). Differences in TMB between high- and low-risk groups were analyzed, and survival comparisons between high- and low-TMB groups were performed.

### Immunotherapy response prediction

The Tumor Immune Dysfunction and Exclusion (TIDE) algorithm was applied to evaluate T-cell dysfunction and exclusion, thereby estimating the potential for immune evasion in LUAD patients (Jiang et al., 2018). In this framework, higher TIDE scores reflect a greater likelihood of immune escape. The distribution of TIDE scores between the high- and low-risk groups was compared to assess differences in predicted immune evasion. Differences in the proportions of patients predicted as responders or non-responders were evaluated using the chi-square test.

### Drug sensitivity analysis

Drug sensitivity was predicted using the 2016 Cancer Genome Project dataset as implemented in the “pRRophetic” R package (Geeleher, Cox & Huang, 2014). Predicted responses to commonly used chemotherapeutic agents were calculated, and differences between high- and low-risk groups were compared.

### scRNA-seq data acquisition and analysis

scRNA-seq data for LUAD were obtained from the Tumor Immune Single-cell Hub 2 (TISCH2), including two cohorts: GSE127465 and GSE146100 (Han et al., 2023). All preprocessing, including quality control, normalization, batch correction, dimensionality reduction, clustering, and cell-type annotation, followed the standardized analytical pipelines implemented on the TISCH2 platform; the processed expression matrices were directly used for downstream visualization and comparison. Additionally, scRNA-seq data were retrieved from the Human Protein Atlas (HPA), and gene expression patterns were examined using the built-in analytical modules provided by the HPA database to validate the expression of the target genes across different cell types (Karlsson et al., 2021).

### RT-qPCR validation

Expression levels of ACSM5 and SKA3 were validated using RT-qPCR. 10 paired LUAD and adjacent normal tissue samples were collected. Total RNA was extracted with TRIzol and reverse-transcribed using PrimeScript RT Master Mix. SYBR Green Master Mix was used for amplification. Each reaction was performed in triplicate with a 20 μL volume. Primer sequences were as follows:

ACSM5, forward 5′-CGCCCATGATGTGCTGGAT-3′,

reverse 5′-CTGTGCCATTGACCCACCA-3′;

SKA3, forward 5′-TACACGAGCAAGAAGCCATTAAC-3′,

reverse 5′-GGATACGATGTACCGCTCAAGT-3′;

GAPDH, forward 5′-GATTCCACCCATGGCAAATTC-3′,

reverse 5′-GTCATGAGTCCTTCCACGATAC-3′.

Relative expression was calculated using the 2ˆ−ΔΔCt method, and differences between paired samples were assessed using paired t-tests.

### Ethics approval and consent to participate

The study was conducted in accordance with the Declaration of Helsinki and was reviewed and approved by the Ethics Committee of Renmin Hospital of Wuhan University before the investigation. The ethical approval number was WDRY2024-K078 (dated April 9, 2024). The Ethics Committee granted a waiver of informed consent for this study.

### Statistical analysis

All statistical analyses were conducted using R (version 4.4.1; R Core Team, 2024). Unless otherwise specified, a two-sided *P* < 0.05 was considered statistically significant. For analyses involving multiple comparisons, nominal *P* values were adjusted using the Benjamini–Hochberg procedure, and results were considered significant at an FDR-adjusted *q* < 0.05 where applicable.

## Results

### Expression profiles and prognostic analysis of PRGs in LUAD

**Figure 1:**
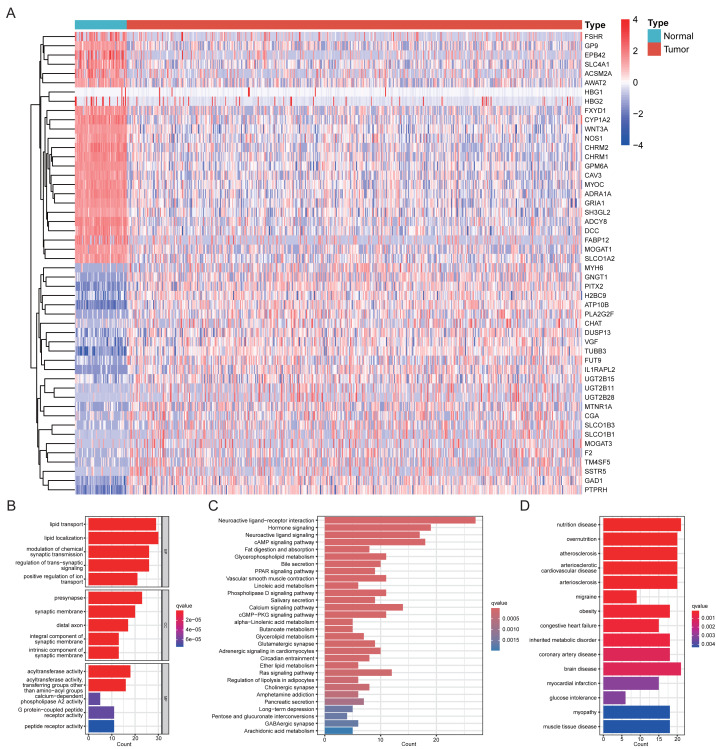
Identification and functional enrichment of differentially expressed PRGs in LUAD. (A) Heatmap showing differentially expressed PRGs between LUAD and normal lung tissues. (B) GO enrichment analysis. (C) KEGG pathway analysis. (D) DO enrichment analysis.

We retrieved protein-coding genes annotated as palmitoylation-related in GeneCards and intersected them with the TCGA-LUAD expression matrix, yielding 2,166 putative PRGs for analysis. After excluding cases with incomplete clinical information or OS < 30 days, 493 LUAD tumor samples and 59 normal lung samples were included in the TCGA cohort. Differential expression analysis (FDR < 0.05 and |log2 FC| > 1) identified 201 significantly dysregulated PRGs between tumor and normal tissues, including 105 downregulated and 96 upregulated genes (Fig. 1A) (Fig. S1A). GO enrichment analysis showed that these differentially expressed PRGs were mainly enriched in lipid transport and metabolism, synaptic transmission, and signal transduction (Fig. 1B). Kyoto Encyclopedia of Genes and Genomes (KEGG) pathway analysis indicated involvement in neuroactive ligand–receptor interaction, hormonal signaling pathways, and lipid metabolic processes (Fig. 1C). DO enrichment analysis further suggested associations with metabolic disorders such as obesity, glucose intolerance, and inherited metabolic diseases, as well as cardiovascular diseases including atherosclerosis, coronary artery disease, myocardial infarction, and congestive heart failure (Fig. 1D).

**Figure 2:**
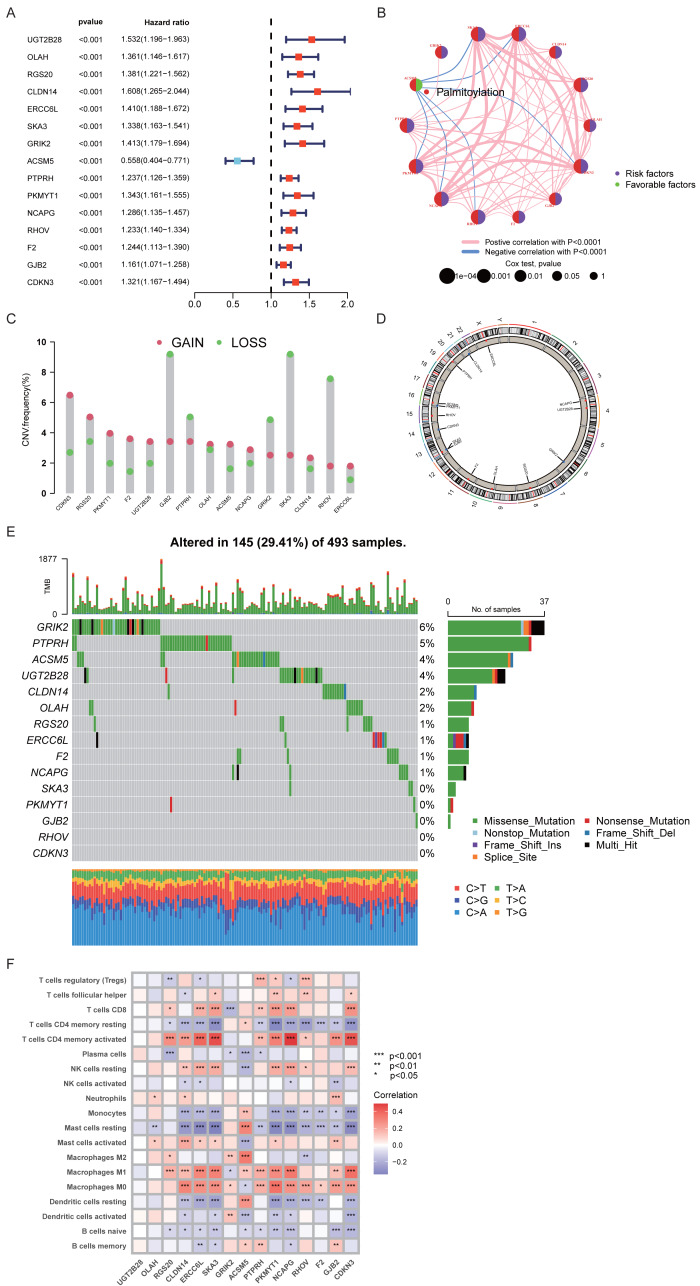
Prognostic significance and molecular characteristics of PRGs in LUAD. (A) Univariate Cox regression analysis of overall survival-related genes. (B) Correlation network among prognostic genes. (C) CNV analysis. (D) Chromosome distribution of prognostic genes. (E) Gene mutation landscape. (F) Correlation between prognostic gene expression and immune cell infiltration.

Based on univariate Cox regression analysis (*P* < 0.001), 15 PRGs were identified as significantly associated with OS in LUAD patients. Among them, ACSM5 was classified as a protective gene (HR < 1), whereas the remaining 14 genes, including SKA3, were considered risk genes (HR > 1) (Fig. 2A). Correlation network analysis revealed multiple intergene correlations among these prognostic PRGs (Fig. 2B). Further analysis indicated that these genes frequently exhibited CNVs, including both amplifications and deletions (Fig. 2C), and were distributed across different chromosomal regions (Fig. 2D). In addition, somatic mutation analysis showed that these genes harbored mutations in 29.41% of LUAD samples, with the highest mutation frequencies observed in GRIK2, PTPRH, and ACSM5 (Fig. 2E). Immune infiltration analysis showed that the expression levels of these prognostic PRGs were significantly correlated with multiple immune cell subsets, including T cells, macrophages, and dendritic cells, suggesting potential links between these genes and the tumor immune microenvironment (Fig. 2F).

### LUAD subtyping based on prognostic genes and characterization of subtypes

**Figure 3:**
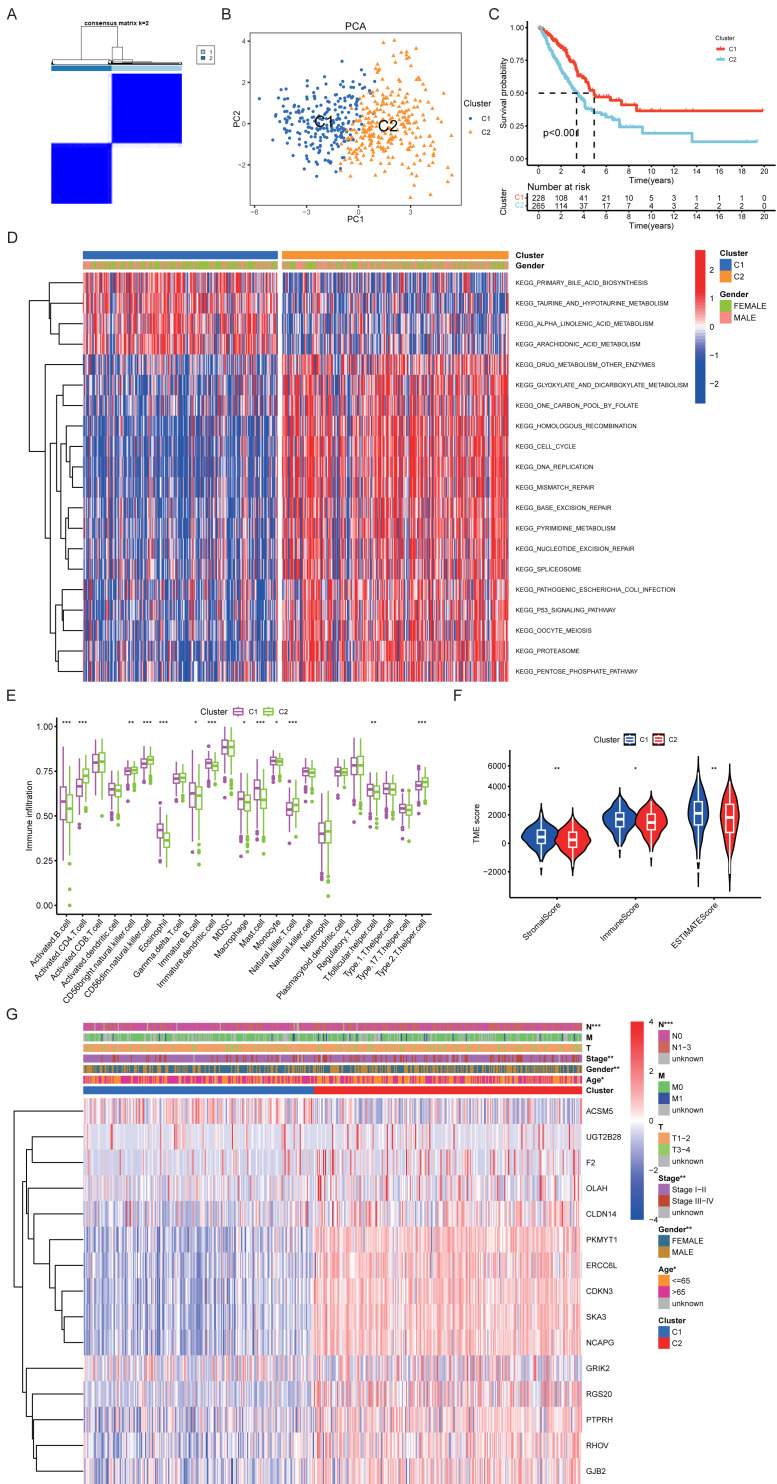
Consensus clustering of prognostic PRG s and characterization of LUAD subtypes. (A) Consensus clustering analysis identifying two distinct subtypes (C1 and C2). (B) PCA validating the separation of C1 and C2. (C) Kaplan–Meier (KM) survival analysis. (D) KEGG pathway analysis. (E) Immune cell infiltration patterns in C1 and C2. (F) TME differences between subtypes. (G) Associations of C1 and C2 with gene expression and clinical characteristics.

Using the expression profiles of the 15 prognostic PRGs, consensus clustering was performed on 493 TCGA-LUAD samples. The clustering results suggested that *k* = 2 provided relatively high within-cluster consistency and between-cluster distinction (Fig. 3A), which was supported by the CDF curves and delta area analysis (Figs. S1B, S1C). PCA further showed a clear separation between the two clusters, which were designated as C1 and C2 (Fig. 3B). Survival analysis indicated that patients in the C1 subtype had significantly better OS than those in the C2 subtype (*P* < 0.001) (Fig. 3C).

KEGG pathway analysis showed that the C1 subtype was mainly enriched in metabolism-related pathways, including primary bile acid biosynthesis, taurine metabolism, and fatty acid metabolism, whereas the C2 subtype was enriched in pathways related to DNA replication, cell cycle regulation, and DNA repair (Fig. 3D). Immune infiltration analysis suggested that the C1 subtype had higher estimated infiltration of activated B cells, eosinophils, and mast cells, while the C2 subtype showed higher estimated levels of activated CD4^+^ T cells, natural killer T cells, and type 2 helper T cells (Fig. 3E). In addition, the two subtypes exhibited differences in the proportions of tumor microenvironment components (Fig. 3F).

At the gene expression level, ACSM5 was more highly expressed in the C1 subtype, whereas genes such as CDKN3, NCAPG, and SKA3 were upregulated in the C2 subtype. The two subtypes also differed in several clinical characteristics, including tumor stage, sex, and age (*P* < 0.05) (Fig. 3G).

**Figure 4:**
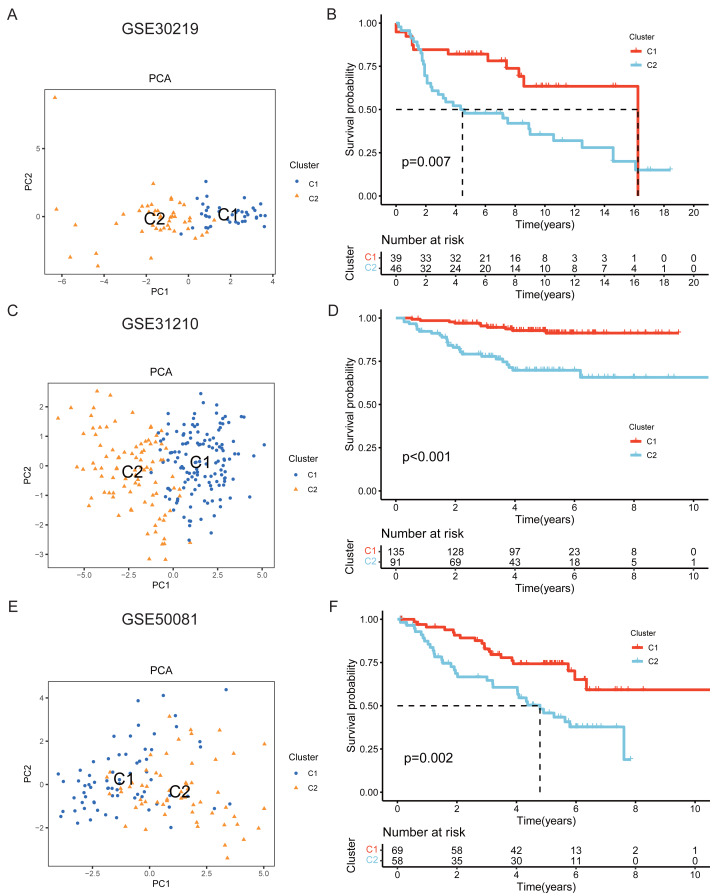
External validation of LUAD subtypes in GEO cohorts. (A, C, E) PCA showing separation of C1 and C2 subtypes in the GSE30219, GSE31210, and GSE50081 cohorts, respectively. (B, D, F) Kaplan–Meier (KM) survival curves between C1 and C2 subtypes in the GSE30219, GSE31210, and GSE50081 cohorts, respectively.

The clustering-based subtyping approach was further applied to the external validation cohorts GSE30219, GSE31210, and GSE50081. PCA similarly showed a distinct separation between the two subtypes in each cohort (Figs. 4A, 4C, 4E), and the survival advantage of the C1 subtype was consistently observed across all three cohorts (*P* < 0.01) (Figs. 4B, 4D, 4F).

### Construction and multi-cohort validation of the prognostic model

**Figure 5:**
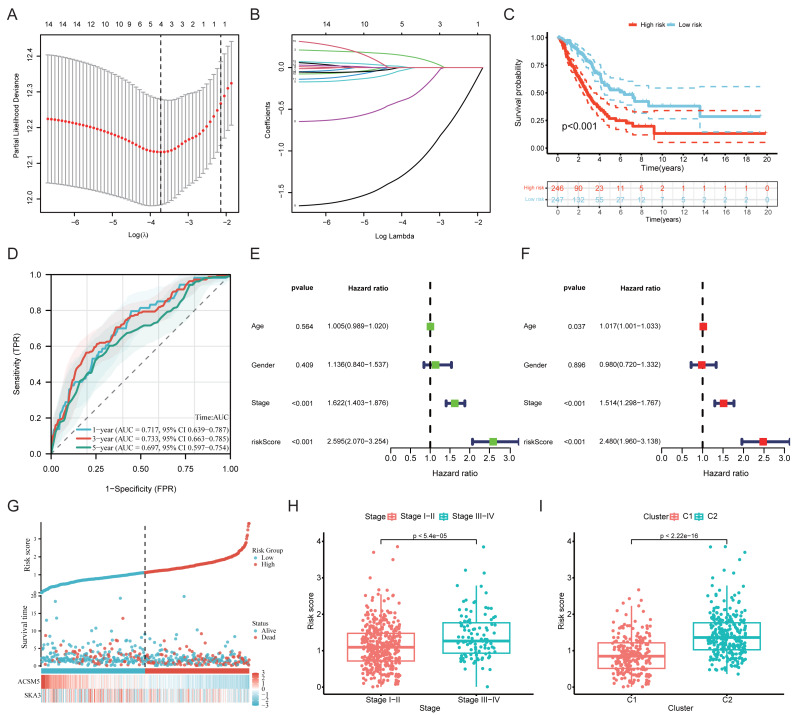
Construction and evaluation of a prognostic risk model based on SKA3 and ACSM5 in LUAD. (A) Cross-validation of the LASSO regression. (B) Coefficient value of prognostic genes. (C) Kaplan–Meier (KM) survival analysis. (D) ROC curves of risk score. (E) Univariate Cox regression analysis. (F) Multivariate Cox regression analysis. (G) Distribution of patient risk scores, survival status, and survival time. (H) Comparison of risk scores between early-stage and advanced-stage patients. (I) Distribution of risk scores between C1 and C2 subtypes.

To further evaluate the prognostic relevance of the 15 genes in LUAD, a LASSO-Cox regression analysis was performed using the TCGA-LUAD dataset. Based on this analysis, a two-gene prognostic model incorporating SKA3 and ACSM5 was constructed, and their individual prognostic value is shown in Figs. S1D, S1E. Patients were stratified into high- and low-risk groups based on the median risk score (Figs. 5A, 5B). Kaplan–Meier survival analysis showed that patients in the high-risk group had significantly worse OS than those in the low-risk group (*P* < 0.001) (Fig. 5C). ROC curve analysis further evaluated the predictive performance of the model, with AUC values of 0.717 (95% CI [0.639–0.787]), 0.733 (95% CI [0.663–0.785]), and 0.697 (95% CI [0.597–0.754]) at 1, 3, and 5 years, respectively, indicating moderate discriminative ability (Fig. 5D). Calibration curves for 1-, 3-, and 5-year OS are shown in Fig. S1F.

As the risk score increased, patient survival time gradually decreased and the number of death events increased (Fig. 5G). Both univariate and multivariate Cox regression analyses indicated that the risk score was independently associated with OS in LUAD patients (Fig. 5E, Fig. 5F). Moreover, patients with advanced-stage disease (stage III-IV) exhibited significantly higher risk scores than those with early-stage disease (stage I-II) (*P* < 0.001) (Fig. 5H). Consistently, in the consensus clustering analysis, the C1 subtype had significantly lower risk scores than the C2 subtype (*P* < 0.001) (Fig. 5I), indicating that the two classification strategies were broadly aligned in their prognostic trends.

**Figure 6:**
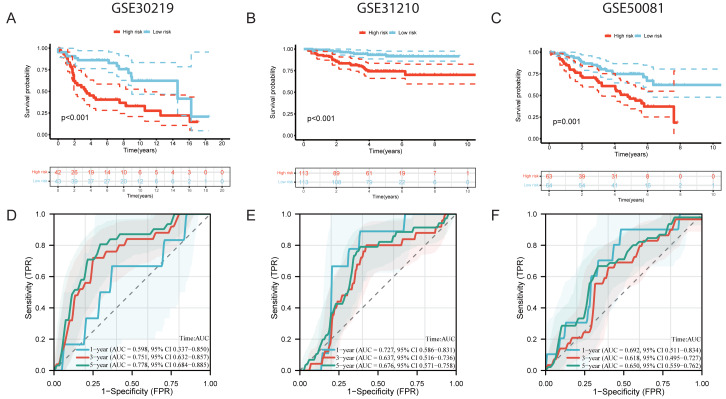
Independent validation of the two-gene prognostic model in GEO cohorts. (A–C) Kaplan–Meier (KM) survival analysis comparing high- and low-risk groups in the GSE30219, GSE31210, and GSE50081 cohorts, respectively. (D–F) ROC curves assessing the predictive performance of the risk model at 1, 2, and 3 years in the GSE30219, GSE31210, and GSE50081 cohorts, respectively.

To further assess the generalizability of the model, independent validation was performed in three external cohorts: GSE30219, GSE31210, and GSE50081. Using the median risk score as the cutoff, patients in each cohort were divided into high- and low-risk groups. Kaplan–Meier survival curves consistently demonstrated that high-risk patients had significantly worse OS than low-risk patients (*P* < 0.001) (Figs. 6A–6C). ROC curve analysis showed that the AUC values at 1, 3, and 5 years were 0.598 (95% CI [0.337–0.850]), 0.751 (95 CI [0.632–0.857]), and 0.778 (95% CI [0.684–0.885]) for the GSE30219 cohort; 0.727 (95% CI [0.586–0.831]), 0.637 (95% CI [0.516–0.736]), and 0.676 (95% CI [0.571–0.758]) for the GSE31210 cohort; and 0.692 (95% CI [0.511–0.834]), 0.618 (95% CI [0.495–0.727]), and 0.650 (95% CI [0.559–0.762]) for the GSE50081 cohort (Figs. 6D–6F).

### Gene mutation, functional pathways, and immune microenvironment features

**Figure 7:**
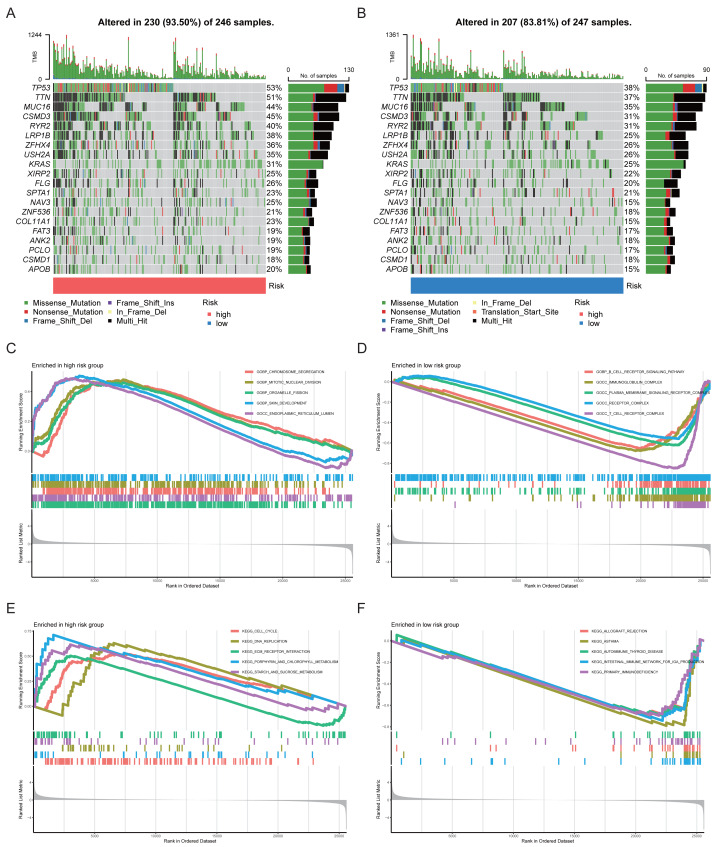
Mutation landscape and functional enrichment analysis of high- and low-risk groups in LUAD. (A, B) Mutational profiles of the top 20 frequently mutated genes in high- and low-risk groups. (C, D) GO enrichment analysis in the high- and low-risk groups, respectively. (E, F) KEGG pathway analysis in high- and low-risk groups, respectively.

To evaluate the mutation characteristics of the high- and low-risk groups, the mutational profiles of the 20 most frequently mutated genes were analyzed (Figs. 7A, 7B). GSEA revealed significant differences in functions and pathways between the two risk groups. GO enrichment analysis showed that the high-risk group was predominantly enriched in pathways related to cell proliferation and tumor progression (Fig. 7C), whereas the low-risk group was mainly enriched in immune-related pathways (Fig. 7D). KEGG pathway analysis further indicated that the high-risk group was enriched in pathways associated with tumor proliferation, metastasis, and infiltration, while the low-risk group was enriched in pathways related to immune diseases (Figs. 7E, 7F), consistent with the GO analysis results.

**Figure 8:**
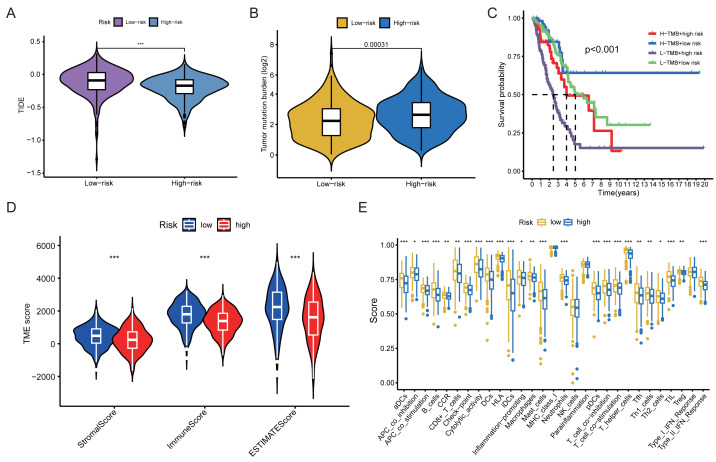
Immunological characteristics and TME analysis of high- and low-risk groups in LUAD. (A) Comparison of TIDE scores between high- and low-risk groups. (B) Comparison of TMB scores between high- and low-risk groups. (C) Kaplan–Meier (KM) survival analysis stratified by combined TMB and risk groups. (D) TME scores between high- and low-risk groups. (E) Immune cell infiltration profiles in high- and low-risk groups.

Further analysis showed that the TIDE scores of the low-risk group were significantly higher than those of the high-risk group (*P* < 0.001) (Fig. 8A), whereas TMB was significantly lower in the low-risk group (*P* < 0.001) (Fig. 8B). In the TIDE framework, higher scores reflect stronger T-cell dysfunction or exclusion and are generally associated with a lower likelihood of response to immune checkpoint blockade, whereas higher TMB has been linked to improved immunotherapy outcomes in several cancer types. In our cohort, however, the low-risk group defined by the ACSM5/SKA3 signature showed higher immune and stromal scores and greater immune-cell infiltration but a TIDE-high/TMB-low pattern, whereas the high-risk group exhibited the opposite pattern. When patients were further stratified into four subgroups according to both TMB and risk score (H-TMB + high risk, H-TMB + low risk, L-TMB + high risk, and L-TMB + low risk), the H-TMB + low-risk subgroup showed the most favorable survival, whereas the L-TMB + high-risk subgroup had the poorest survival (*P* < 0.001) (Fig. 8C). Tumor microenvironment analysis showed that the low-risk group had higher stromal, immune, and ESTIMATE scores and lower tumor purity than the high-risk group (Fig. 8D). Consistently, most immune cell populations were more abundantly infiltrated in the low-risk group than in the high-risk group (Fig. 8E). Together, these data highlight an apparent discordance between immune-cell infiltration and TIDE/TMB-derived indices within this cohort and suggest that their implications for immunotherapy response should be interpreted with caution.

**Figure 9:**
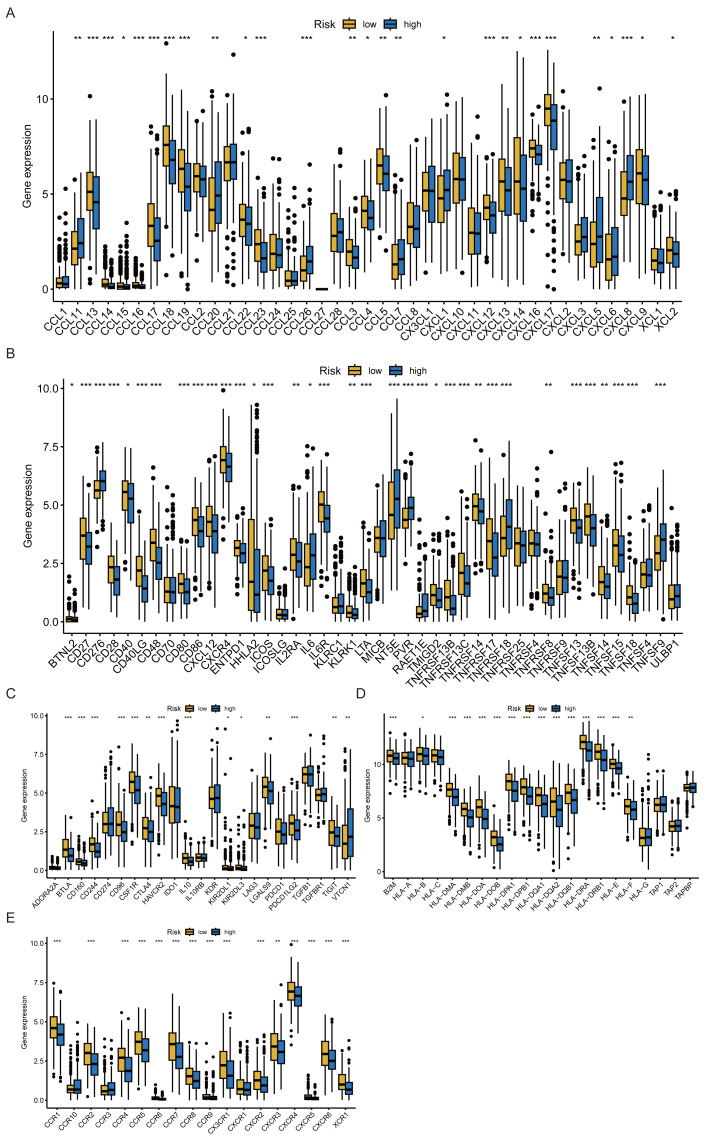
Immune-related molecular characteristics. (A–E) Expression of chemokines, immunostimulators, immunoinhibitors, MHC molecules, and receptors in high- and low-risk groups.

To further explore the immunological differences between the risk groups, we systematically analyzed the expression of chemokines, immune stimulatory factors, immune inhibitory factors, MHC molecules, and chemokine receptors (Figs. 9A–9E). Most immune-related molecules were significantly upregulated in the low-risk group, consistent with a more active immune microenvironment in this group.

### Drug sensitivity analysis of the prognostic model

We evaluated the relative drug sensitivity of the high- and low-risk groups, and the overall results of the predicted drug responses are summarized in Fig. S2. Furthermore, we examined the correlations between gene expression levels and drug sensitivity and highlighted the 16 gene-drug pairs with the highest correlation coefficients (Fig. S3).

### Gene expression, palmitoylation-related regulation, and immune feature analysis

To further investigate potential links between ACSM5/SKA3 and palmitoylation-related processes in LUAD, we first analyzed expression correlations between putative palmitoylation regulators (members of the ZDHHC, ABHD, and LYPLA families) and ACSM5 or SKA3. The results showed that the risk gene SKA3 was significantly negatively correlated with most ZDHHC family members (*r* = −0.46 to −0.20, *P* < 0.001) and positively correlated with a few ABHD and LYPLA genes (*r* = 0.18–0.27, *P* < 0.001). In contrast, the protective gene ACSM5 was mainly positively correlated with certain ZDHHC and ABHD genes (*r* = 0.12–0.18, *P* < 0.05) (Table S1). Overall, these modest but consistent correlation patterns between ACSM5/SKA3 and palmitoylation regulators are in line with their opposite prognostic associations and are compatible with a possible connection to palmitoylation-related pathways.

**Figure 10:**
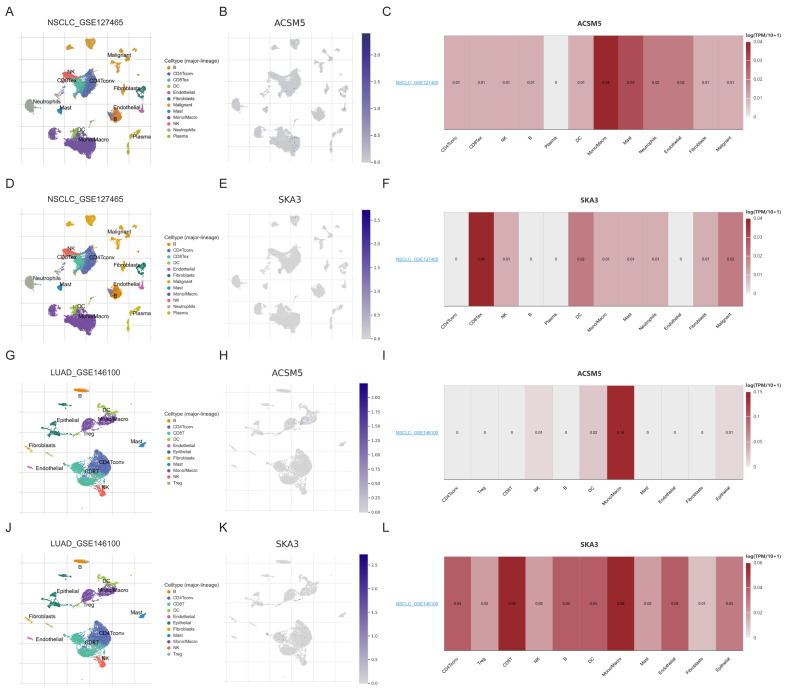
Single-cell RNA-seq analysis of ACSM5 and SKA3 expression in LUAD. (A–L) Dimensionality reduction and clustering of single-cell RNA-seq data from GSE127465 and GSE146100 cohorts, showing cell-type-specific expression of ACSM5 and SKA3.

Subsequently, scRNA-seq data were subjected to dimensionality reduction and clustering analysis using Seurat, and cell distributions were examined in two cohorts: GSE127465 (untreated) and GSE146100 (post-pembrolizumab treatment). The results indicated that ACSM5 was predominantly expressed in macrophages, whereas SKA3 was mainly expressed in CD8^+^ T cells and macrophages (Figs. 10A–10L), revealing cell type-associated expression patterns. These findings were further supported by single-cell transcriptomic data from the HPA database (Fig. S4).

**Figure 11:**
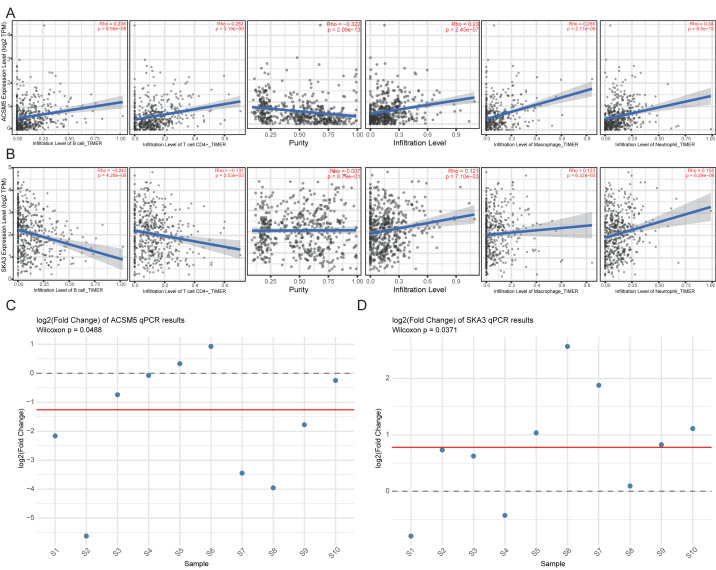
Immune correlation and experimental validation of ACSM5 and SKA3 in LUAD. (A) Correlation analysis between ACSM5 and immune cell infiltration. (B) Correlation analysis between SKA3 expression and immune cell infiltration. (C) qPCR validation of ACSM5 expression. (D) qPCR validation of SKA3 expression.

Immune correlation analysis revealed that ACSM5 was significantly positively correlated with B cells, CD4^+^ T cells, macrophages, and neutrophils and negatively correlated with tumor purity (Fig. 11A). In contrast, SKA3 showed weaker correlations with most immune cell populations and tumor purity (Fig. 11B).

Finally, we evaluated ACSM5 and SKA3 expression in paired tumor and adjacent normal samples from a small validation cohort of 10 LUAD patients. RT-qPCR results showed that ACSM5 expression was significantly lower in tumor tissues than in normal tissues (*P* = 0.048), whereas SKA3 expression was significantly higher in tumor tissues (*P* = 0.037) (Figs. 11C, 11D). Although based on a limited sample size and modest effect sizes, these findings provide preliminary experimental support for the differential expression patterns of ACSM5 and SKA3 observed in the public datasets.

## Discussion

In this study, we systematically analyzed the expression profiles and clinical significance of PRGs in LUAD. Our results revealed that 201 differentially expressed PRGs were significantly enriched in pathways related to lipid metabolism, neuroactive ligand–receptor interaction, and signal transduction, and were enriched for disease terms involving metabolic disorders such as obesity, abnormal glucose metabolism, and cardiovascular diseases. These findings are consistent with previous studies emphasizing the critical role of lipid metabolic dysregulation in tumorigenesis and progression, suggesting that PRGs may contribute to LUAD development at least in part through metabolic pathways (Luo et al., 2018; Martin-Perez et al., 2022; Ke et al., 2025; Zhang et al., 2025).

Through Cox regression analysis, we further identified 15 key genes significantly associated with OS, among which ACSM5 functioned as a protective factor, whereas most others, including SKA3, acted as risk factors. Notably, these genes exhibited frequent CNVs and somatic mutations and showed significant correlations with immune cell infiltration, suggesting potential roles in tumor progression and immune regulation. Subtype analysis based on the 15 genes revealed two molecular subtypes of LUAD: the C1 subtype, enriched in lipid metabolism-related pathways and characterized by a favorable prognosis and distinctive immune features; and the C2 subtype, associated with cell cycle and DNA repair pathways and exhibiting poorer prognosis together with enhanced immune cell infiltration and elevated expression of immune checkpoint molecules. These patterns resemble previously reported “metabolic” *versus* “proliferative” LUAD subtypes and collectively indicate that PRGs may participate in both metabolic and proliferative programs and are closely linked to remodeling of the tumor immune microenvironment (Eisenberg et al., 2020; Ma et al., 2020).

Based on these findings, we employed a LASSO-Cox regression analysis to identify ACSM5 and SKA3, which were used to construct a two-gene prognostic model. This model demonstrated reproducible predictive performance in both the TCGA cohort and multiple external GEO datasets, extending prior work that had already implicated ACSM5 and SKA3 individually as prognostic markers in LUAD and other malignancies. ACSM5, a member of the acyl-CoA synthetase family, primarily participates in fatty acid metabolism. In hepatocellular carcinoma, ACSM5 has been reported to exert antitumor effects by promoting ferroptosis, regulating lipid metabolism, and inhibiting the STAT3 signaling pathway (Yang et al., 2024; Wu et al., 2025b), whereas in LUAD and colorectal cancer its functions have mainly been linked to prognostic evaluation and modulation of the immune microenvironment (Parsazad et al., 2023; Ma, 2024; Fang et al., 2025). Taken together, current evidence suggests that ACSM5 may function as a metabolism-related tumor suppressor and prognostic biomarker in several contexts, although its causal role in LUAD progression remains to be clarified. In contrast, SKA3, a spindle- and kinetochore-associated protein, plays a central role in cell cycle regulation and mitosis. Overexpression of SKA3 has been reported to drive abnormal proliferation, migration, and malignant progression in various cancers (Li et al., 2025). In LUAD, SKA3 promotes cell proliferation and migration through activation of the EGFR pathway (Hu et al., 2020; Zheng et al., 2025), enhances glycolysis and chemoresistance (Chen et al., 2023; Liu et al., 2024), and is subject to negative regulation by specific microRNAs (Xie et al., 2022). These observations indicate that SKA3 exerts multifaceted functions in tumor progression, with its high expression consistently associated with unfavorable prognosis and representing a candidate biomarker and potential therapeutic target that warrants further experimental evaluation.

Importantly, neither ACSM5 nor SKA3 has yet been demonstrated to be directly S-palmitoylated or to act as a core enzymatic regulator of palmitoylation. In this study, “palmitoylation-related genes” were defined based on curated database annotations and pathway enrichment, as well as their correlations with established palmitoylation machinery and lipid-metabolism-related processes, rather than on direct biochemical evidence of palmitoylation. Therefore, the ACSM5/SKA3 model should be regarded as a palmitoylation-associated prognostic signature that captures pathways linked to palmitoylation dynamics, rather than as proof that these two genes themselves are palmitoylated effectors. Future functional studies will be required to determine whether ACSM5, SKA3, or their upstream/downstream partners are bona fide substrates or regulators within palmitoylation networks.

Immunological analyses revealed significant differences in the TME and immune characteristics between the high- and low-risk groups defined by the ACSM5/SKA3 model. The low-risk group exhibited higher immune and stromal scores, greater infiltration of multiple immune cell subsets, and significant upregulation of immune-related molecules, including chemokines, immune stimulatory factors, and MHC molecules, suggesting a more inflamed and immunologically active TME. In contrast, the high-risk group was enriched in pathways associated with tumor proliferation, metastasis, and invasion and displayed a higher TMB. While increased TMB has been associated with improved response to immune checkpoint inhibitors (ICIs) in some cancer types, TIDE analysis in our study indicated that the low-risk group had higher TIDE scores than the high-risk group, reflecting stronger T-cell dysfunction or exclusion and thus a lower predicted likelihood of benefiting from ICIs. This apparent discordance between TMB and TIDE, as well as between immune cell infiltration and predicted immune escape, underscores the complexity of the LUAD immune microenvironment and suggests that our signature is not yet suitable for guiding immunotherapy decision-making. Rather, these findings should be interpreted as hypothesis-generating and highlight the need for prospective, treatment-annotated cohorts to clarify how ACSM5/SKA3-defined risk groups relate to actual clinical responses to immunotherapy (Liu et al., 2025).

Single-cell transcriptomic analysis further revealed distinct patterns of ACSM5 and SKA3 expression at the cellular level. ACSM5 was predominantly expressed in macrophages and positively correlated with B cells, CD4^+^ T cells, macrophages, and neutrophils, suggesting that it may be involved in shaping the tumor immune microenvironment, for example by modulating macrophage or myeloid cell function. In contrast, SKA3 was mainly distributed in CD8^+^ T cells and macrophages but showed weaker correlations with immune features. Given that SKA3 is a canonical cell cycle-associated gene and that the high-risk group was consistently enriched for proliferation-related programs, its dominant contribution in LUAD is likely driven primarily by proliferating malignant cells rather than immune compartments alone. Accordingly, the SKA3 signal observed in immune subsets may reflect cycling immune subpopulations and/or technical factors. RT-qPCR experiments further validated the downregulation of ACSM5 and the upregulation of SKA3 in LUAD tissues, consistent with database analyses, thereby supporting their reliability as potential biomarkers at the transcript level. Nevertheless, the small sample size and lack of protein-level and functional validation mean that these findings should be interpreted cautiously.

Several recent studies have focused on palmitoylation in LUAD and other cancers, including ZDHHC5-based prognostic models, single-cell palmitoylation landscape analyses, and pan-cancer evaluations of the ZDHHC enzyme family as potential biomarkers and therapeutic targets (Bian et al., 2024; Lu et al., 2025; Wei et al., 2025; Wang et al., 2025b; Wu et al., 2025a). Compared with these ZDHHC-centered approaches, which primarily interrogate the “writer” enzymes of palmitoylation, our work starts from a broader set of palmitoylation-related expression profiles, identifies molecular subtypes, and distills a minimal two-gene signature that links lipid metabolism, cell-cycle programs, and immune features in LUAD. Thus this study complements existing ZDHHC-focused research by providing a palmitoylation-associated prognostic framework that can be integrated with future mechanistic and single-cell studies of palmitoylation.

Palmitoylation, as a dynamic PTM, likely interacts with other modifications such as phosphorylation and ubiquitination to regulate tumor progression. For example, ZDHHC20-mediated palmitoylation of fatty acid synthase regulates protein stability and lipid metabolism by competing with the E3 ubiquitin ligase complex SNX8-TRIM28 in the ubiquitin-proteasome pathway (Mo et al., 2024); palmitoylation-driven ubiquitination of PHF2 remodels lipid metabolism through the SREBP1c axis and contributes to hepatocellular carcinoma progression (Jeong et al., 2023); and non-palmitoylated ERK2 exhibits distinct phosphorylation patterns that affect downstream transcriptional programs (Azizi et al., 2023). In addition, recent work has highlighted palmitoylation of immune-related molecules, such as PD-L1 and interferon receptor components, as critical regulators of antitumor immunity and response to ICIs (Yao et al., 2019; Du et al., 2021). These findings collectively suggest that palmitoylation exerts multifaceted roles in tumor metabolism, signal transduction, and immune regulation and provide a conceptual basis for our observation that a palmitoylation-associated signature is closely linked to both metabolic pathways and the TME in LUAD.

This study has several limitations. First, it mainly relied on retrospective data from public databases and a relatively small RT-qPCR cohort of 10 paired LUAD and adjacent non-tumor tissues, without protein-level or in-depth functional validation, which may introduce bias and limit the generalizability of the findings. Second, the definition of PRGs and the association between ACSM5/SKA3 and palmitoylation were inferred from bioinformatics annotations and correlation analyses with known palmitoylation-related genes rather than from direct biochemical evidence, so the proposed signature should be regarded as palmitoylation-associated and hypothesis-generating. Third, the observed discordance between TMB- and TIDE-based predictions indicates that the ability of the ACSM5/SKA3 model to predict actual responses to immunotherapy remains uncertain and needs to be further evaluated in prospective, treatment-annotated cohorts and mechanistic studies.

In summary, our study proposes a palmitoylation-associated prognostic signature based on ACSM5 and SKA3, identifies two PRG-based LUAD molecular subtypes, and links this signature to distinct metabolic, proliferative, and immune microenvironmental features. While these findings strengthen the evidence that lipid metabolism and palmitoylation-related pathways are intertwined with LUAD progression and immune regulation, they should be viewed primarily as hypothesis-generating. Future studies integrating multi-omics profiling, biochemical validation of palmitoylation events, and mechanistic experiments *in vitro* and *in vivo*, as well as prospective clinical cohorts receiving targeted therapy or immunotherapy, will be essential to unravel the precise molecular mechanisms and to determine whether ACSM5/SKA3-based signatures can be translated into clinically useful tools for risk stratification and therapeutic decision-making in LUAD.

## Conclusion

This study systematically characterized putative palmitoylation-related genes in LUAD, delineating their dysregulated expression, molecular subgroups, prognostic associations, and links with immune-related features. Using ACSM5 and SKA3, we constructed a two-gene prognostic model that stratifies patients by overall survival across TCGA and three external cohorts and is associated with distinct molecular and tumor microenvironment characteristics. These correlative findings support ACSM5 and SKA3 as palmitoylation-associated prognostic biomarker candidates and may provide an initial palmitoylation-associated framework for risk stratification in LUAD.

## Supplemental Information

10.7717/peerj.21160/supp-1 Supplemental Information 1 Raw data

10.7717/peerj.21160/supp-2 Supplemental Information 2 MIQE checklist

10.7717/peerj.21160/supp-3 Supplemental Information 3 Code

10.7717/peerj.21160/supp-4 Supplemental Information 4 Correlation analysis between palmitoylation regulators and ACSM5 or SKA3 in LUAD

10.7717/peerj.21160/supp-5 Supplemental Information 5 Supplemental Figures

10.7717/peerj.21160/supp-6 Supplemental Information 6 Complete list of TCGA-LUAD case IDs

## Additional Information and Declarations

### Data Availability

The following information was supplied regarding data availability: The raw data is available in the Supplemental Files. The TCGA data is available at the GDC data portal: https://portal.gdc.cancer.gov/projects/TCGA-LUAD. - The complete list of 577 TCGA-LUAD case IDs is available in the Supplemental Files. The datasets are available at GEO: GSE30219, GSE31210, and GSE50081.
