# Supplementary material for: Integrated analysis identifies a palmitoylation-associated prognostic model (ACSM5/SKA3) for lung adenocarcinoma across multiple cohorts
Source: PeerJ. 2026 Apr 29;14:e21160. doi: 10.7717/peerj.21160 (PMC13135332; doi:10.7717/peerj.21160)
Supplement: Supplemental Information 4 [file peerj-14-21160-s004.docx]

|  |  | cor | p |
| --- | --- | --- | --- |
| ZDHHC1 | ACSM5 | 0.0608699 | 0.178554527 |
| ZDHHC2 | ACSM5 | 0.115073066 | 0.010796463 |
| ZDHHC3 | ACSM5 | 0.218836707 | 1.00E-06 |
| ZDHHC4 | ACSM5 | -0.106649888 | 0.018200926 |
| ZDHHC5 | ACSM5 | -0.0037353 | 0.934270185 |
| ZDHHC6 | ACSM5 | -0.041227547 | 0.362470826 |
| ZDHHC7 | ACSM5 | 0.113826948 | 0.011687847 |
| ZDHHC8 | ACSM5 | -0.01928771 | 0.670180111 |
| ZDHHC9 | ACSM5 | -0.029554559 | 0.513956317 |
| ZDHHC11 | ACSM5 | 0.006997471 | 0.877214009 |
| ZDHHC12 | ACSM5 | -0.048395414 | 0.284993683 |
| ZDHHC13 | ACSM5 | -0.118116652 | 0.0088677 |
| ZDHHC14 | ACSM5 | 0.220691997 | 8.07E-07 |
| ZDHHC15 | ACSM5 | 0.270551734 | 1.15E-09 |
| ZDHHC16 | ACSM5 | -0.008538369 | 0.850464667 |
| ZDHHC17 | ACSM5 | -0.17598145 | 9.00E-05 |
| ZDHHC18 | ACSM5 | -0.033105034 | 0.464693665 |
| ZDHHC19 | ACSM5 | 0.191491025 | 1.98E-05 |
| ZDHHC20 | ACSM5 | 0.081379642 | 0.071892151 |
| ZDHHC21 | ACSM5 | 0.028150381 | 0.534160879 |
| ZDHHC22 | ACSM5 | 0.068584147 | 0.129498377 |
| ZDHHC23 | ACSM5 | -0.175498288 | 9.41E-05 |
| ZDHHC24 | ACSM5 | -0.033993188 | 0.452793215 |
| ABHD10 | ACSM5 | -0.04530424 | 0.316921227 |
| ABHD17A | ACSM5 | 0.124989504 | 0.005595887 |
| ABHD17B | ACSM5 | -0.019122989 | 0.672833144 |
| ABHD17C | ACSM5 | -0.105374275 | 0.019642879 |
| LYPLA1 | ACSM5 | -0.105436151 | 0.019570705 |
| LYPLA2 | ACSM5 | -0.086007884 | 0.057101927 |
| ZDHHC1 | SKA3 | -0.4593036 | 6.09E-27 |
| ZDHHC2 | SKA3 | -0.213146943 | 1.93E-06 |
| ZDHHC3 | SKA3 | -0.230326415 | 2.54E-07 |
| ZDHHC4 | SKA3 | 0.041122043 | 0.363701456 |
| ZDHHC5 | SKA3 | 0.085060074 | 0.059905686 |
| ZDHHC6 | SKA3 | 0.079752137 | 0.077784167 |
| ZDHHC7 | SKA3 | -0.384792445 | 9.69E-19 |
| ZDHHC8 | SKA3 | -0.20398397 | 5.32E-06 |
| ZDHHC9 | SKA3 | -0.274070437 | 6.84E-10 |
| ZDHHC11 | SKA3 | -0.316904427 | 6.82E-13 |
| ZDHHC12 | SKA3 | 0.234615796 | 1.49E-07 |
| ZDHHC13 | SKA3 | 0.101538476 | 0.024593956 |
| ZDHHC14 | SKA3 | 0.124301254 | 0.005865792 |
| ZDHHC15 | SKA3 | -0.322897636 | 2.36E-13 |
| ZDHHC16 | SKA3 | -0.268560033 | 1.53E-09 |
| ZDHHC17 | SKA3 | -0.055222044 | 0.222388589 |
| ZDHHC18 | SKA3 | 0.197052841 | 1.11E-05 |
| ZDHHC19 | SKA3 | 0.041892218 | 0.354777827 |
| ZDHHC20 | SKA3 | 0.188677254 | 2.63E-05 |
| ZDHHC21 | SKA3 | -0.217194187 | 1.21E-06 |
| ZDHHC22 | SKA3 | 0.070871386 | 0.117168732 |
| ZDHHC23 | SKA3 | 0.219044388 | 9.78E-07 |
| ZDHHC24 | SKA3 | 0.010543591 | 0.815914604 |
| ABHD10 | SKA3 | 0.268911266 | 1.46E-09 |
| ABHD17A | SKA3 | -0.153867264 | 0.000631593 |
| ABHD17B | SKA3 | 0.073922513 | 0.102174449 |
| ABHD17C | SKA3 | -0.149319396 | 0.000914828 |
| LYPLA1 | SKA3 | 0.272795216 | 8.25E-10 |
| LYPLA2 | SKA3 | -0.127234002 | 0.004791547 |
