# Supplementary material for: Integrated analysis identifies a palmitoylation-associated prognostic model (ACSM5/SKA3) for lung adenocarcinoma across multiple cohorts
Source: PeerJ. 2026 Apr 29;14:e21160. doi: 10.7717/peerj.21160 (PMC13135332; doi:10.7717/peerj.21160)
Supplement: Supplemental Information 5 [file peerj-14-21160-s005.zip › peerj-125255-Supplementary_Figure_S4.pdf]

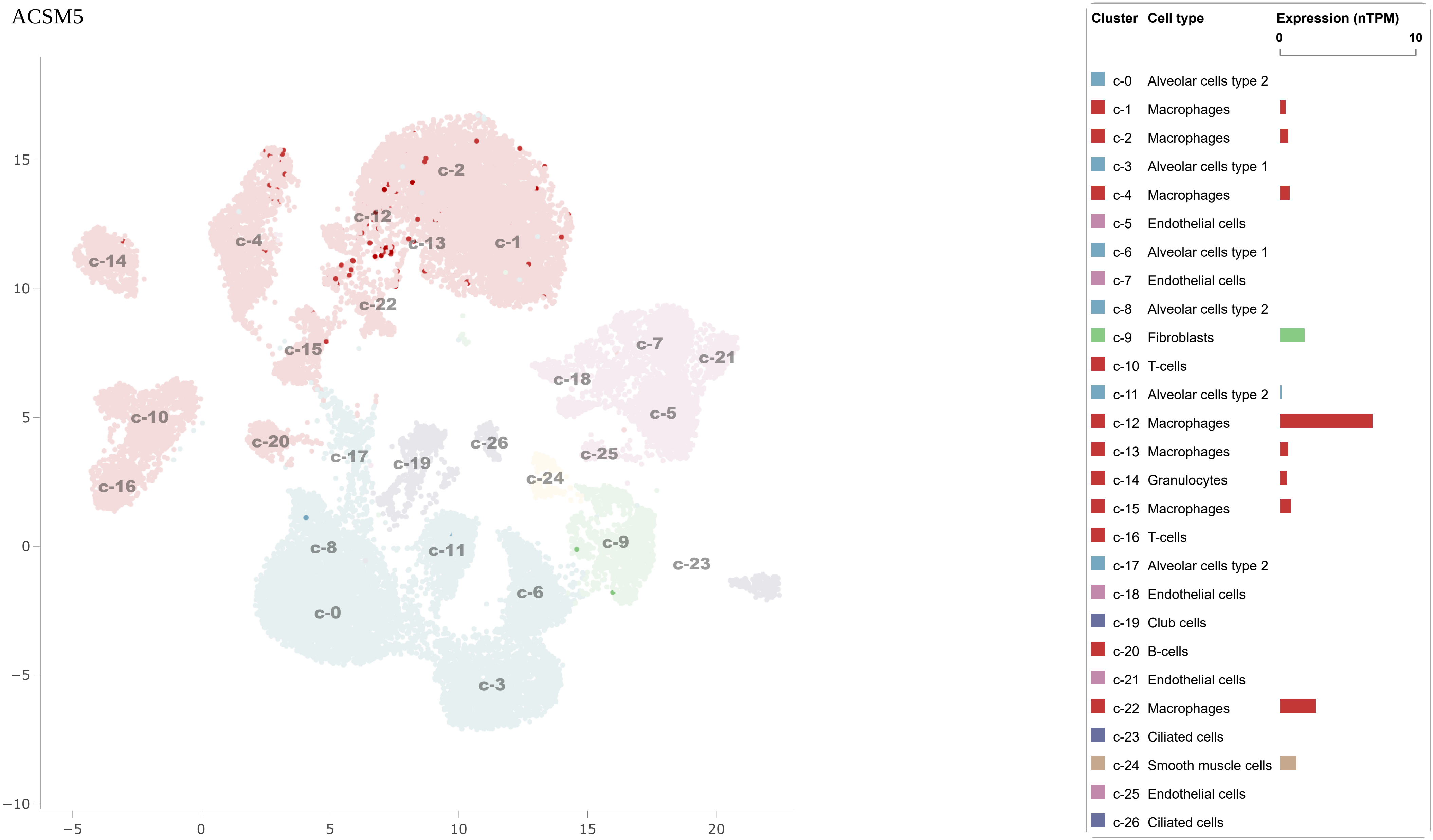
ACSM5
Cluster
Cell type
Expression (nTPM)
0
10
c-0 Alveolar cells type 2
c-1 Macrophages
c-2 Macrophages
c-3 Alveolar cells type 1
c-4 Macrophages
c-5 Endothelial cells
c-6 Alveolar cells type 1
c-7 Endothelial cells
c-8 Alveolar cells type 2
c-9 Fibroblasts
c-10 T-cells
c-11 Alveolar cells type 2
c-12 Macrophages
c-13 Macrophages
c-14 Granulocytes
c-15 Macrophages
c-16 T-cells
c-17 Alveolar cells type 2
c-18 Endothelial cells
c-19 Club cells
c-20 B-cells
c-21 Endothelial cells
c-22 Macrophages
c-23 Ciliated cells
c-24 Smooth muscle cells
c-25 Endothelial cells
c-26 Ciliated cells

ACSM5

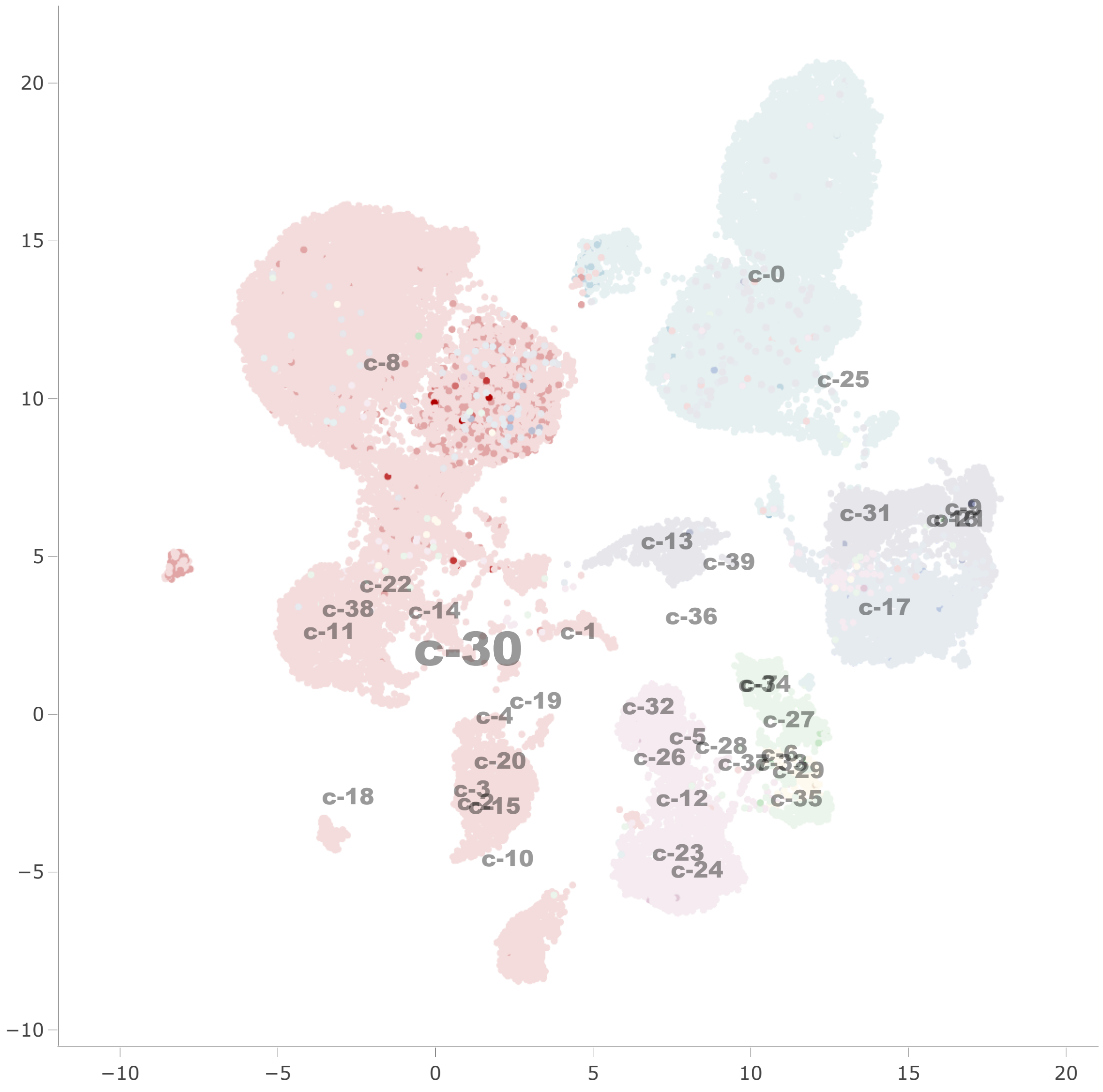

Cluster	Cell type	Expression (nTPM) 0–10
c-0	Type ii pneumocyte	
c-1	Neutrophil	
c-2	Cd4-positive alpha-beta t cell	
c-3	Cd8-positive alpha-beta t cell	
c-4	Nk cell	
c-5	Bronchial vessel endothelial cell	
c-6	Smooth muscle cell	
c-7	Adventitial cell	
c-8	Macrophage	
c-9	Respiratory mucous cell	
c-10	Basophil	
c-11	Classical monocyte	
c-12	Endothelial cell of artery	
c-13	Lung ciliated cell	
c-14	Dendritic cell	
c-15	Cd4-positive, alpha-beta t cell	
c-16	Respiratory goblet cell	
c-17	Basal cell	
c-18	Plasma cell	
c-19	B cell	
c-20	Cd8-positive, alpha-beta t cell	
c-21	Serous cell of epithelium of bronchus	
c-22	Non-classical monocyte	
c-23	Capillary endothelial cell	
c-24	Capillary aerocyte	
c-25	Type i pneumocyte	
c-26	Vein endothelial cell	
c-27	Alveolar fibroblast	
c-28	Endothelial cell of lymphatic vessel	
c-29	Bronchial smooth muscle cell	
c-30	Plasmacytoid dendritic cell	
c-31	Club cell	
c-32	Lung microvascular endothelial cell	
c-33	Vascular associated smooth muscle cell	
c-34	Myofibroblast cell	
c-35	Pericyte cell	
c-36	Mesothelial cell	
c-37	Fibroblast	
c-38	Intermediate monocyte	
c-39	Pulmonary ionocyte	

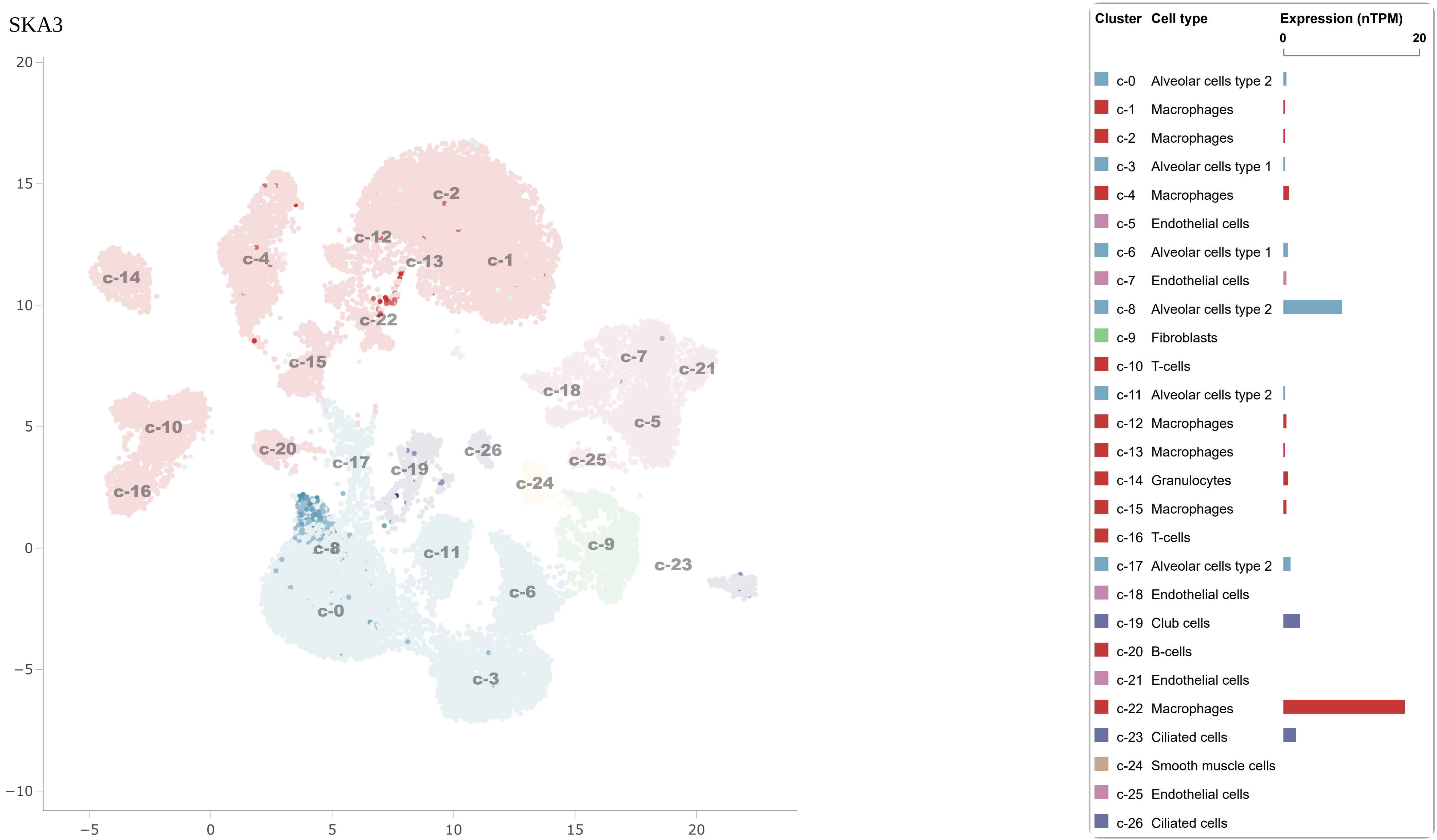

SKA3
c-0
c-1
c-2
c-3
c-4
c-5
c-6
c-7
c-8
c-9
c-10
c-11
c-12
c-13
c-14
c-15
c-16
c-17
c-18
c-19
c-20
c-21
c-22
c-23
c-24
c-25
c-26
20
15
10
5
0
−5
−10
−5
0
5
10
15
20
Cluster
Cell type
Expression (nTPM)
0
20
c-0 Alveolar cells type 2
c-1 Macrophages
c-2 Macrophages
c-3 Alveolar cells type 1
c-4 Macrophages
c-5 Endothelial cells
c-6 Alveolar cells type 1
c-7 Endothelial cells
c-8 Alveolar cells type 2
c-9 Fibroblasts
c-10 T-cells
c-11 Alveolar cells type 2
c-12 Macrophages
c-13 Macrophages
c-14 Granulocytes
c-15 Macrophages
c-16 T-cells
c-17 Alveolar cells type 2
c-18 Endothelial cells
c-19 Club cells
c-20 B-cells
c-21 Endothelial cells
c-22 Macrophages
c-23 Ciliated cells
c-24 Smooth muscle cells
c-25 Endothelial cells
c-26 Ciliated cells

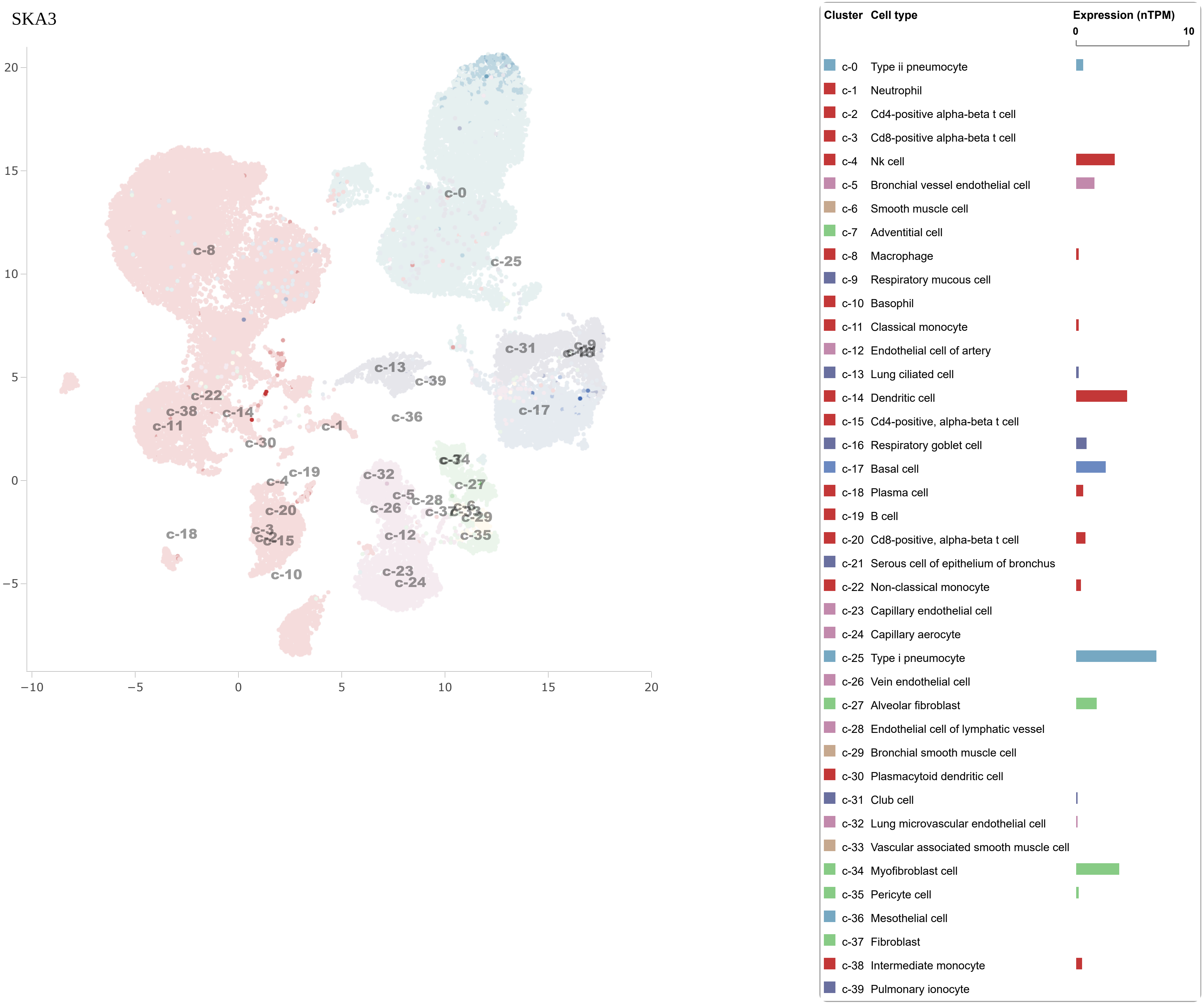

SKA3
Cluster Cell type Expression (nTPM)
0 10
c-0 Type ii pneumocyte
c-1 Neutrophil
c-2 Cd4-positive alpha-beta t cell
c-3 Cd8-positive alpha-beta t cell
c-4 Nk cell
c-5 Bronchial vessel endothelial cell
c-6 Smooth muscle cell
c-7 Adventitial cell
c-8 Macrophage
c-9 Respiratory mucous cell
c-10 Basophil
c-11 Classical monocyte
c-12 Endothelial cell of artery
c-13 Lung ciliated cell
c-14 Dendritic cell
c-15 Cd4-positive, alpha-beta t cell
c-16 Respiratory goblet cell
c-17 Basal cell
c-18 Plasma cell
c-19 B cell
c-20 Cd8-positive, alpha-beta t cell
c-21 Serous cell of epithelium of bronchus
c-22 Non-classical monocyte
c-23 Capillary endothelial cell
c-24 Capillary aerocyte
c-25 Type i pneumocyte
c-26 Vein endothelial cell
c-27 Alveolar fibroblast
c-28 Endothelial cell of lymphatic vessel
c-29 Bronchial smooth muscle cell
c-30 Plasmacytoid dendritic cell
c-31 Club cell
c-32 Lung microvascular endothelial cell
c-33 Vascular associated smooth muscle cell
c-34 Myofibroblast cell
c-35 Pericyte cell
c-36 Mesothelial cell
c-37 Fibroblast
c-38 Intermediate monocyte
c-39 Pulmonary ionocyte
−10 −5 0 5 10 15 20
20 15 10 5 0 −5
