# Supplementary material for: Integrated analysis identifies a palmitoylation-associated prognostic model (ACSM5/SKA3) for lung adenocarcinoma across multiple cohorts
Source: PeerJ. 2026 Apr 29;14:e21160. doi: 10.7717/peerj.21160 (PMC13135332; doi:10.7717/peerj.21160)
Supplement: Supplemental Information 5 [file peerj-14-21160-s005.zip › peerj-125255-Supplementary_Figure_S2.pdf]

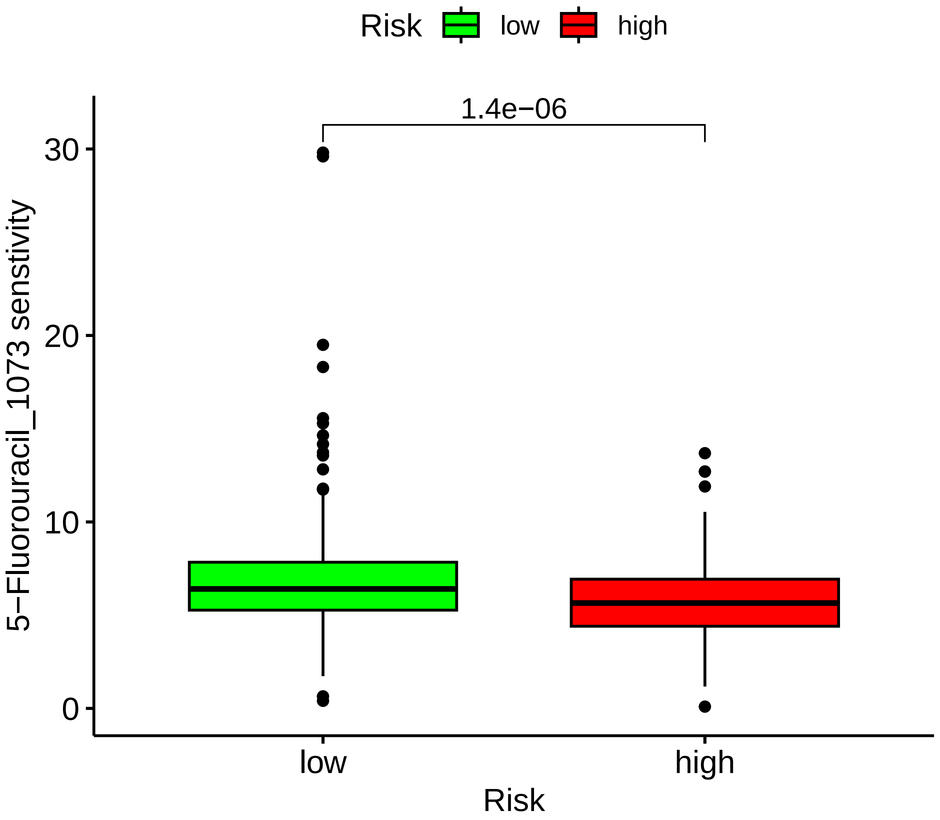

Risk
low
high
1.4e−06
30
20
10
0
5−Fluorouracil_1073 senstivity
low
high
Risk

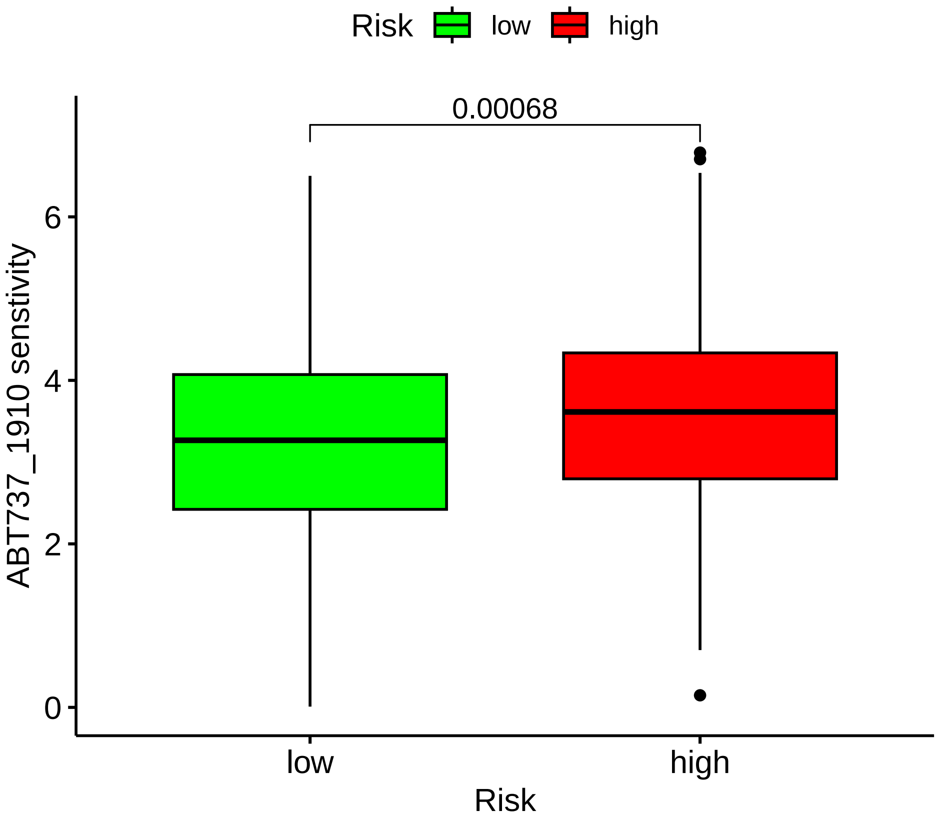

Risk
low
high
0.00068
ABT737_1910 senstivity
0
2
4
6
low
high
Risk

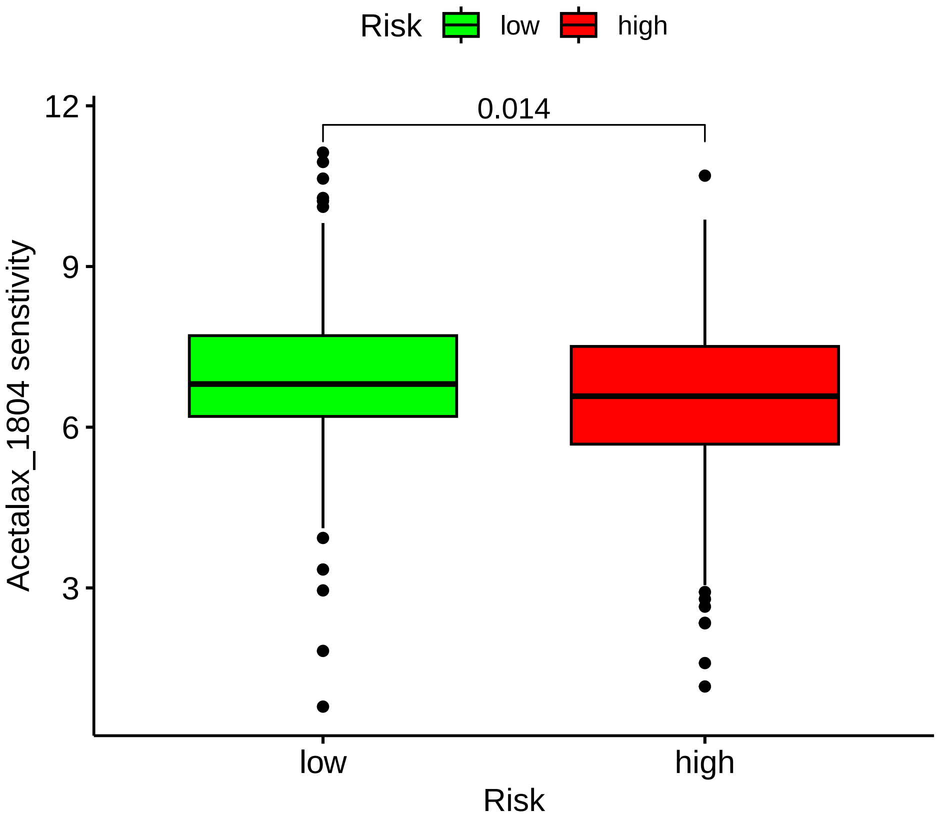
Risk
low
high
0.014
12
9
6
3
Acetalax_1804 senstivity
low
high
Risk

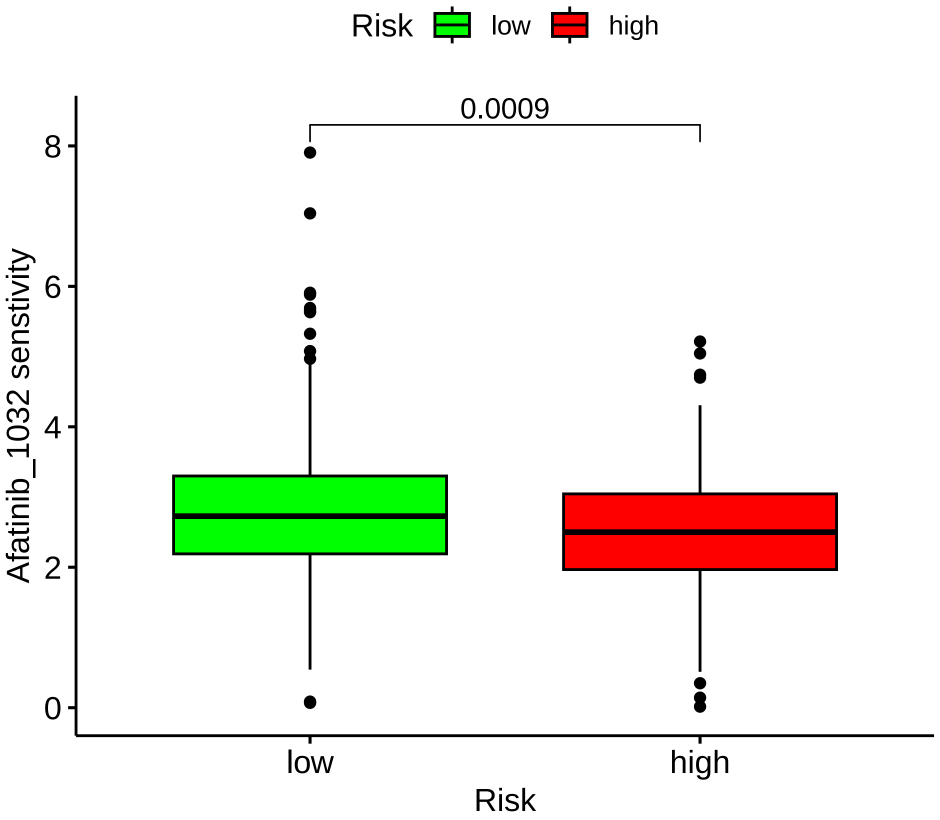

Risk
low
high
0.0009
Afatinib_1032 senstivity
8
6
4
2
0
low
high
Risk

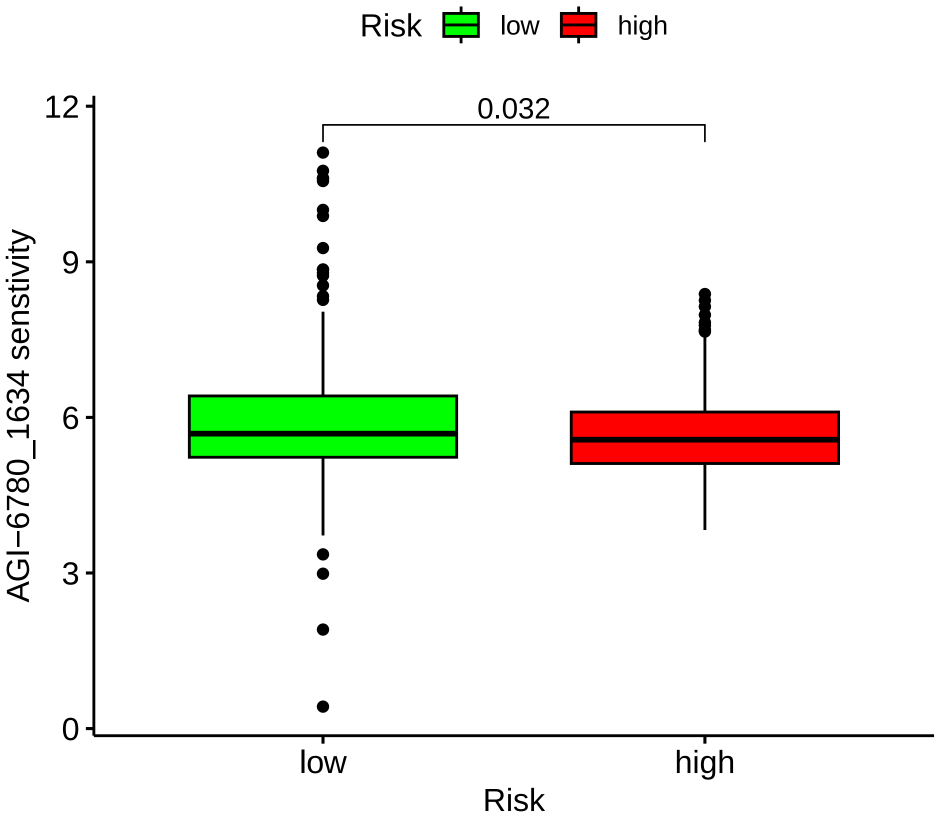

Risk
low
high
0.032
AGI−6780_1634 senstivity
12
9
6
3
0
low
high
Risk

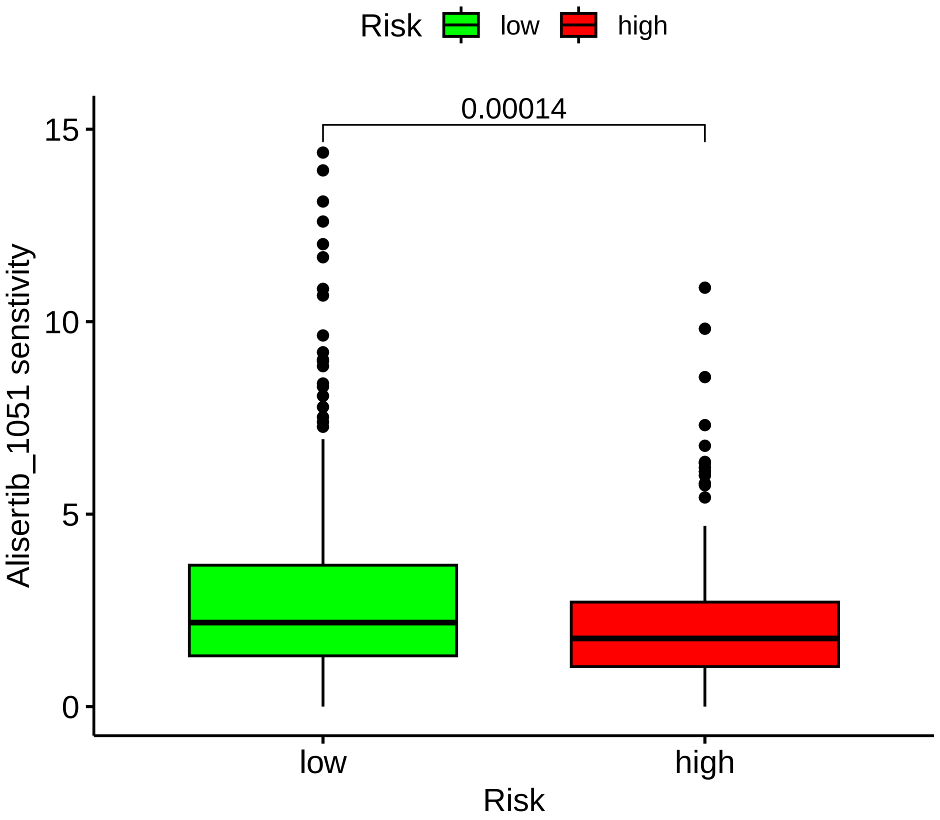

Risk
low
high
0.00014
Alisertib_1051 senstivity
15
10
5
0
low
high
Risk

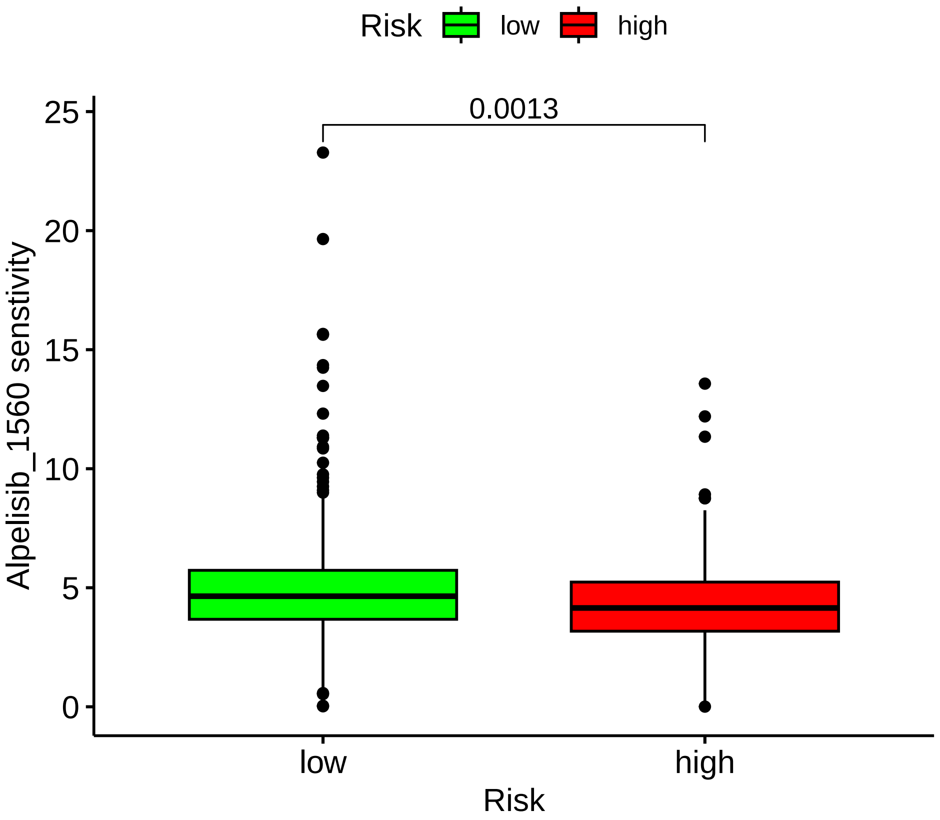

Risk
low
high
0.0013
Alpelisib_1560 senstivity
25
20
15
10
5
0
low
high
Risk

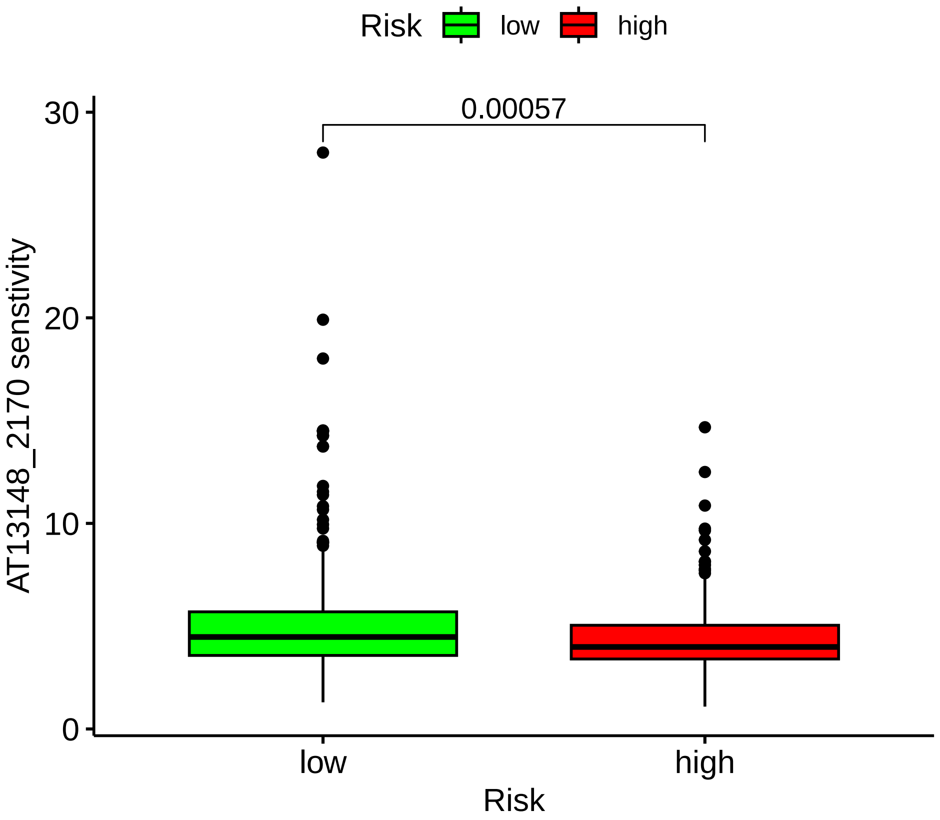
Risk
low
high
0.00057
30
20
10
0
AT13148_2170 senstivity
low
high
Risk

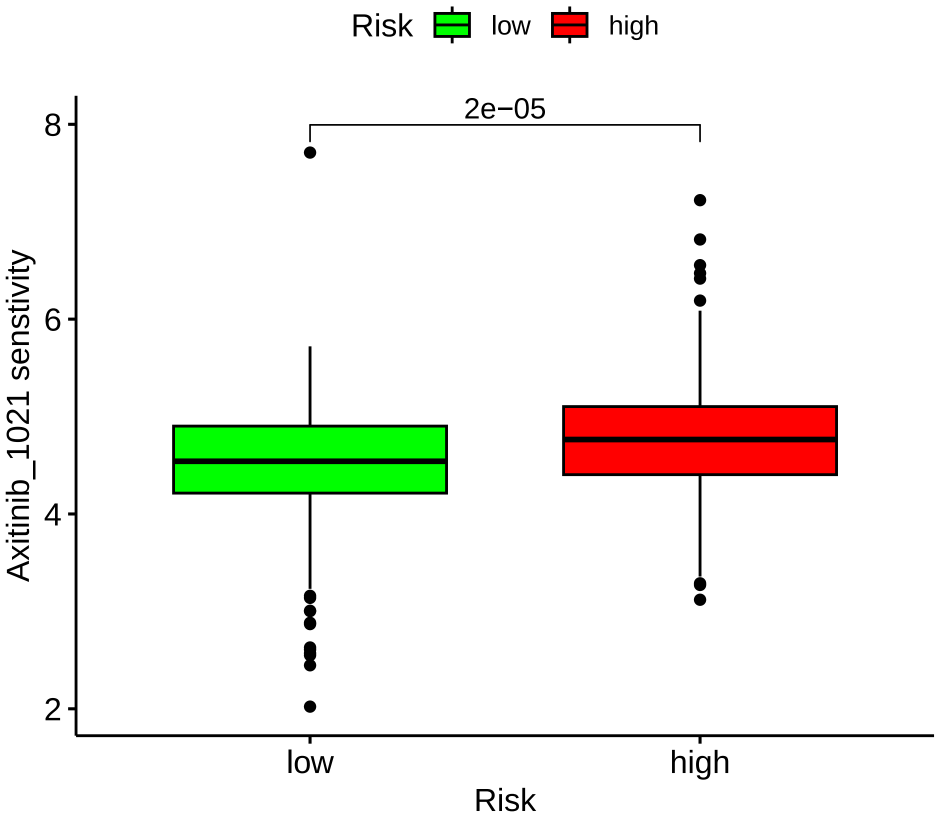

Risk
low
high
2e−05
Axitinib_1021 senstivity
8
6
4
2
low
high
Risk

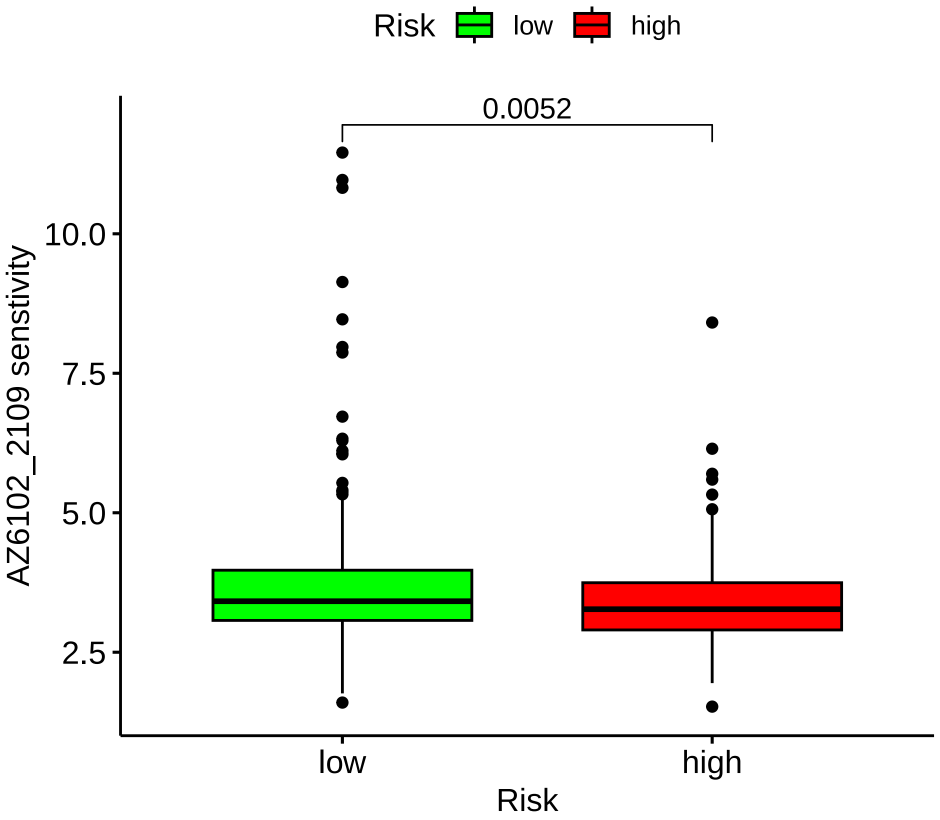

Risk
low
high
0.0052
AZ6102_2109 senstivity
10.0
7.5
5.0
2.5
low
high
Risk

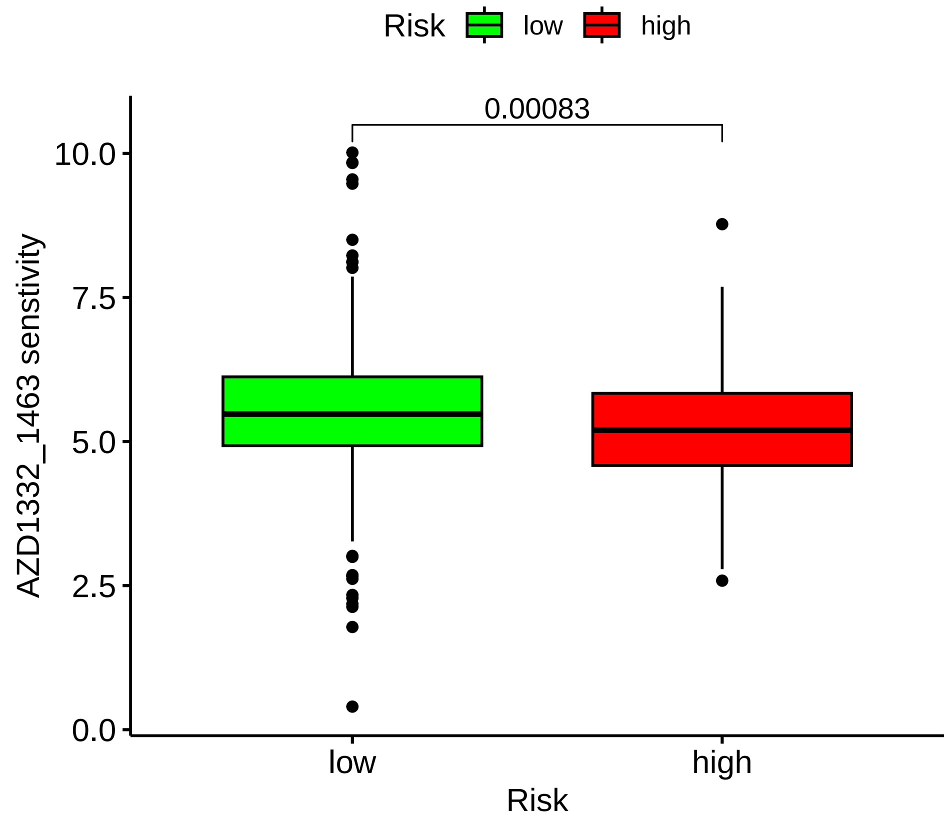

Risk
low
high
0.00083
10.0
7.5
5.0
2.5
0.0
AZD1332_1463 senstivity
low
high
Risk

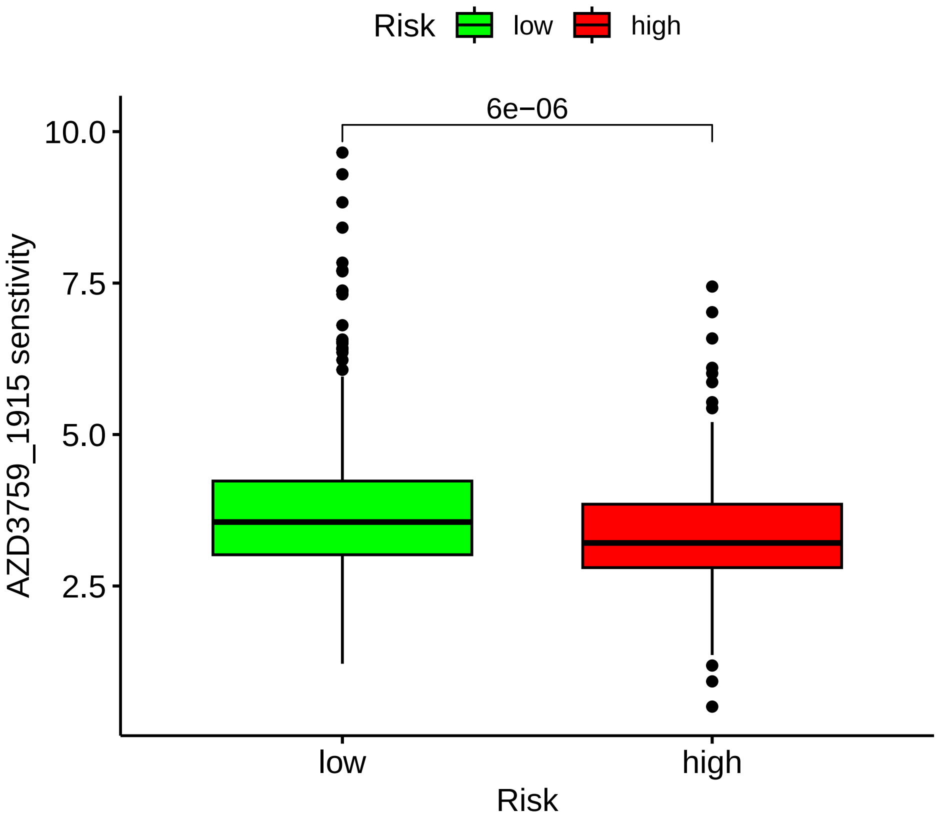

Risk
low
high
6e−06
10.0
7.5
5.0
2.5
AZD3759_1915 senstivity
low
high
Risk

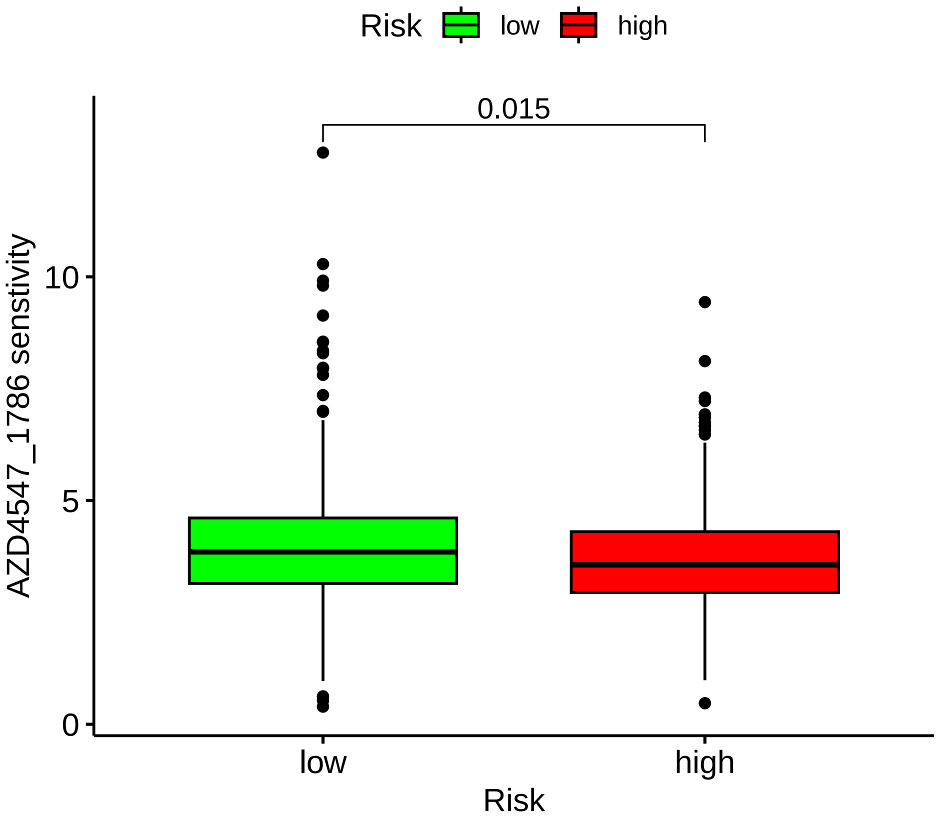
Risk
low
high
0.015
AZD4547_1786 senstivity
10
5
0
low
high
Risk

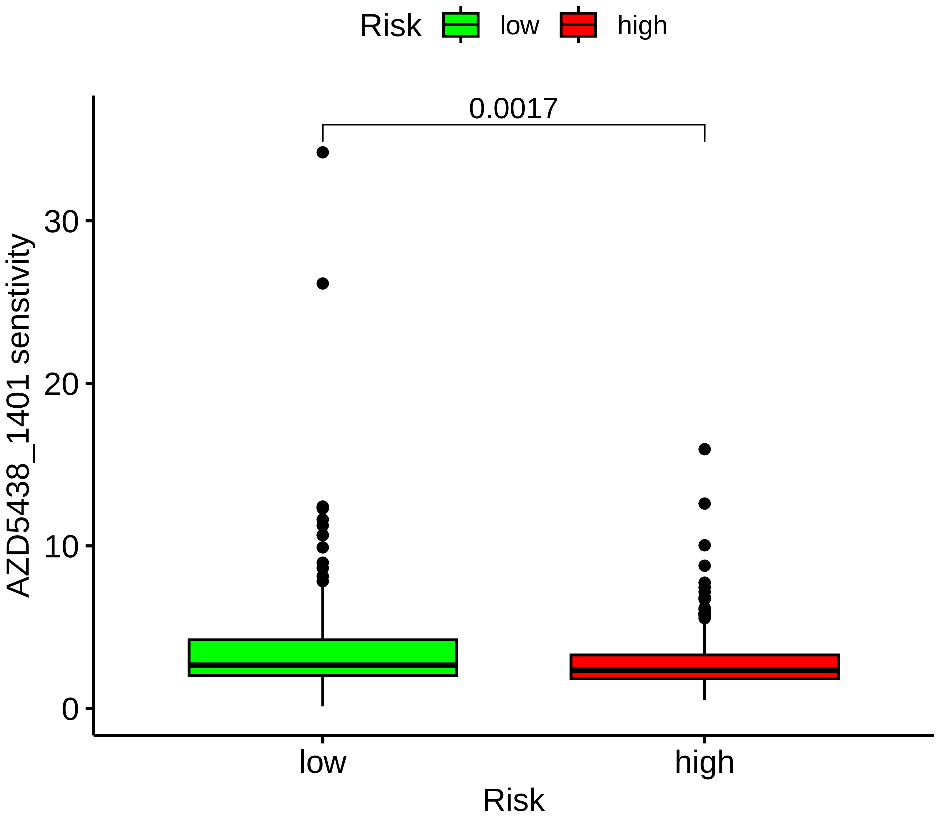
Risk
low
high
0.0017
AZD5438_1401 senstivity
30
20
10
0
low
high
Risk

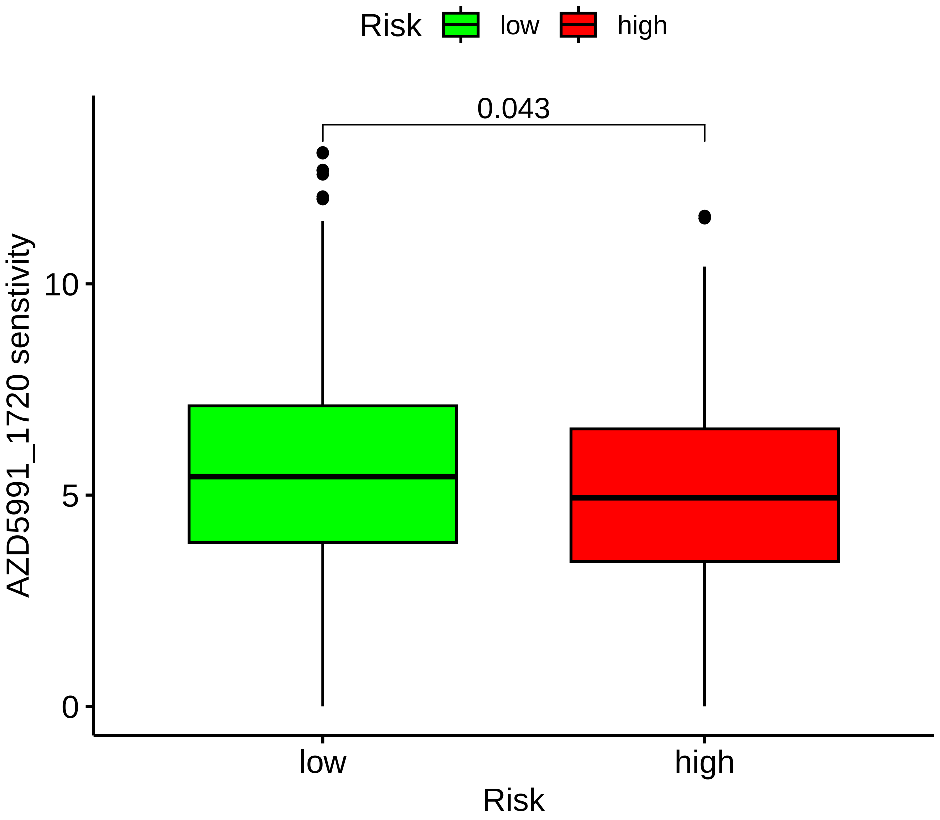

Risk
low
high
0.043
AZD5991_1720 senstivity
10
5
0
low
high
Risk

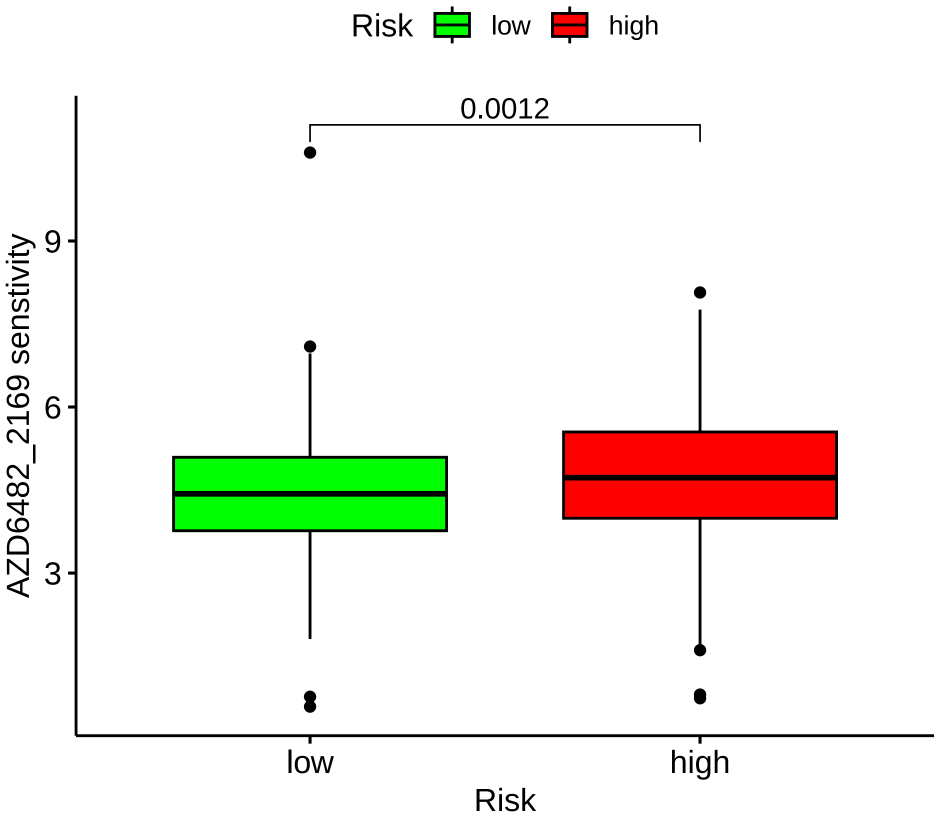

Risk
low
high
0.0012
AZD6482_2169 senstivity
9
6
3
low
high
Risk

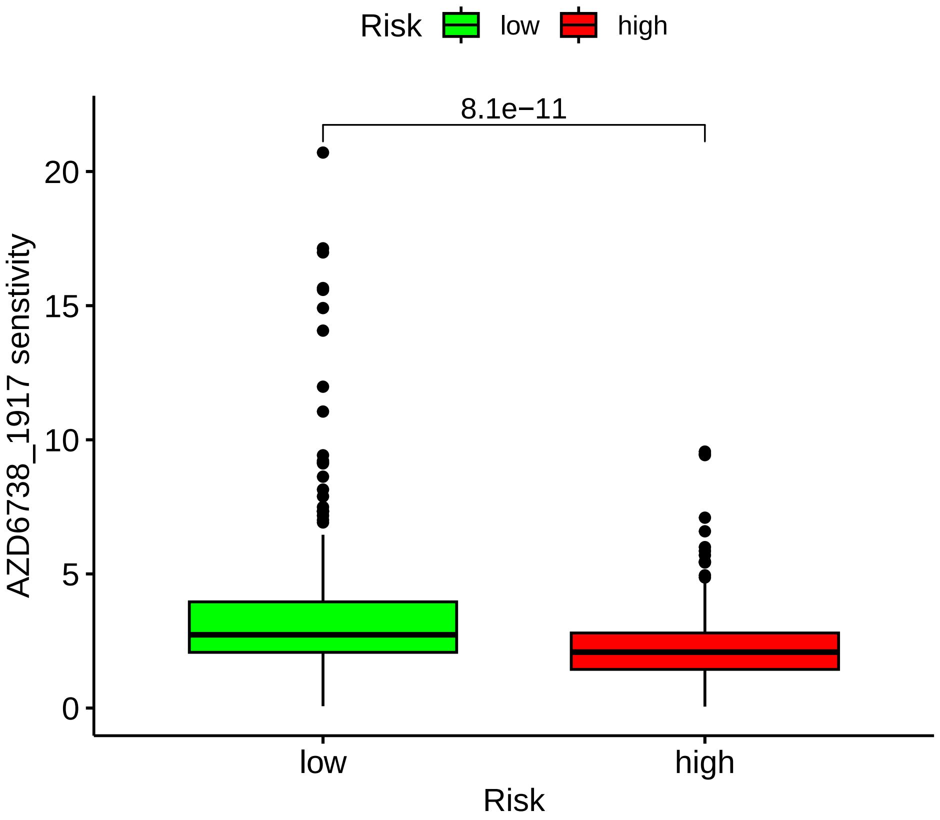
Risk
low
high
8.1e−11
AZD6738_1917 senstivity
0
5
10
15
20
low
high
Risk

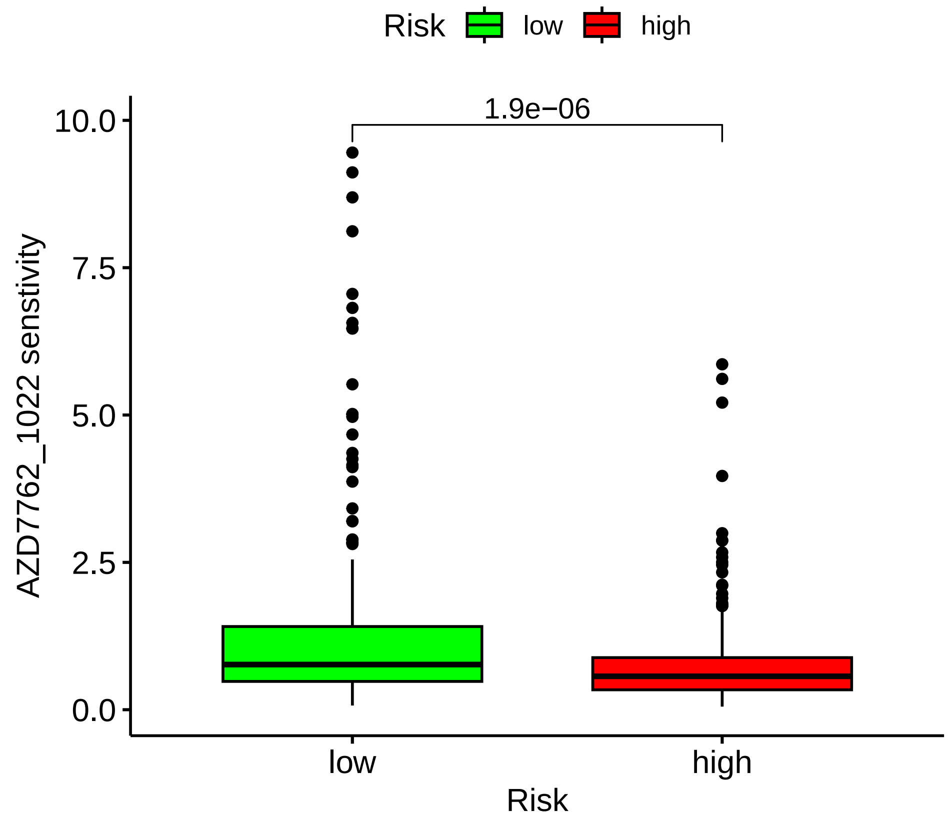
Risk
low
high
1.9e−06
10.0
7.5
5.0
2.5
0.0
AZD7762_1022 senstivity
low
high
Risk

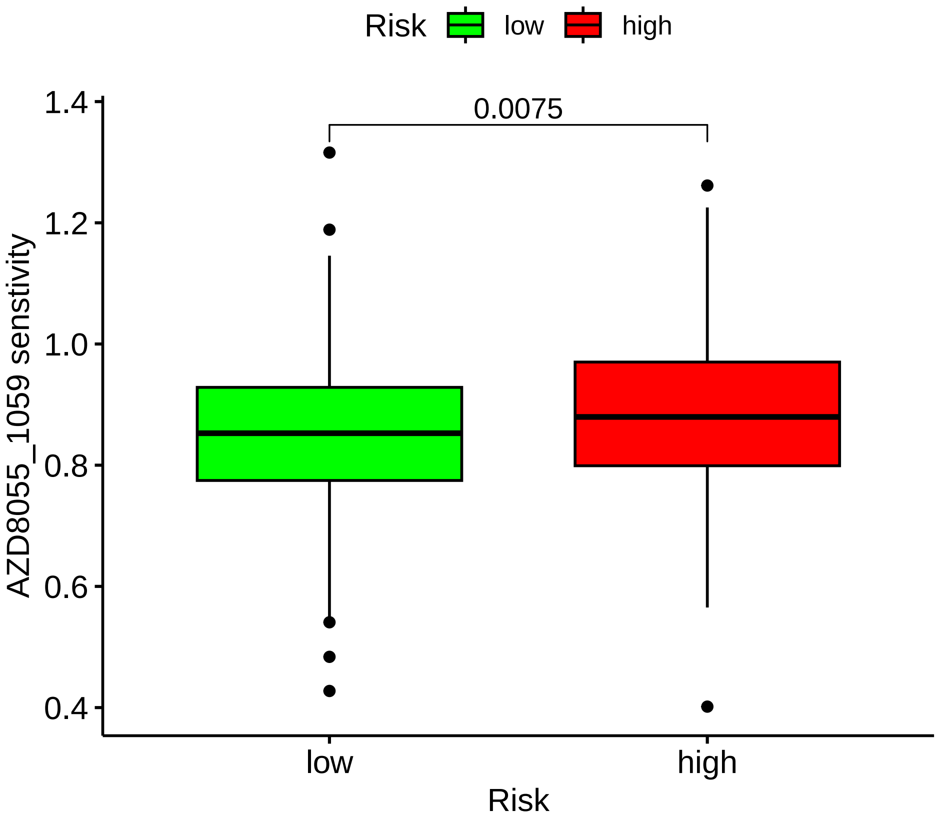
Risk
low
high
0.0075
AZD8055_1059 senstivity
1.4
1.2
1.0
0.8
0.6
0.4
low
high
Risk

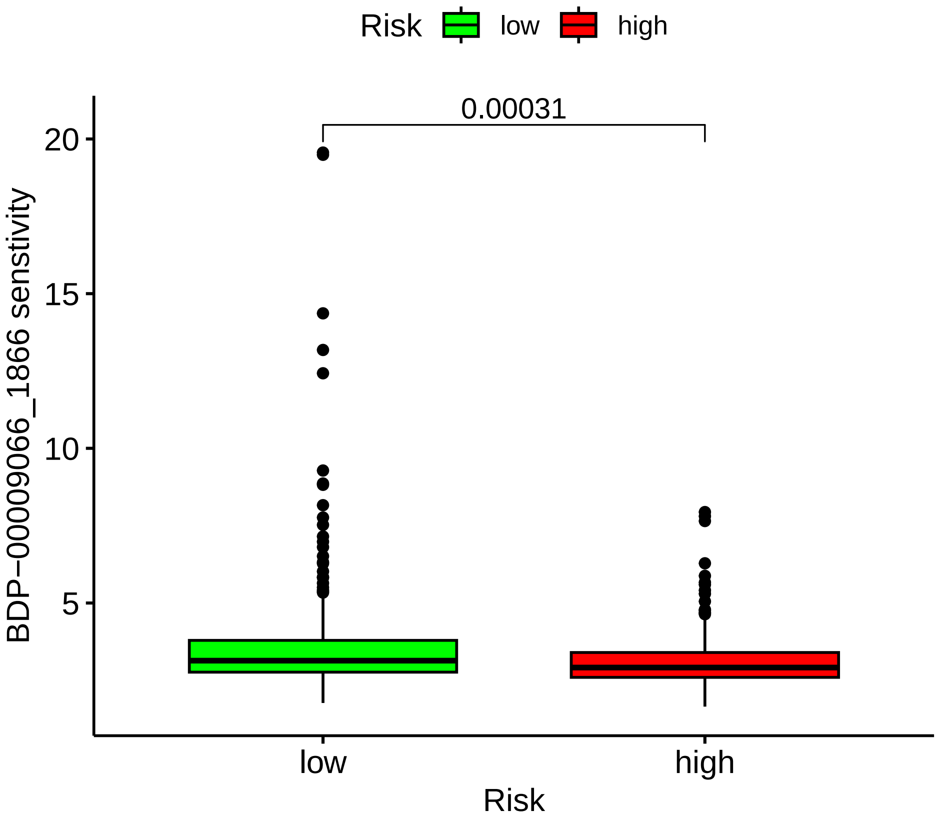

Risk
low
high
0.00031
20
15
10
5
BDP−00009066_1866 senstivity
low
high
Risk

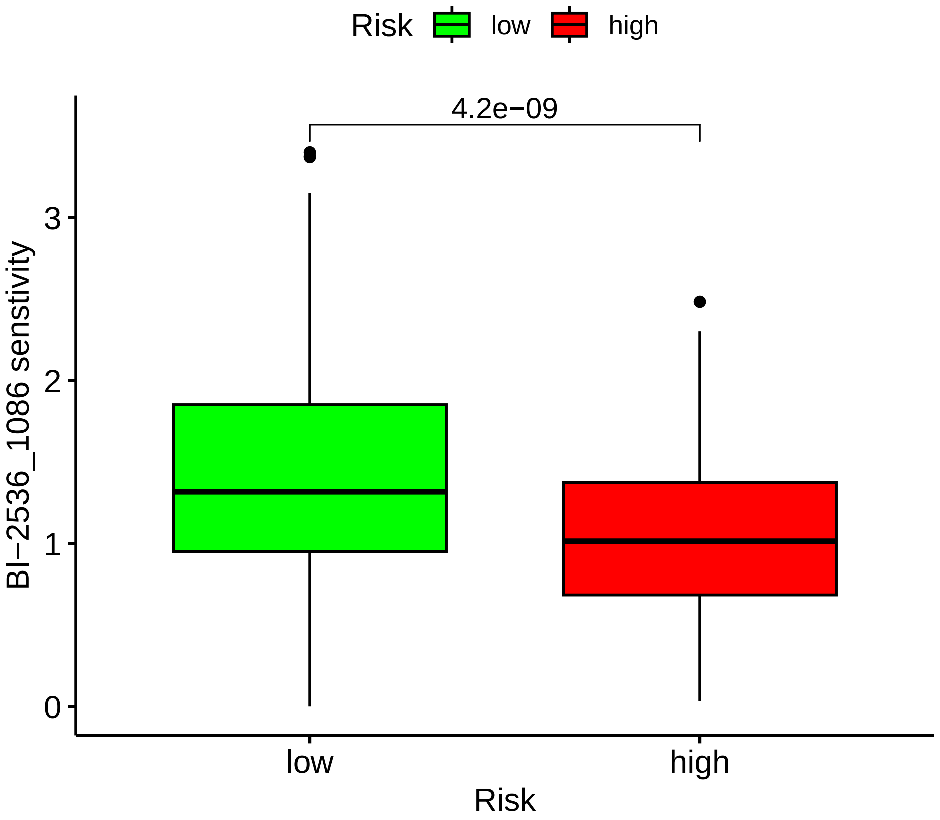
Risk
low
high
4.2e−09
BI−2536_1086 senstivity
0
1
2
3
low
high
Risk

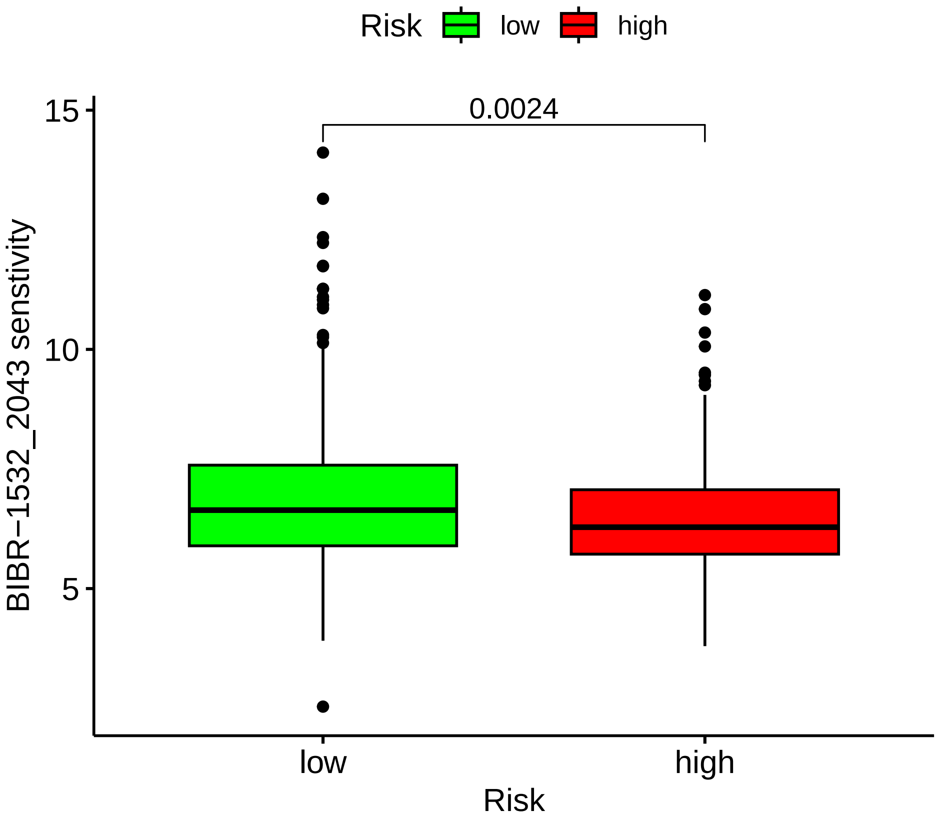

Risk
low
high
0.0024
15
10
5
BIBR−1532_2043 senstivity
low
high
Risk

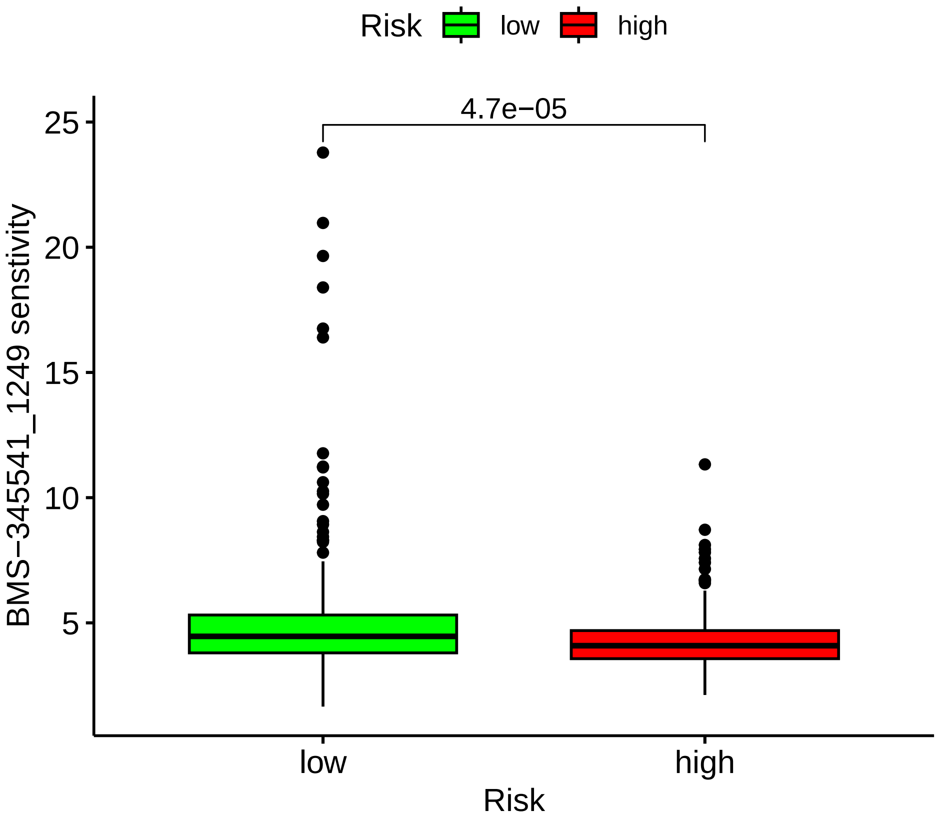

Risk
low
high
4.7e−05
BMS−345541_1249 senstivity
25
20
15
10
5
low
high
Risk

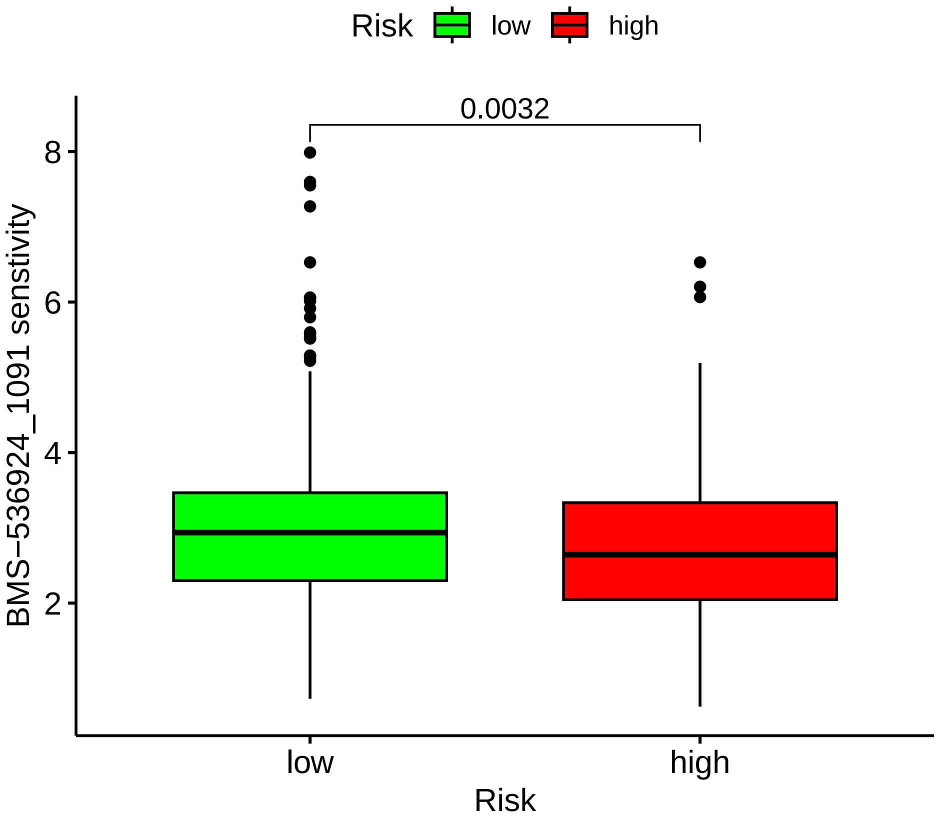

Risk
low
high
0.0032
BMS−536924_1091 senstivity
8
6
4
2
low
high
Risk

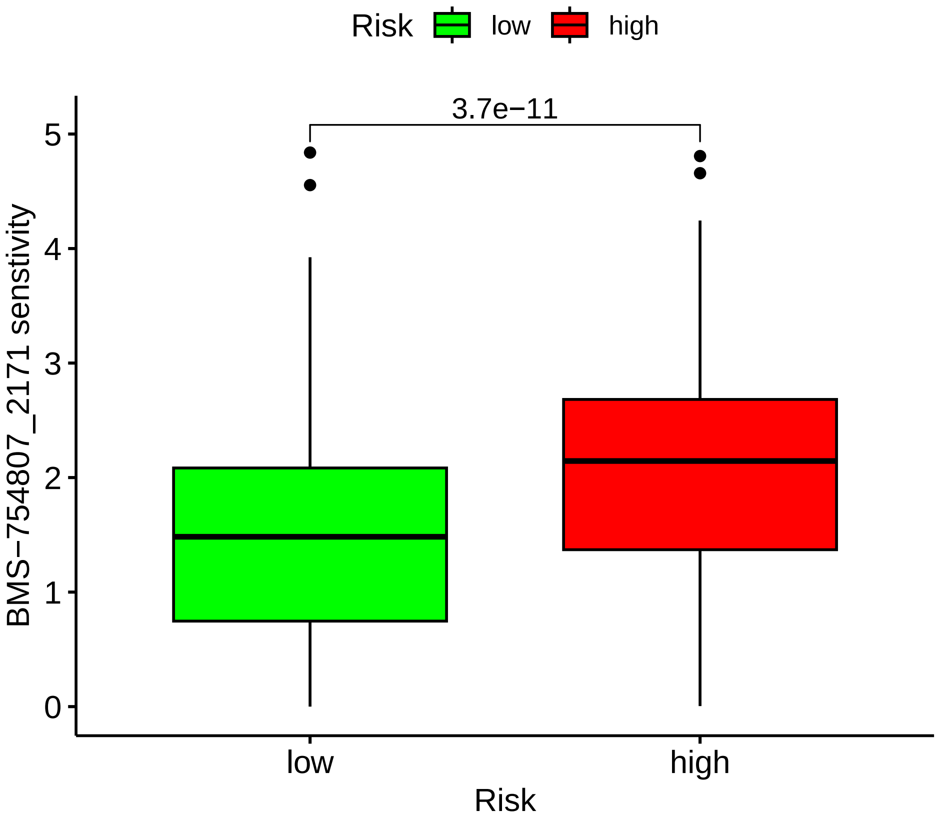
Risk
low
high
3.7e−11
BMS−754807_2171 senstivity
5
4
3
2
1
0
low
high
Risk

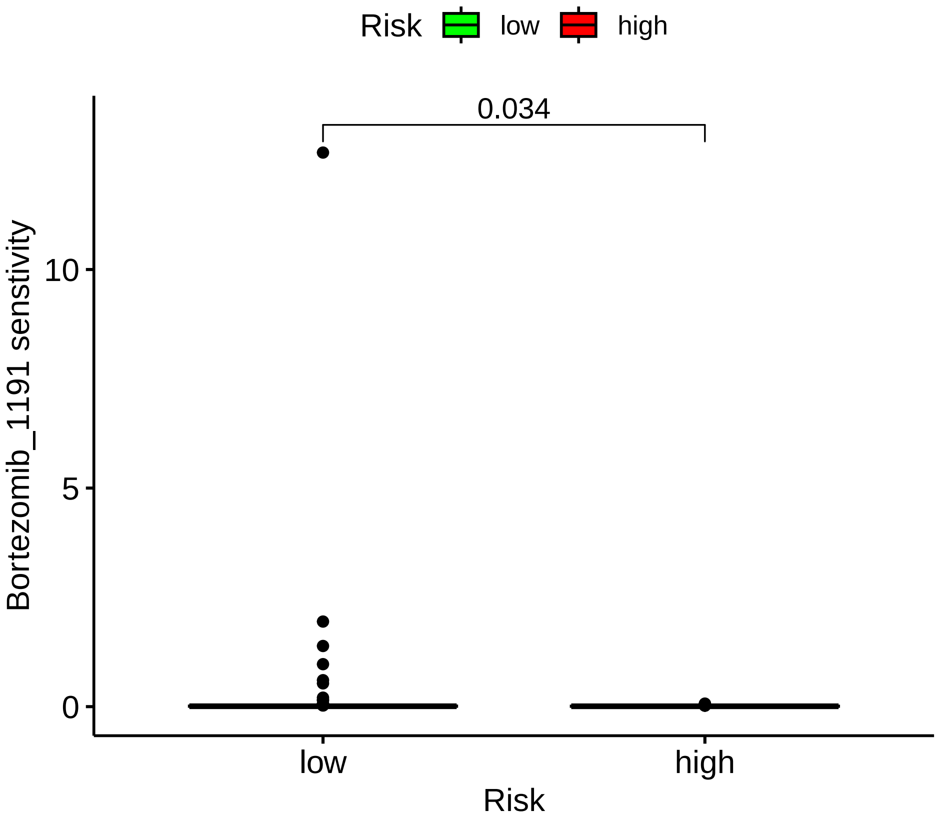
Risk
low
high
0.034
Bortezomib_1191 senstivity
10
5
0
low
high
Risk

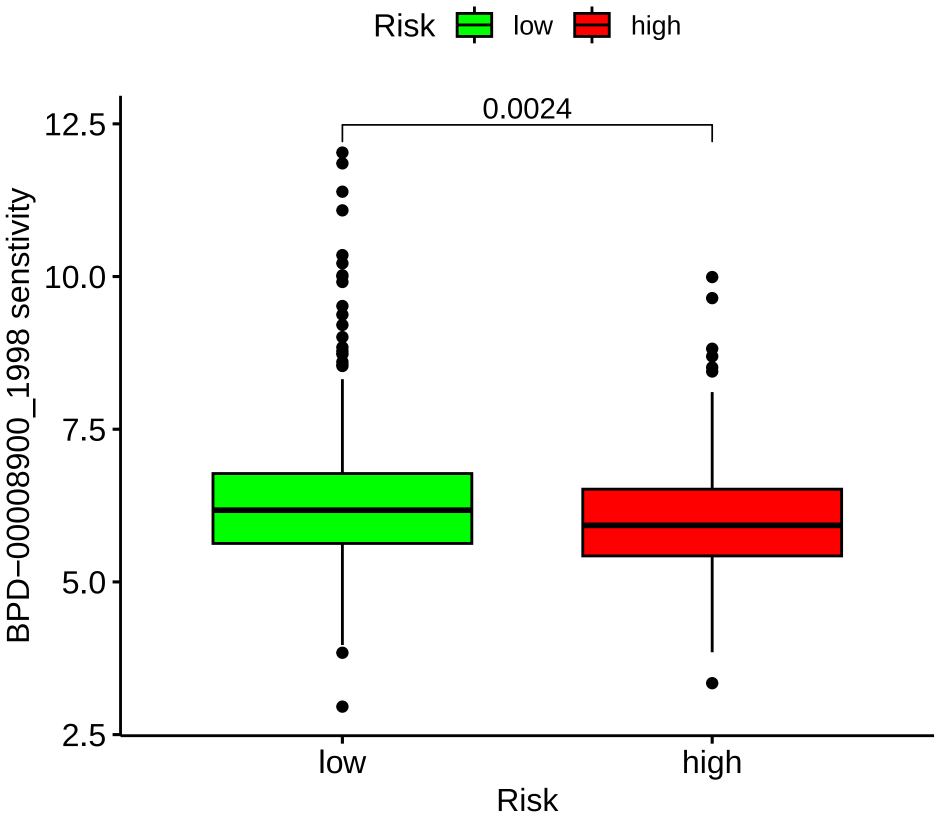

Risk
low
high
0.0024
12.5
10.0
7.5
5.0
2.5
BPD−00008900_1998 senstivity
low
high
Risk

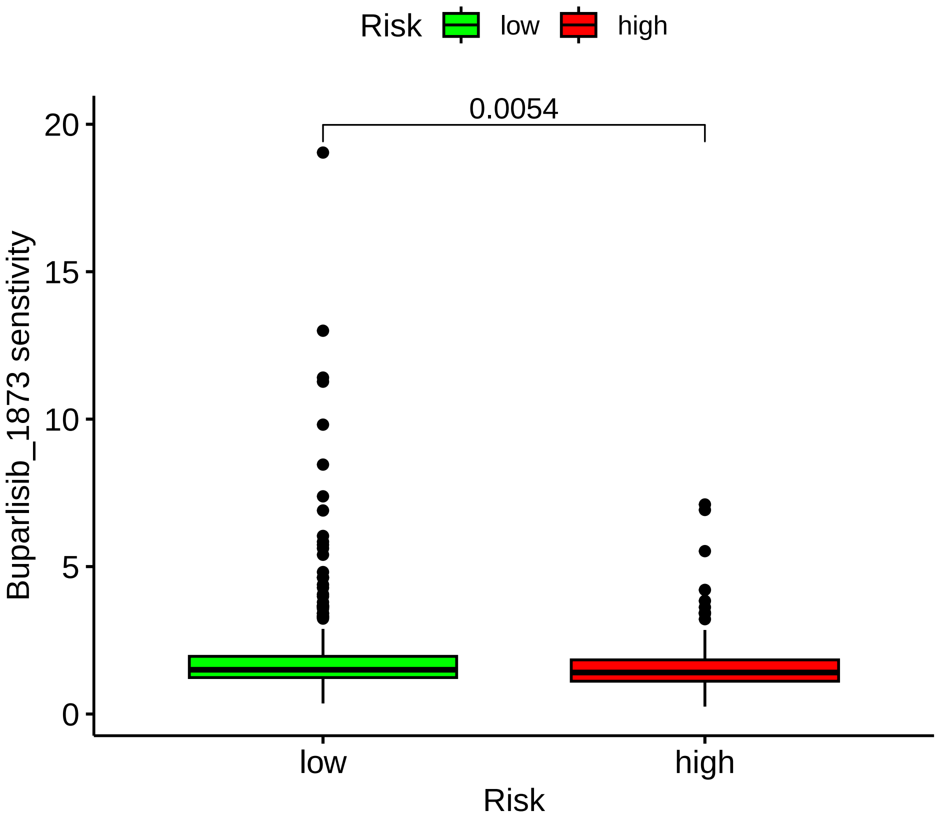
Risk
low
high
0.0054
Buparlisib_1873 senstivity
20
15
10
5
0
low
high
Risk

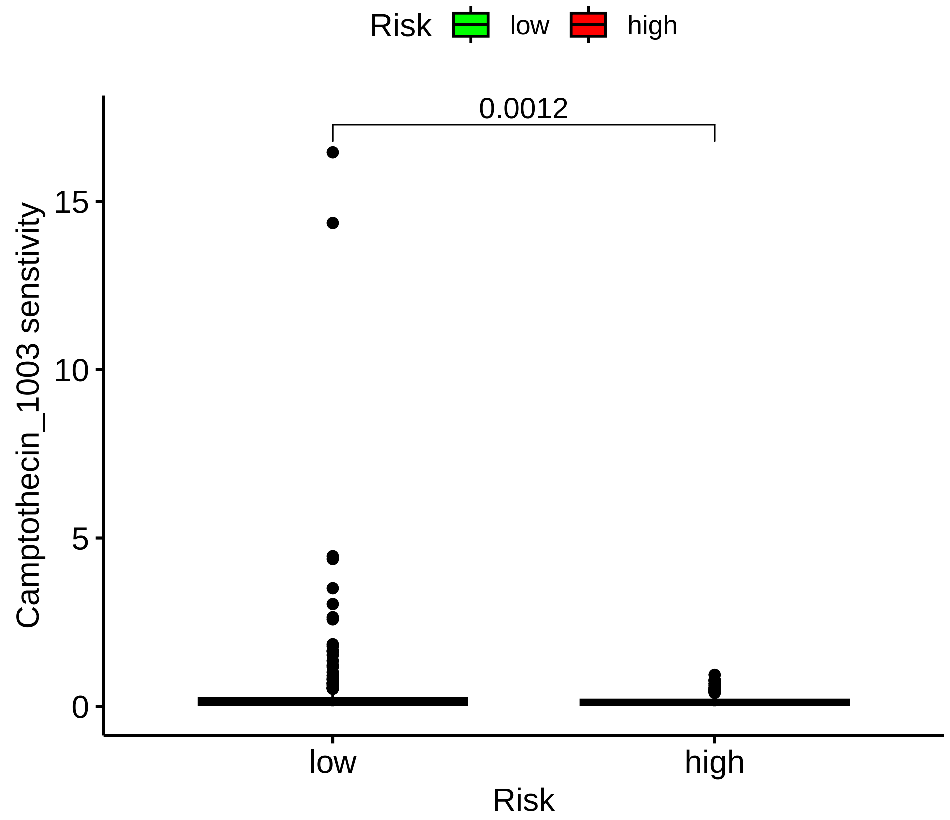

Risk
low
high
0.0012
Camptothecin_1003 senstivity
15
10
5
0
low
high
Risk

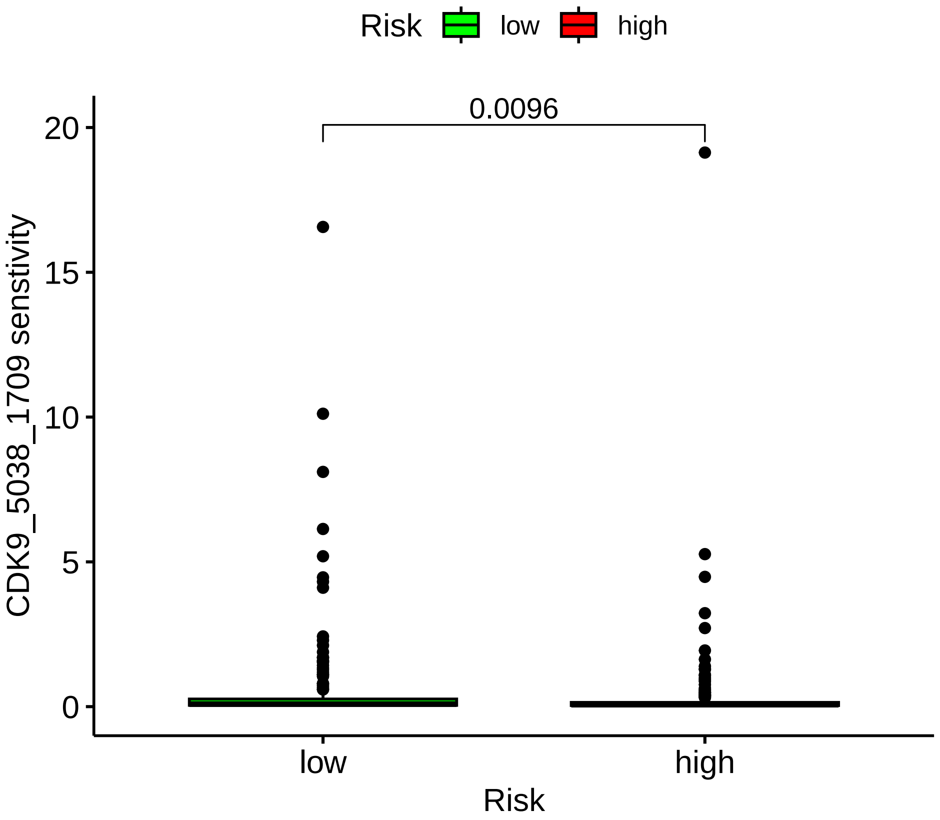

Risk
low
high
0.0096
20
15
10
5
0
CDK9_5038_1709 senstivity
low
high
Risk

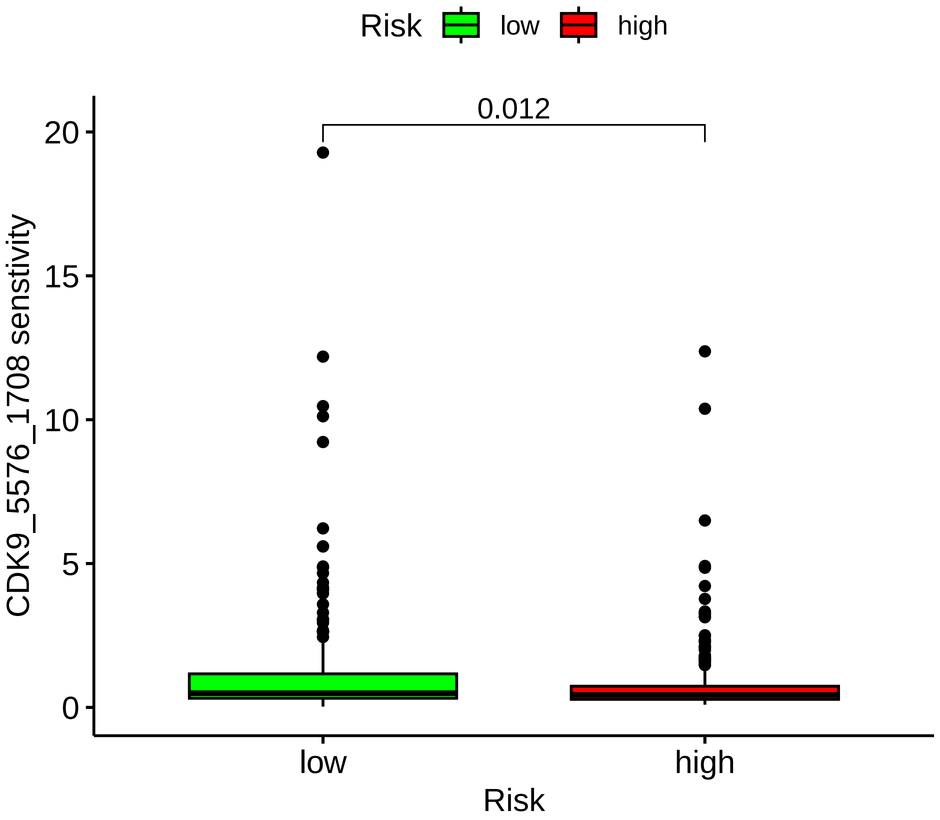

Risk
low
high
0.012
CDK9_5576_1708 senstivity
20
15
10
5
0
low
high
Risk

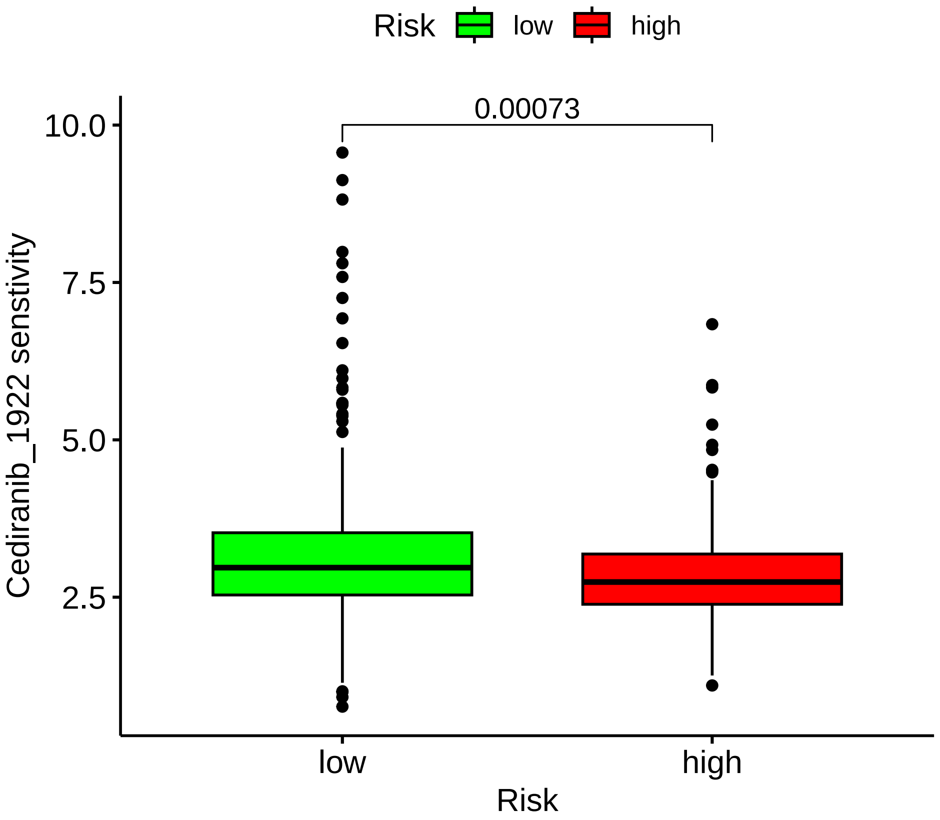
Risk
low
high
0.00073
10.0
7.5
5.0
2.5
Cediranib_1922 senstivity
low
high
Risk

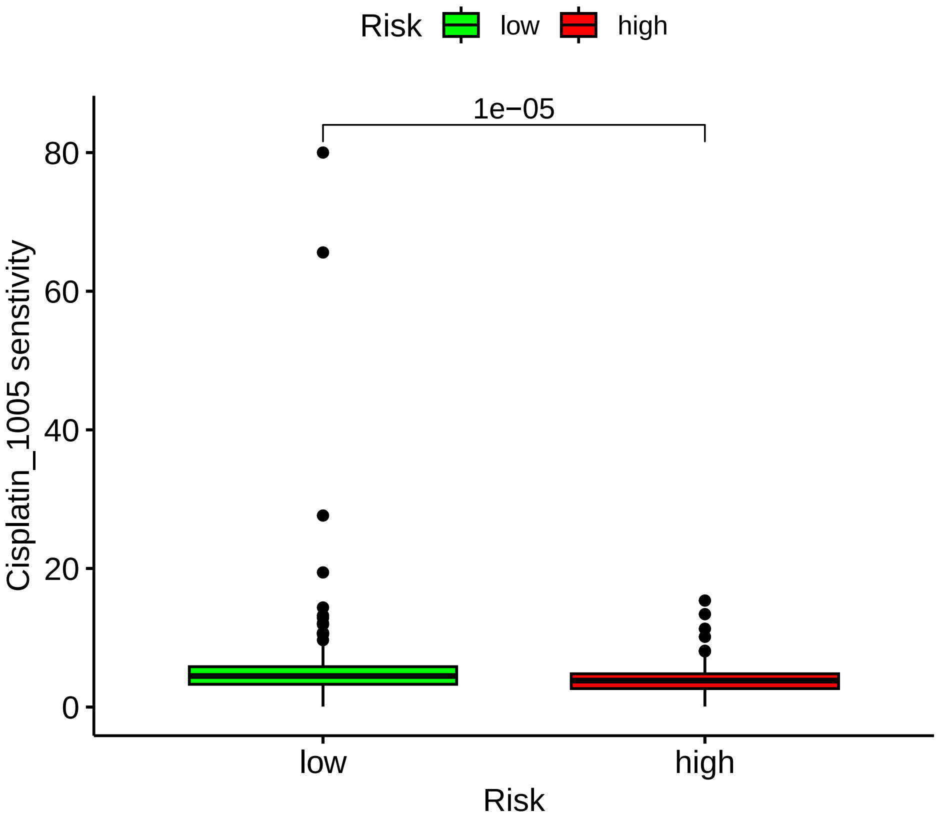
Risk
low
high
1e−05
80
60
40
20
0
Cisplatin_1005 senstivity
low
high
Risk

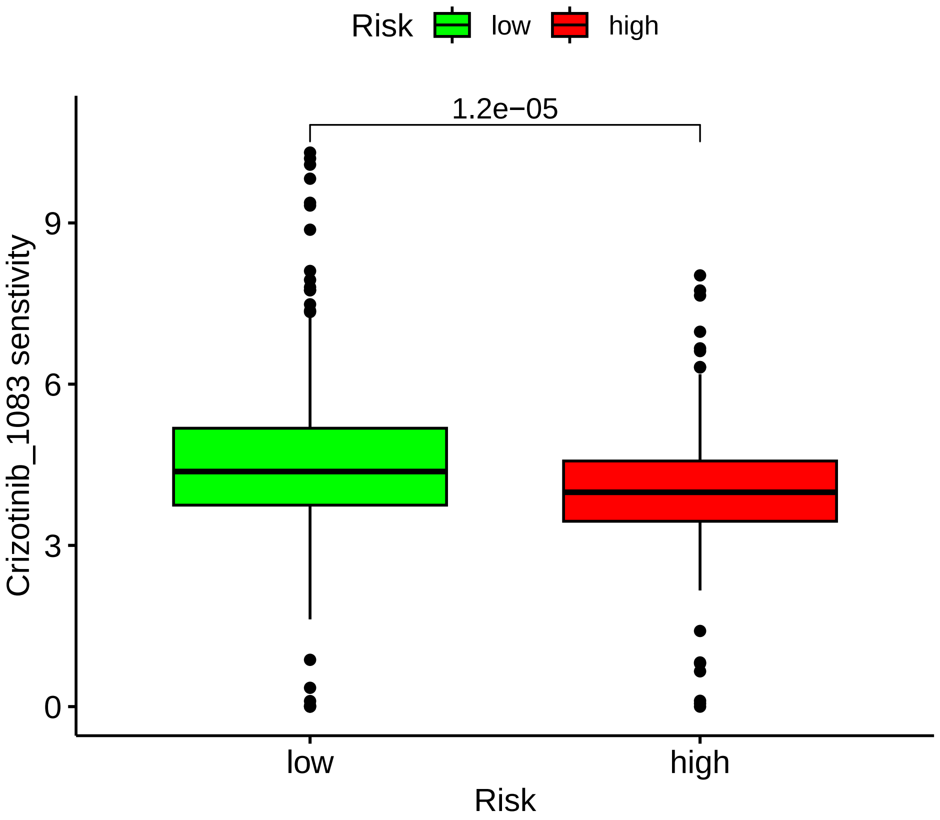

Risk
low
high
1.2e−05
Crizotinib_1083 senstivity
9
6
3
0
low
high
Risk

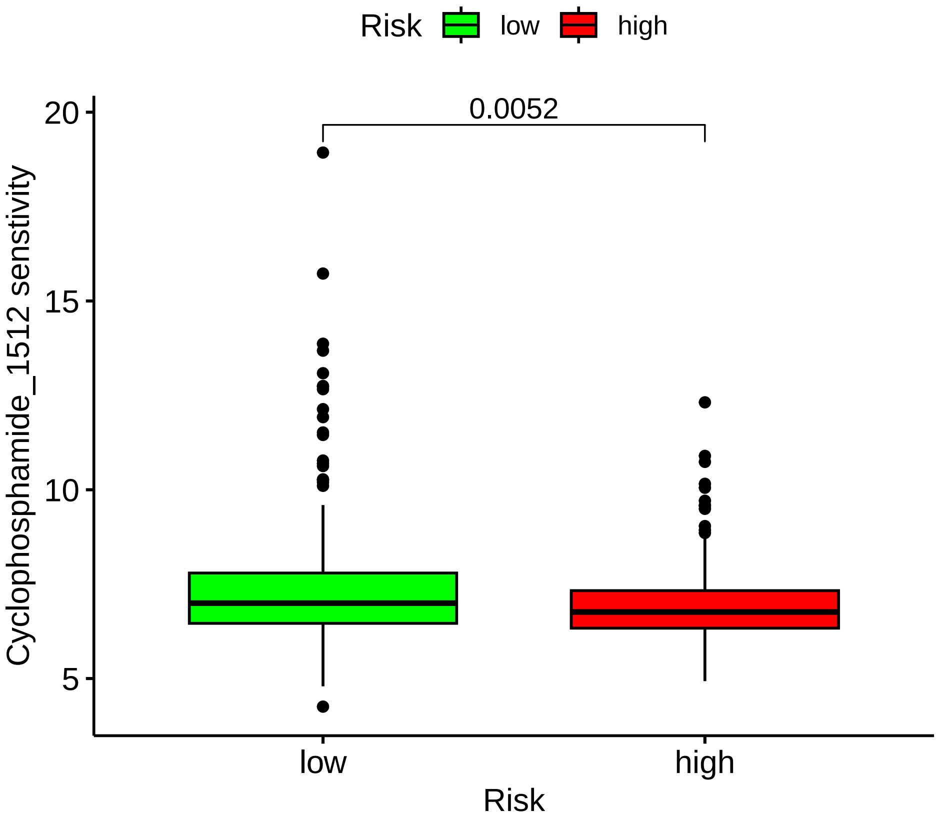
Risk
low
high
0.0052
20
15
10
5
Cyclophosphamide_1512 senstivity
low
high
Risk

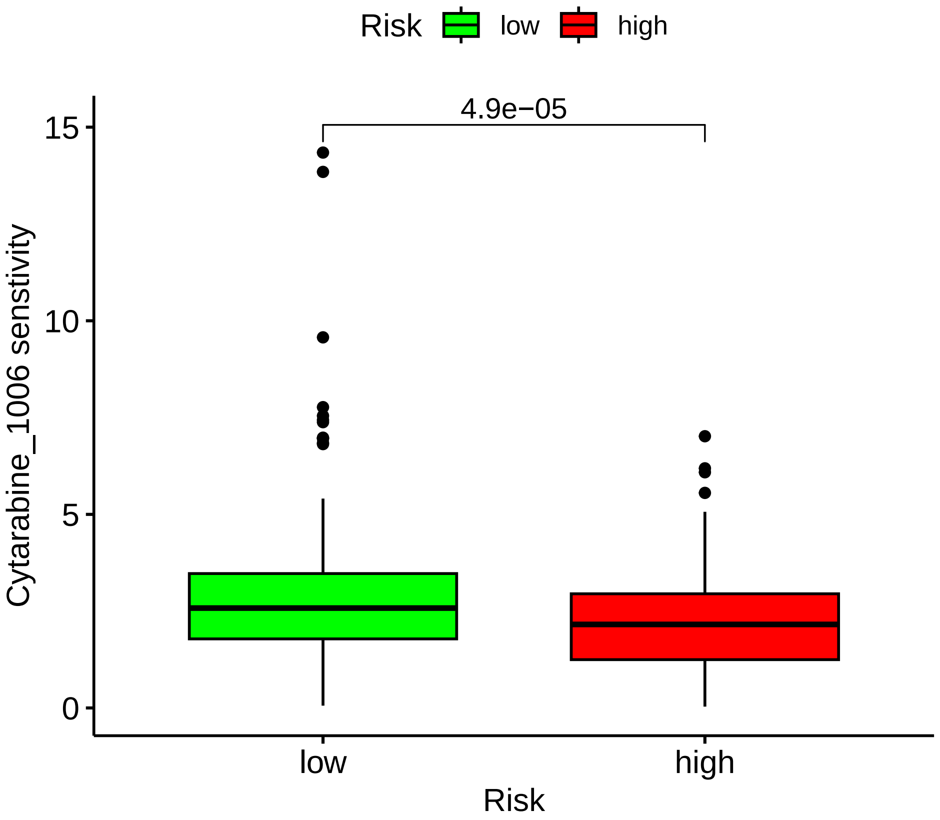

Risk
low
high
4.9e−05
15
10
5
0
Cytarabine_1006 senstivity
low
high
Risk

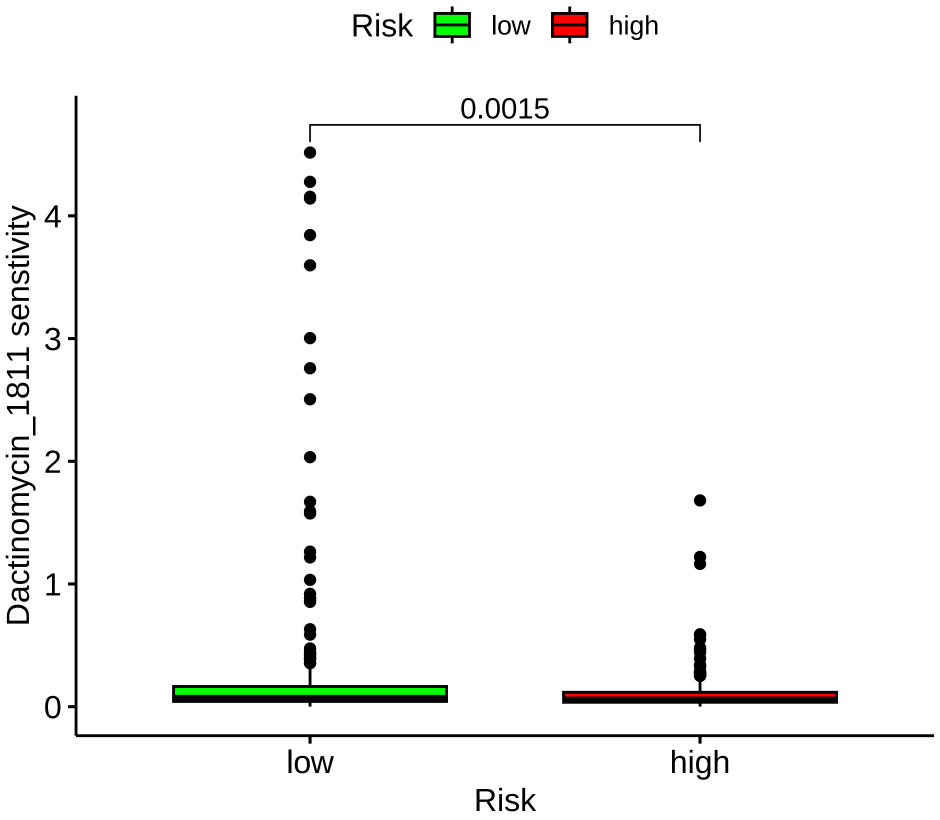

Risk
low
high
0.0015
Dactinomycin_1811 senstivity
4
3
2
1
0
low
high
Risk

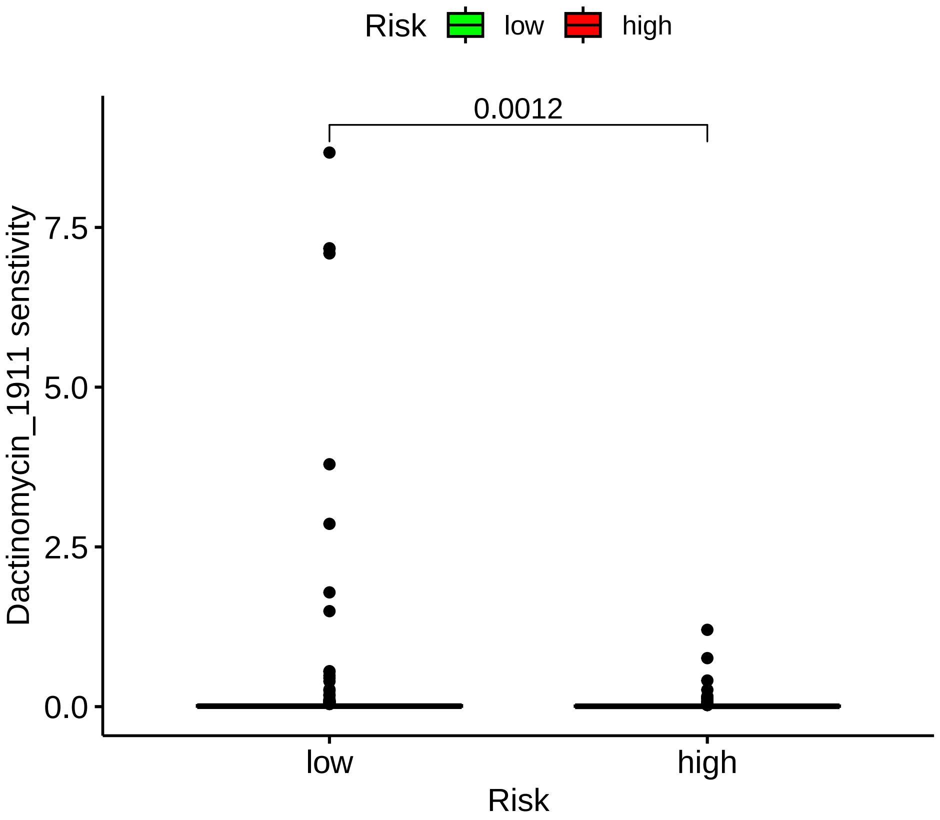

Risk
low
high
0.0012
Dactinomycin_1911 senstivity
7.5
5.0
2.5
0.0
low
high
Risk

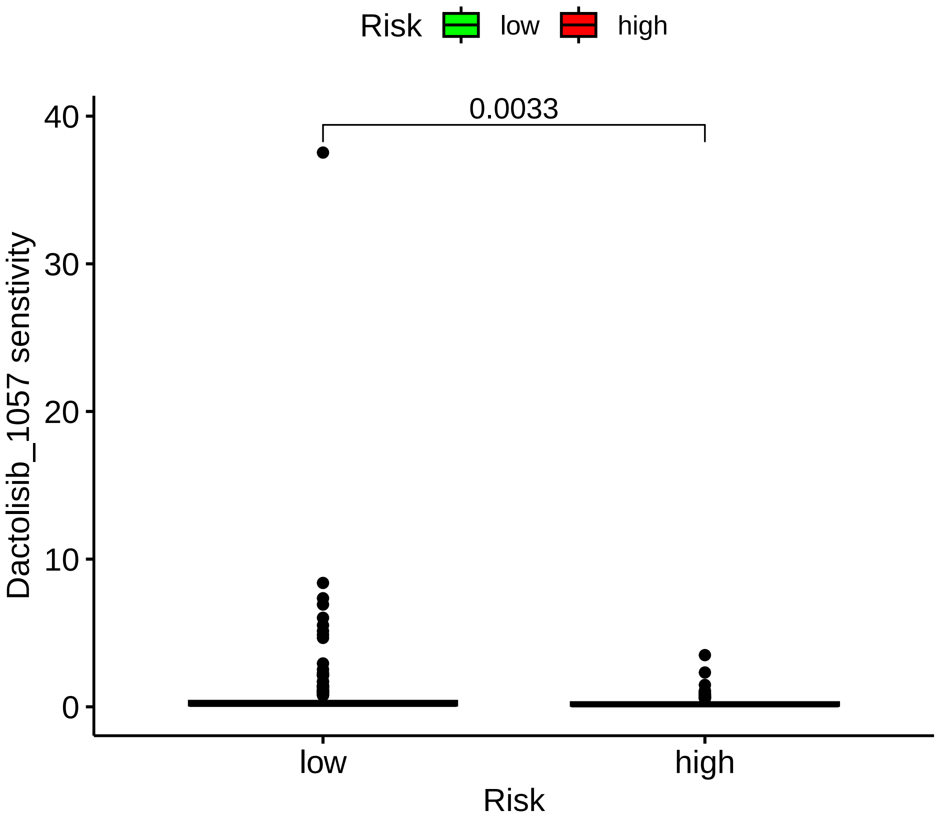

Risk
low
high
0.0033
Dactolisib_1057 senstivity
40
30
20
10
0
low
high
Risk

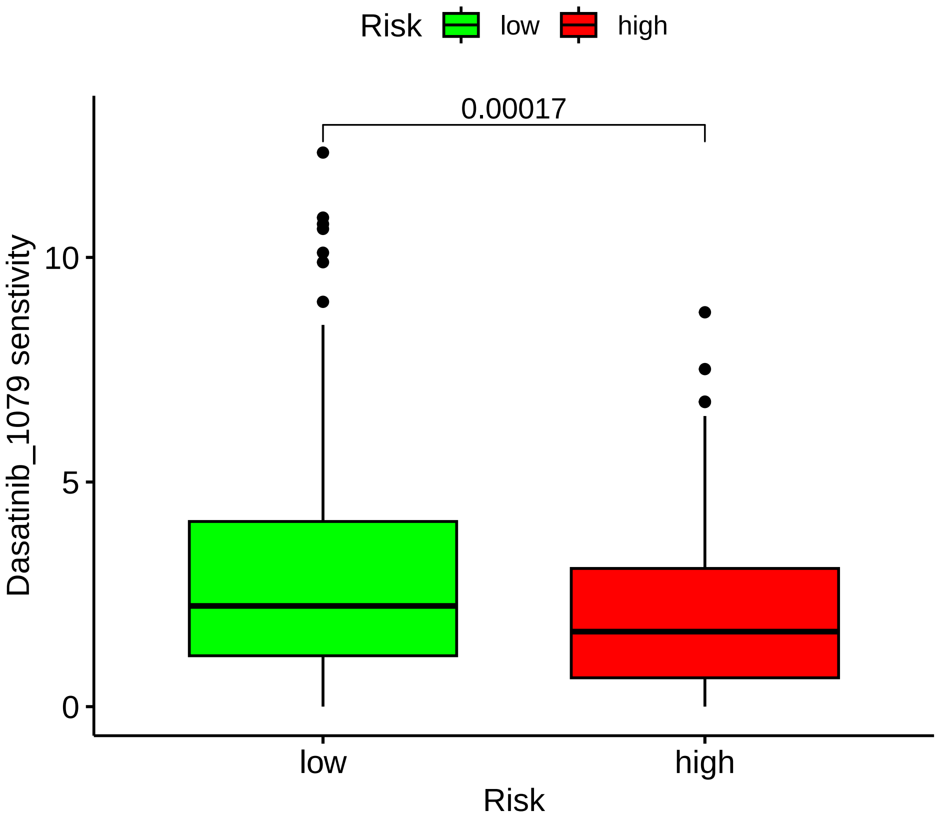
Risk
low
high
0.00017
Dasatinib_1079 senstivity
10
5
0
low
high
Risk

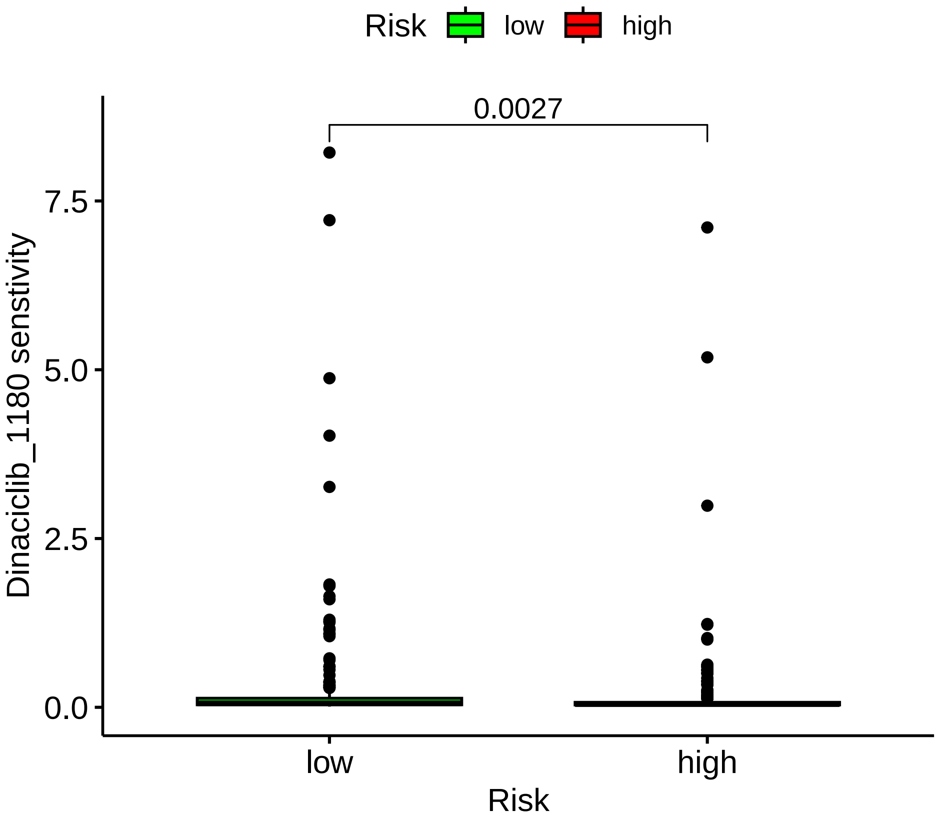
Risk
low
high
0.0027
Dinaciclib_1180 senstivity
7.5
5.0
2.5
0.0
low
high
Risk

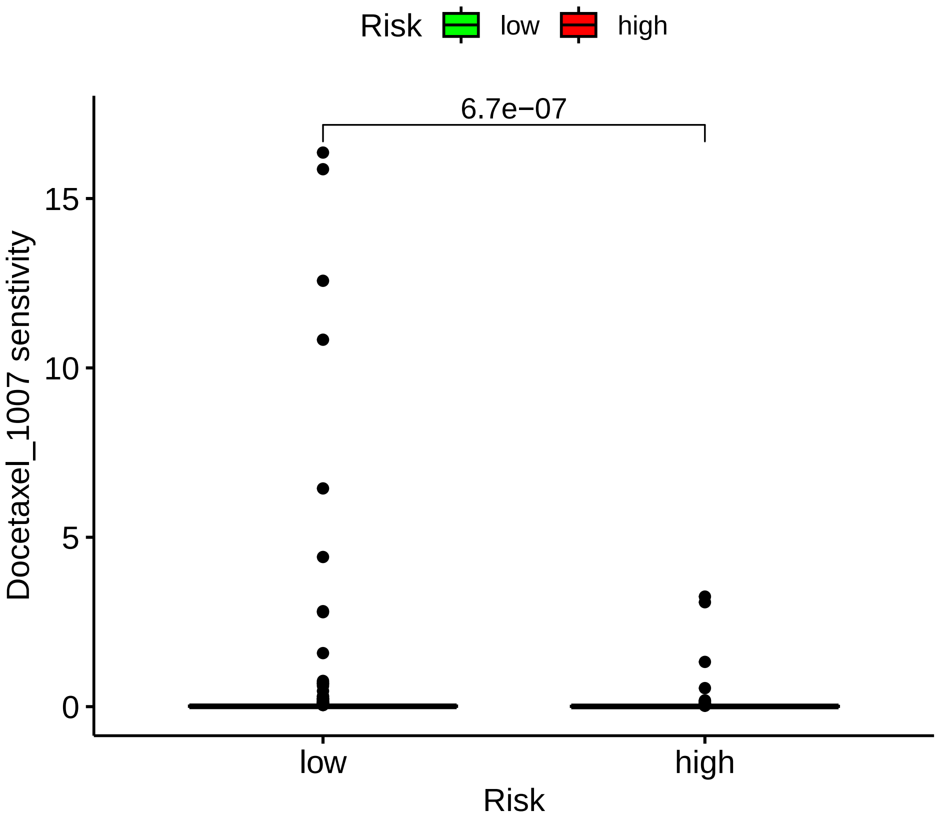

Risk
low
high
6.7e−07
Docetaxel_1007 senstivity
15
10
5
0
low
high
Risk

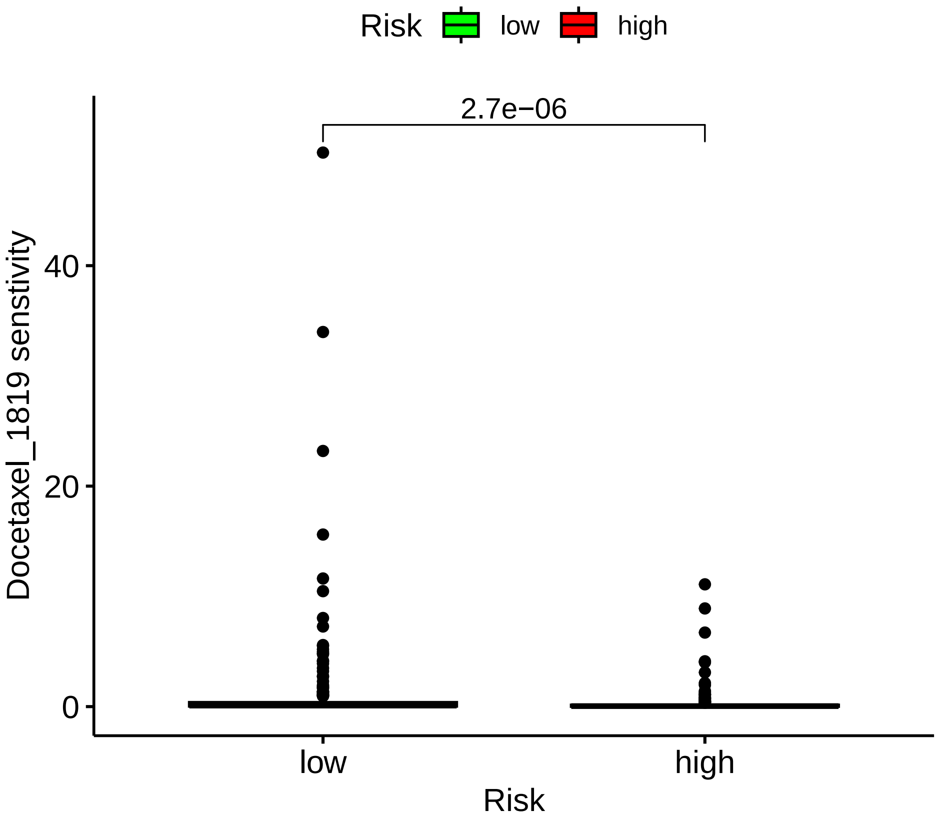

Risk
low
high
2.7e−06
Docetaxel_1819 senstivity
40
20
0
low
high
Risk

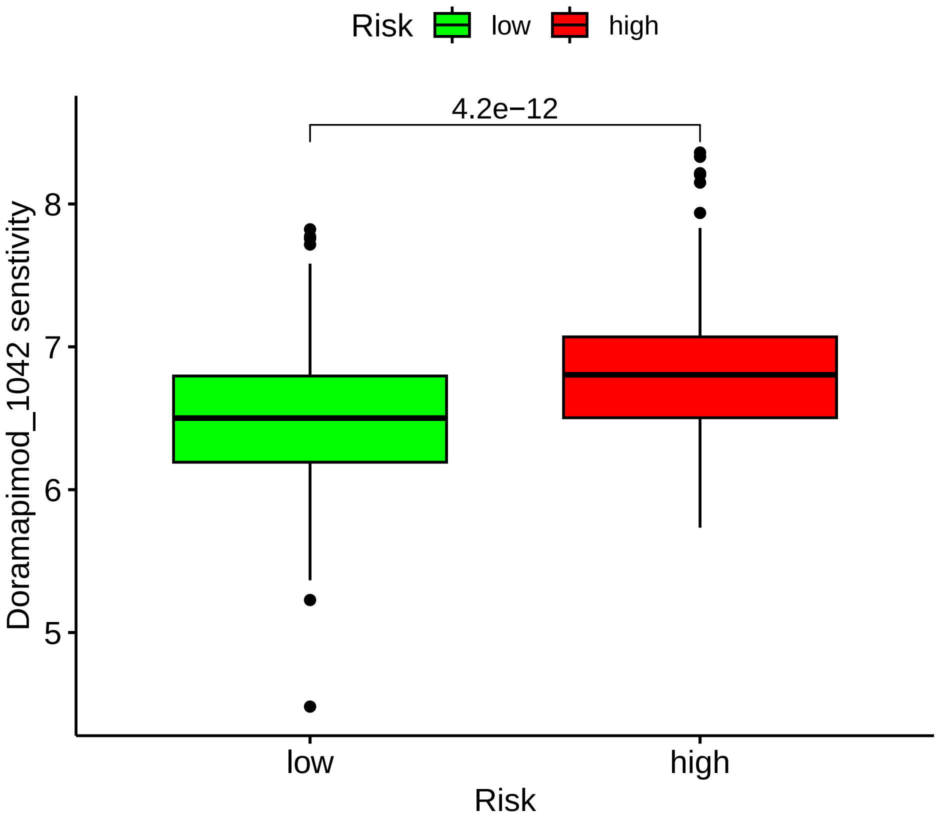
Risk
low
high
4.2e−12
Doramapimod_1042 senstivity
8
7
6
5
low
high
Risk

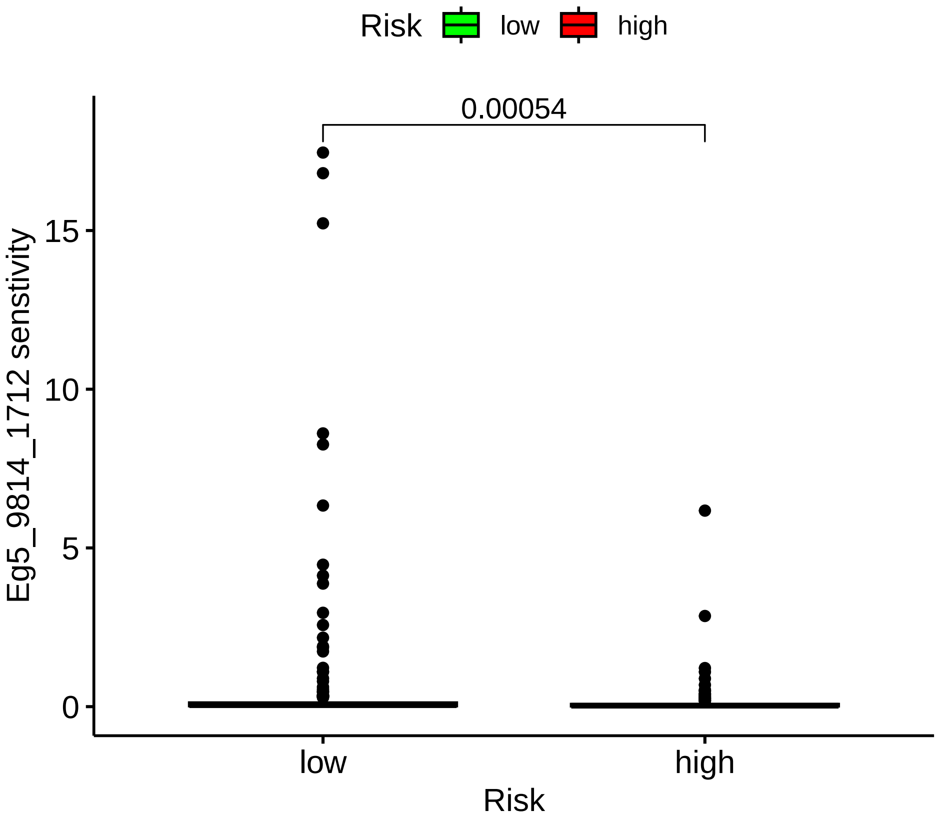

Risk
low
high
0.00054
Eg5_9814_1712 senstivity
15
10
5
0
low
high
Risk

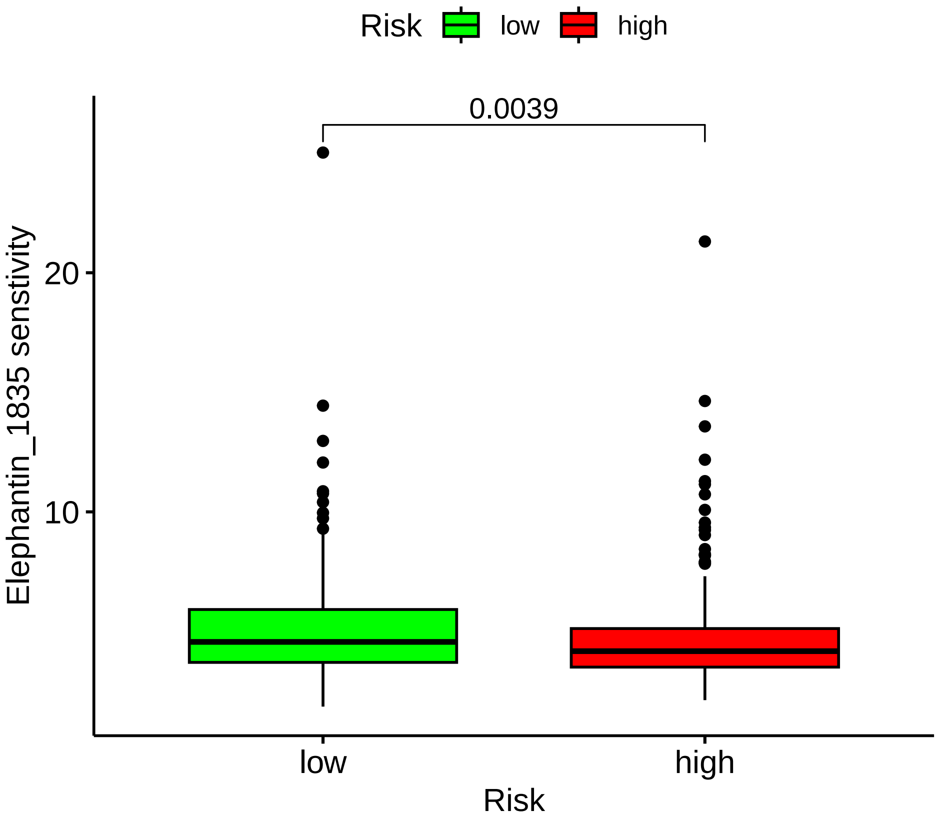

Risk
low
high
0.0039
20
10
Elephantin_1835 senstivity
low
high
Risk

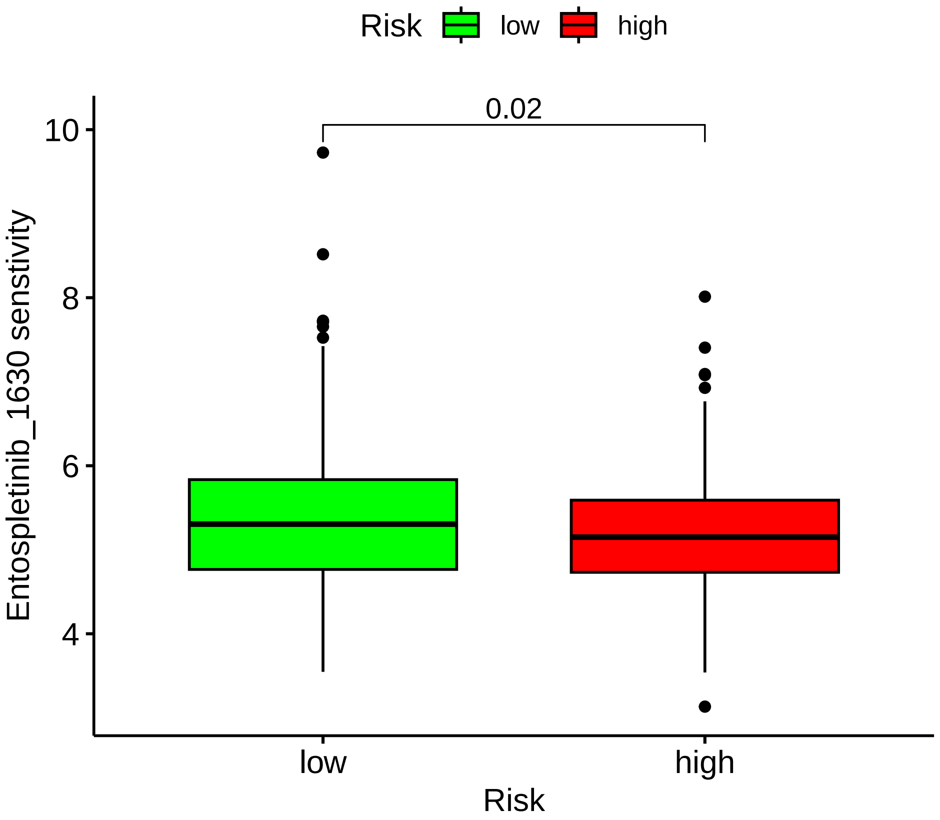
Risk
low
high
0.02
10
8
6
4
Entospletinib_1630 senstivity
low
high
Risk

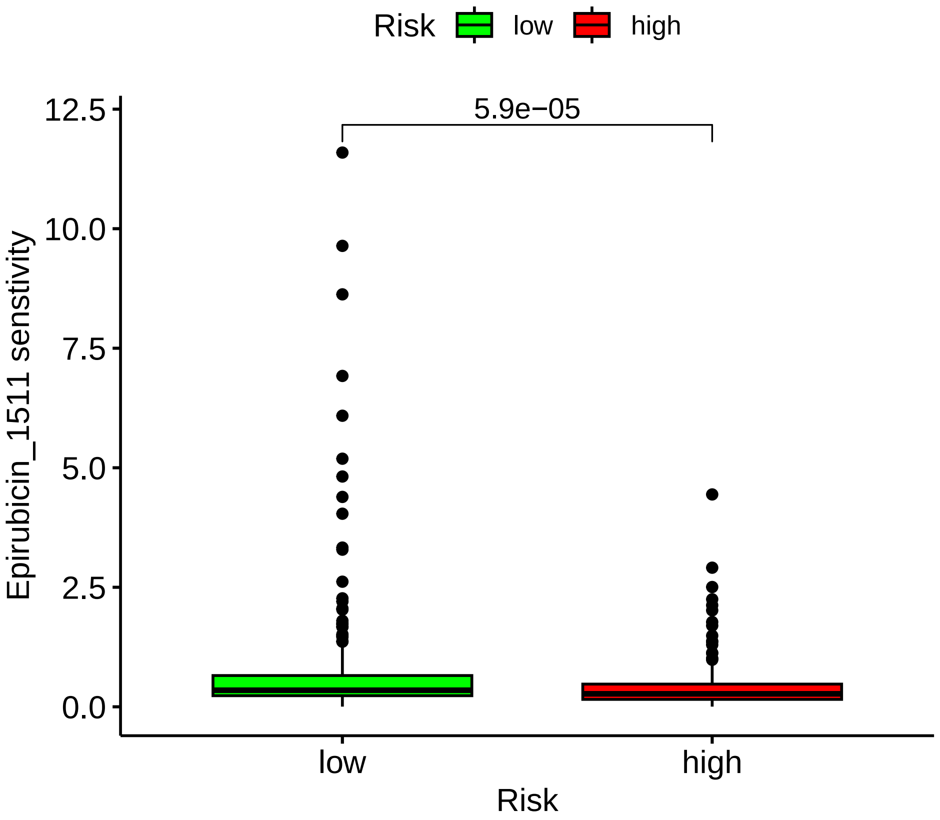

Risk
low
high
5.9e−05
12.5
10.0
7.5
5.0
2.5
0.0
Epirubicin_1511 senstivity
low
high
Risk

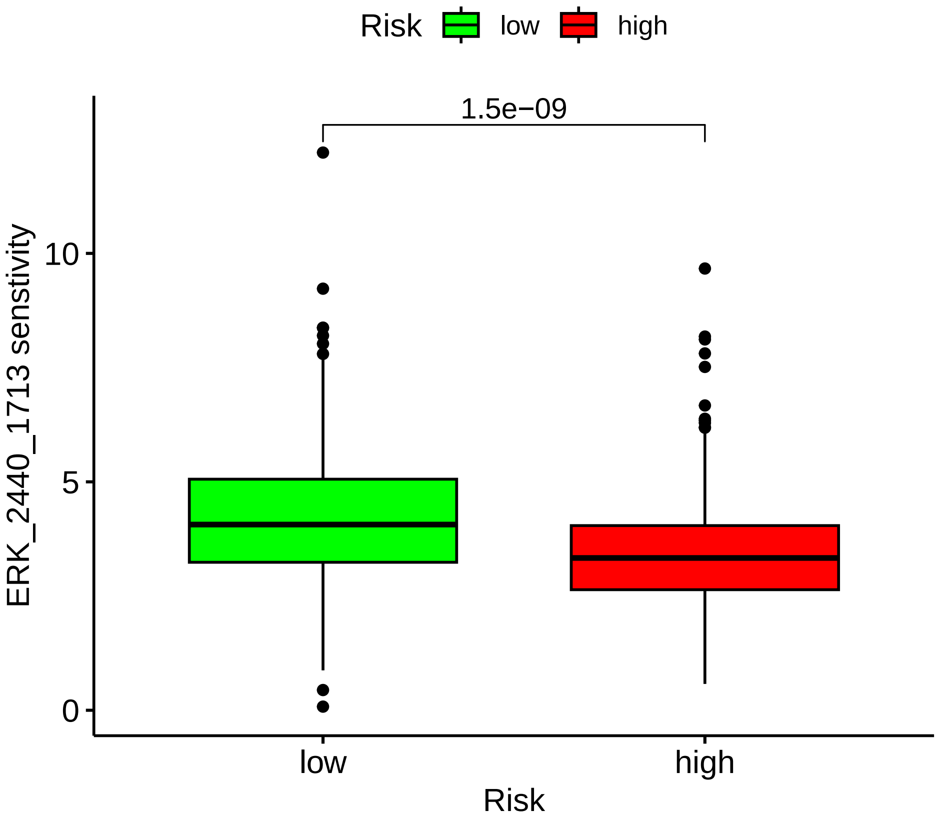

Risk
low
high
1.5e−09
ERK_2440_1713 senstivity
10
5
0
low
high
Risk

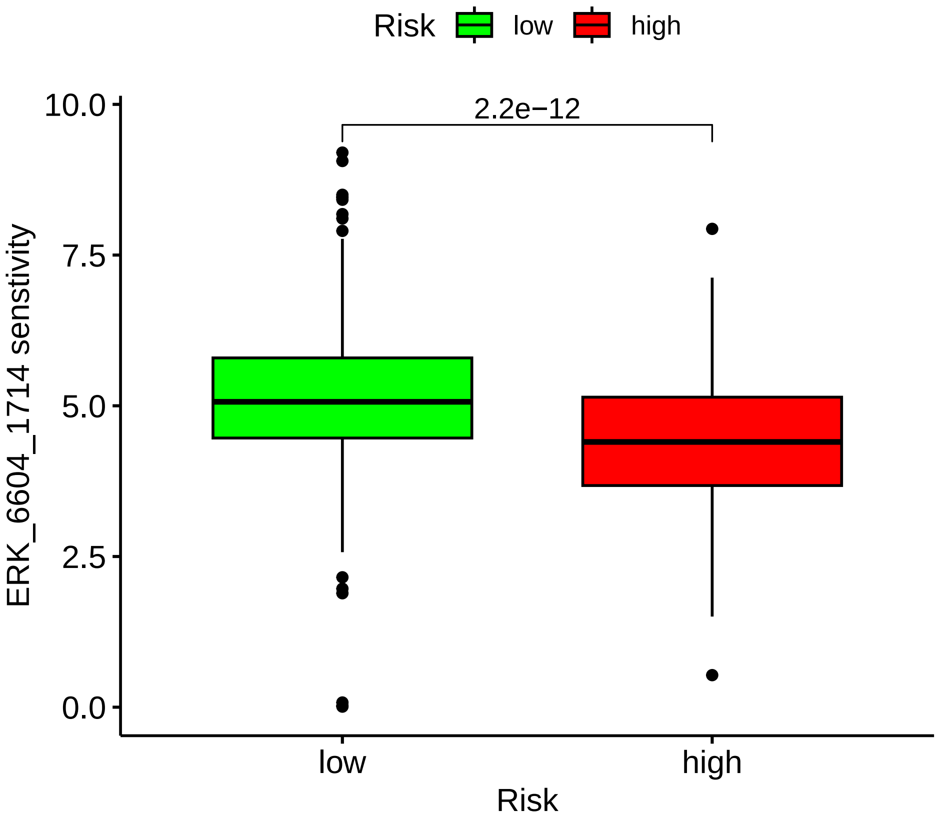
Risk
low
high
2.2e−12
10.0
7.5
5.0
2.5
0.0
ERK_6604_1714 senstivity
low
high
Risk

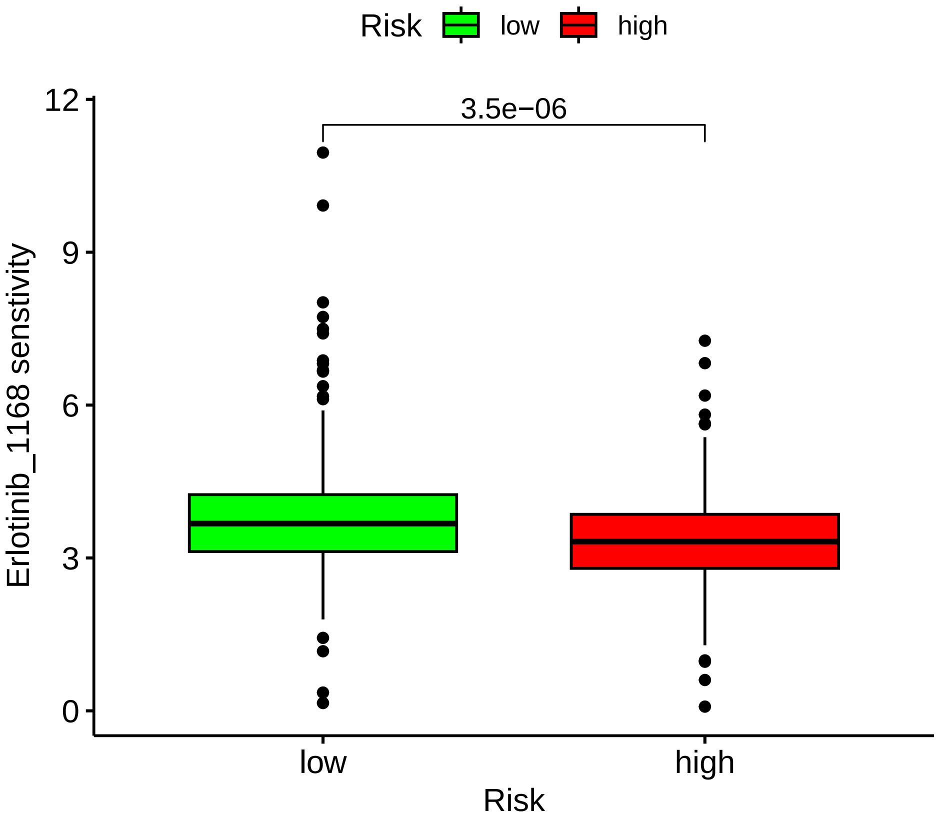
Risk
low
high
3.5e−06
12
9
6
3
0
Erlotinib_1168 senstivity
low
high
Risk

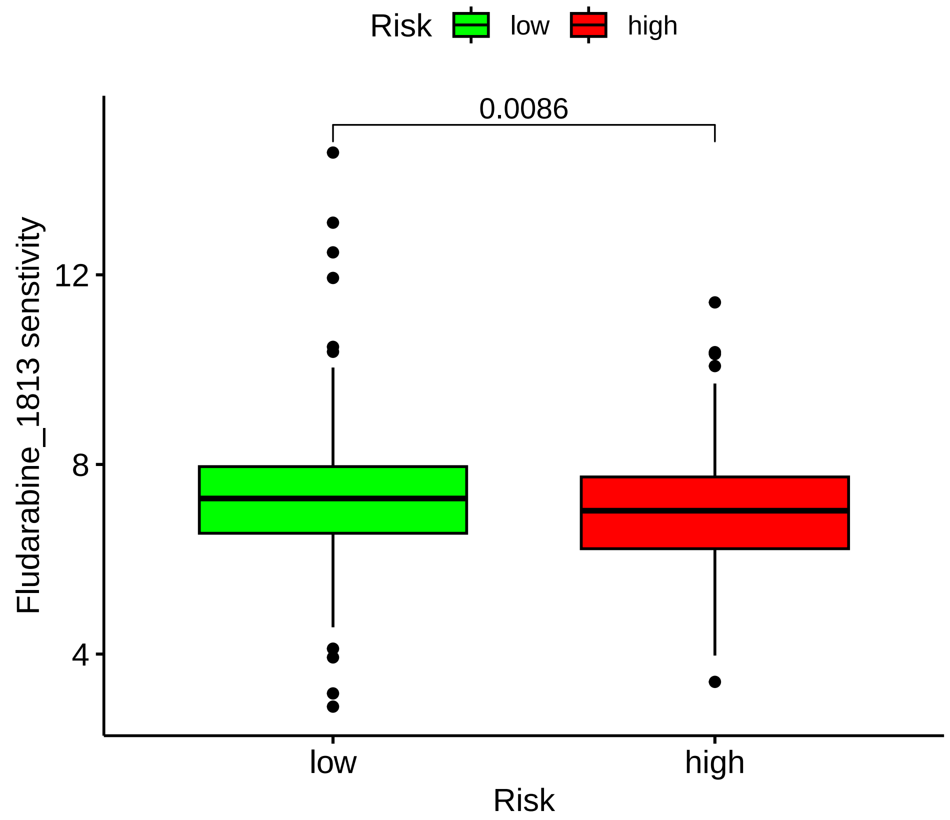
Risk
low
high
0.0086
Fludarabine_1813 senstivity
12
8
4
low
high
Risk

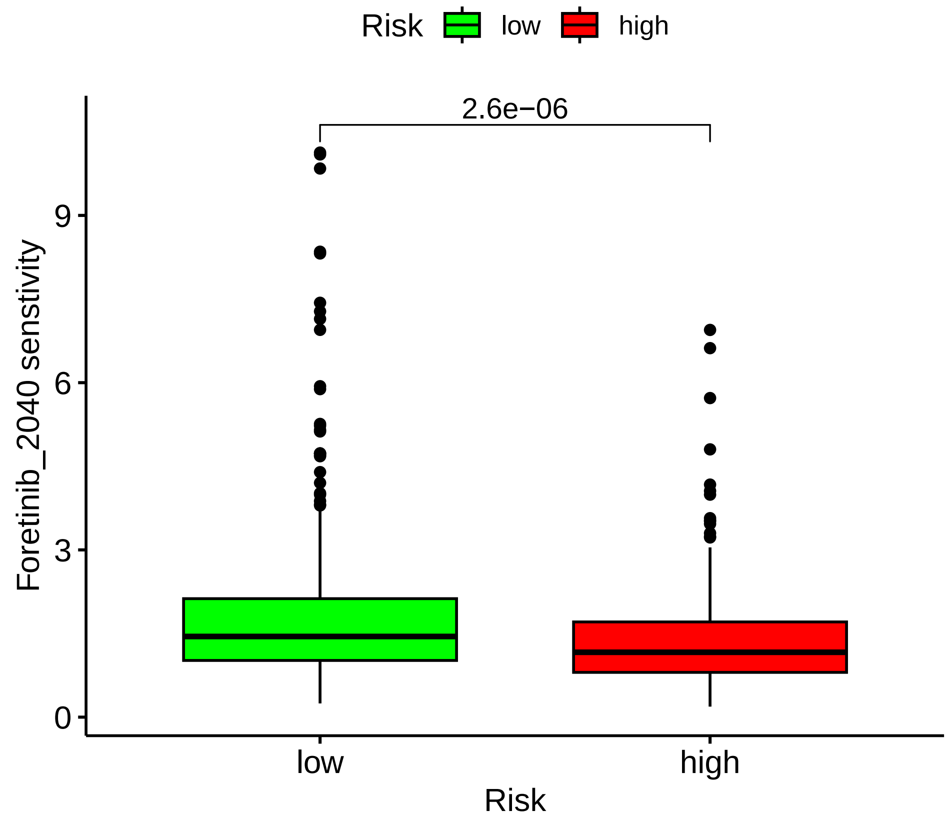

Risk
low
high
2.6e−06
Foretinib_2040 senstivity
9
6
3
0
low
high
Risk

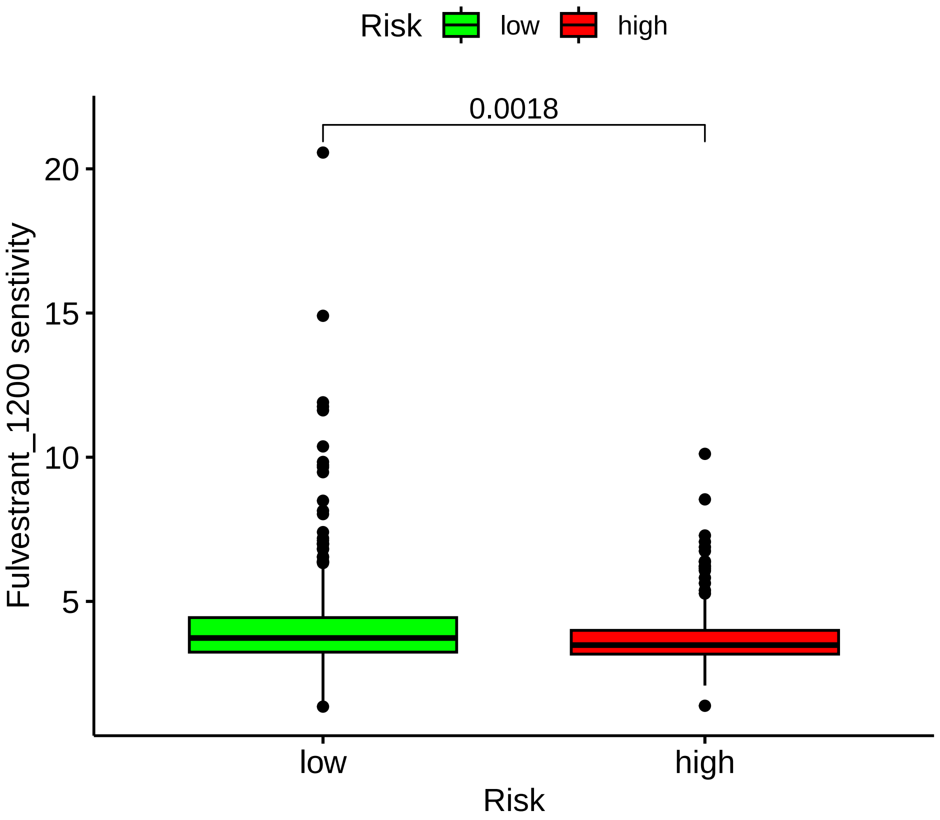

Risk
low
high
0.0018
20
15
10
5
Fulvestrant_1200 senstivity
low
high
Risk

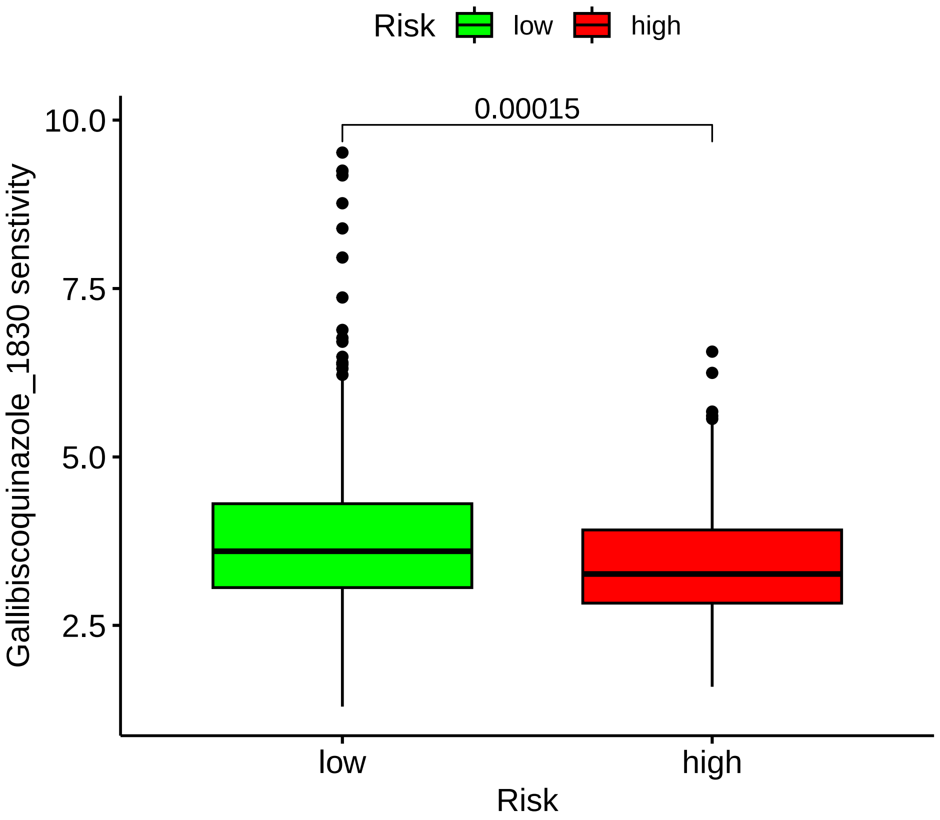

Risk
low
high
0.00015
10.0
7.5
5.0
2.5
Gallibiscoquinazole_1830 senstivity
low
high
Risk

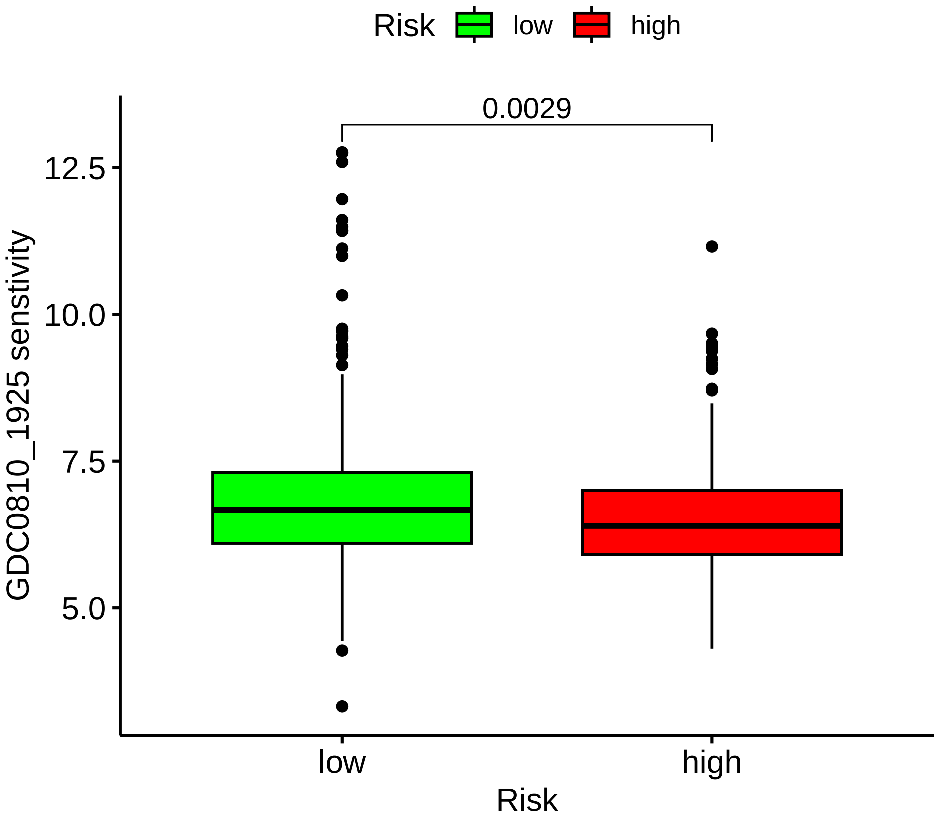

Risk
low
high
0.0029
12.5
10.0
7.5
5.0
GDC0810_1925 senstivity
low
high
Risk

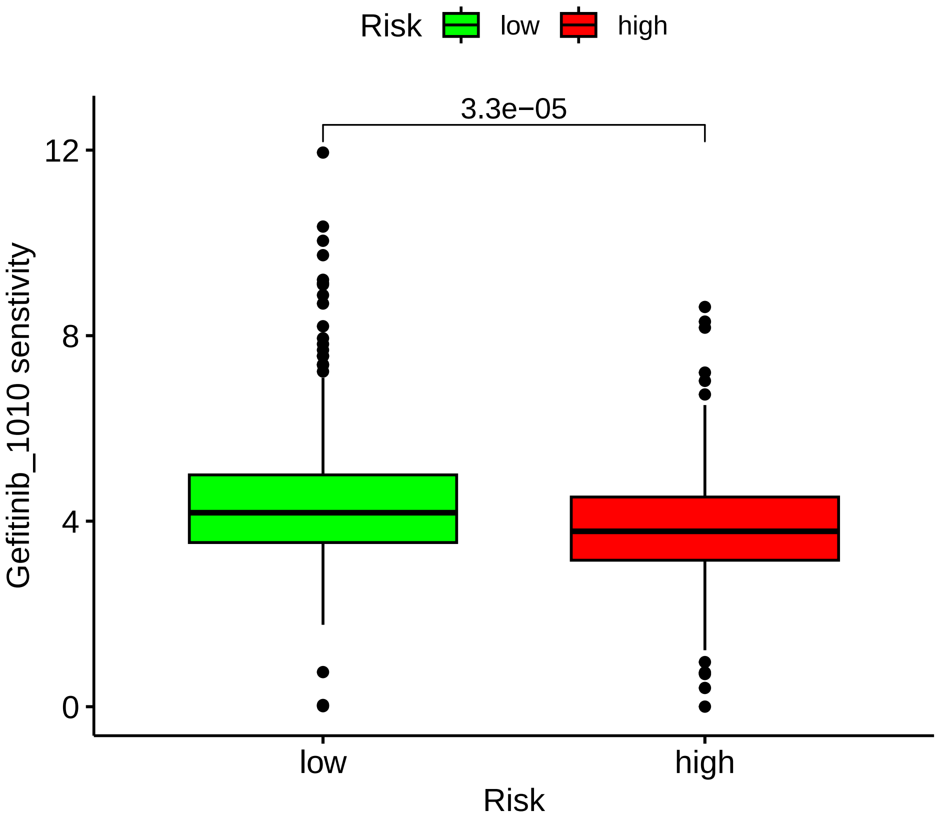
Risk
low
high
3.3e−05
12
8
4
0
Gefitinib_1010 senstivity
low
high
Risk

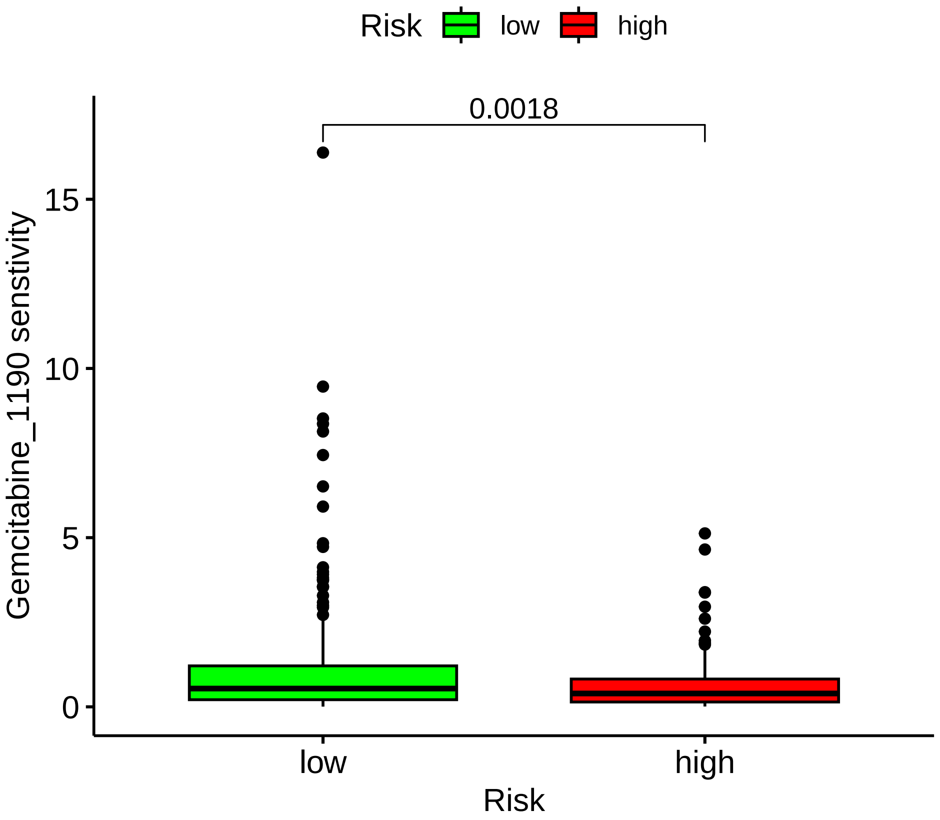
Risk
low
high
0.0018
Gemcitabine_1190 senstivity
15
10
5
0
low
high
Risk

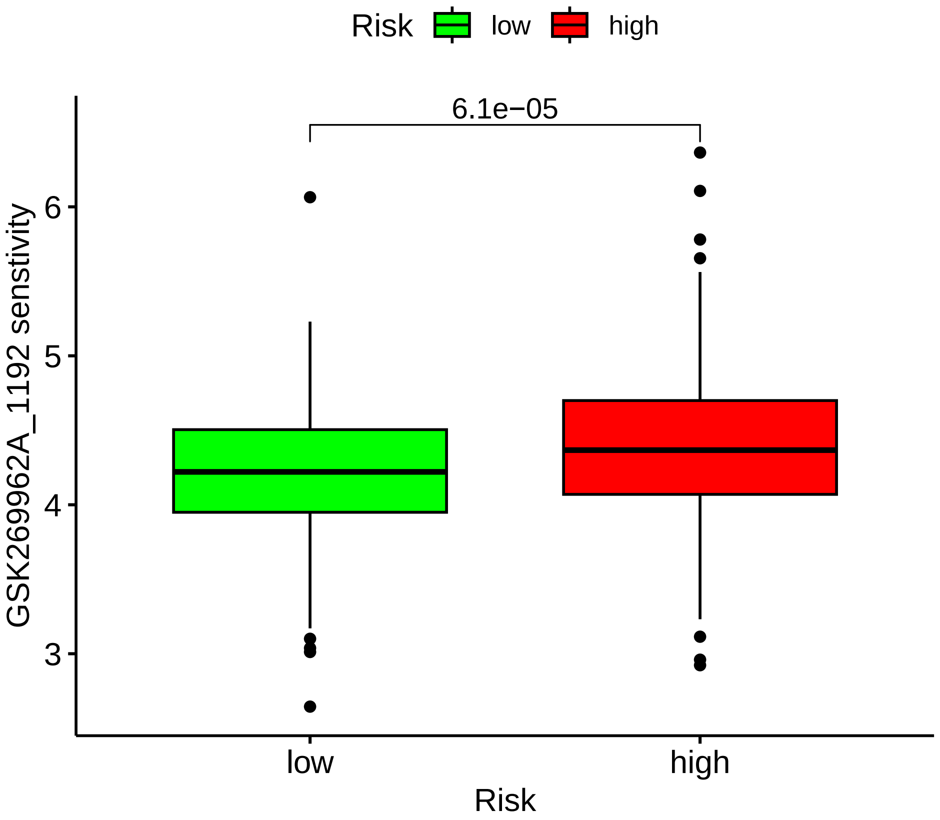

Risk
low
high
6.1e−05
GSK269962A_1192 senstivity
6
5
4
3
low
high
Risk

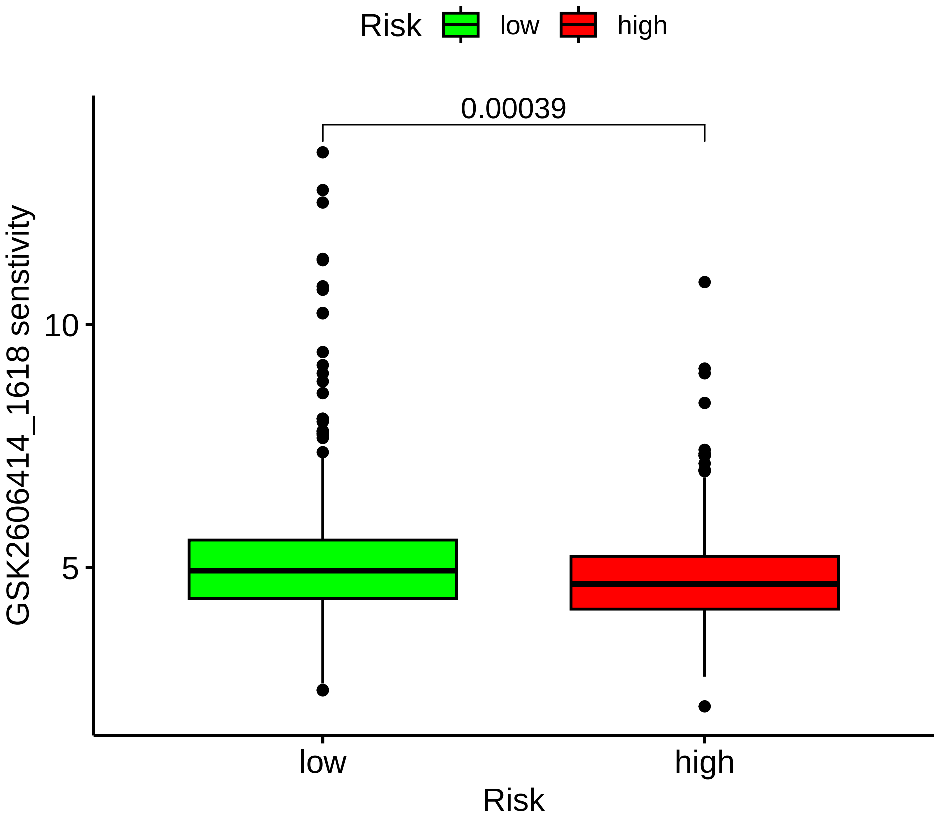
Risk
low
high
0.00039
GSK2606414_1618 senstivity
10
5
low
high
Risk

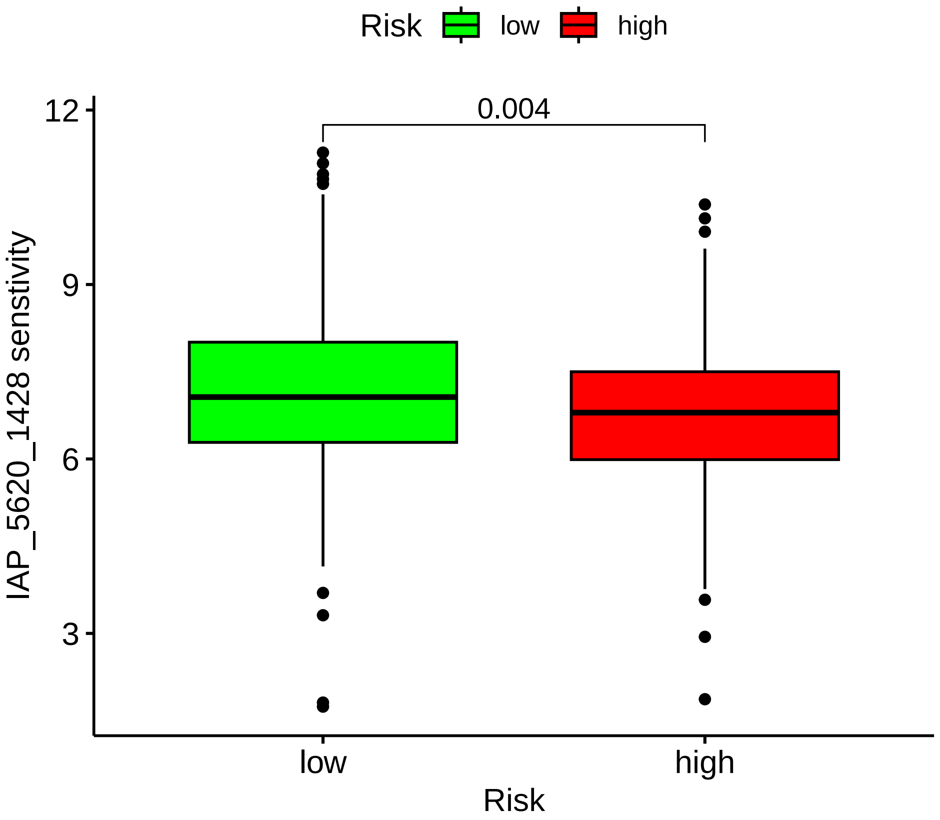

Risk
low
high
0.004
12
9
6
3
IAP_5620_1428 senstivity
low
high
Risk

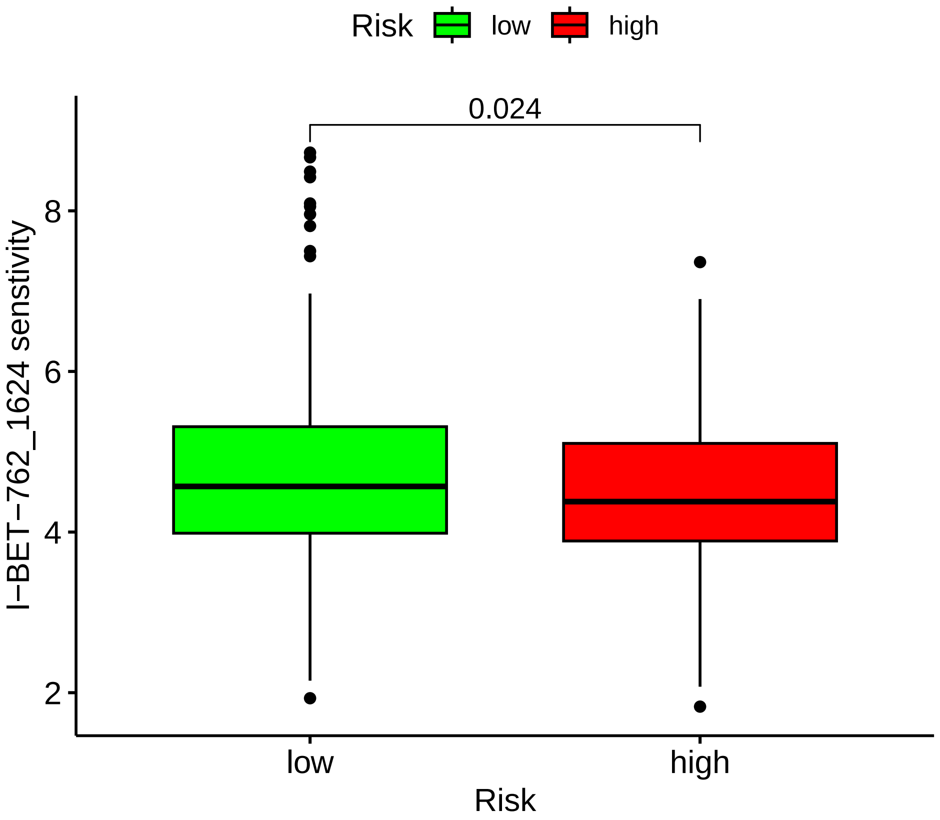
Risk
low
high
0.024
I−BET−762_1624 senstivity
8
6
4
2
low
high
Risk

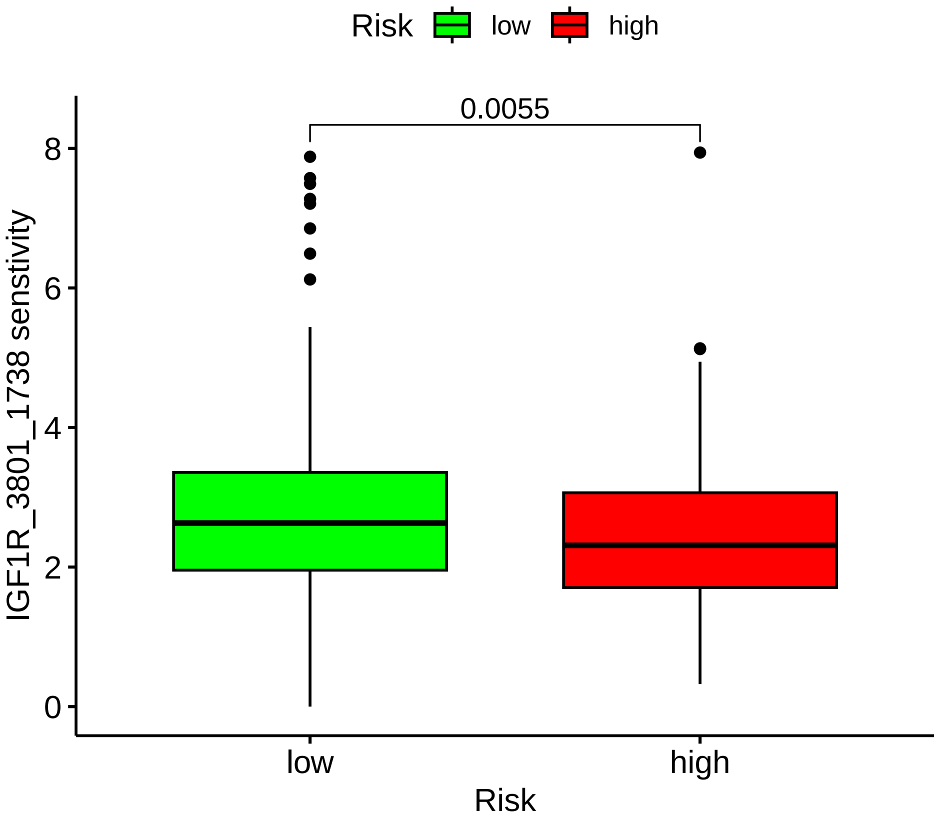

Risk
low
high
0.0055
8
6
4
2
0
IGF1R_3801_1738 senstivity
low
high
Risk

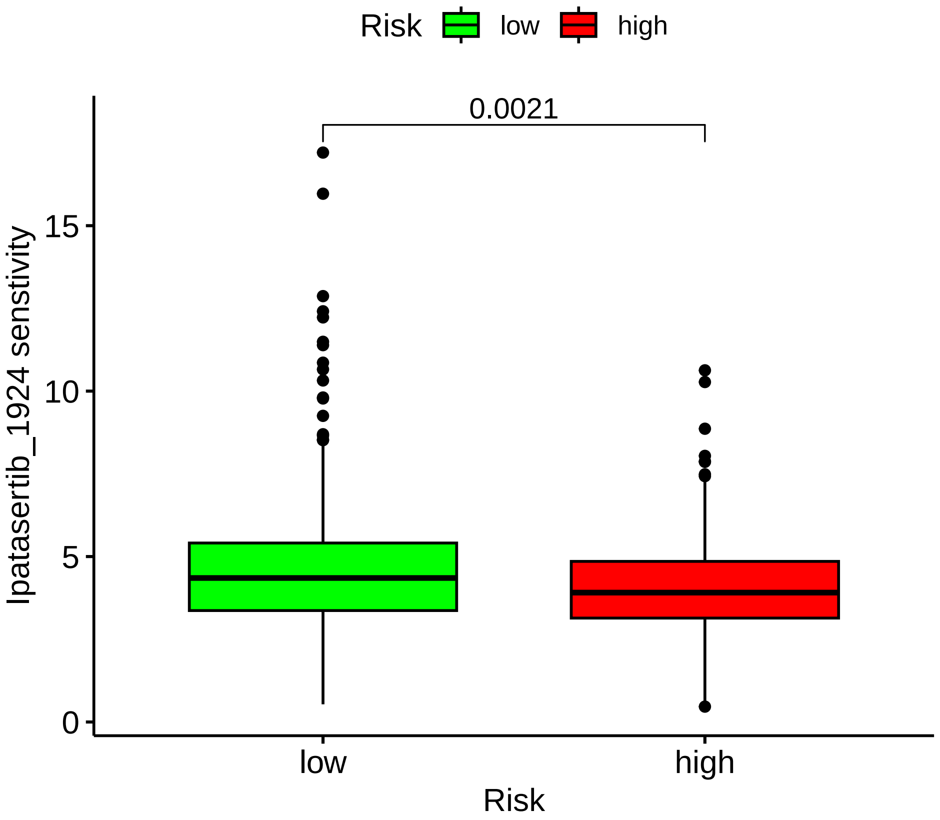

Risk
low
high
0.0021
Ipatasertib_1924 senstivity
15
10
5
0
low
high
Risk

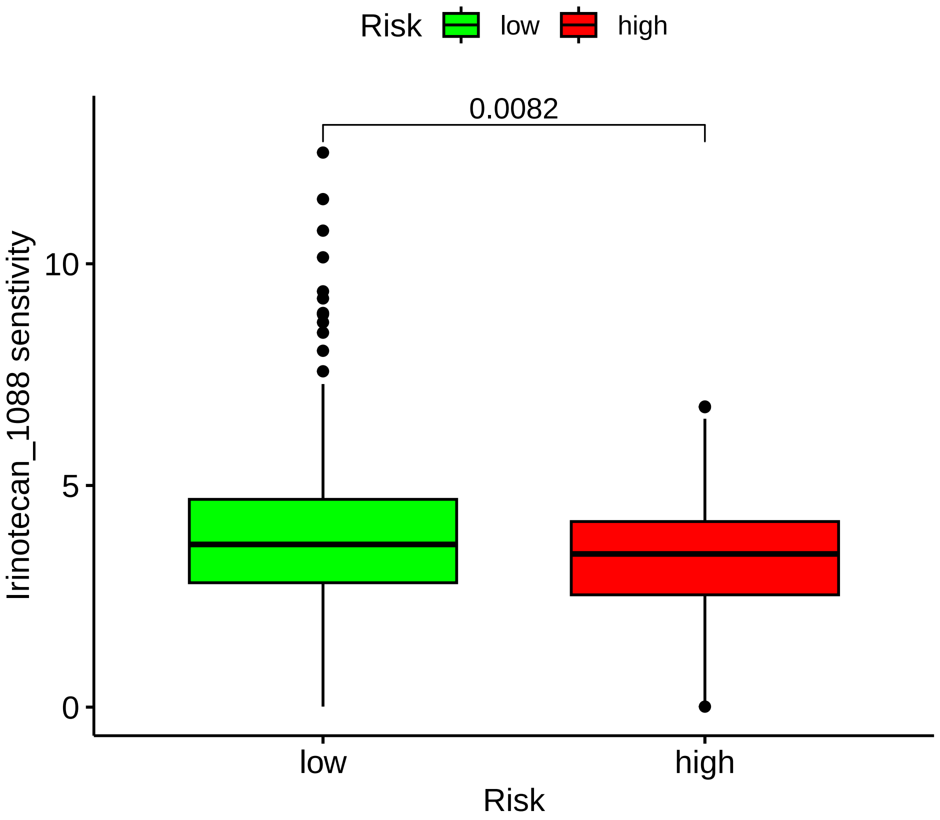

Risk
low
high
0.0082
Irinotecan_1088 senstivity
10
5
0
low
high
Risk

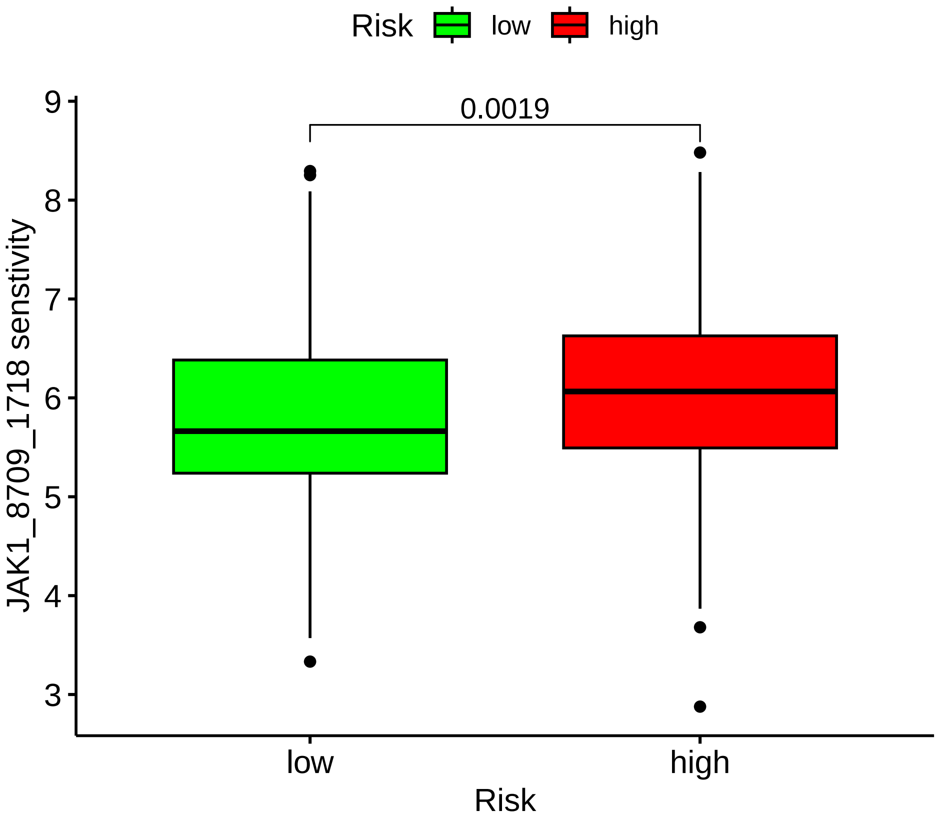
Risk
low
high
0.0019
JAK1_8709_1718 senstivity
9
8
7
6
5
4
3
low
high
Risk

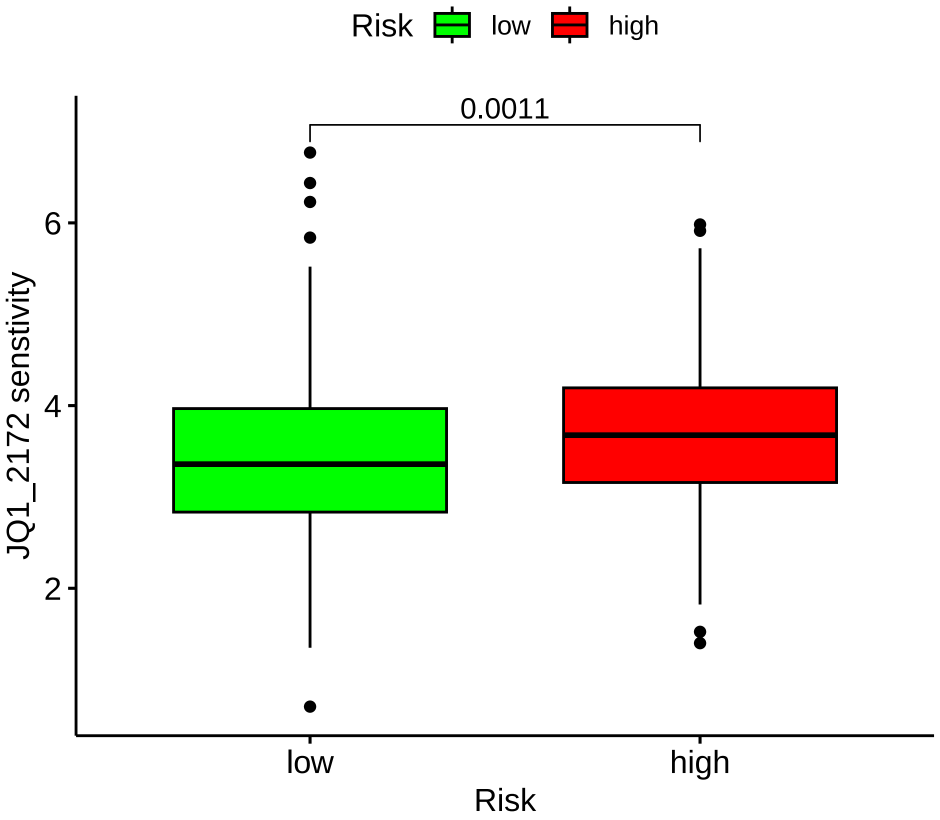
Risk
low
high
0.0011
JQ1_2172 senstivity
6
4
2
low
high
Risk

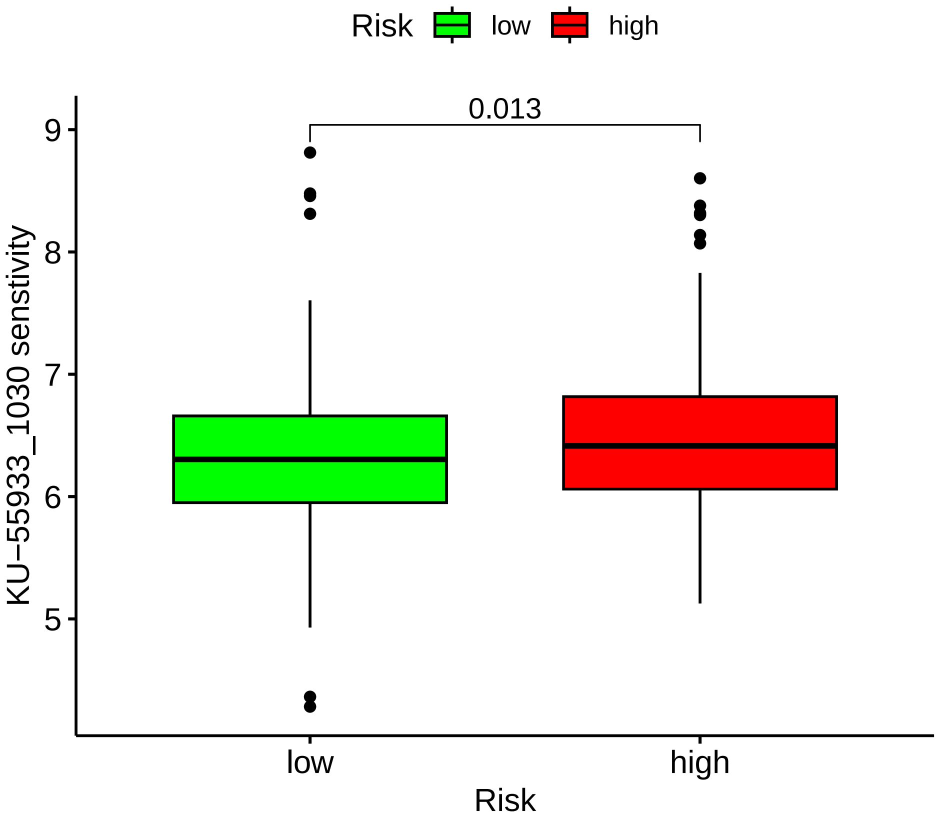

Risk
low
high
0.013
9
8
7
6
5
KU−55933_1030 senstivity
low
high
Risk

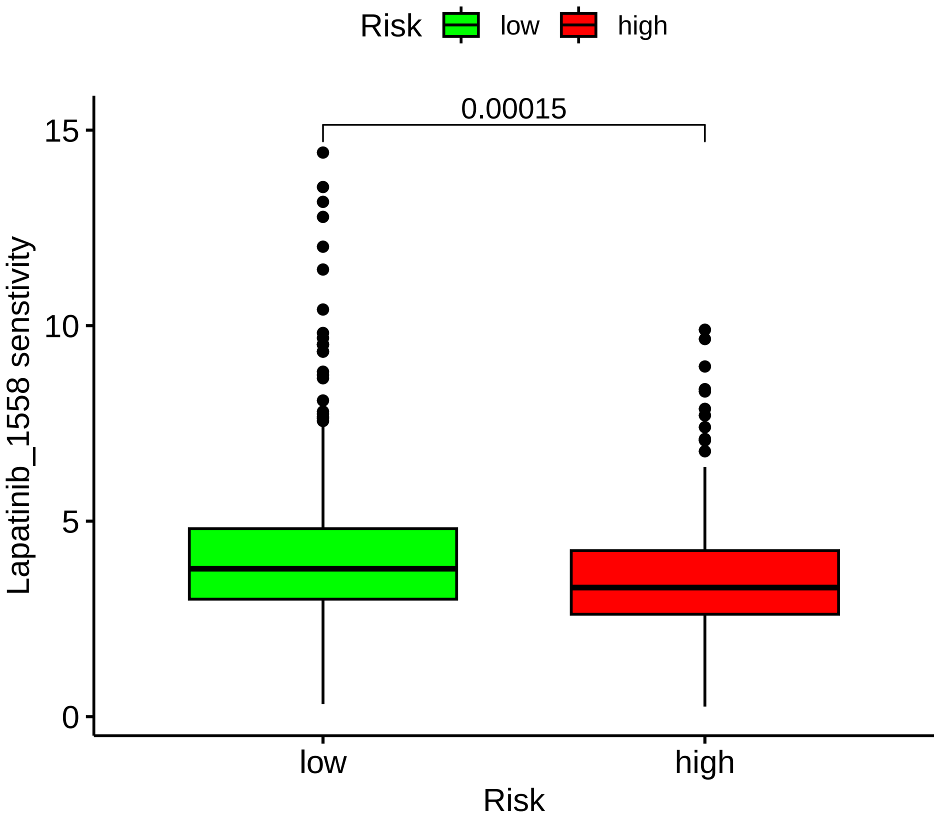

Risk
low
high
0.00015
15
10
5
0
Lapatinib_1558 senstivity
low
high
Risk

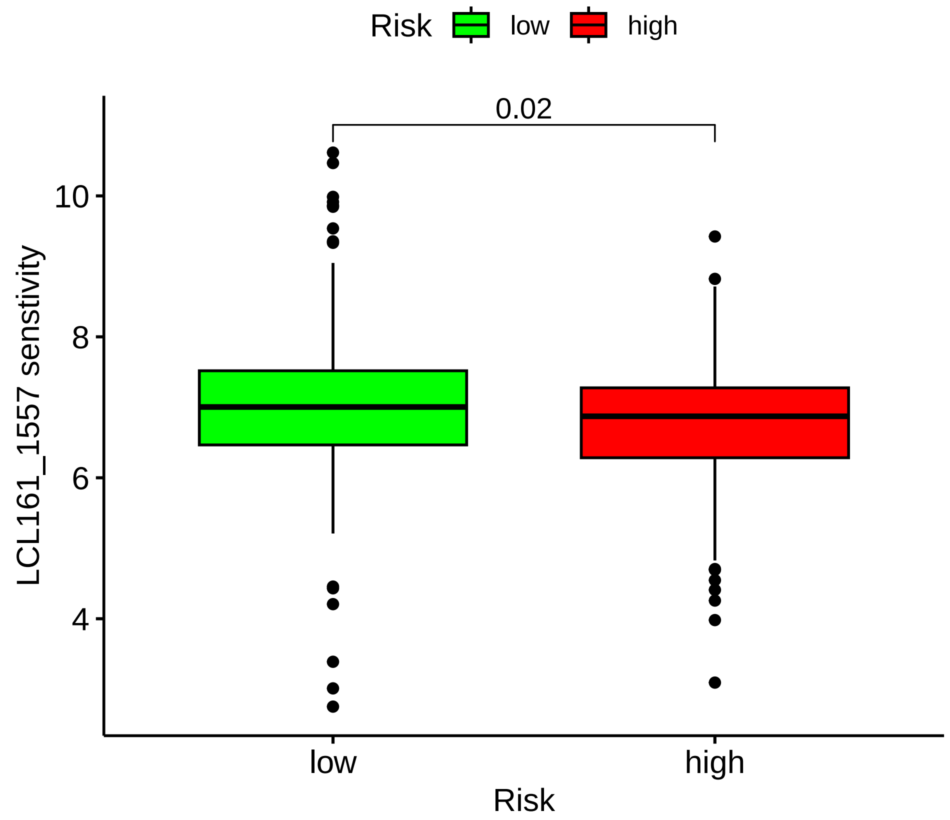

Risk
low
high
0.02
LCL161_1557 senstivity
10
8
6
4
low
high
Risk

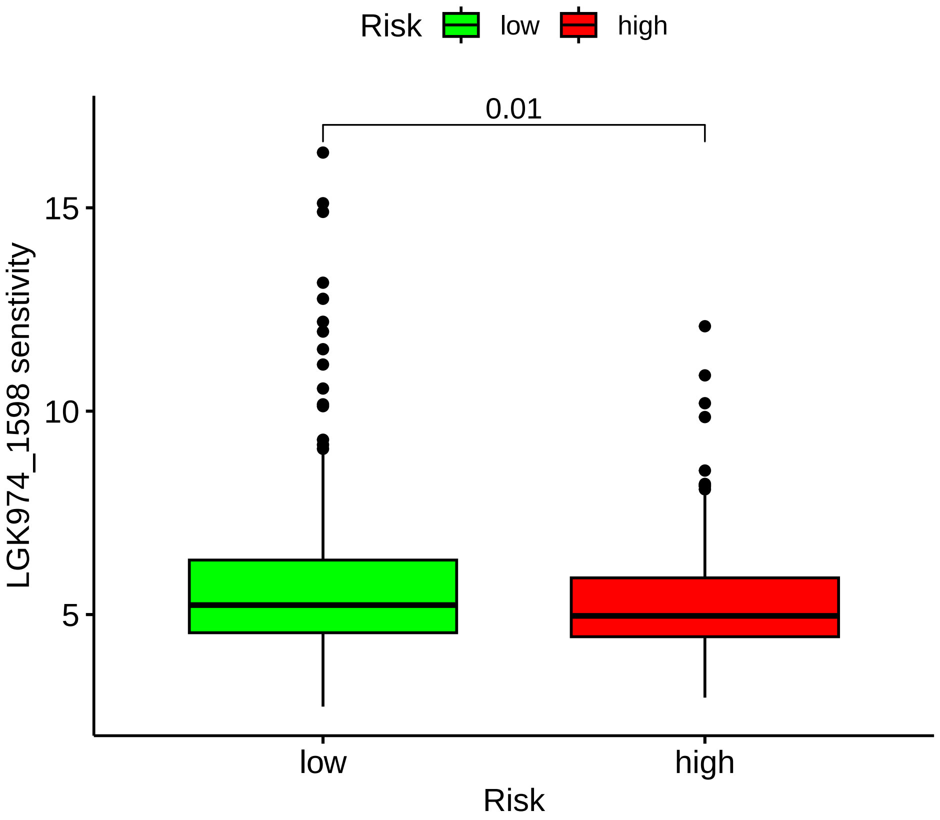

Risk
low
high
0.01
15
10
5
LGK974_1598 senstivity
low
high
Risk

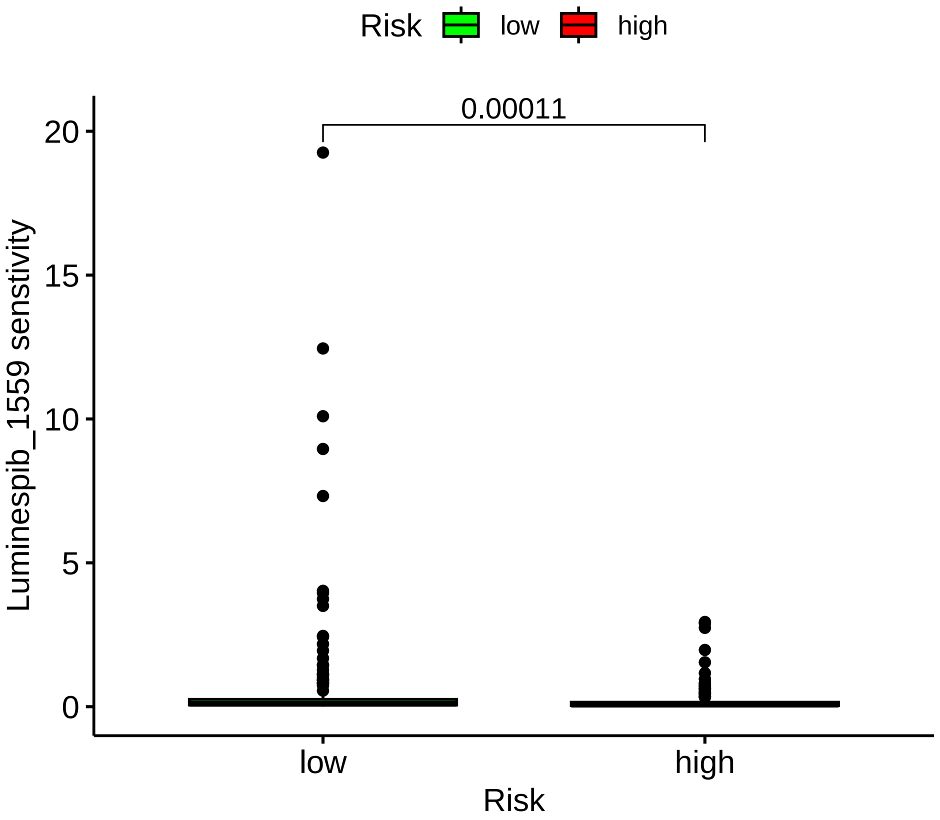

Risk
low
high
0.00011
Luminespib_1559 senstivity
20
15
10
5
0
low
high
Risk

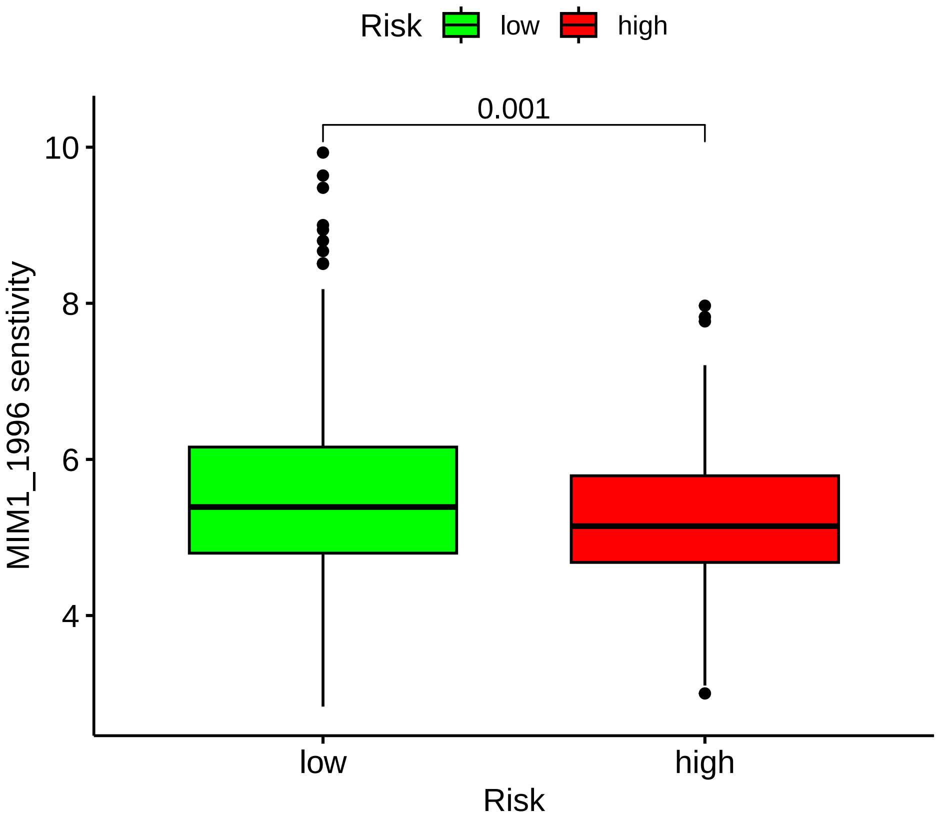

Risk
low
high
0.001
10
8
6
4
MIM1_1996 senstivity
low
high
Risk

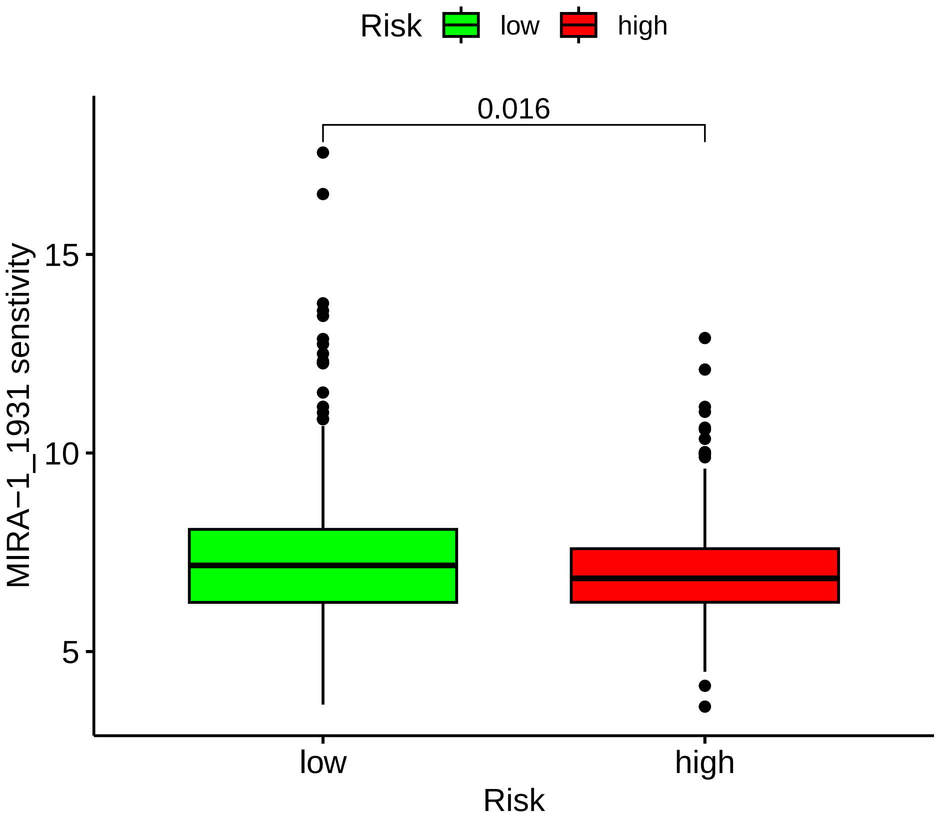
Risk
low
high
0.016
MIRA−1_1931 senstivity
15
10
5
low
high
Risk

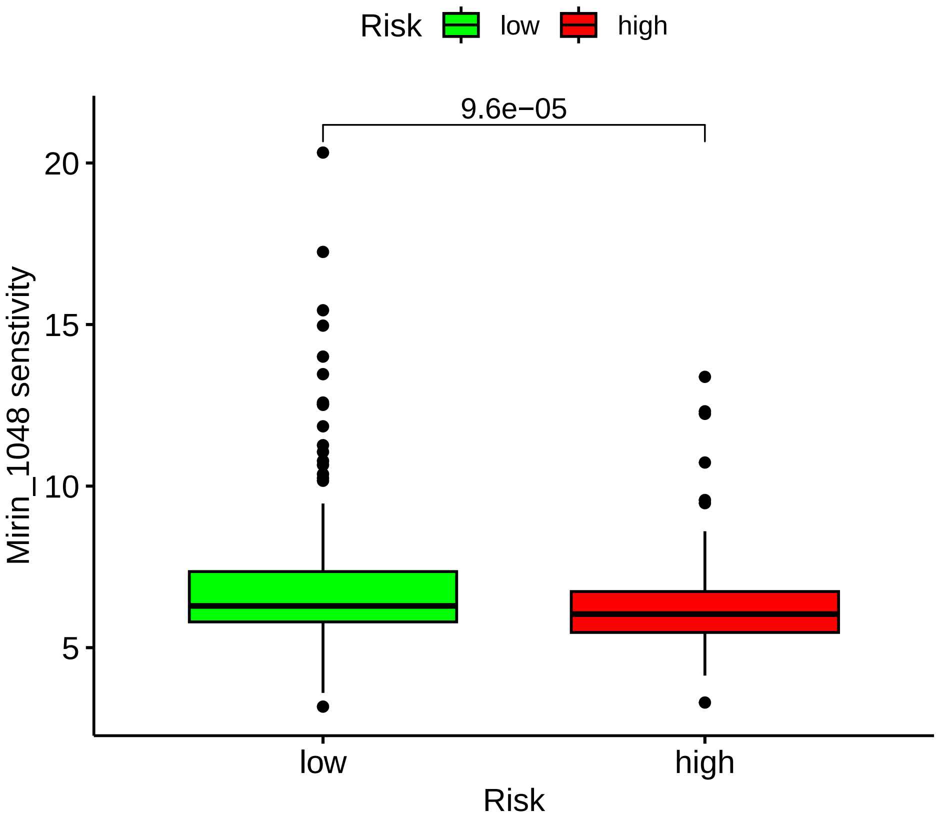

Risk
low
high
9.6e−05
Mirin_1048 senstivity
20
15
10
5
low
high
Risk

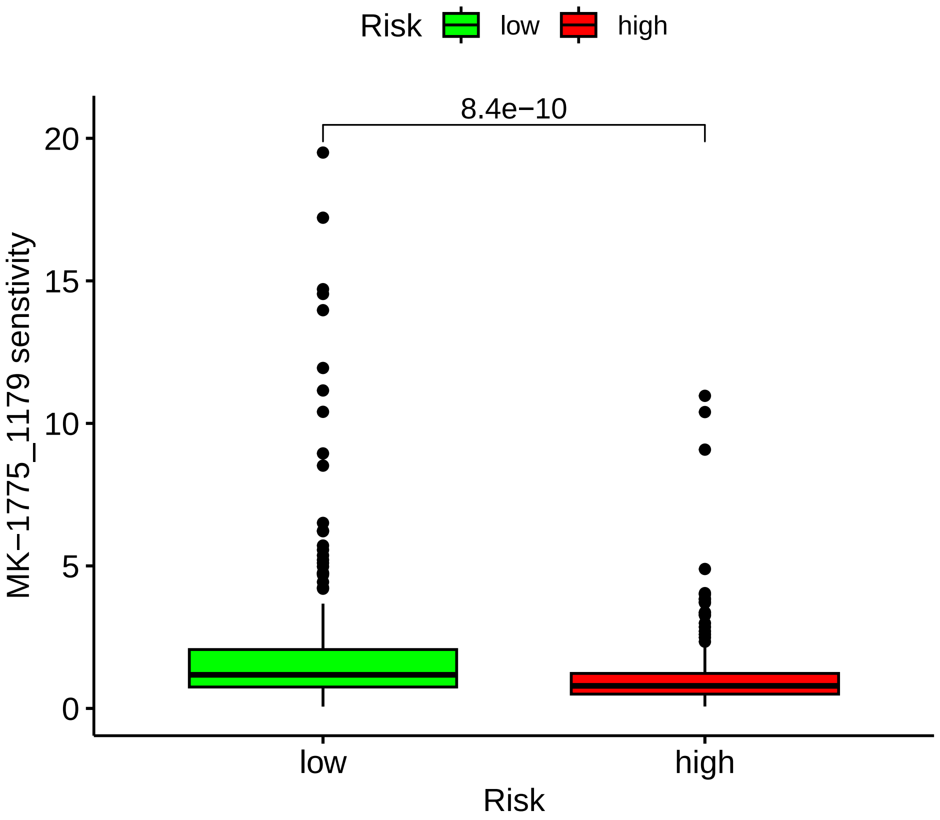
Risk
low
high
8.4e−10
20
15
10
5
0
MK−1775_1179 senstivity
low
high
Risk

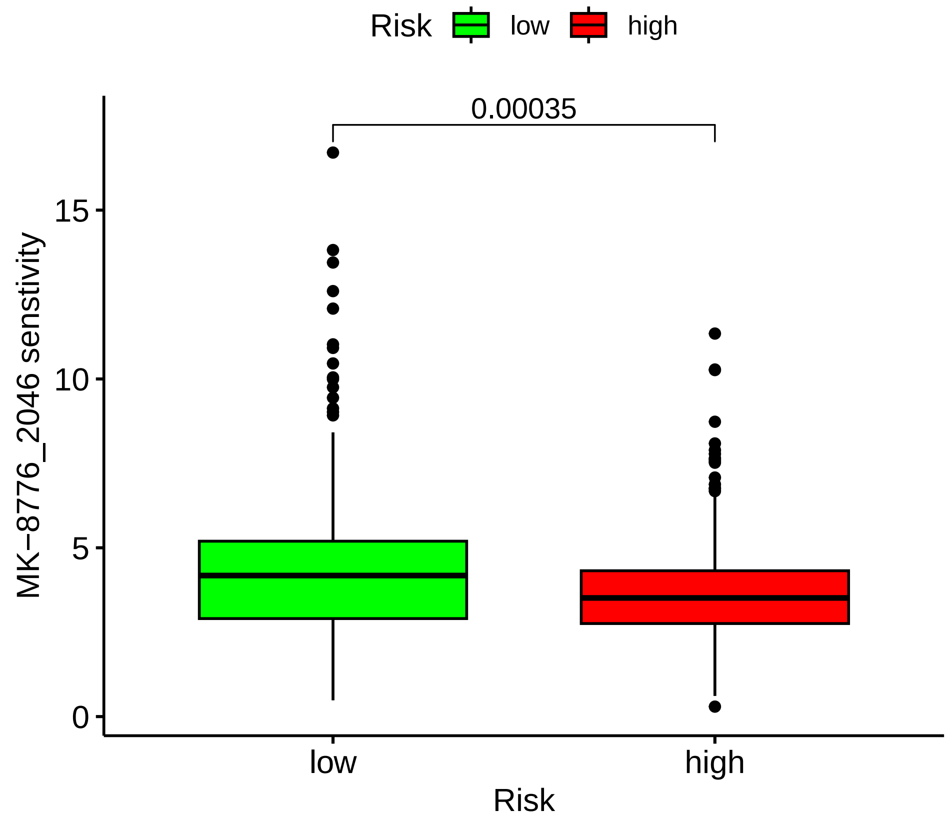
Risk
low
high
0.00035
MK−8776_2046 senstivity
15
10
5
0
low
high
Risk

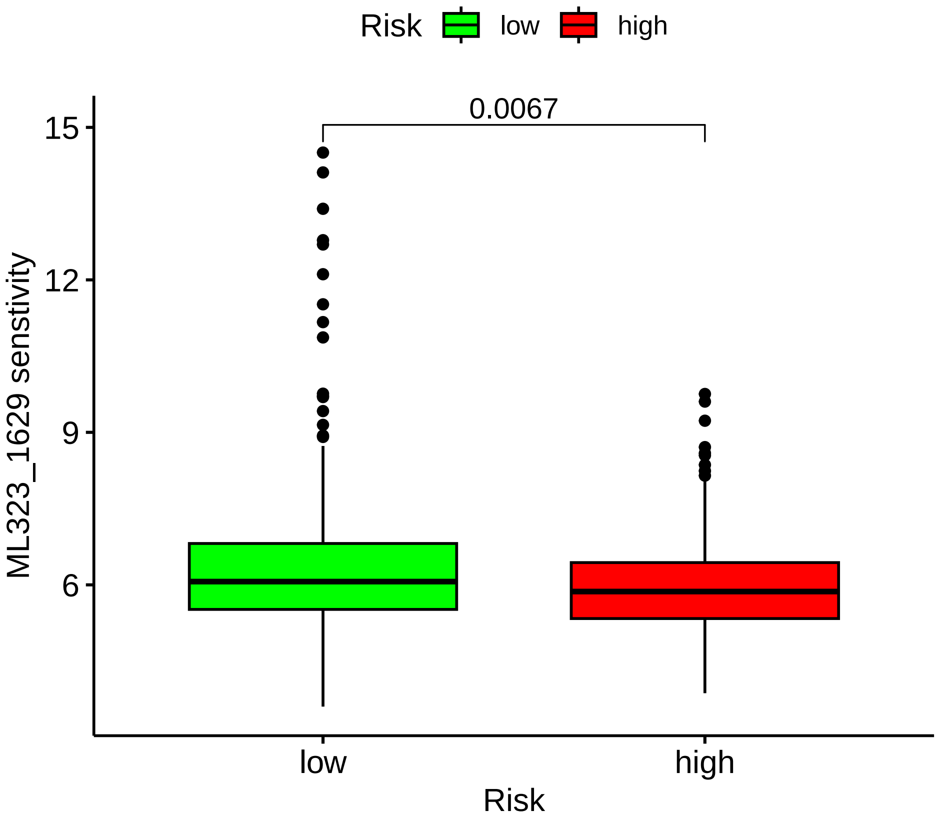
Risk
low
high
0.0067
15
12
9
6
ML323_1629 senstivity
low
high
Risk

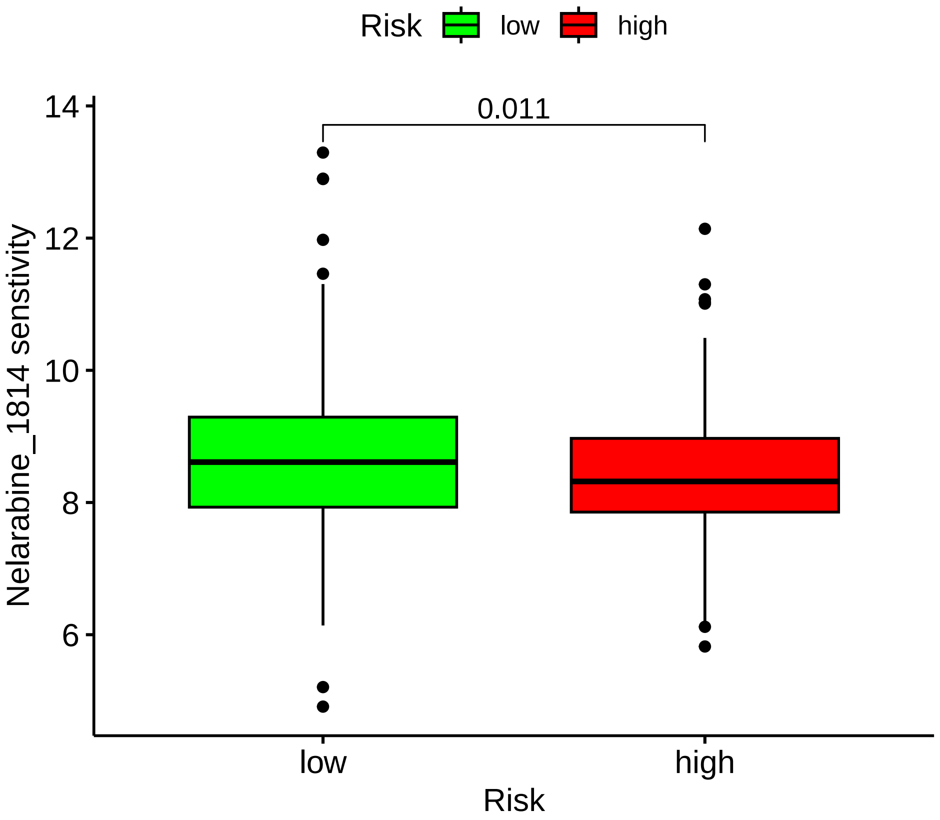

Risk
low
high
0.011
14
12
10
8
6
Nelarabine_1814 senstivity
low
high
Risk

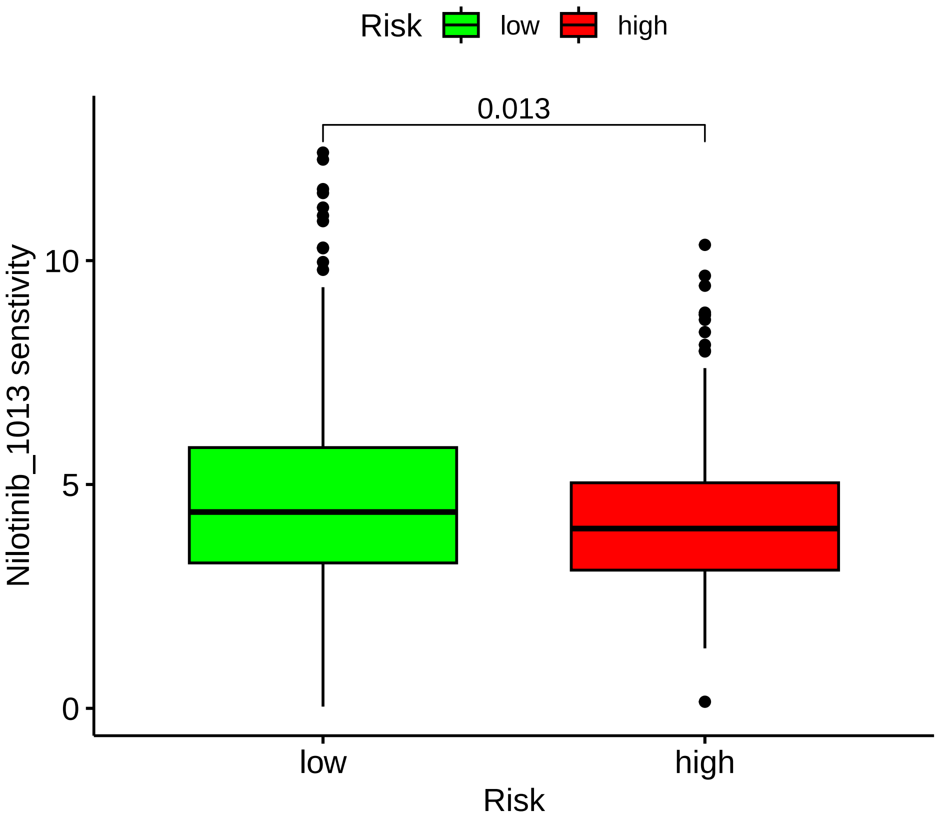

Risk
low
high
0.013
Nilotinib_1013 senstivity
10
5
0
low
high
Risk

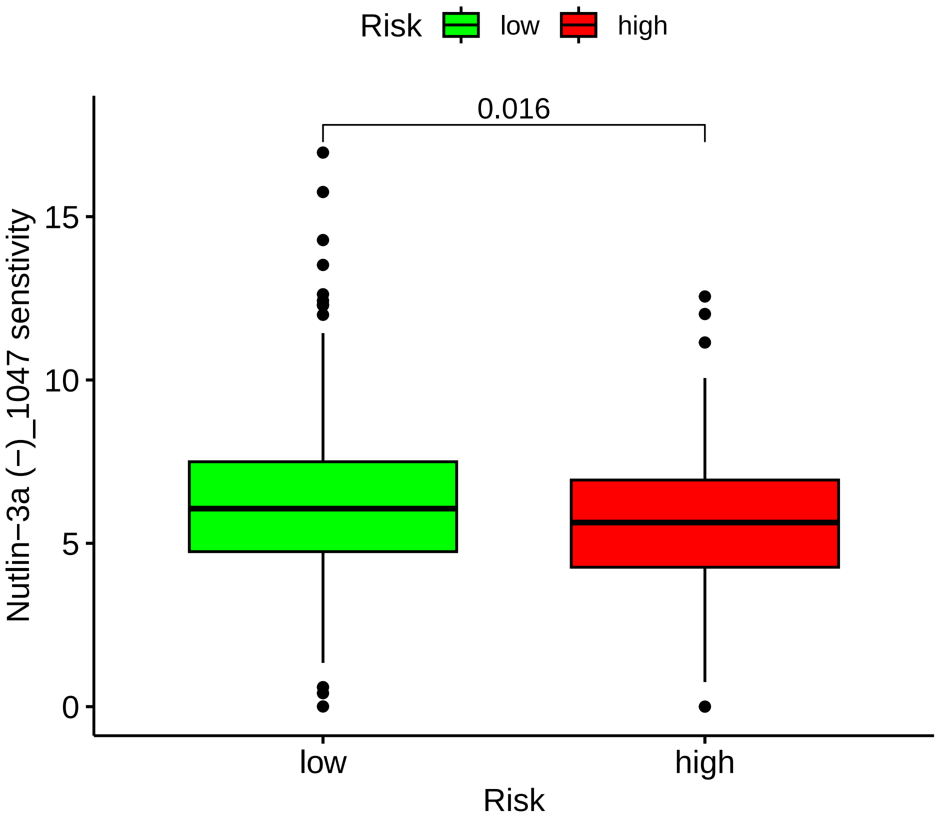

Risk
low
high
0.016
Nutlin−3a (−)_1047 senstivity
15
10
5
0
low
high
Risk

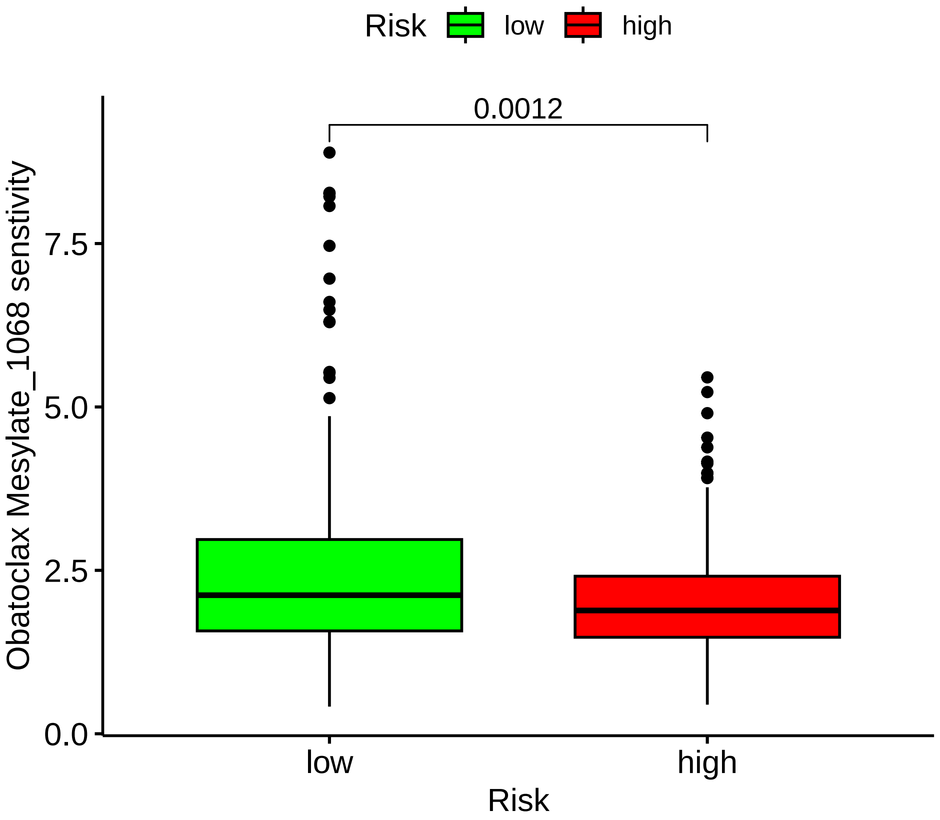
Risk
low
high
0.0012
Obatoclax Mesylate_1068 senstivity
7.5
5.0
2.5
0.0
low
high
Risk

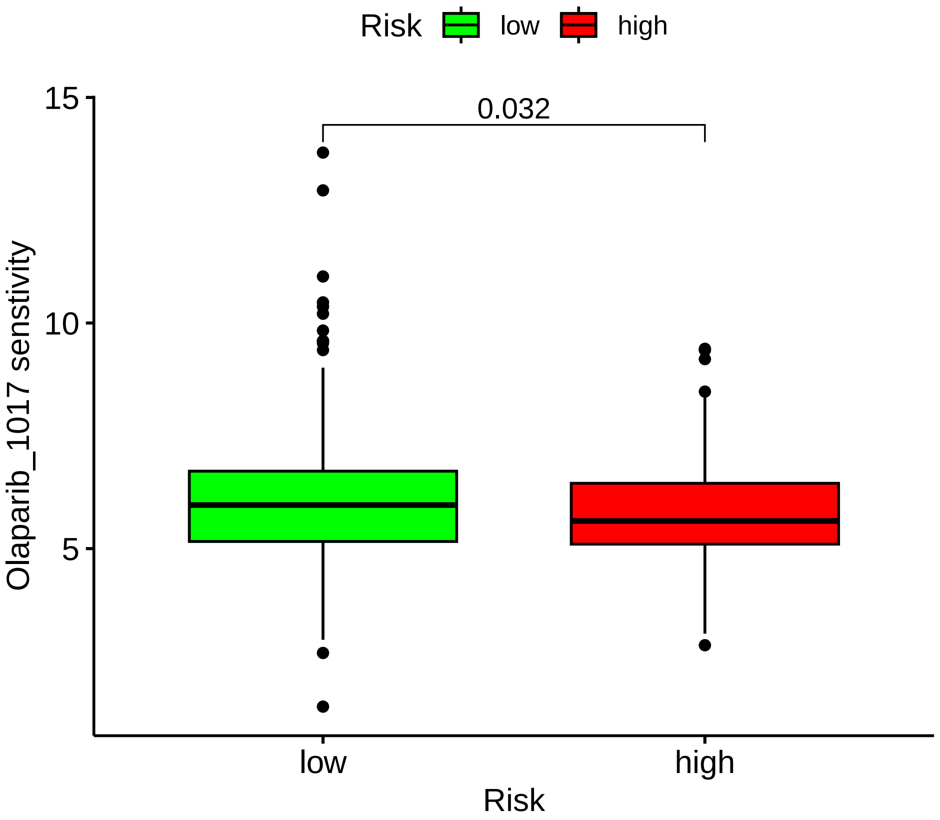
Risk
low
high
15
10
5
0.032
Olaparib_1017 senstivity
low
high
Risk

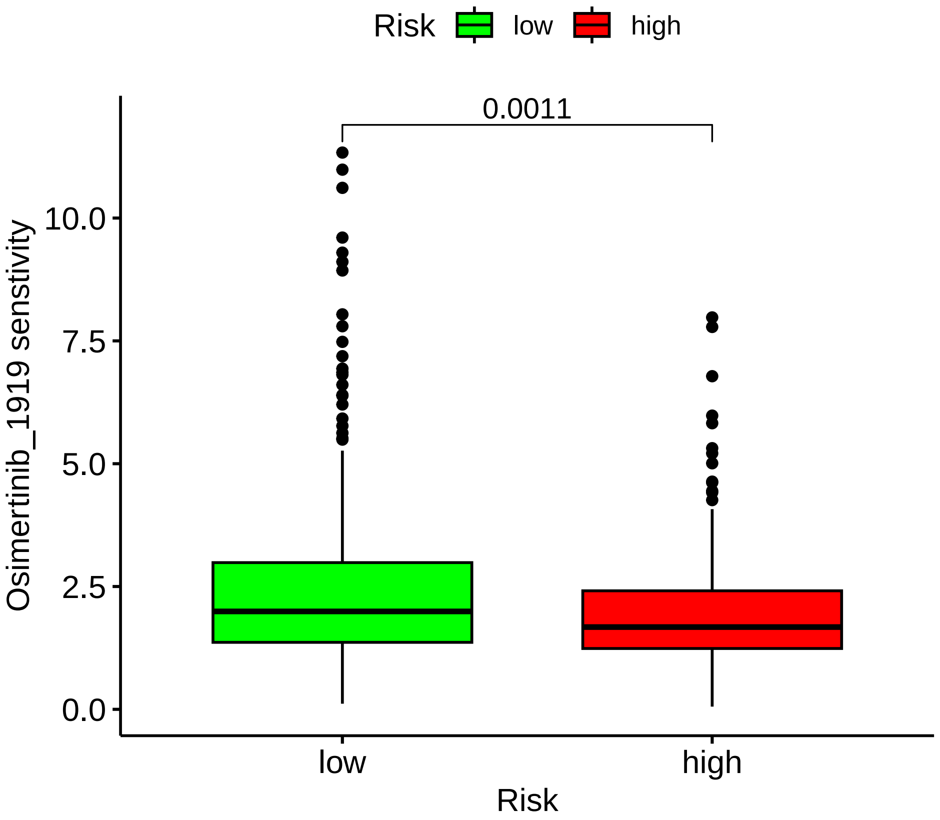

Risk
low
high
0.0011
Osimertinib_1919 senstivity
10.0
7.5
5.0
2.5
0.0
low
high
Risk

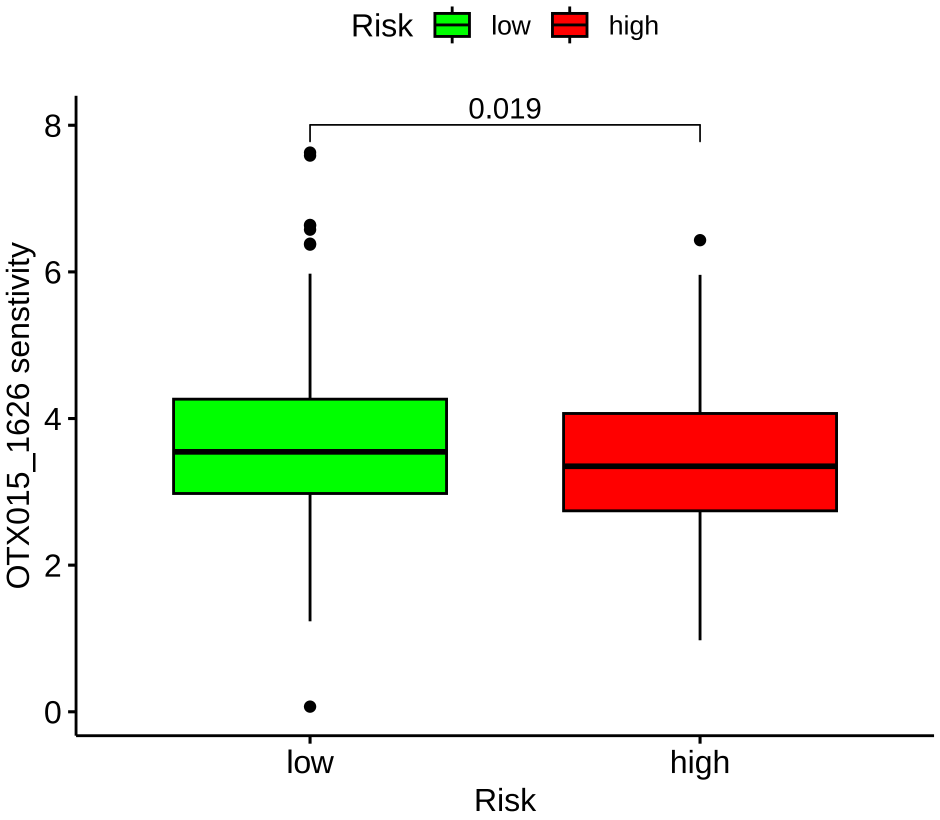
Risk
low
high
0.019
OTX015_1626 senstivity
8
6
4
2
0
low
high
Risk

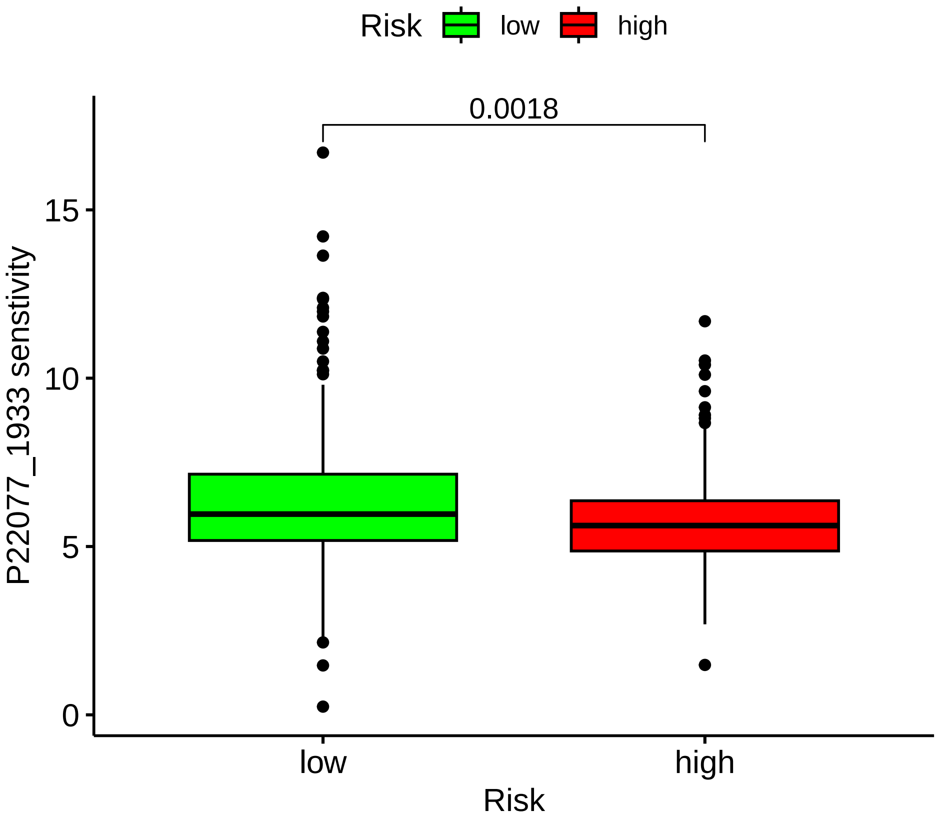

Risk
low
high
0.0018
P22077_1933 senstivity
15
10
5
0
low
high
Risk

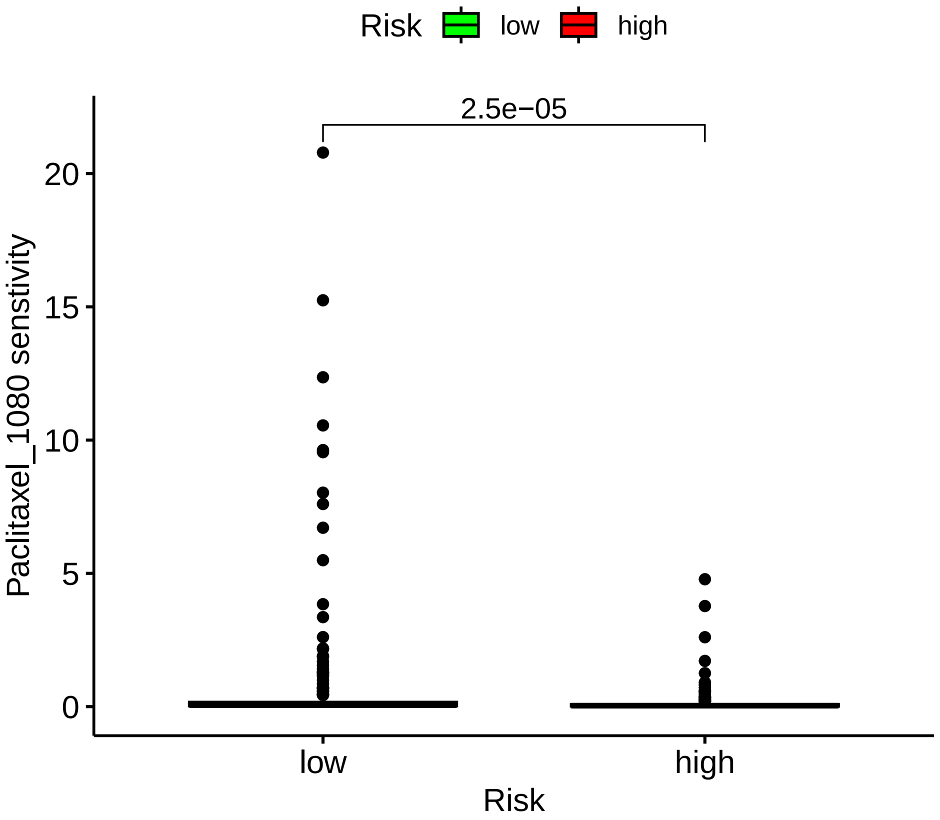
Risk
low
high
2.5e−05
Paclitaxel_1080 senstivity
20
15
10
5
0
low
high
Risk

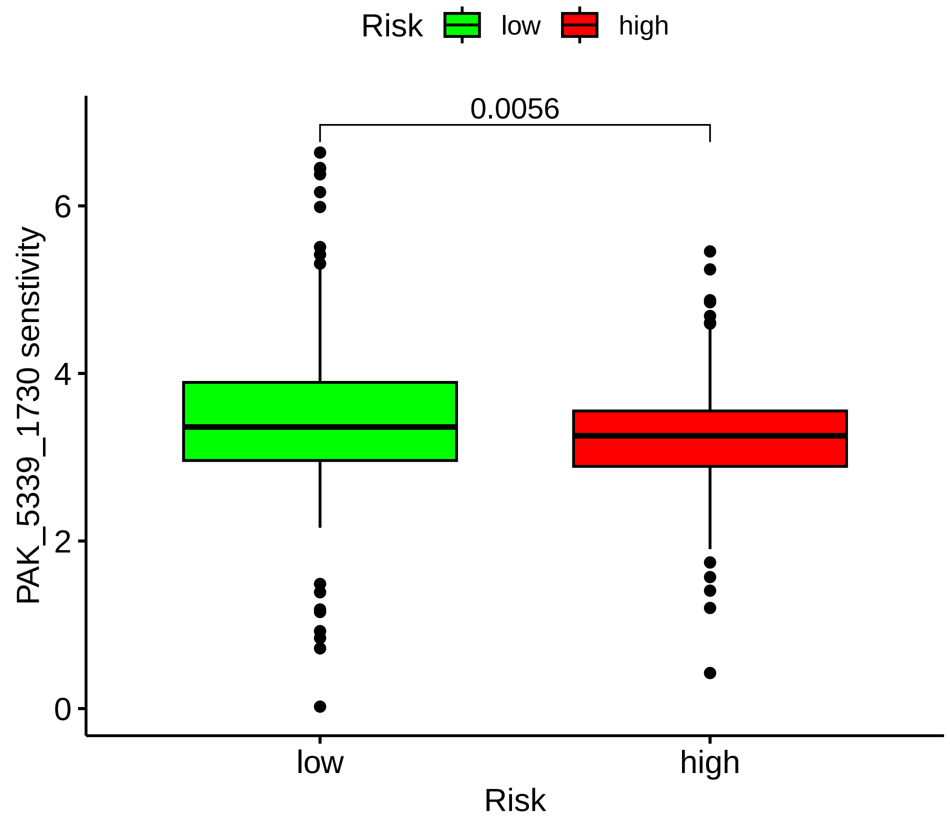

Risk
low
high
0.0056
PAK_5339_1730 senstivity
0
2
4
6
low
high
Risk

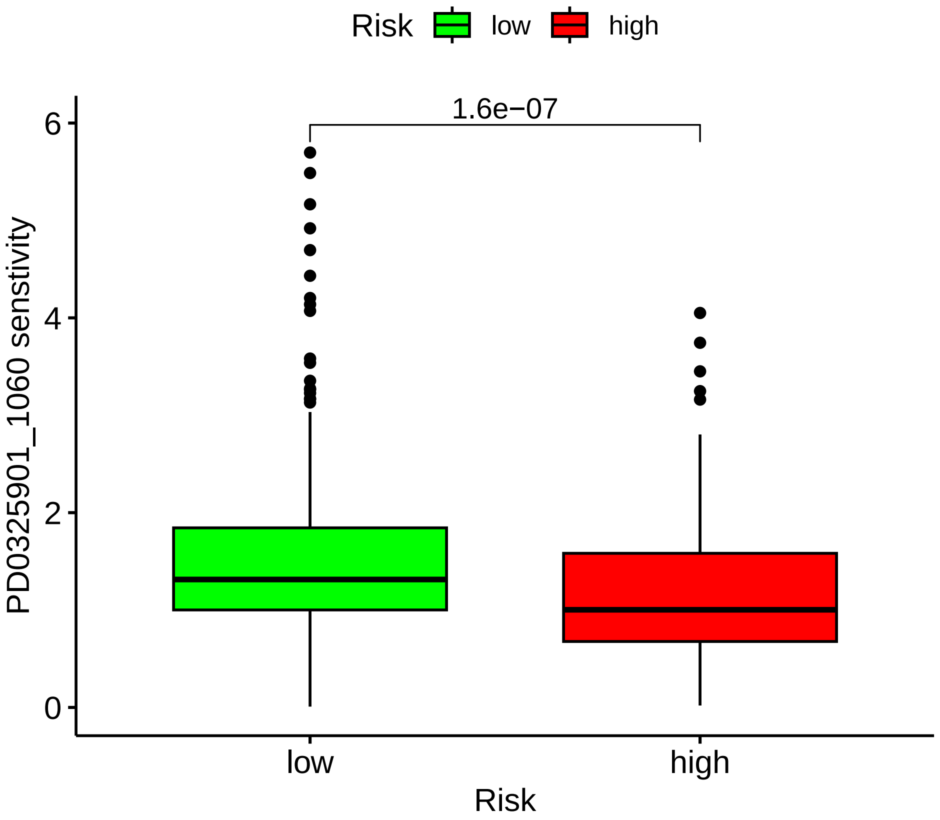
Risk
low
high
1.6e−07
PD0325901_1060 senstivity
6
4
2
0
low
high
Risk

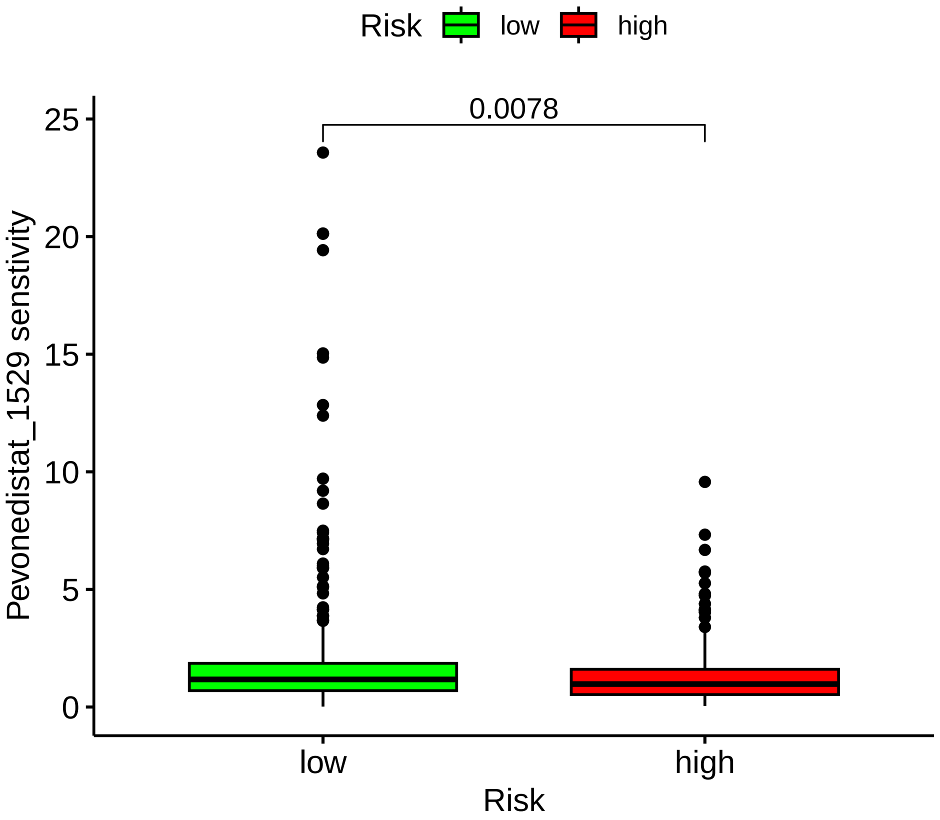

Risk
low
high
0.0078
Pevonedistat_1529 senstivity
25
20
15
10
5
0
low
high
Risk

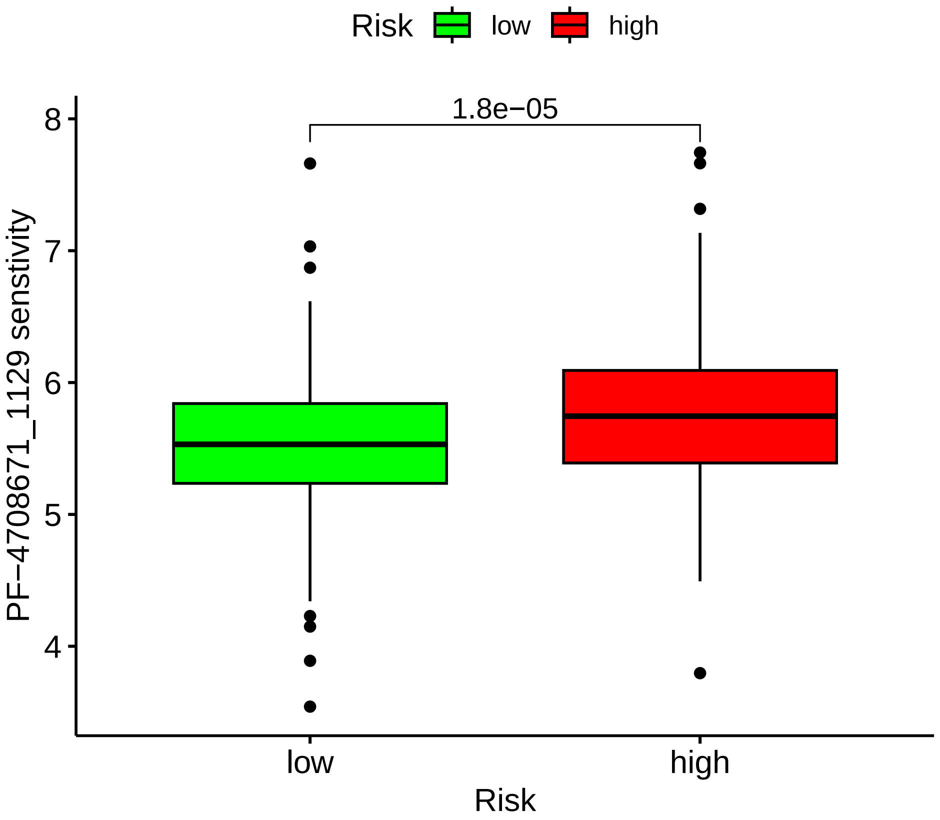
Risk
low
high
1.8e−05
8
7
6
5
4
PF−4708671_1129 senstivity
low
high
Risk

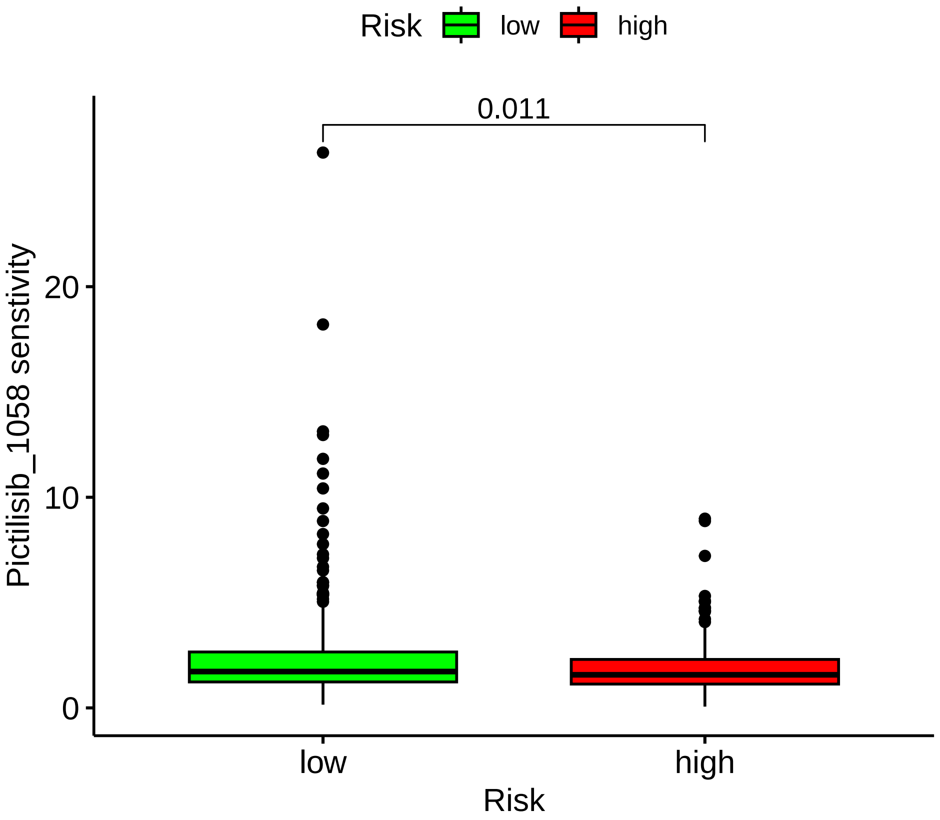

Risk
low
high
0.011
Pictilisib_1058 senstivity
20
10
0
low
high
Risk

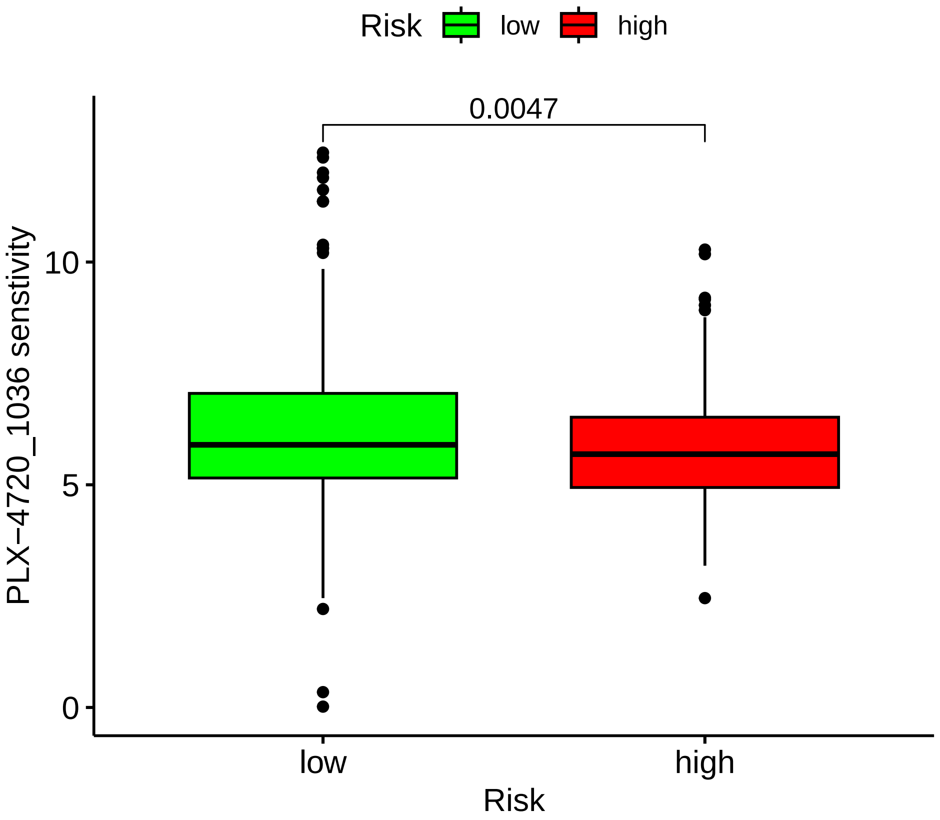
Risk
low
high
0.0047
PLX−4720_1036 senstivity
10
5
0
low
high
Risk

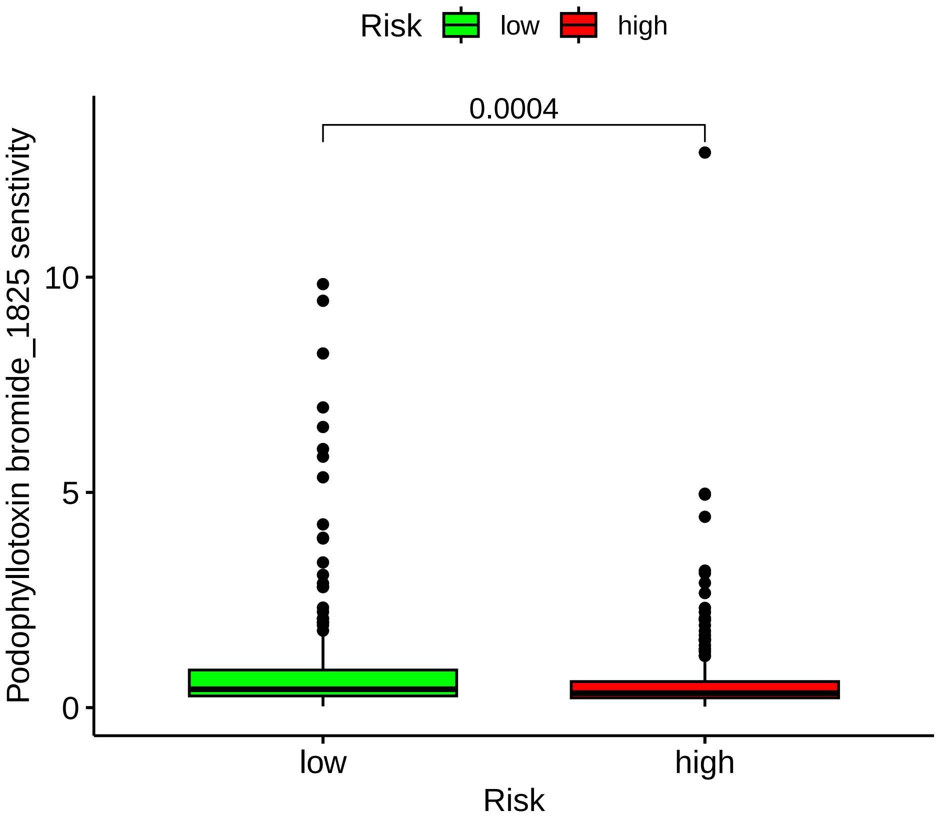

Risk
low
high
0.0004
Podophyllotoxin bromide_1825 senstivity
10
5
0
low
high
Risk

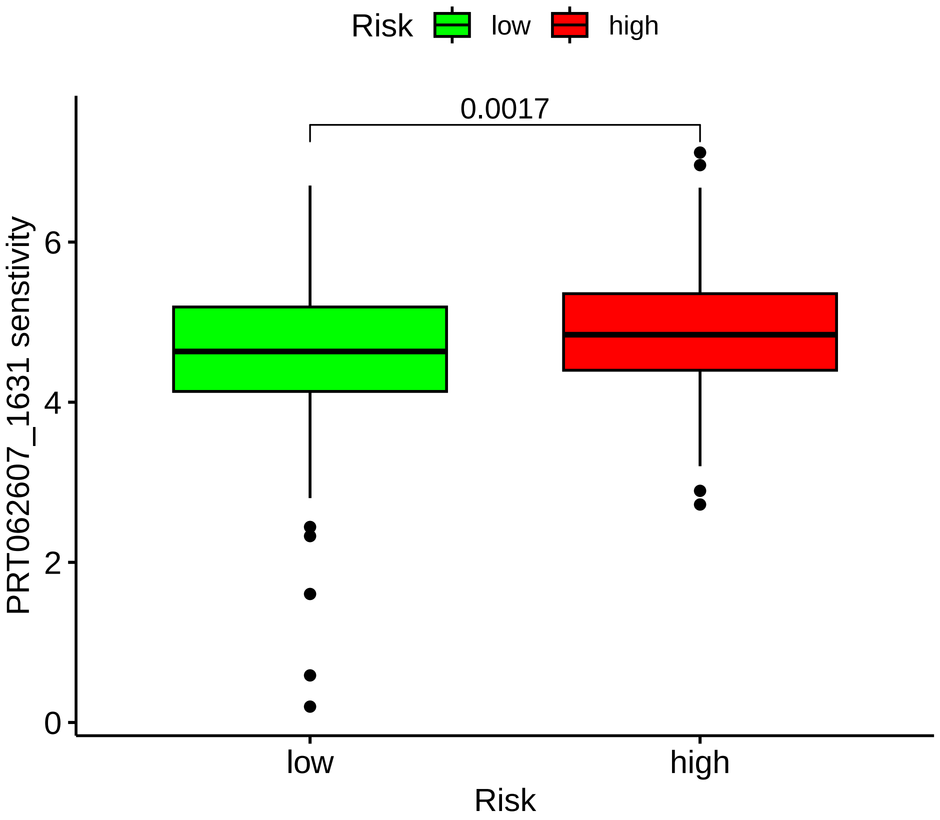
Risk
low
high
0.0017
PRT062607_1631 senstivity
6
4
2
0
low
high
Risk

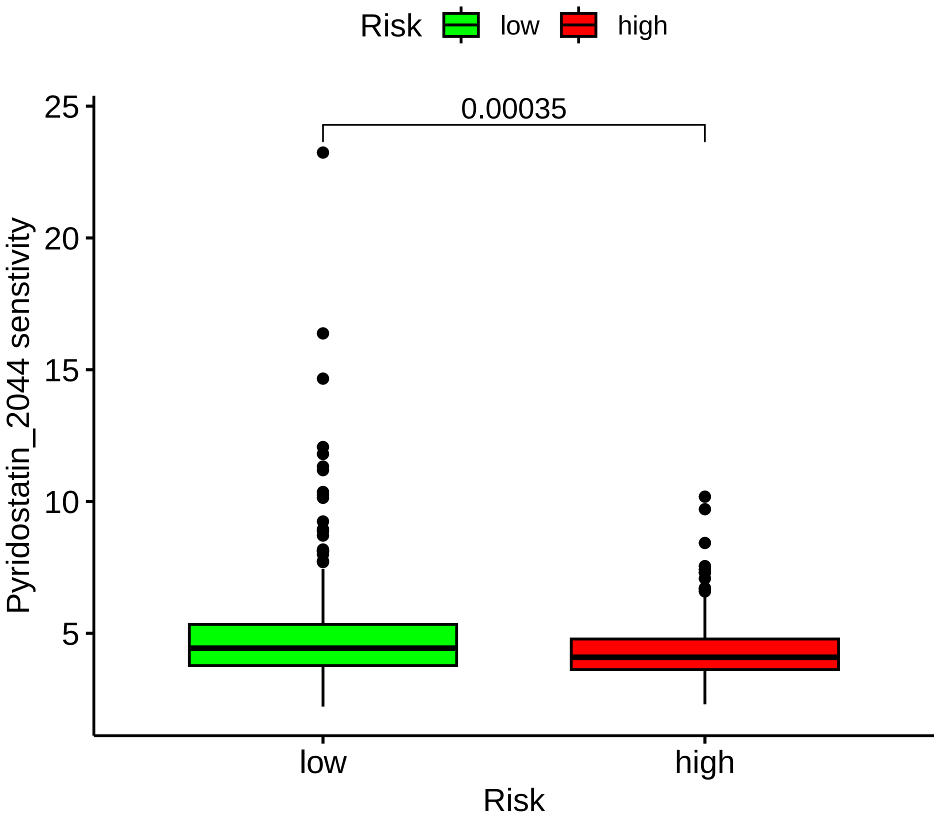

Risk
low
high
0.00035
25
20
15
10
5
Pyridostatin_2044 senstivity
low
high
Risk

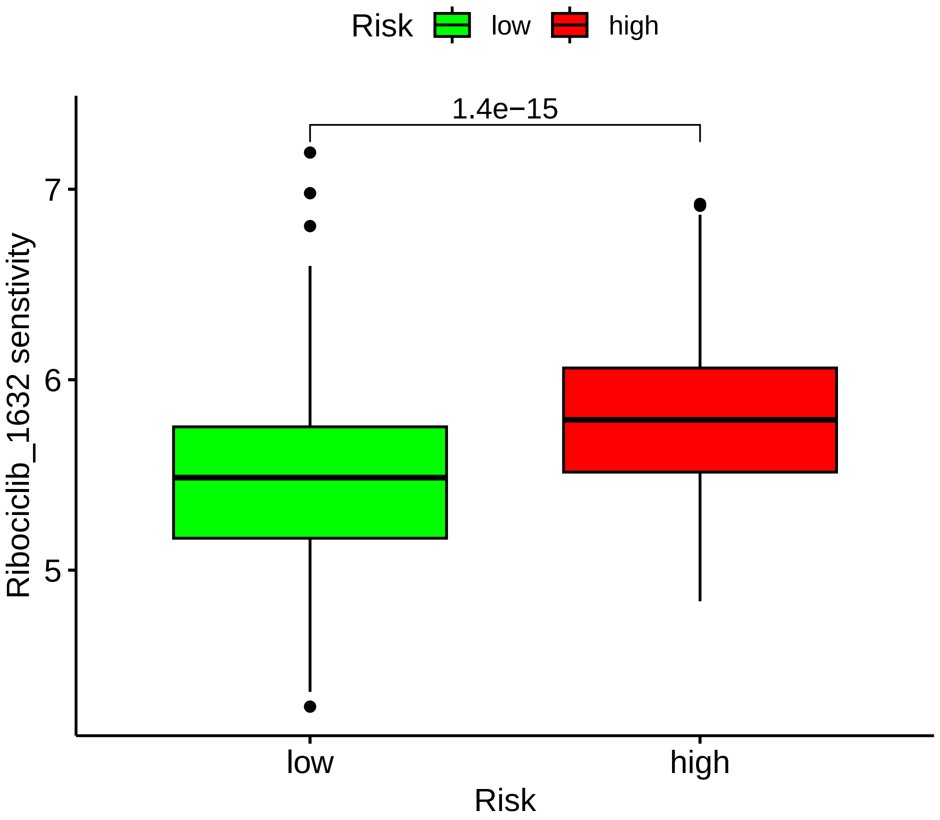

Risk
low
high
1.4e−15
Ribociclib_1632 senstivity
7
6
5
low
high
Risk

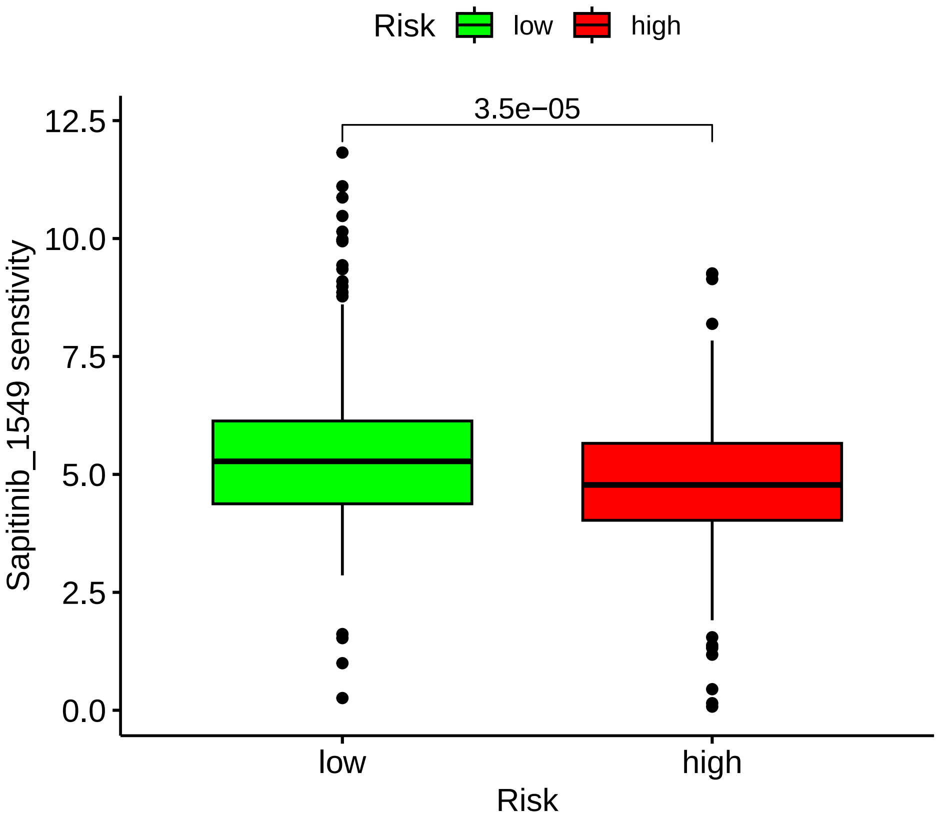

Risk
low
high
3.5e−05
12.5
10.0
7.5
5.0
2.5
0.0
Sapitinib_1549 senstivity
low
high
Risk

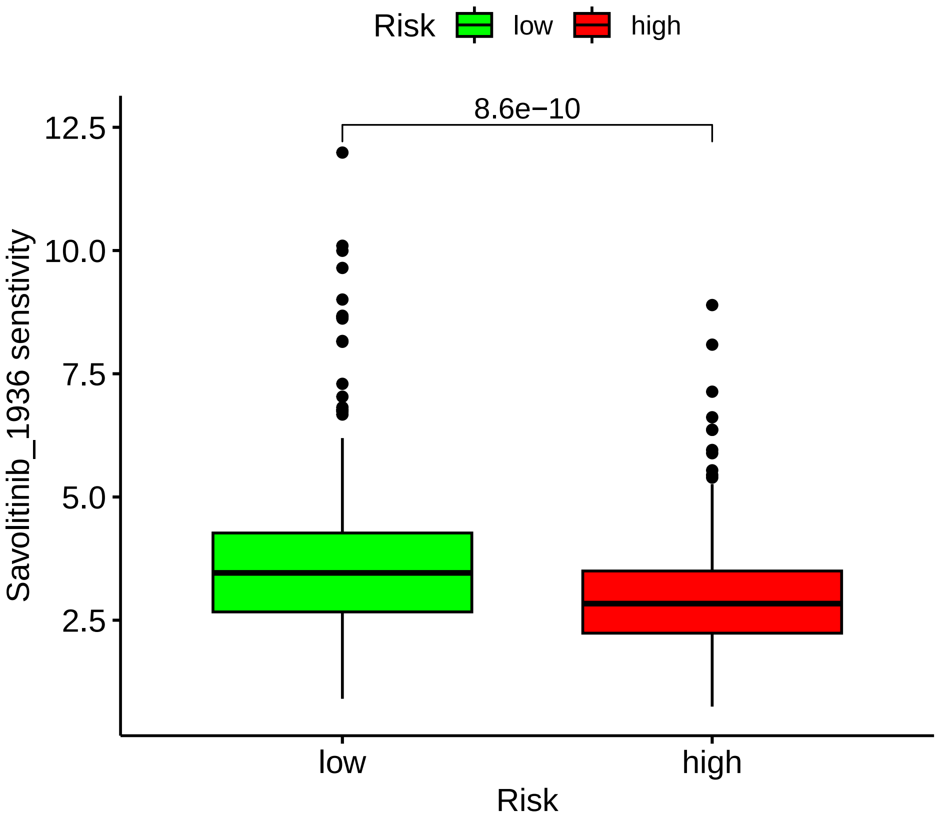

Risk
low
high
8.6e−10
Savolitinib_1936 senstivity
12.5
10.0
7.5
5.0
2.5
low
high
Risk

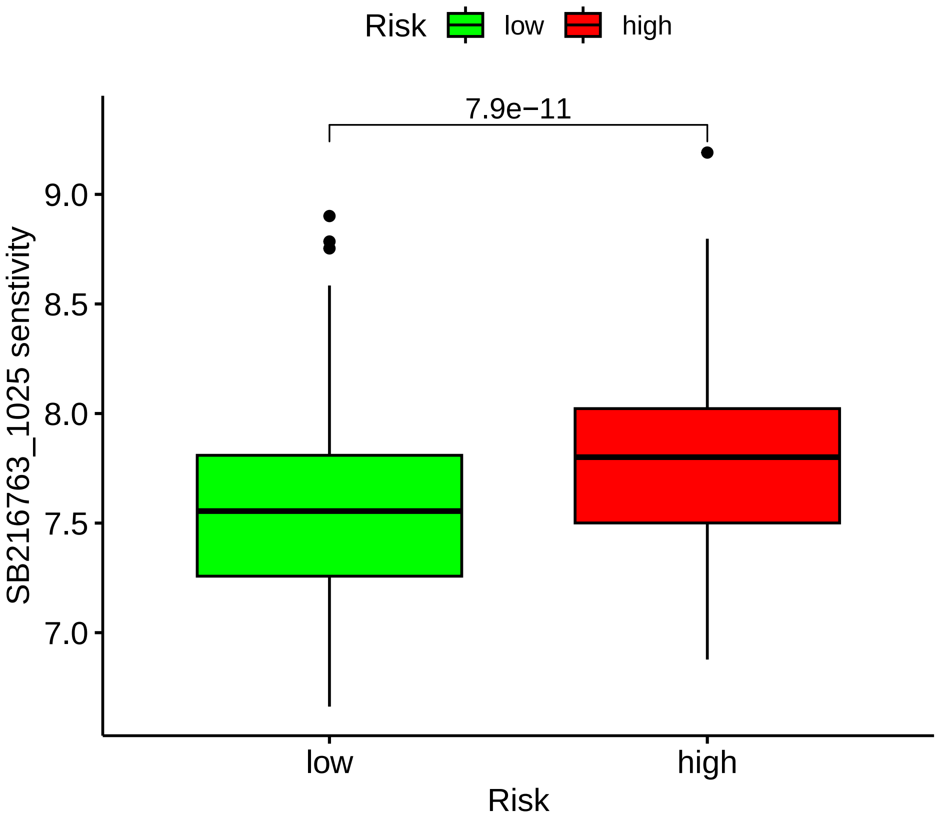

Risk
low
high
7.9e−11
9.0
8.5
8.0
7.5
7.0
SB2167763_1025 senstivity
low
high
Risk

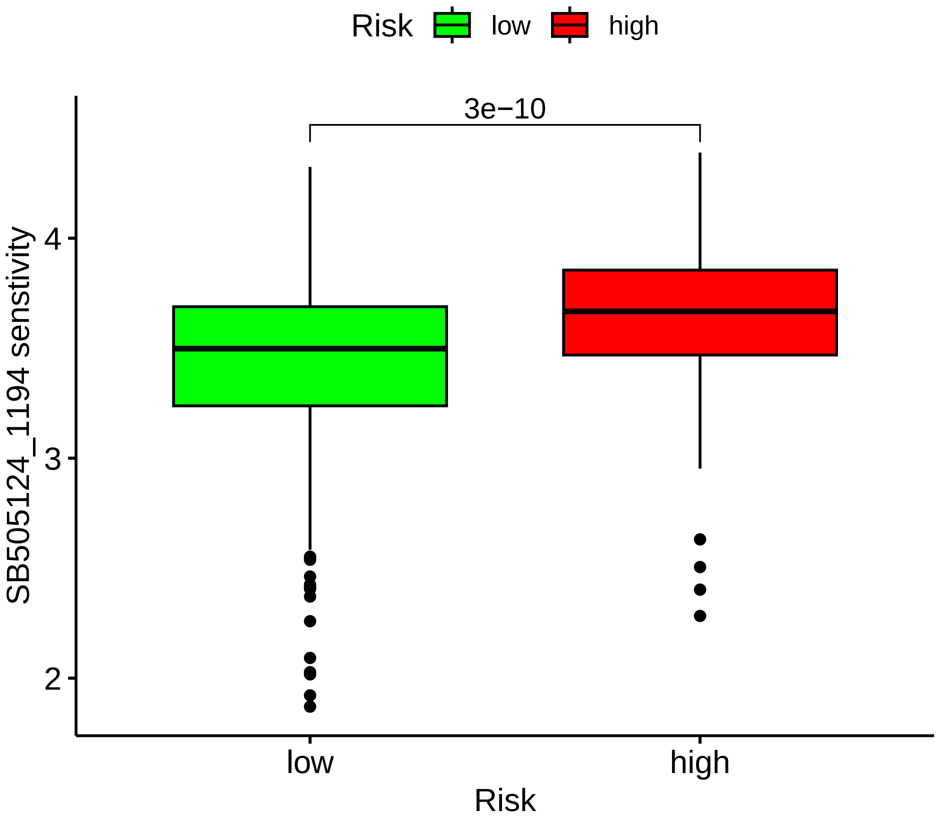

Risk
low
high
3e−10
SB505124_1194 senstivity
4
3
2
low
high
Risk

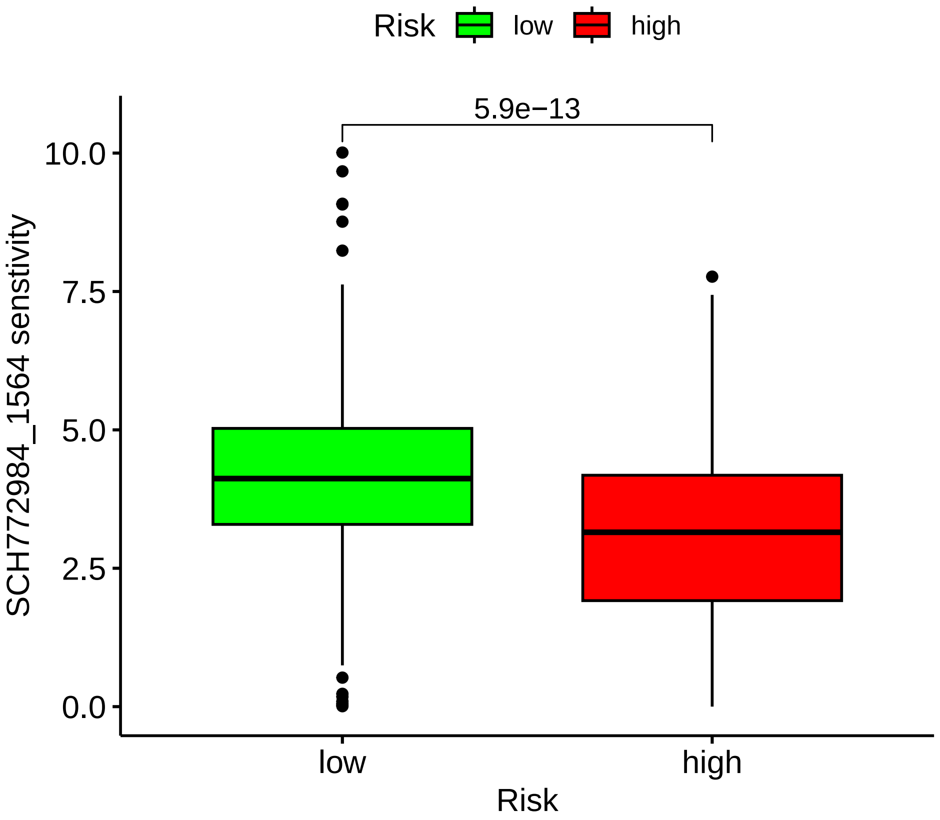
Risk
low
high
5.9e−13
SCH772984_1564 senstivity
10.0
7.5
5.0
2.5
0.0
low
high
Risk

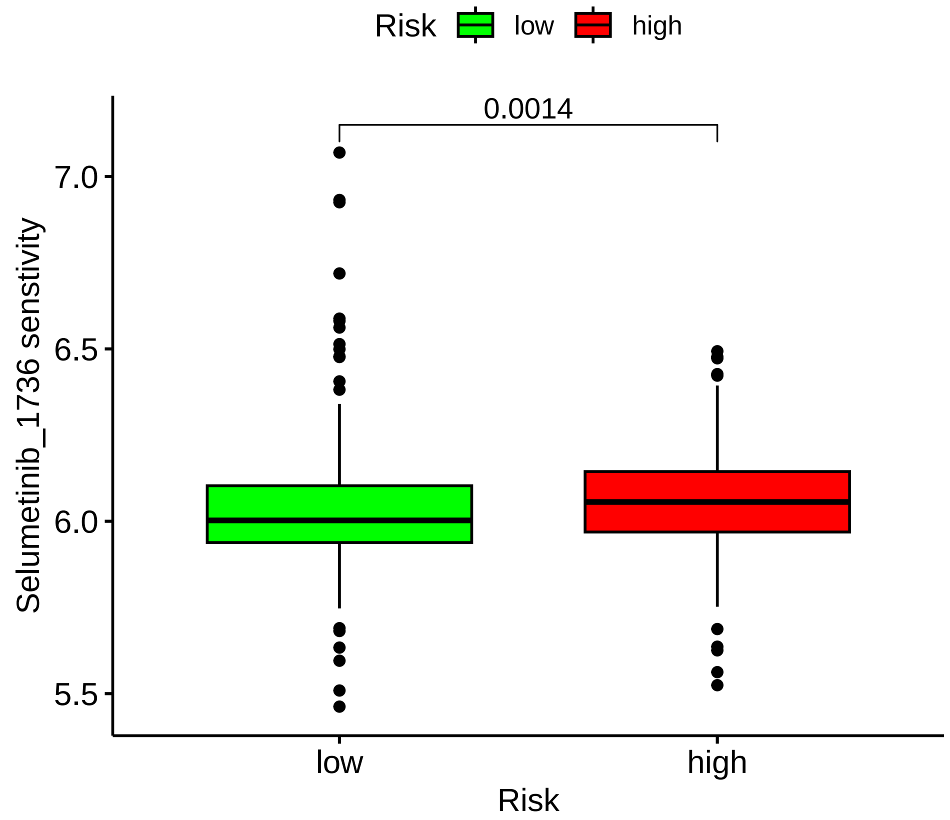

Risk
low
high
0.0014
7.0
6.5
6.0
5.5
Selumetinib_1736 senstivity
low
high
Risk

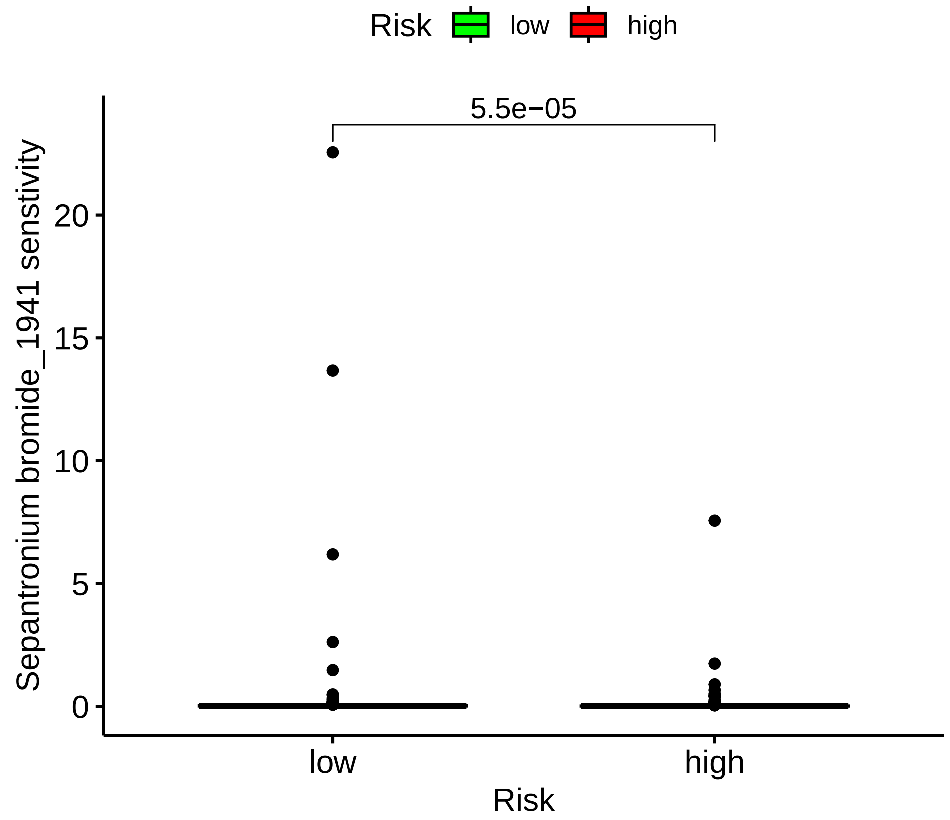
Risk
low
high
5.5e−05
Sepantronium bromide_1941 senstivity
20
15
10
5
0
low
high
Risk

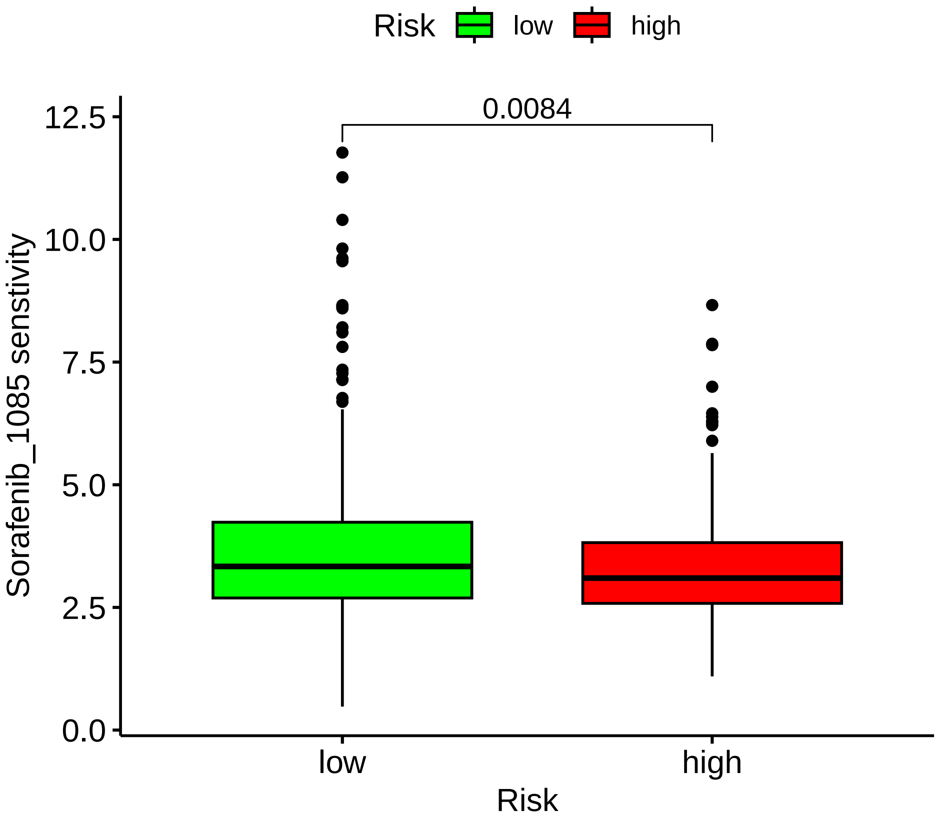
Risk
low
high
0.0084
12.5
10.0
7.5
5.0
2.5
0.0
Sorafenib_1085 senstivity
low
high
Risk

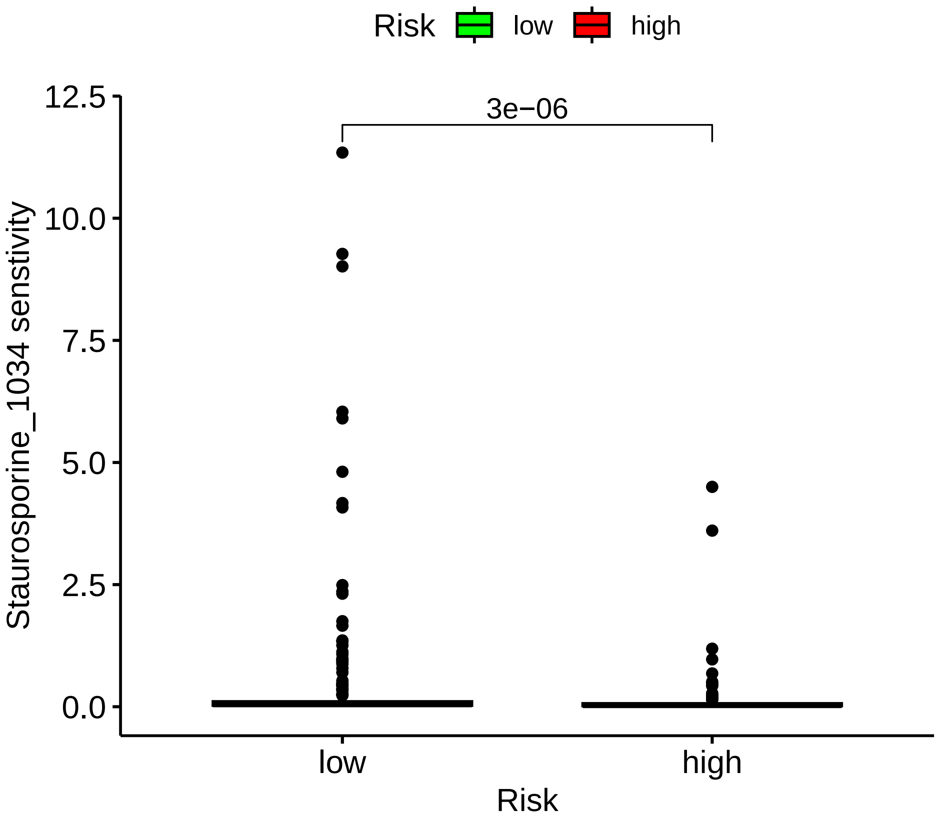

Risk
low
high
12.5
10.0
7.5
5.0
2.5
0.0
3e−06
Staurosporine_1034 senstivity
low
high
Risk

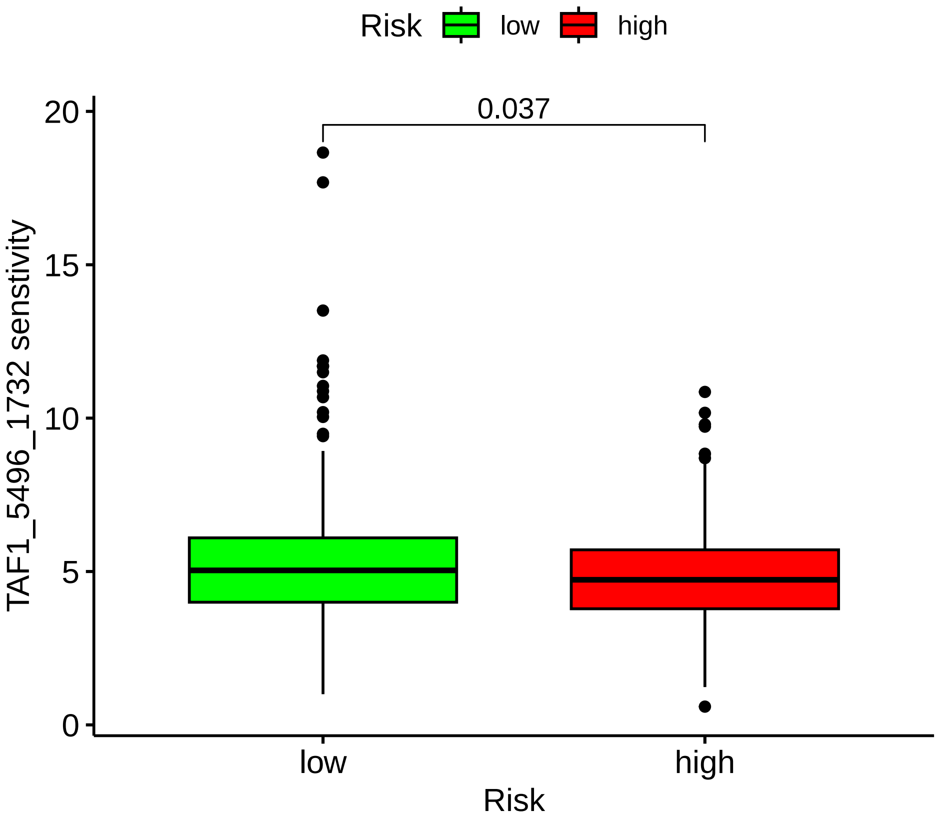

Risk
low
high
0.037
20
15
10
5
0
TAF1_5496_1732 senstivity
low
high
Risk

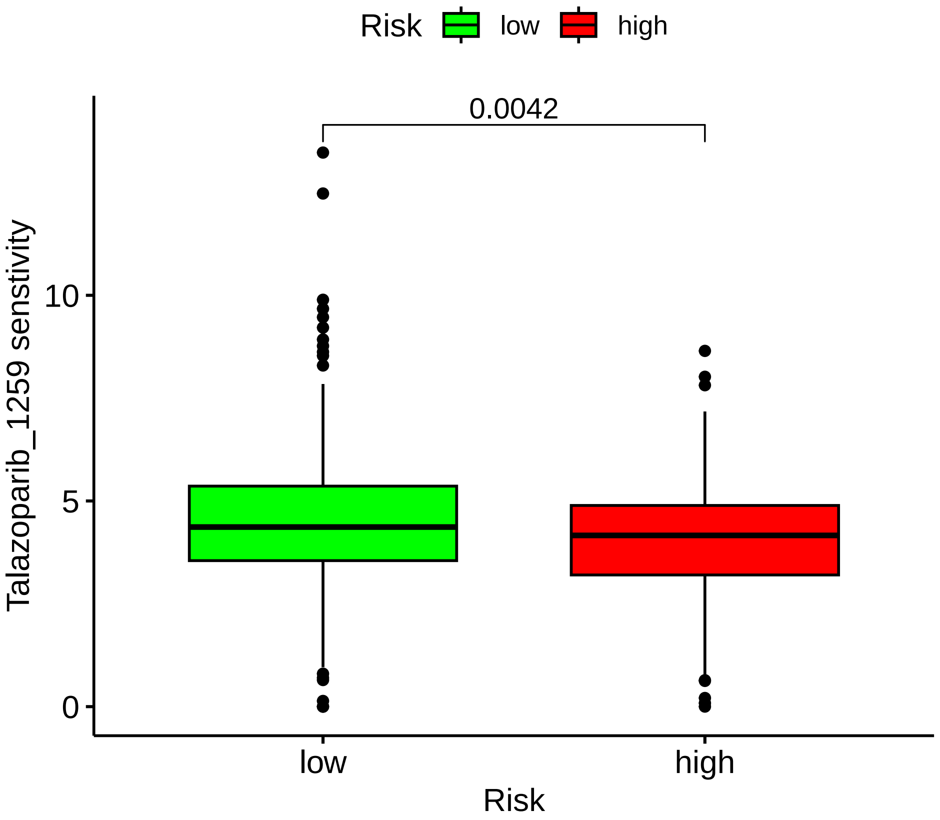
Risk
low
high
0.0042
Talazoparib_1259 senstivity
10
5
0
low
high
Risk

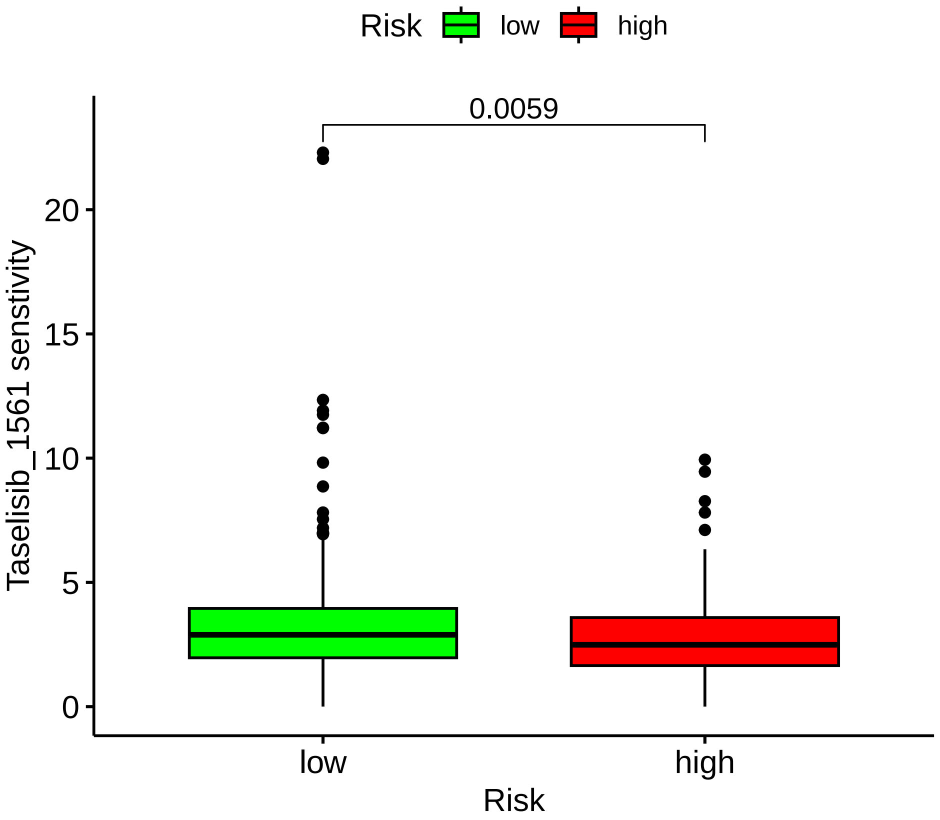
Risk
low
high
0.0059
Taselisib_1561 senstivity
0
5
10
15
20
low
high
Risk

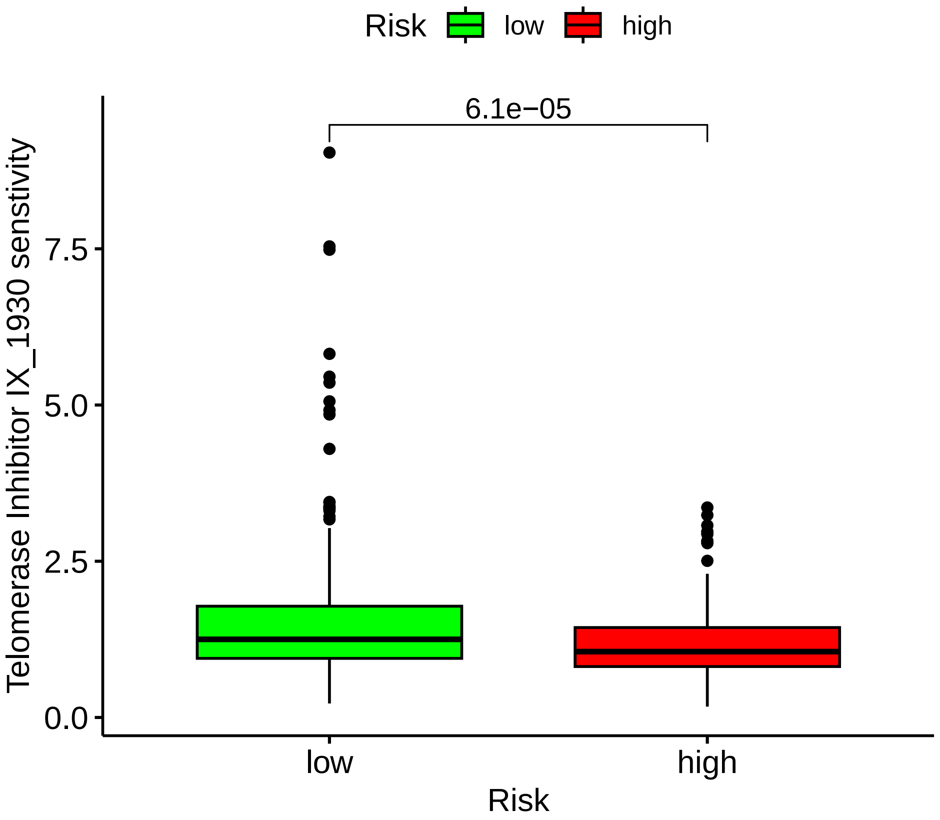
Risk
low
high
6.1e−05
Telomerase Inhibitor IX_1930 senstivity
7.5
5.0
2.5
0.0
low
high
Risk

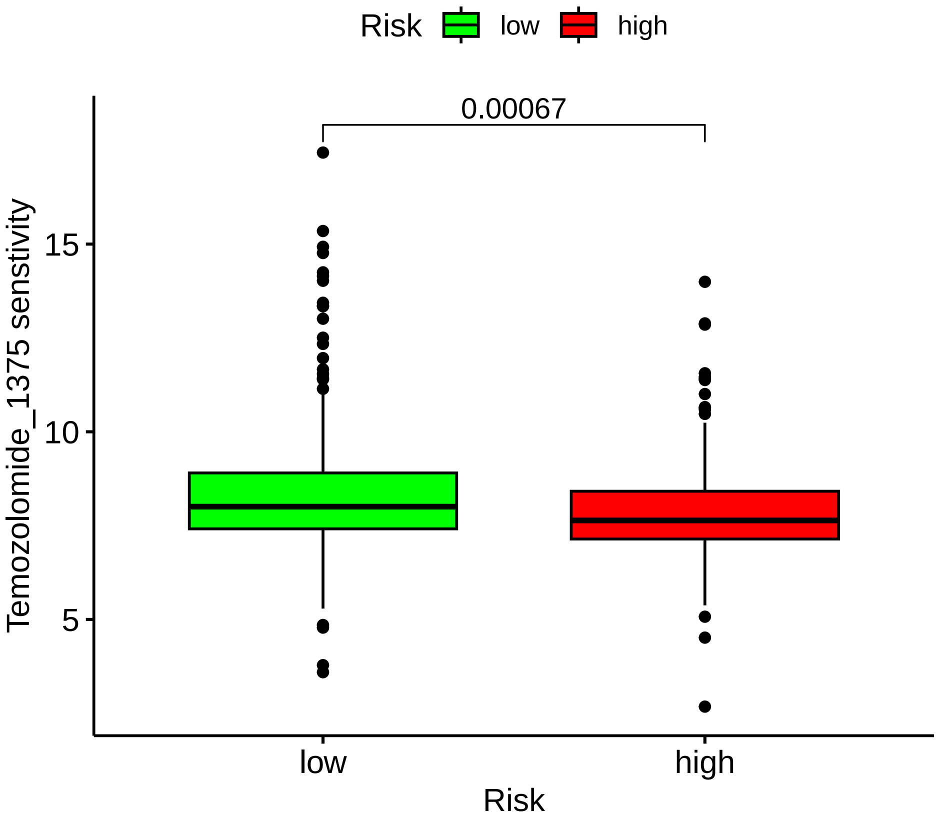

Risk
low
high
0.00067
Temozolomide_1375 senstivity
15
10
5
low
high
Risk

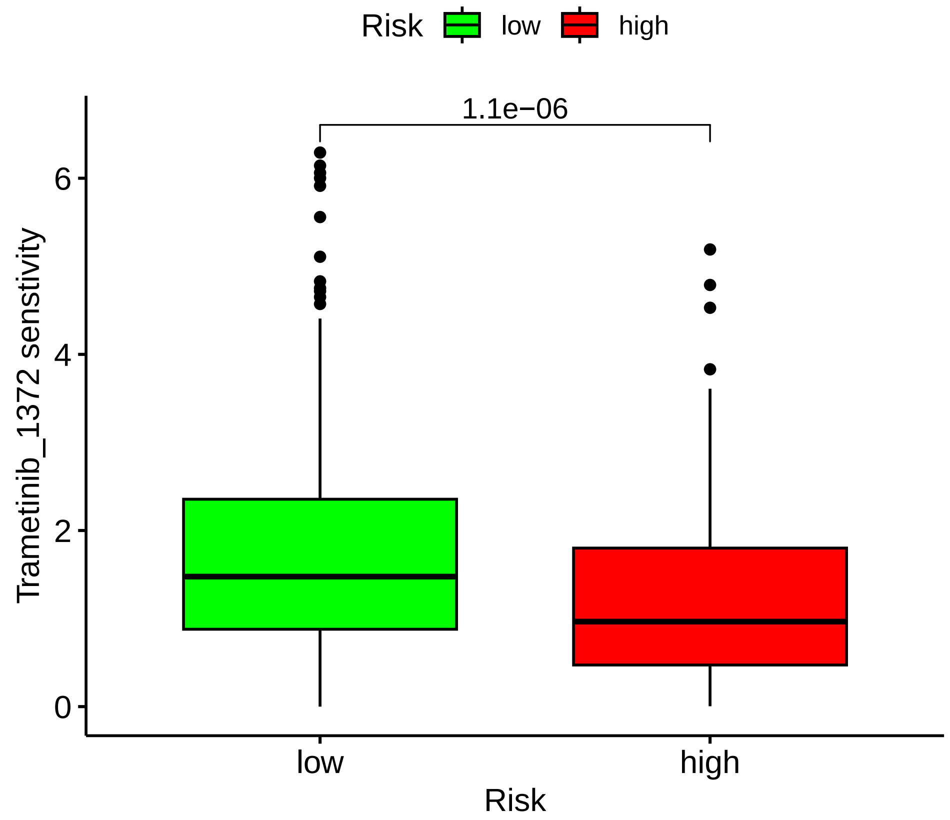

Risk
low
high
1.1e−06
Trametinib_1372 senstivity
6
4
2
0
low
high
Risk

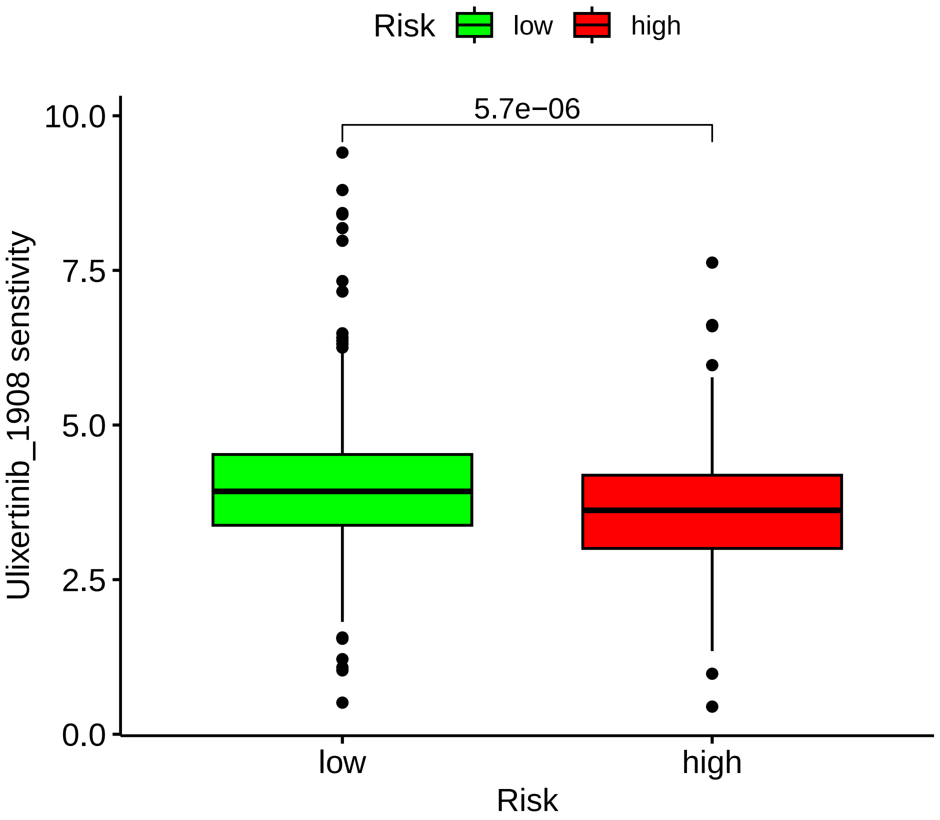

Risk
low
high
5.7e−06
10.0
7.5
5.0
2.5
0.0
Ulixertinib_1908 senstivity
low
high
Risk

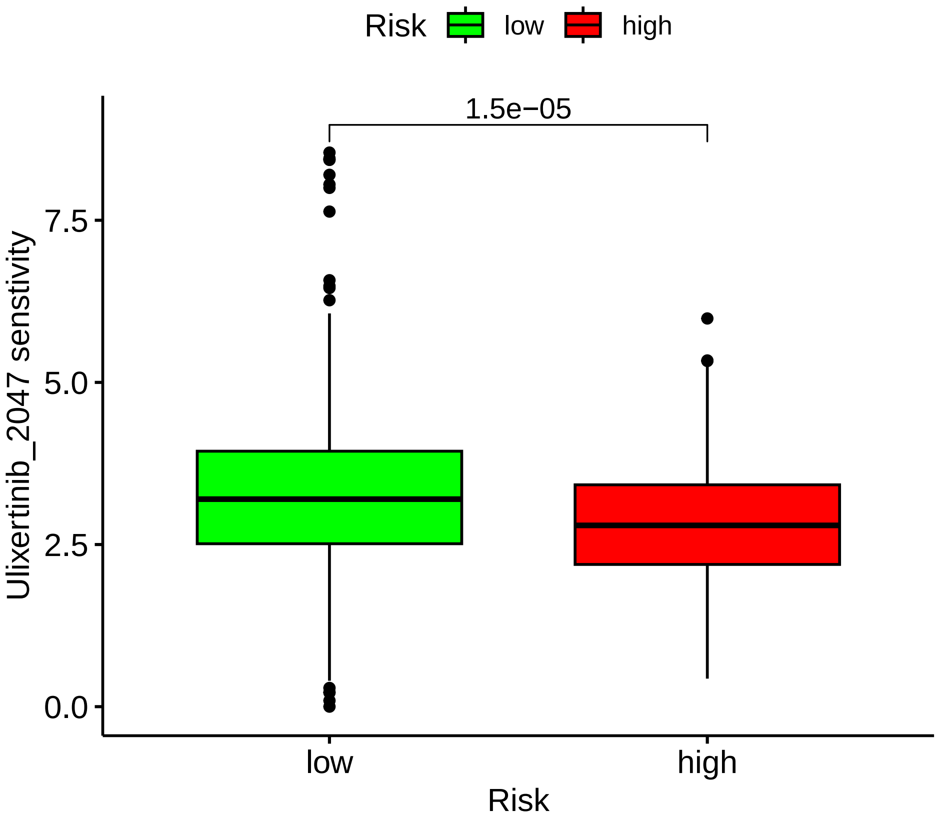

Risk
low
high
1.5e−05
Ulixertinib_2047 senstivity
7.5
5.0
2.5
0.0
low
high
Risk

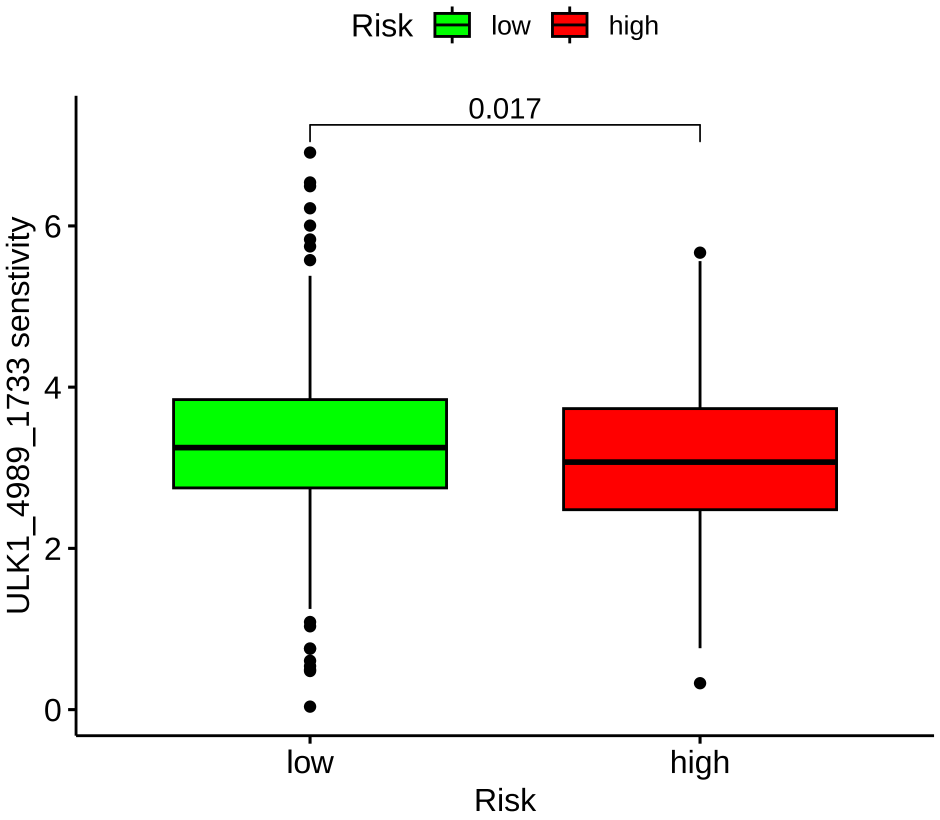

Risk
low
high
0.017
ULK1_4989_1733 senstivity
6
4
2
0
low
high
Risk

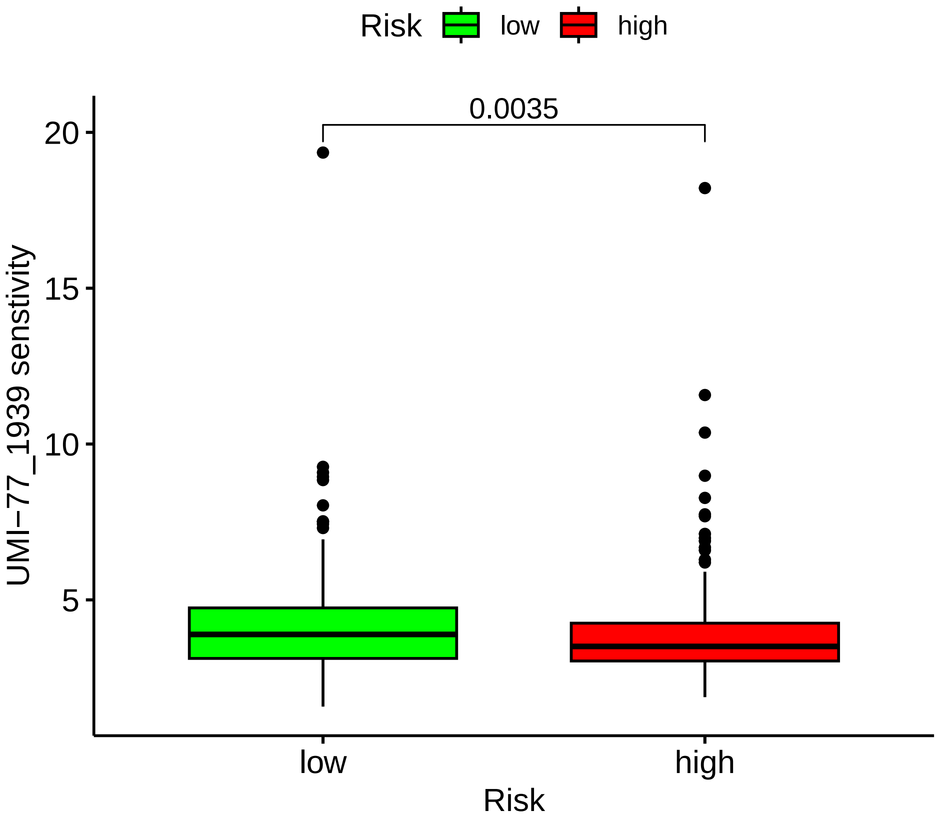

Risk
low
high
0.0035
UMI−77_1939 senstivity
20
15
10
5
low
high
Risk

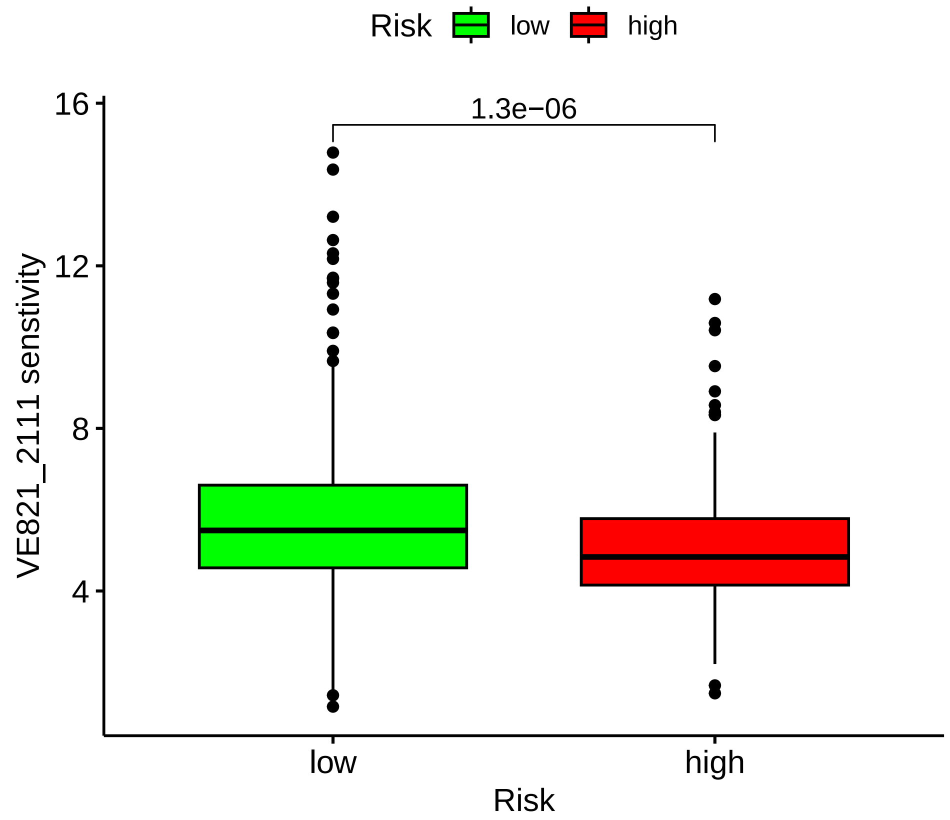

Risk
low
high
1.3e−06
16
12
8
4
VE821_2111 senstivity
low
high
Risk

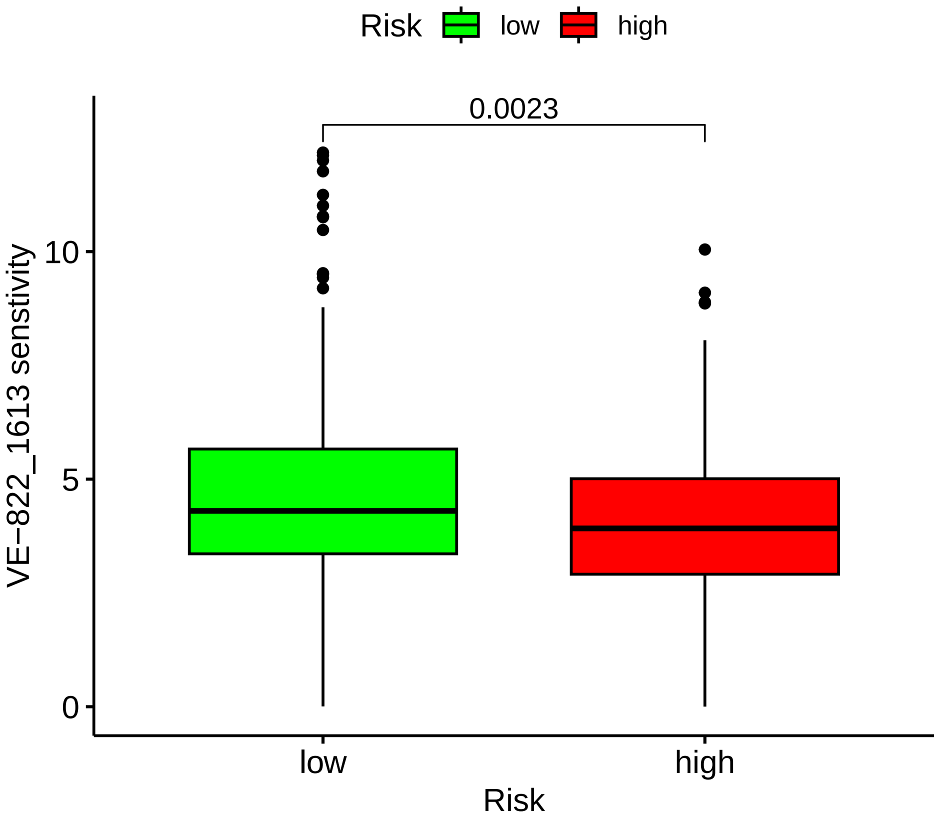
Risk
low
high
0.0023
VE−822_1613 senstivity
10
5
0
low
high
Risk

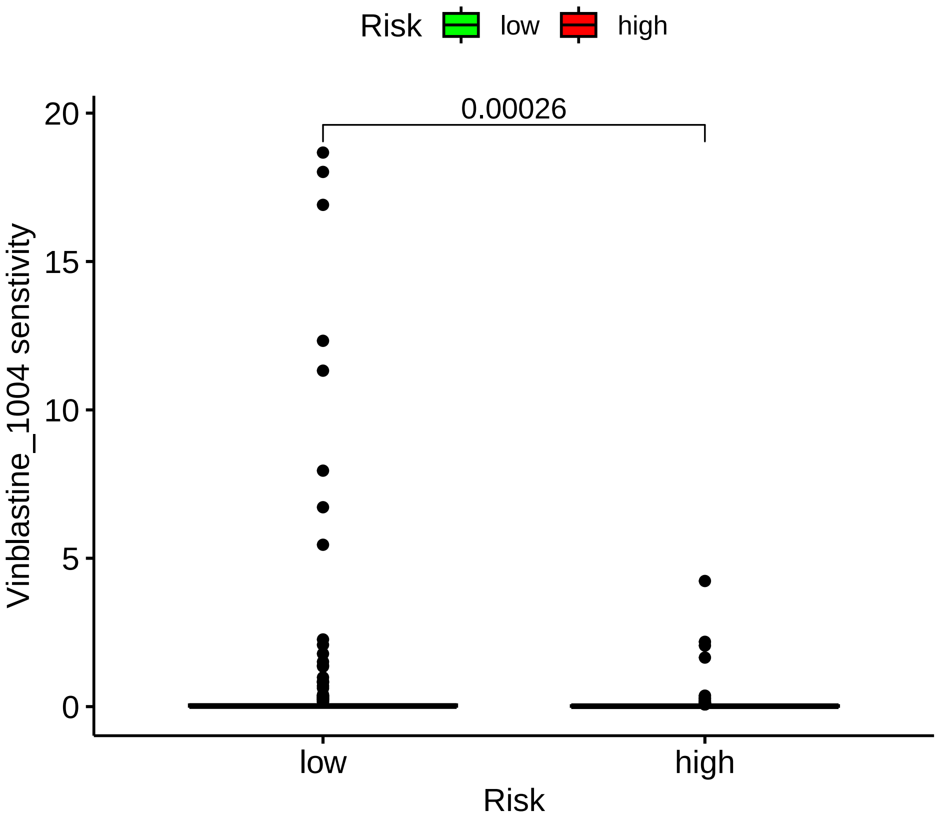
Risk
low
high
0.00026
Vinblastine_1004 senstivity
20
15
10
5
0
low
high
Risk

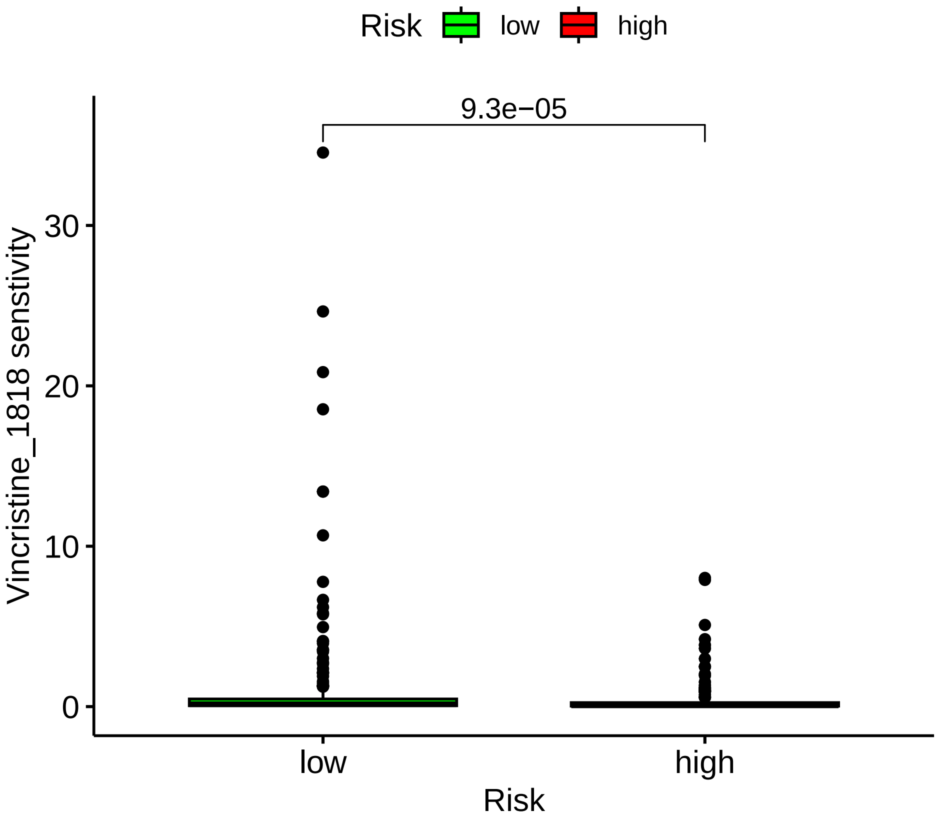
Risk
low
high
9.3e−05
Vincristine_1818 senstivity
30
20
10
0
low
high
Risk

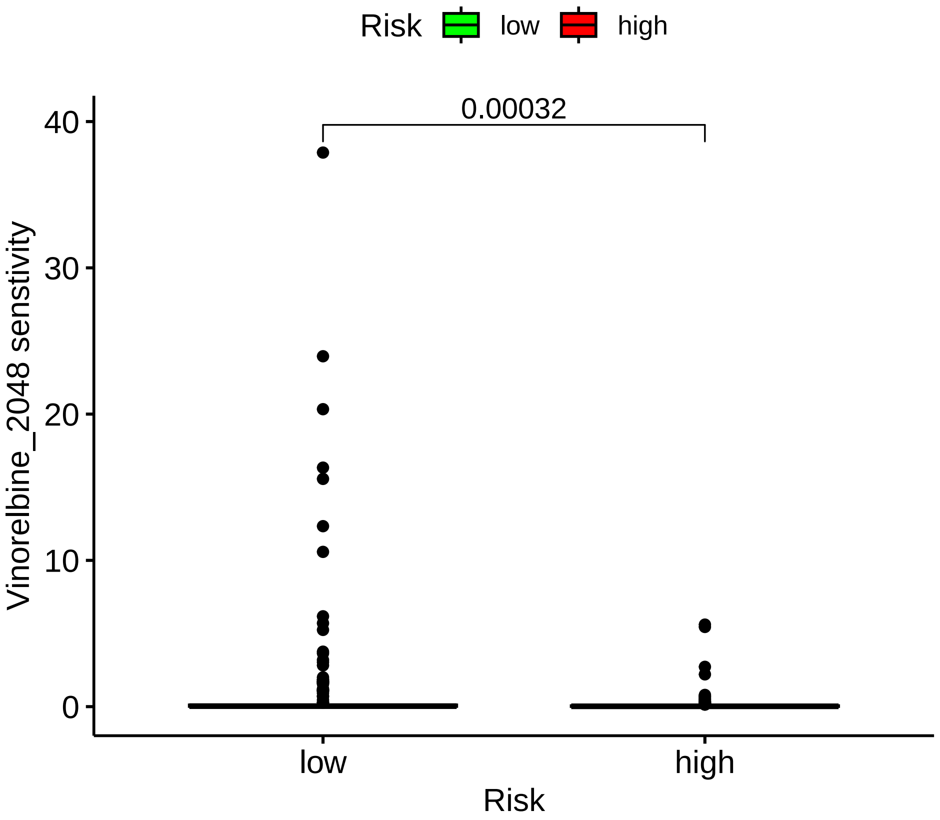
Risk
low
high
0.00032
Vinorelbine_2048 senstivity
40
30
20
10
0
low
high
Risk

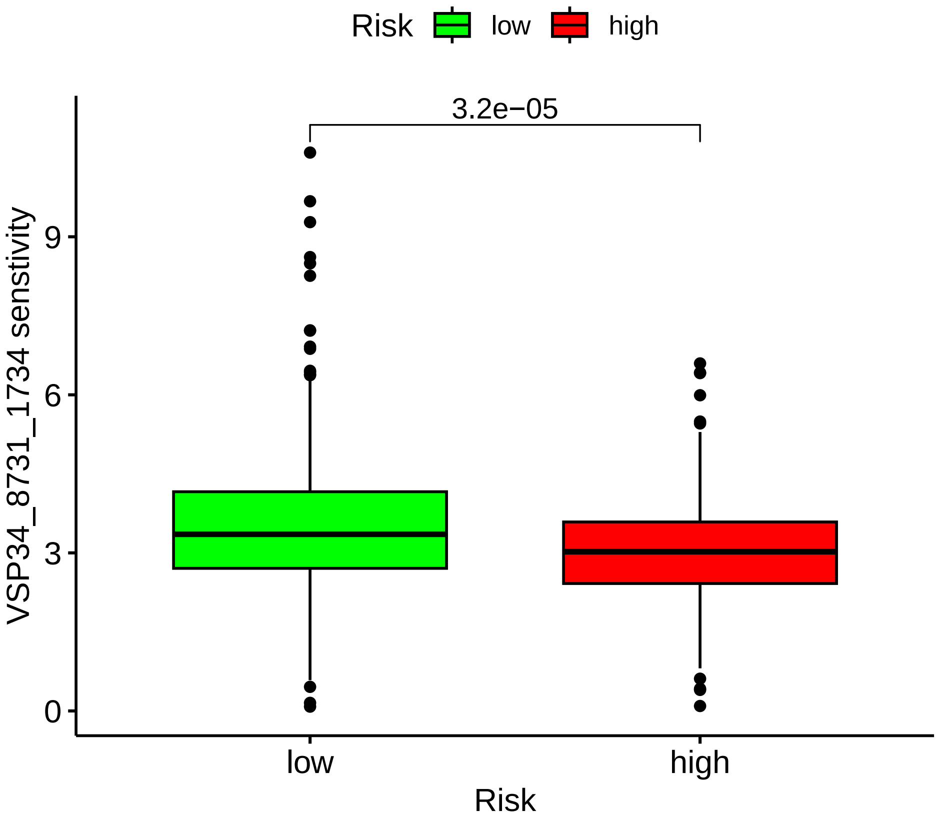

Risk
low
high
3.2e−05
VSP34_8731_1734 senstivity
0
3
6
9
low
high
Risk

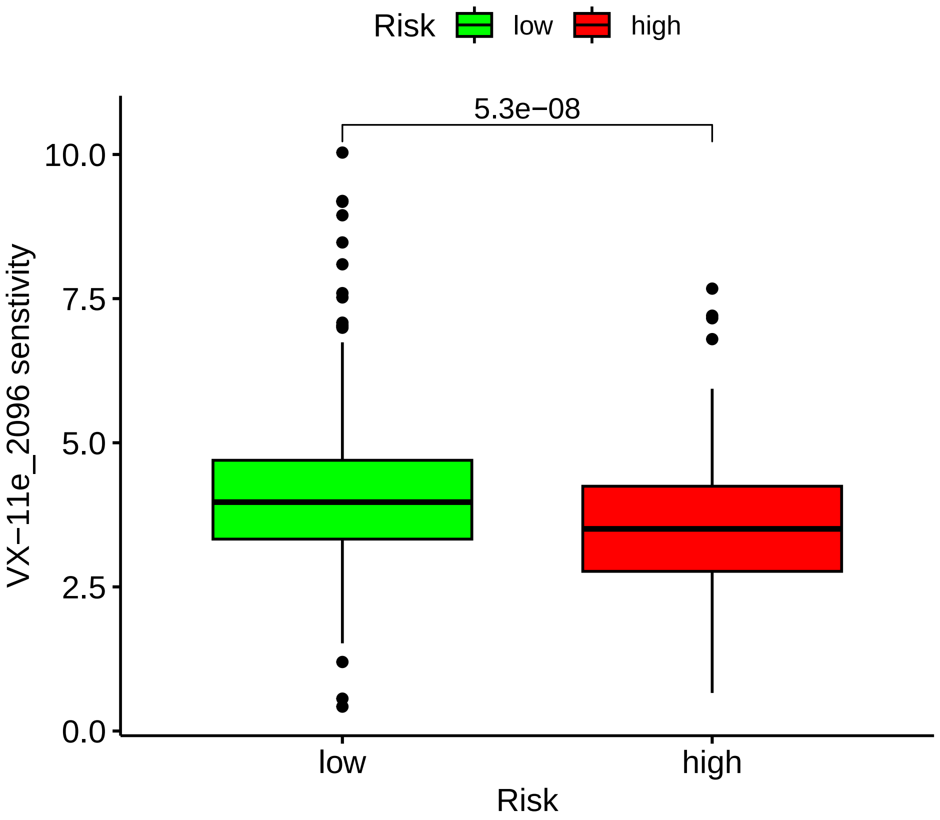

Risk
low
high
5.3e−08
10.0
7.5
5.0
2.5
0.0
VX−11e_2096 senstivity
low
high
Risk

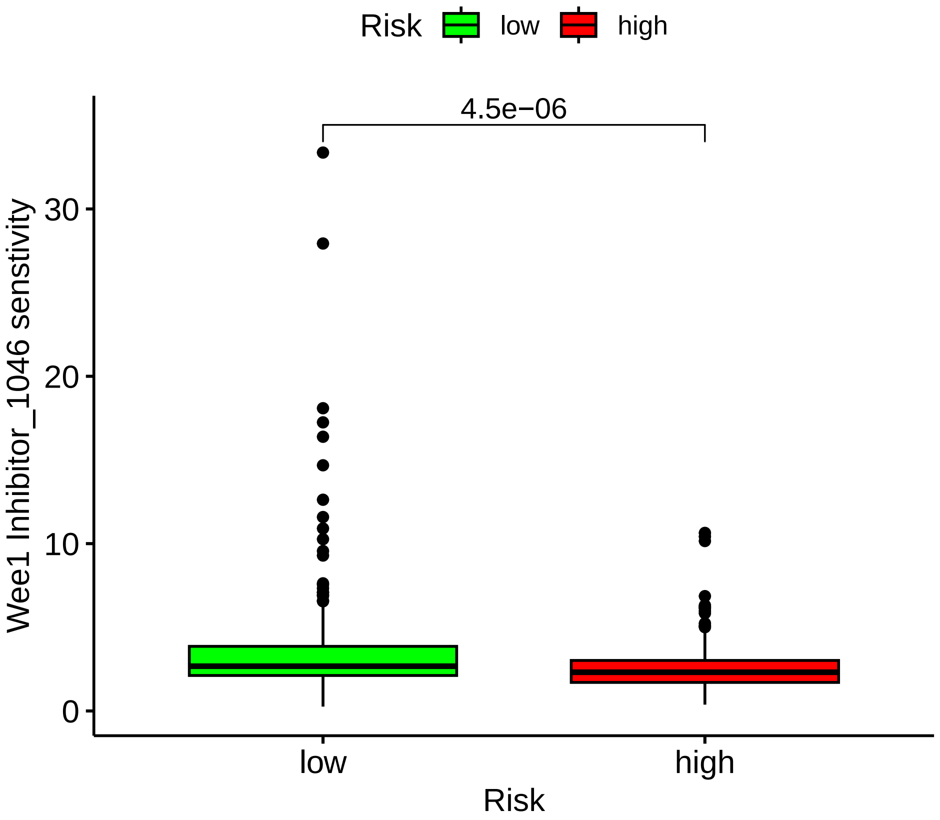
Risk
low
high
4.5e−06
Wee1 Inhibitor_1046 senstivity
30
20
10
0
low
high
Risk

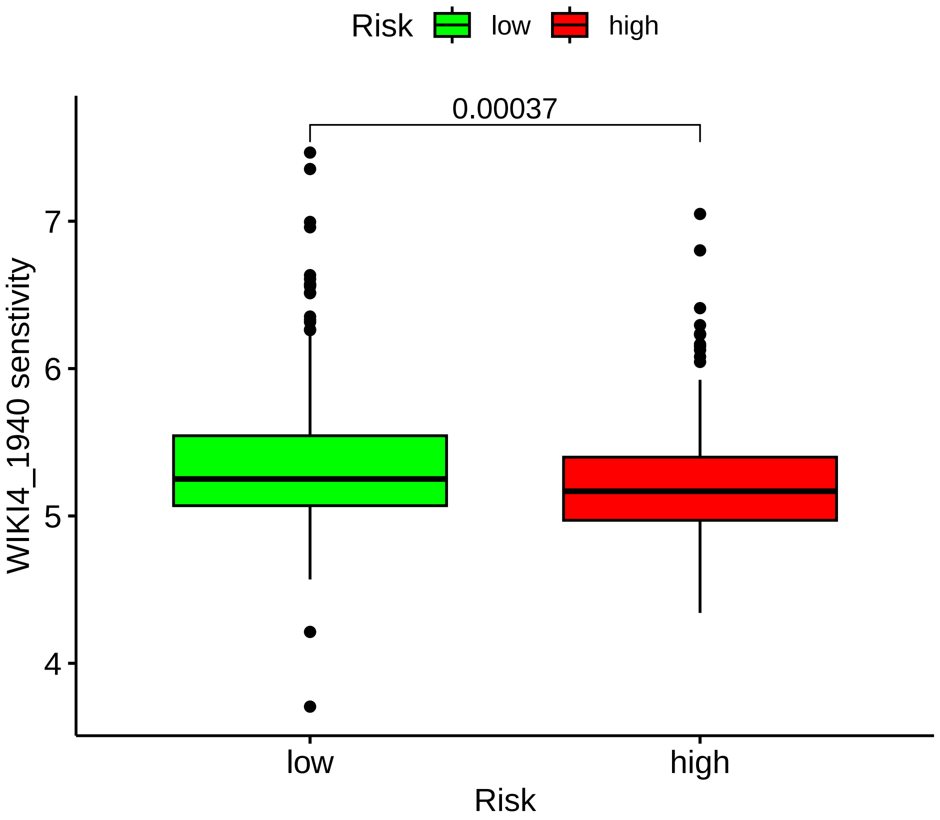
Risk
low
high
0.00037
WIKI4_1940 senstivity
7
6
5
4
low
high
Risk

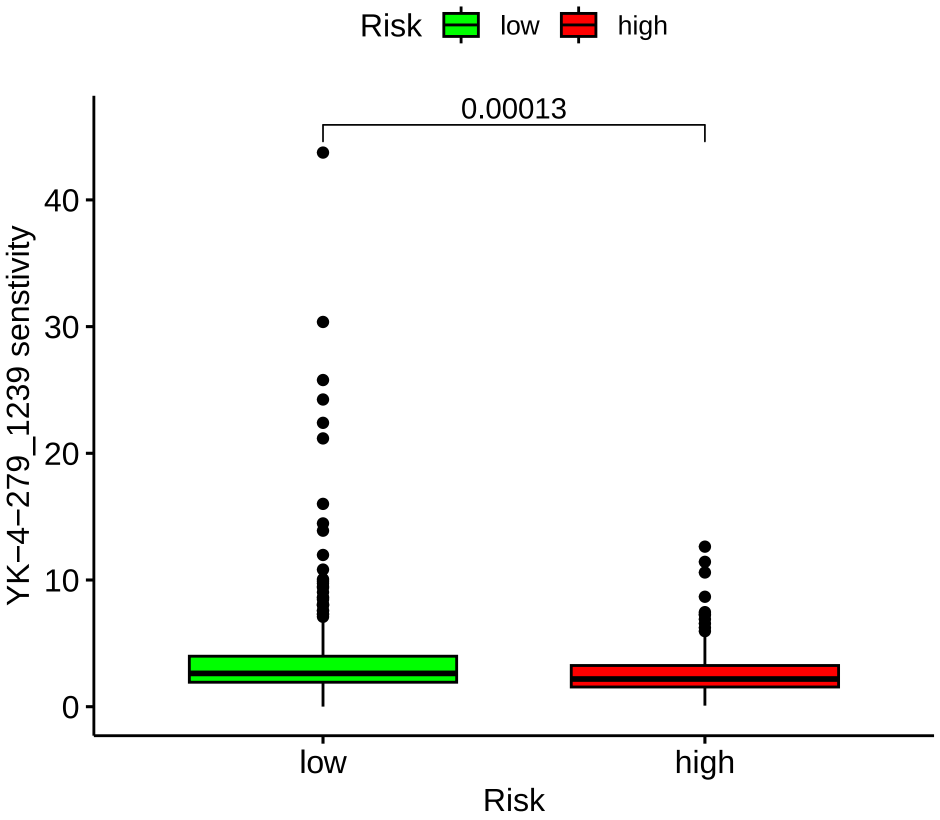

Risk
low
high
0.00013
YK−4−279_1239 senstivity
40
30
20
10
0
low
high
Risk

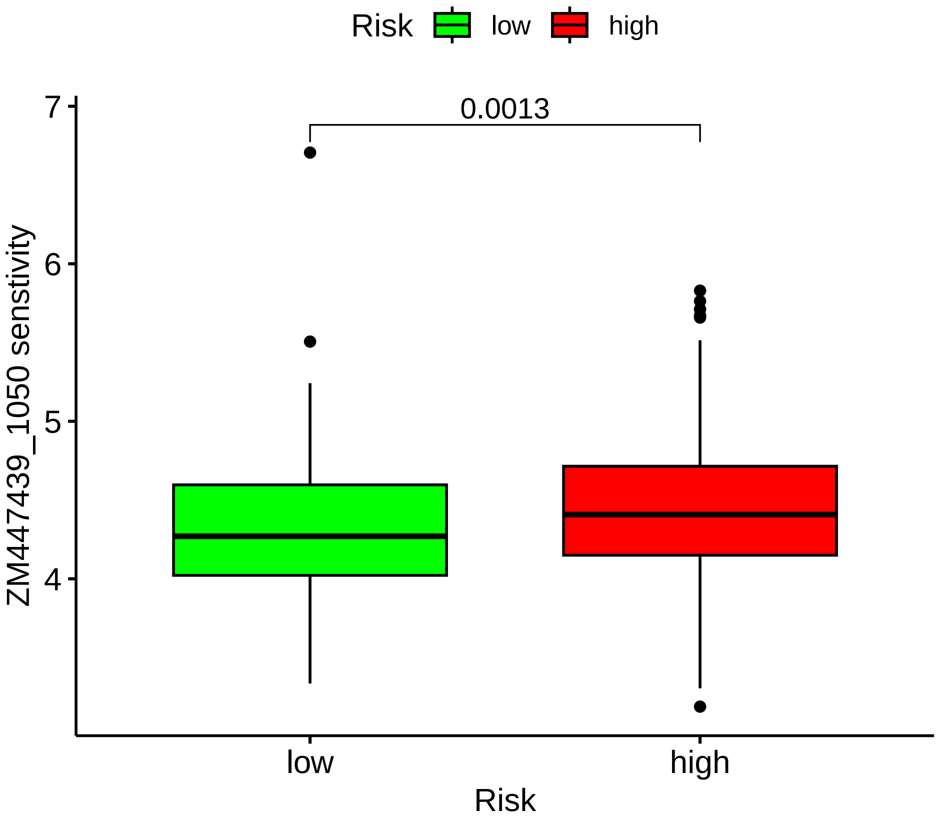

Risk
low
high
0.0013
7
6
5
4
ZM447439_1050 senstivity
low
high
Risk
