# Supplementary material for: Integrated analysis identifies a palmitoylation-associated prognostic model (ACSM5/SKA3) for lung adenocarcinoma across multiple cohorts
Source: PeerJ. 2026 Apr 29;14:e21160. doi: 10.7717/peerj.21160 (PMC13135332; doi:10.7717/peerj.21160)
Supplement: Supplemental Information 5 [file peerj-14-21160-s005.zip › peerj-125255-Supplementary_Figure_S3.pdf]

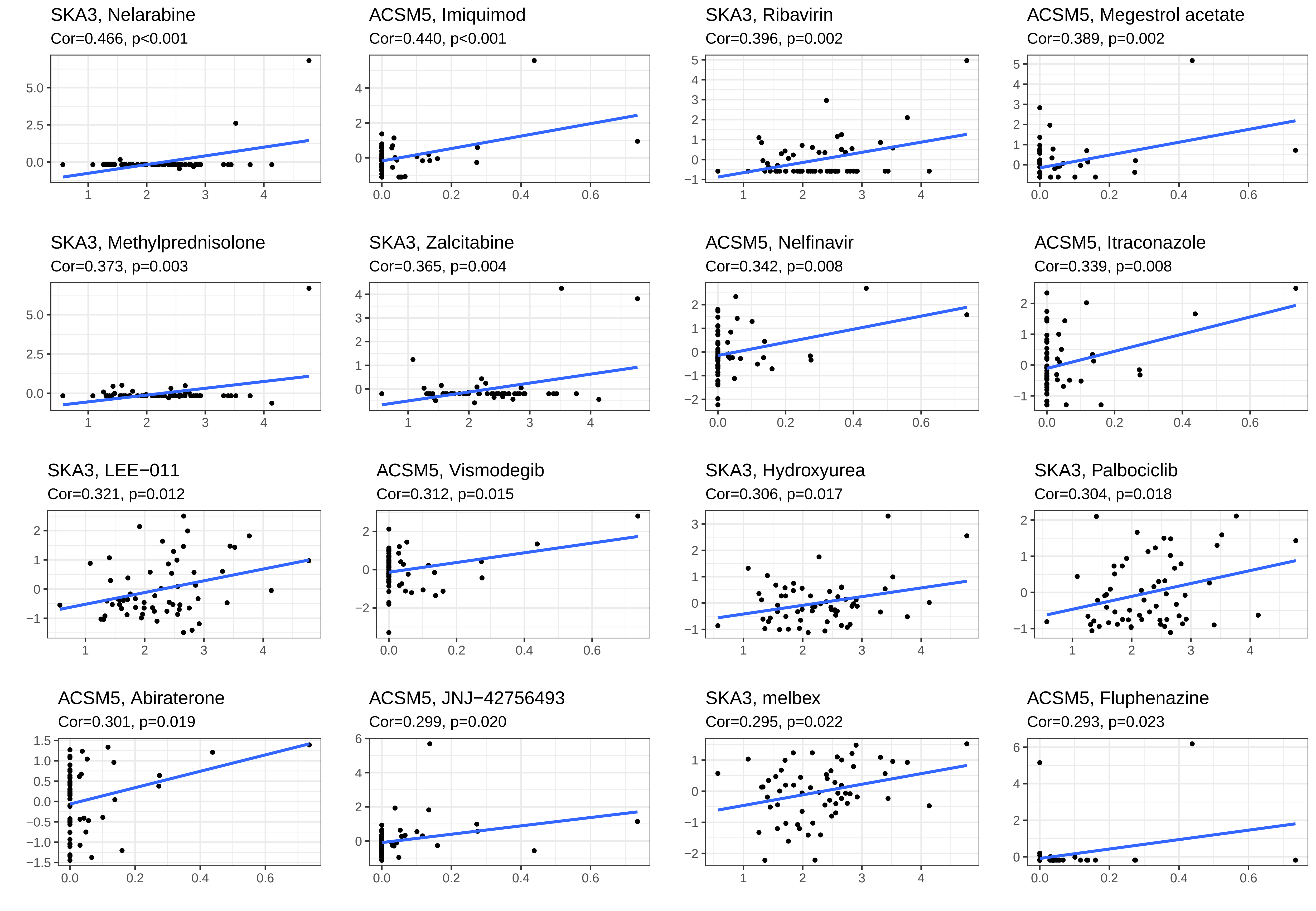
SKA3, Nelarabine
Cor=0.466, p<0.001
ACSM5, Imiquimod
Cor=0.440, p<0.001
SKA3, Ribavirin
Cor=0.396, p=0.002
ACSM5, Megestrol acetate
Cor=0.389, p=0.002
SKA3, Methylprednisolone
Cor=0.373, p=0.003
SKA3, Zalcitabine
Cor=0.365, p=0.004
ACSM5, Nelfinavir
Cor=0.342, p=0.008
ACSM5, Itraconazole
Cor=0.339, p=0.008
SKA3, LEE−011
Cor=0.321, p=0.012
ACSM5, Vismodegib
Cor=0.312, p=0.015
SKA3, Hydroxyurea
Cor=0.306, p=0.017
SKA3, Palbociclib
Cor=0.304, p=0.018
ACSM5, Abiraterone
Cor=0.301, p=0.019
ACSM5, JNJ−42756493
Cor=0.299, p=0.020
SKA3, melbex
Cor=0.295, p=0.022
ACSM5, Fluphenazine
Cor=0.293, p=0.023
