# Supplementary figures and images for: Integrated analysis identifies a palmitoylation-associated prognostic model (ACSM5/SKA3) for lung adenocarcinoma across multiple cohorts
Source: PeerJ. 2026 Apr 29;14:e21160. doi: 10.7717/peerj.21160 (PMC13135332; doi:10.7717/peerj.21160)

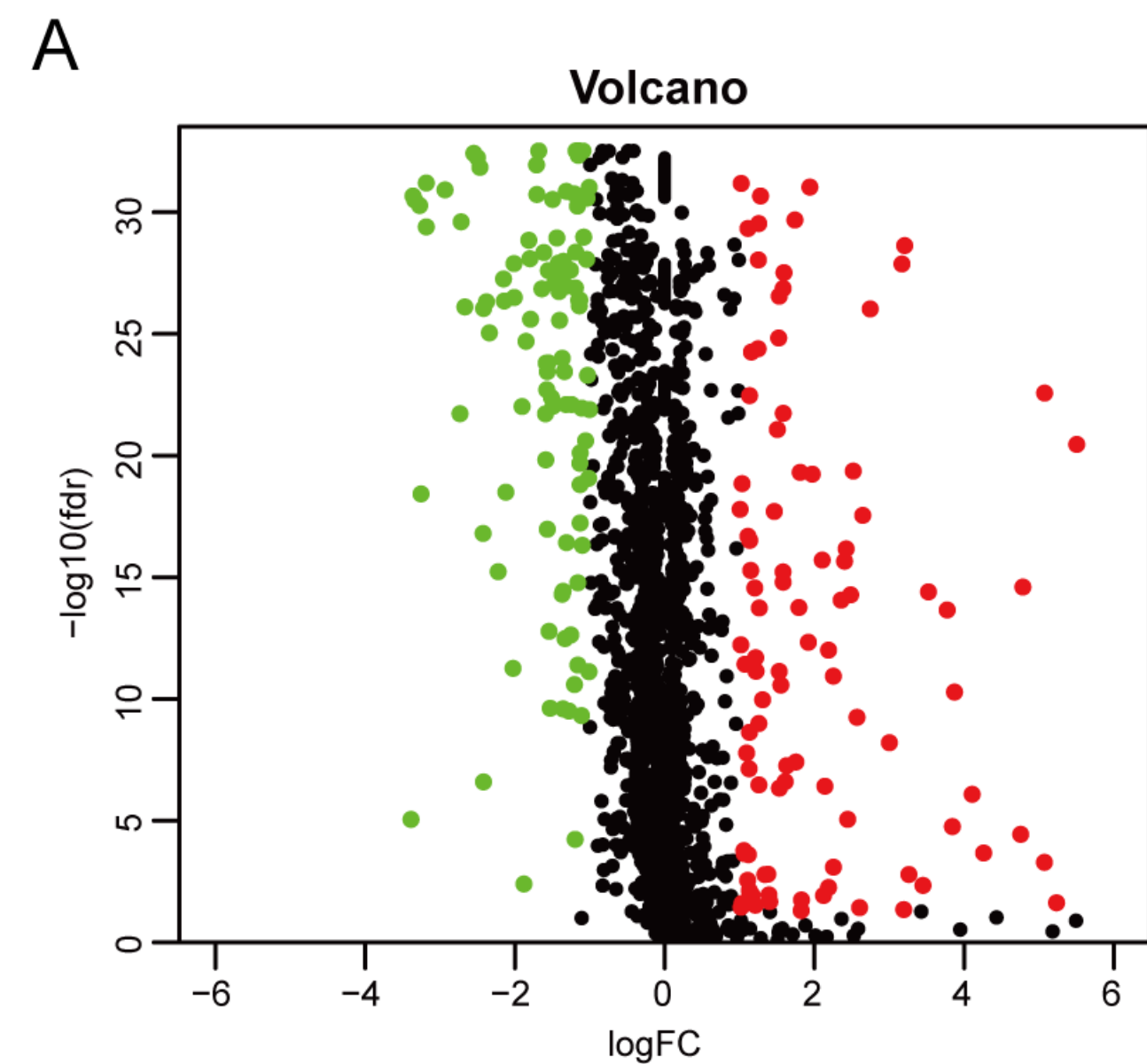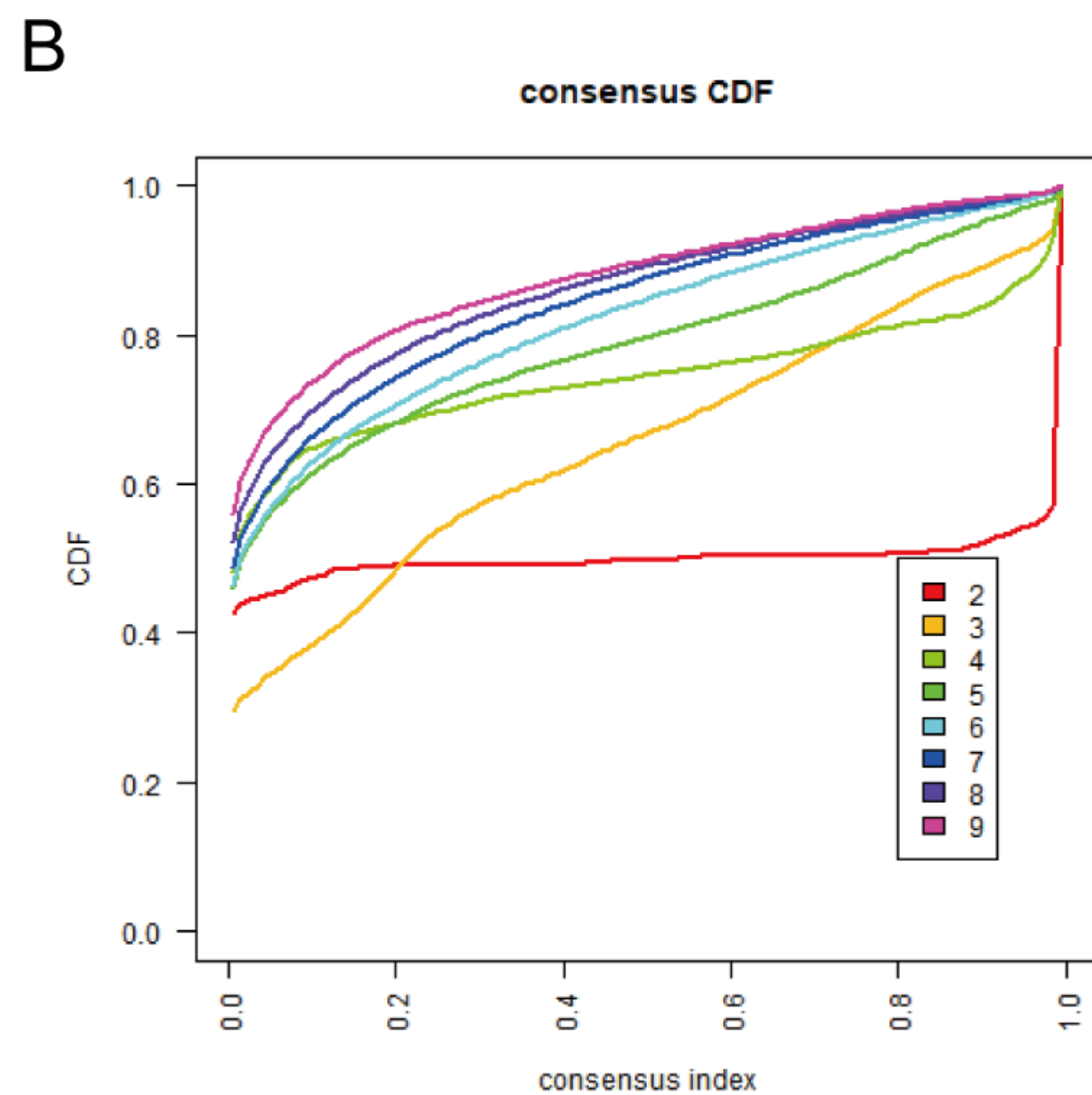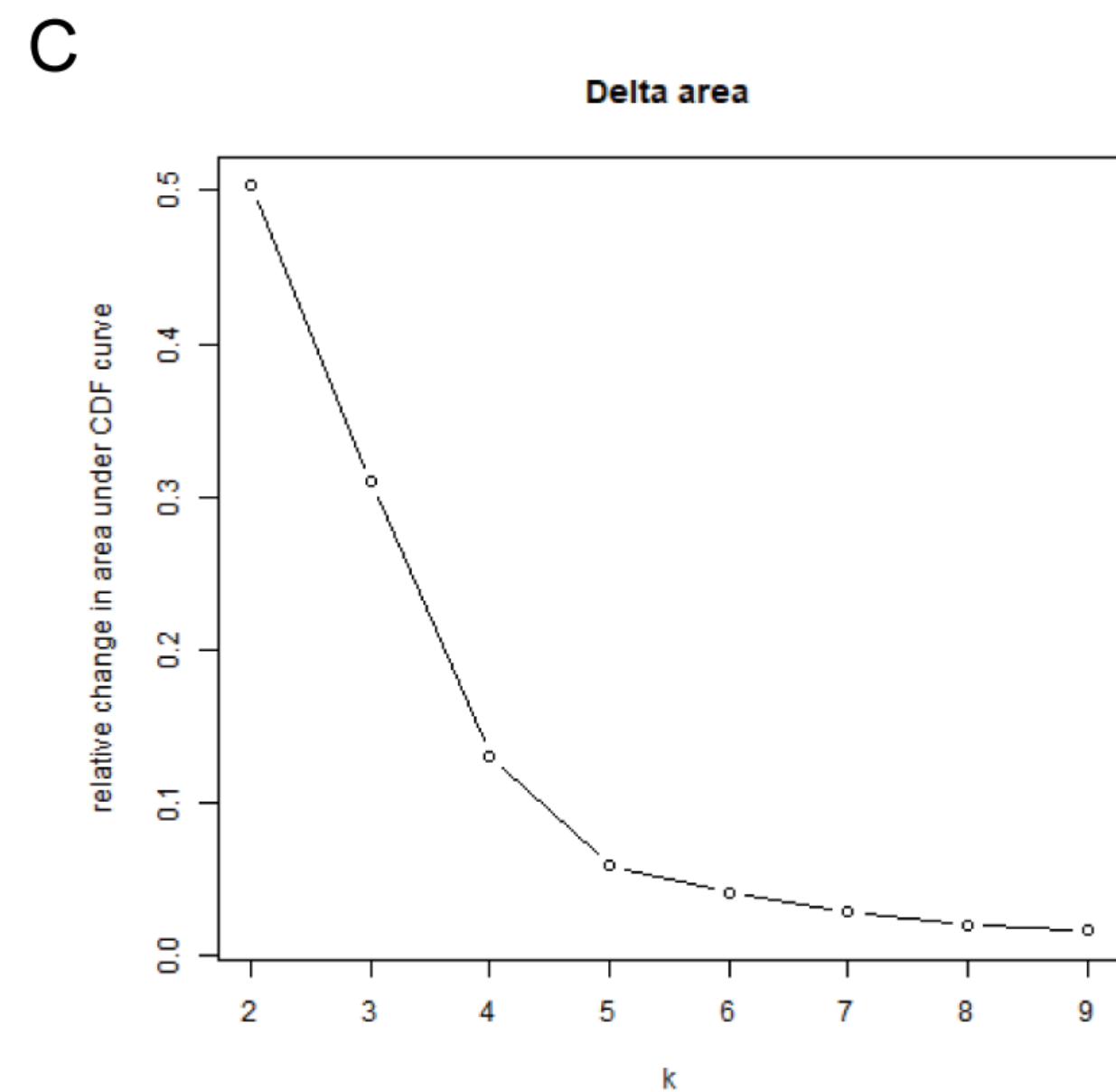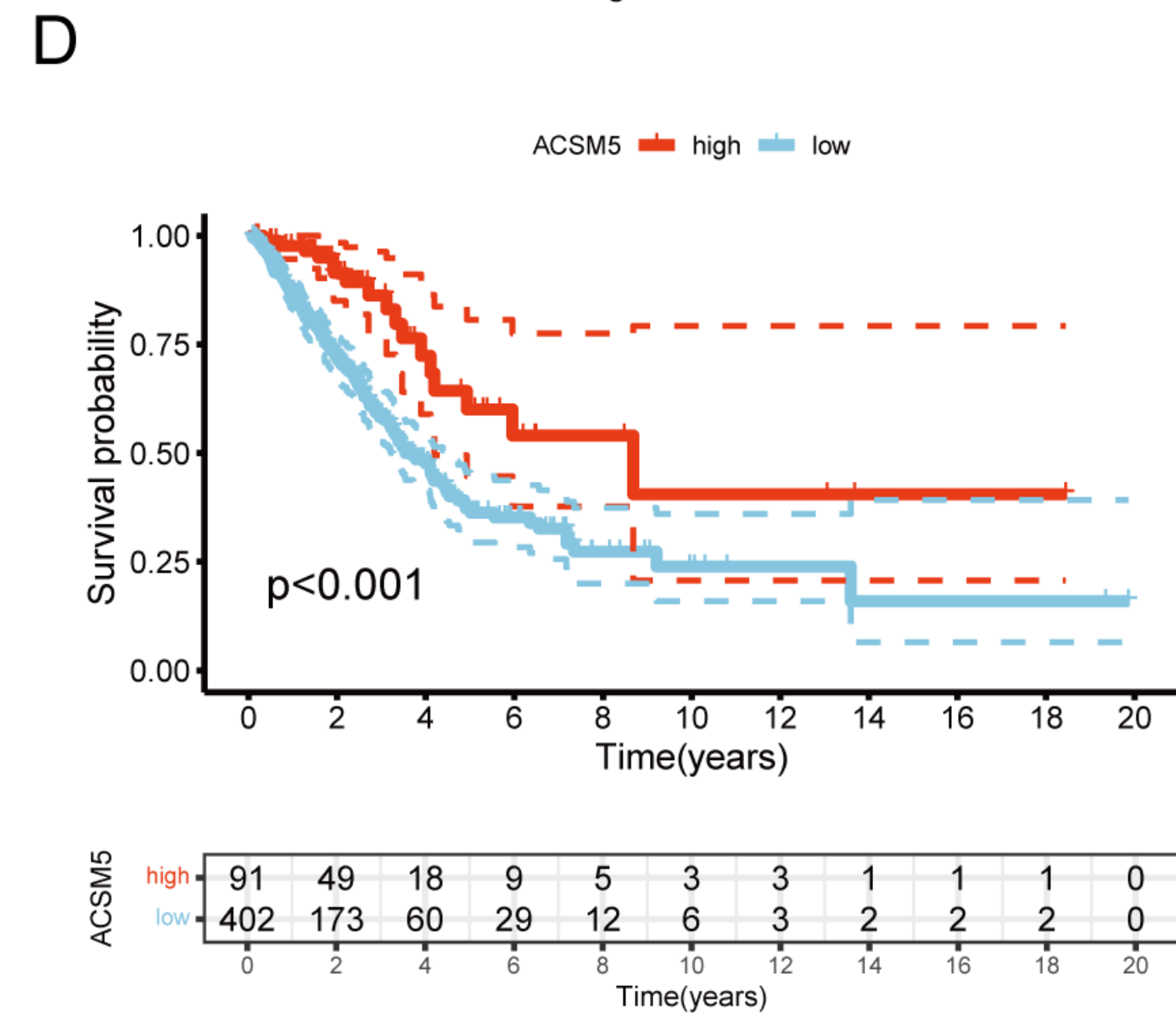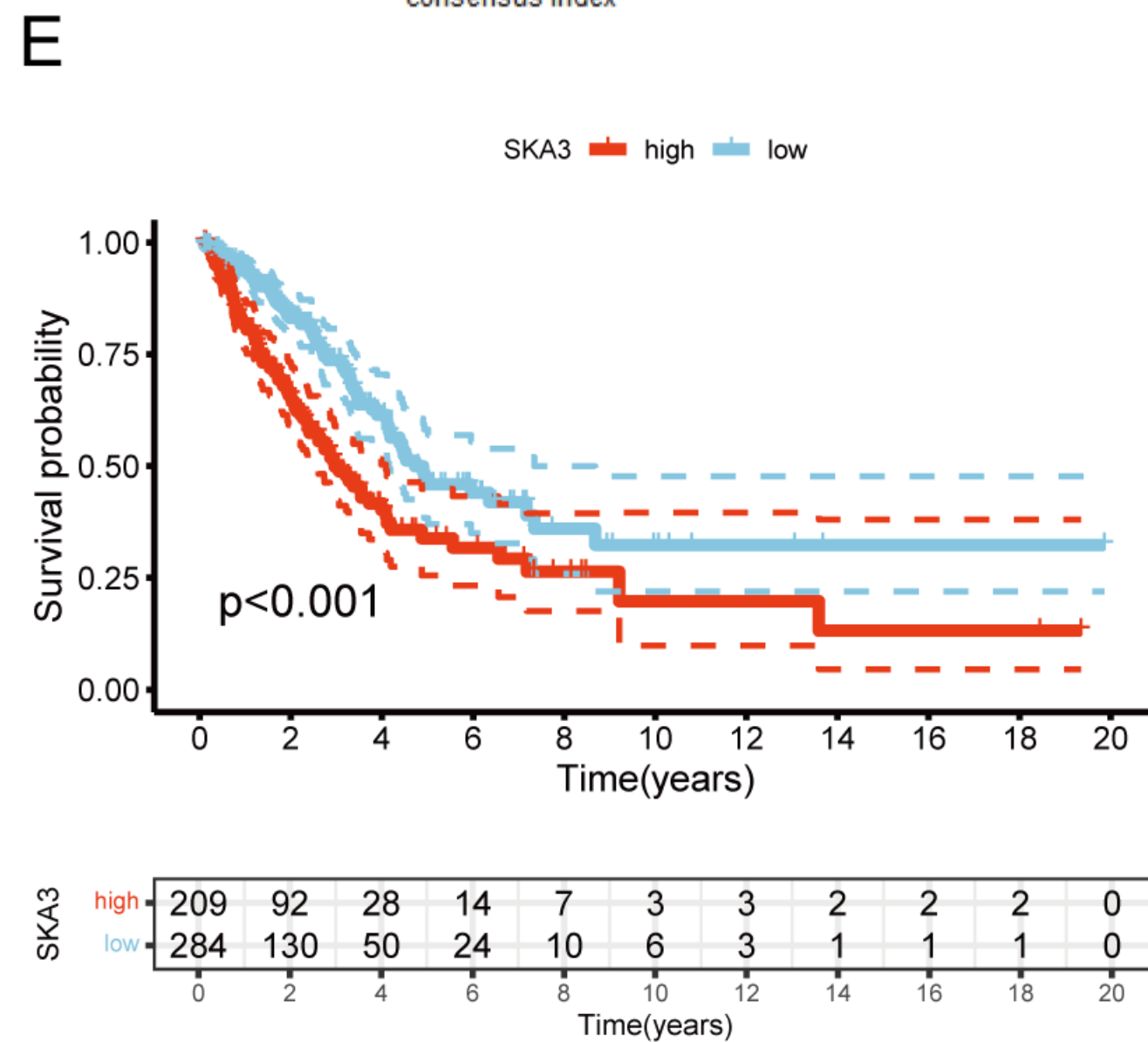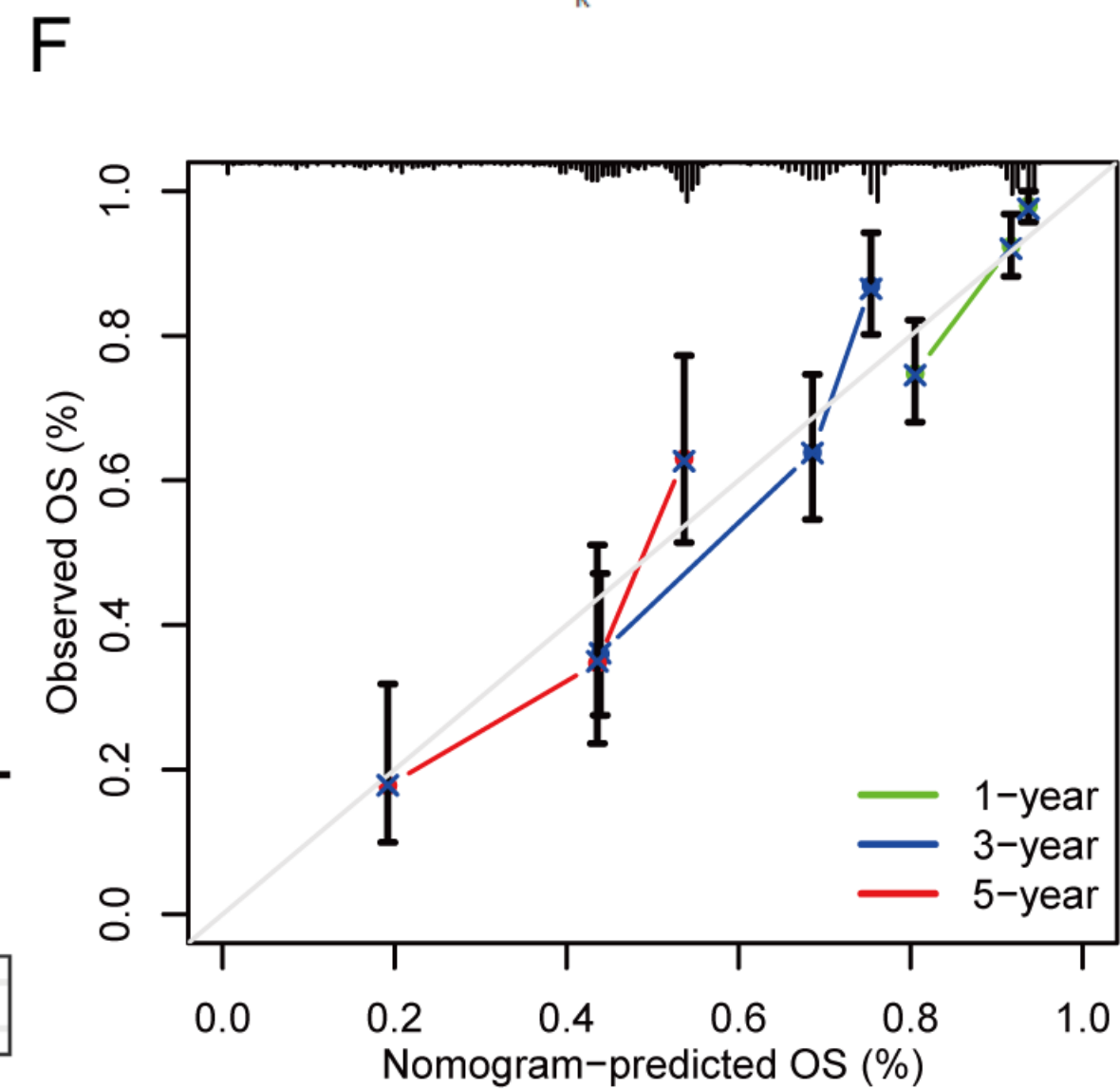

Supplement: Supplemental Information 5 [file peerj-14-21160-s005.zip › peerj-125255-Supplementary_Figure_S1.pdf]
